# Optimization of Pyrazole-Based Activators of Kv3.1

**DOI:** 10.1021/acsmedchemlett.6c00272

**Published:** 2026-06-25

**Authors:** Paul K. Spearing, Vaishali Satpute Janve, Ian M. Romaine, Navoda Jayakodiarachchi, Benjamin W. Guttentag, Somnath Jana, Kwangho Kim, Jerod S. Denton, Craig W. Lindsley, Alex G. Waterson

**Affiliations:** ‡ Warren Center for Neuroscience Drug Discovery, 5718 Vanderbilt University, Nashville, Tennessee 37232, United States; § Department of Anesthesiology, 12328 Vanderbilt University Medical Center, Nashville, Tennessee 37232, United States; ∥ Vanderbilt Institute of Chemical Biology, 5718 Vanderbilt University, Nashville, Tennessee 37232, United States; ⊥ Department of Pharmacology, 12327 Vanderbilt University School of Medicine, Nashville, Tennessee 37232, United States; # Department of Chemistry, 5718 Vanderbilt University, Nashville, Tennessee 37232, United States

**Keywords:** potassium channel, Kv3.1 activation, ion channel research, CNS drug discovery

## Abstract

Potentiators of the Kv3.1 voltage-gated potassium channel may have utility as therapies for certain CNS disorders. A SAR survey around a previously reported Kv3.1 activator scaffold led to the identification of compounds with superior potency and higher activation levels than reported molecules. We further demonstrated that these compounds show relevant activity in a patch-clamp electrophysiology assay. Together, the defined tractability in the chemotype and the promising biological activity indicate that these compounds may be leads for continued optimization toward therapies for neurological disorders.

## Graphical abstract

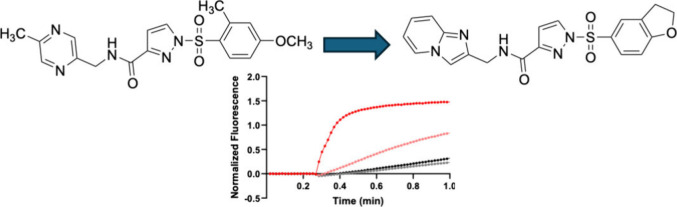

## Introduction

Voltage-gated potassium (Kv) channels regulate neuronal excitability by restoring the resting membrane potential following action potential depolarizations. The voltage-gated potassium channel subfamily C (Kv3) family (Kv3.1–3.4) is distinguished by a high activation threshold (> –20 mV) and ultrafast activation and deactivation kinetics.^1–3^ These properties enable rapid membrane repolarization and brief refractory periods that support high-frequency action potential firing. Particularly, the Kv3.1 channels are essential for generation and synchronization of high-frequency neuronal oscillations.^2,4–6^ High-frequency network oscillations mediate critical functions, including cognition, learning and memory, sensory information processing, and perception.^7–11^ Given this significant role, Kv3.1 hypofunction has been linked to several neurological disorders, including epilepsy, autism, schizophrenia, anxiety, and hearing disorders.^12–16^ Further, positive allosteric modulators of Kv3.1 channels have demonstrated efficacy in improving disease-related symptoms in rodent disease models of fragile X syndrome, schizophrenia, and bipolar mania-like behaviors.^17–19^ Thus, Kv3.1 activators are being pursued as promising targets for neurological disorders. Additionally, Kv3.1 channels are enriched in the central nervous system (CNS) and predominantly expressed in the fast-spiking parvalbumin-containing GABAergic interneurons,^20–25^ making them an attractive target for the discovery of CNS therapies.

## Kv3.1 Activator Landscape

Several potentiators of Kv3.1 have been previously reported. Pyridine-substituted imidazolinediones, including AUT1 (**1**) (Figure 1), were reported to modulate both Kv3.1 and Kv3.2 in neurons and cells overexpressing the channels, inducing hyperpolarizing shifts in the activation voltage dependence in both settings.^26,27^ A mechanistic study of the action of a related molecule, AUT5 (**2**), revealed that the activity of these compounds was based on stabilization of an open conformation of the channel, which, based on the cryo-EM structure of AUT5 bound to Kv3.1, is due to compound-induced rearrangements in the turret regions.^28^ Compound **4** (**3**), from a similar overall scaffold but bearing a variation on the imidazolinedione section of the AUT molecules, has likewise been found to activate the Kv3 channels and was, like AUT5, determined by cryo-EM to bind near the extracellular portion of the channel, in the turret region.^29^

**Figure 1:**
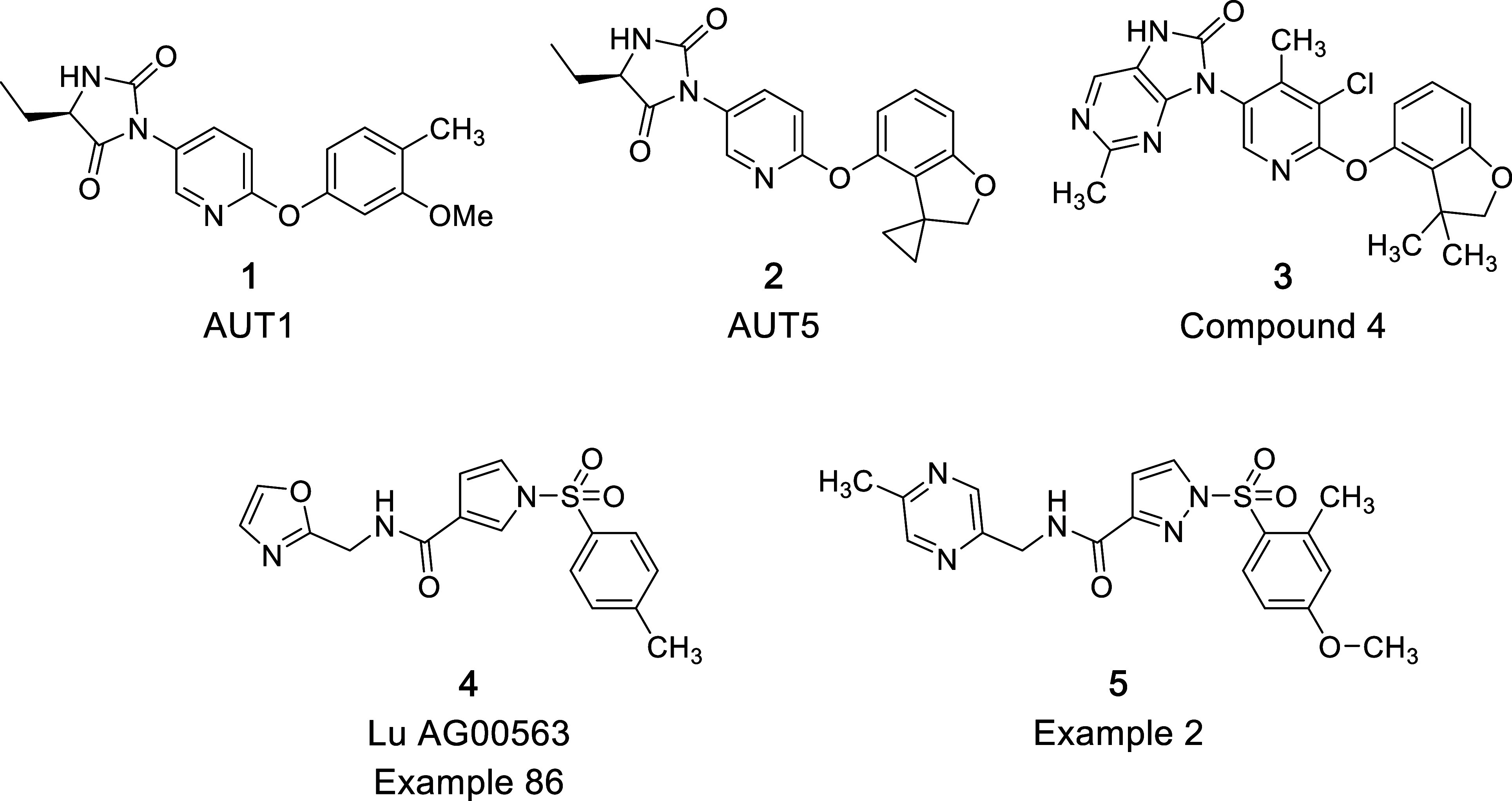
Selected previously reported activators of Kv3.1.

In contrast, pyrrole LuAG00563 (**4**),^30^ which is also a potentiator of Kv3.1 function, was found to bind to a different portion of the protein.^31^ This compound binds between the voltage-sensor domain and the pore of the channel, closer to the region in which many disease-associated mutations occur. The binding mode implies a mode of action different from the compounds that bind the turret regions. A closely related set of compounds bearing a central pyrazole has also been described in the patent literature^32^ as activators of Kv3.1. This work is highlighted by reported example 2 (**5**) (Figure 1), the most potent example in the series.

Intrigued by the novel binding mode and mechanism of Lu A00563, which likely extends to example 2, we further examine the potential of this highly modular chemotype in search of drug-like Kv3.1 activators with superior potency to the reported examples.

## Assay Development

To enable characterization of the analogues, a monoclonal cell line expressing human Kv3.1 channels was generated (see the Supporting Information). We used tetracycline-regulated expression HEK-293 (T-REx-HEK-293) cells, which allow tetracycline-inducible expression of Kv3.1 channels prior to the assay but prevent channel overexpression and cell line degeneration over time. The Kv3.1 cell line was characterized by using the gold standard patch-clamp electrophysiology assay. Large voltage-dependent outwardly rectifying currents were observed with depolarizing current steps in Kv3.1 monoclonal cells (Figure 2B and D), whereas parental T-REx-HEK-293 cells exhibited only small-amplitude outward currents typical of endogenous Kv currents (Figure 2A and D). Kv3.1 currents had slow inactivation with sustained depolarization and were partly inhibited with 2 mM non-specific potassium channel blocker tetraethylammonium chloride (TEAC) (Figure 2C and D). Figure 2D shows the current-to-voltage (*I*–*V*) curve with large outward currents with the activation voltage of approximately −40 mV. Thus, the Kv3.1 monoclonal cell line exhibits electrophysiological properties that are characteristic of Kv3.1 channels.

**Figure 2:**
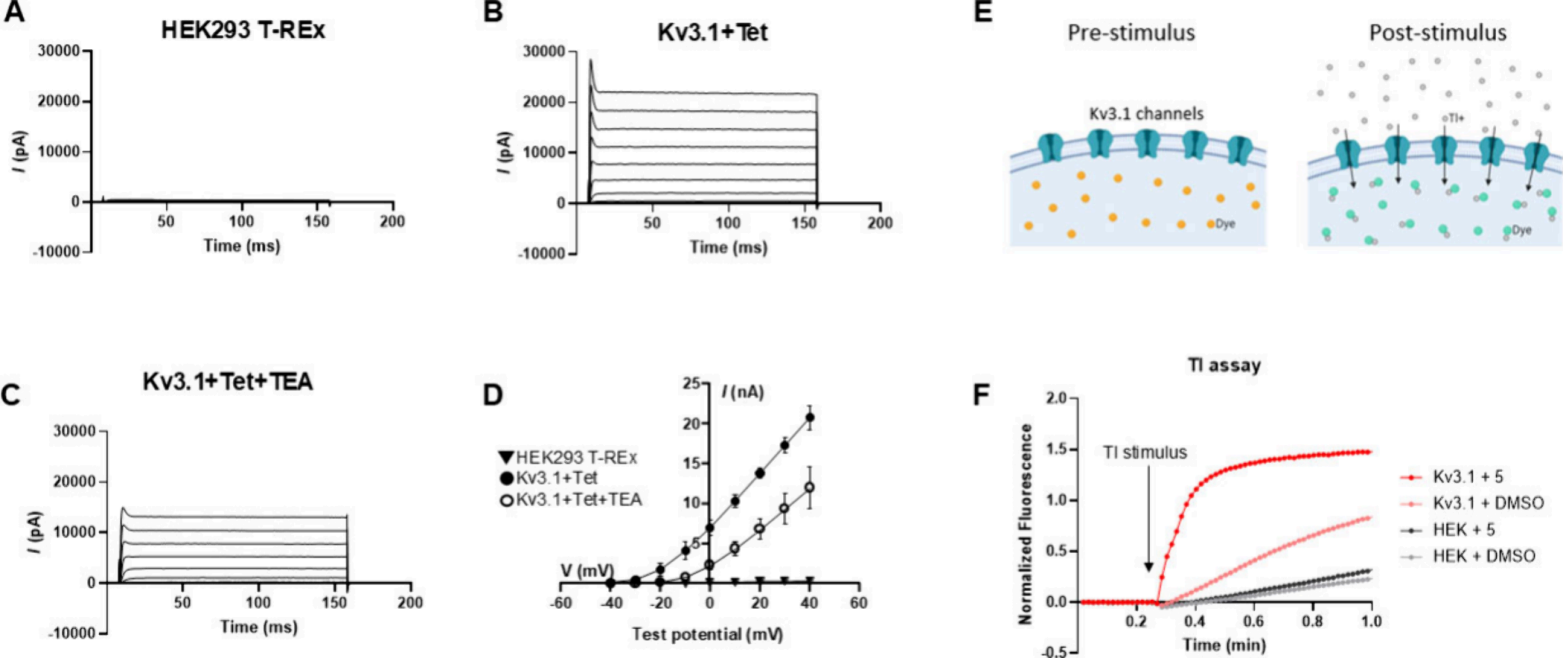
Discovery of Kv3.1 activators using a Kv3.1 monoclonal cell line in a high-throughput quantitative Tl^+^ assay. (A, B, and C) Representative current traces from whole cell voltage clamp recordings. Large outward rectifying currents were observed with depolarizing steps in Kv3.1 monoclonal cells (B) but were absent in the parental HEK293 T-REx cell (A). Non-specific potassium channel blocker TEAC partially inhibited the outward currents in Kv3.1 cells (C). (D) *I*–*V* curves show the activation voltage of approximately −40 mV, typical for Kv3.1 channels. Partial current inhibition with 2 mM TEAC results in the right shifted curve with activation around −10 mV. The parental HEK293 T-REx cells exhibit significantly smaller currents and lack pronounced outward currents compared to Kv3.1 cells. (E) Schematic of the Tl^+^ assay shows Thallos AM dye (orange circles) loaded in cells expressing Kv3.1 channels. Stimulation with Tl^+^ (gray circles) buffer leads to a rapid influx of Tl^+^ ions via Kv3.1 channels and a sudden change in the fluorescence signal upon binding to the dye. (F) Representative fluorescence signal before and after Tl^+^ stimulus. In Kv3.1 cells, preincubation with the activator **5** (10 μM) produces a sudden increase in the fluorescence signal after Tl^+^ stimulation compared to incubation with the DMSO vehicle control, while parental HEK293T cells had a small and linear increase in the fluorescence signal with a similar magnitude with and without **5**.

Next, we developed a robust thallium (Tl^+^) flux assay that enables quantitative fluorescence imaging and high-throughput functional screens of analogues and concentration response measurements. Kv3.1-expressing cells were incubated with the Tl^+^-sensitive Thallos AM dye, which readily permeates the cell membrane and remains quenched until fluorescence is triggered upon Tl^+^ binding (Figure 2E). The rapid increase in the fluorescence intensity upon external application and inward flux of Tl^+^ serves as a surrogate of channel opening and Kv3.1-mediated Tl^+^ influx (Figure 2F).

## Structure–Activity Relationships

We hypothesized that **4** and **5** are part of a larger shared chemotype and, thus, that this chemotype would tolerate changes in the central heterocycle. On the basis of that hypothesis, we began our search for improved Kv3.1 activators by synthesizing a diverse set of five- and six-membered heterocycles, using flanking substitutions found in reported activators (Table 1).

**Table 1:**
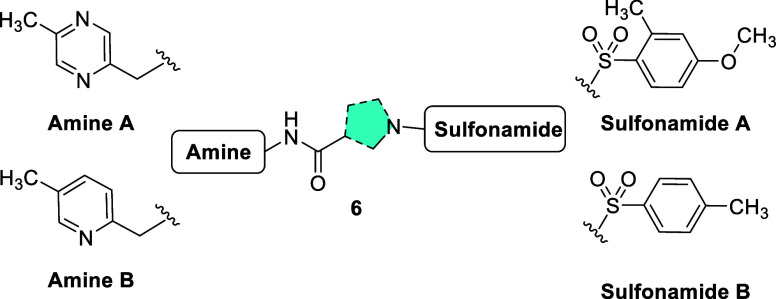

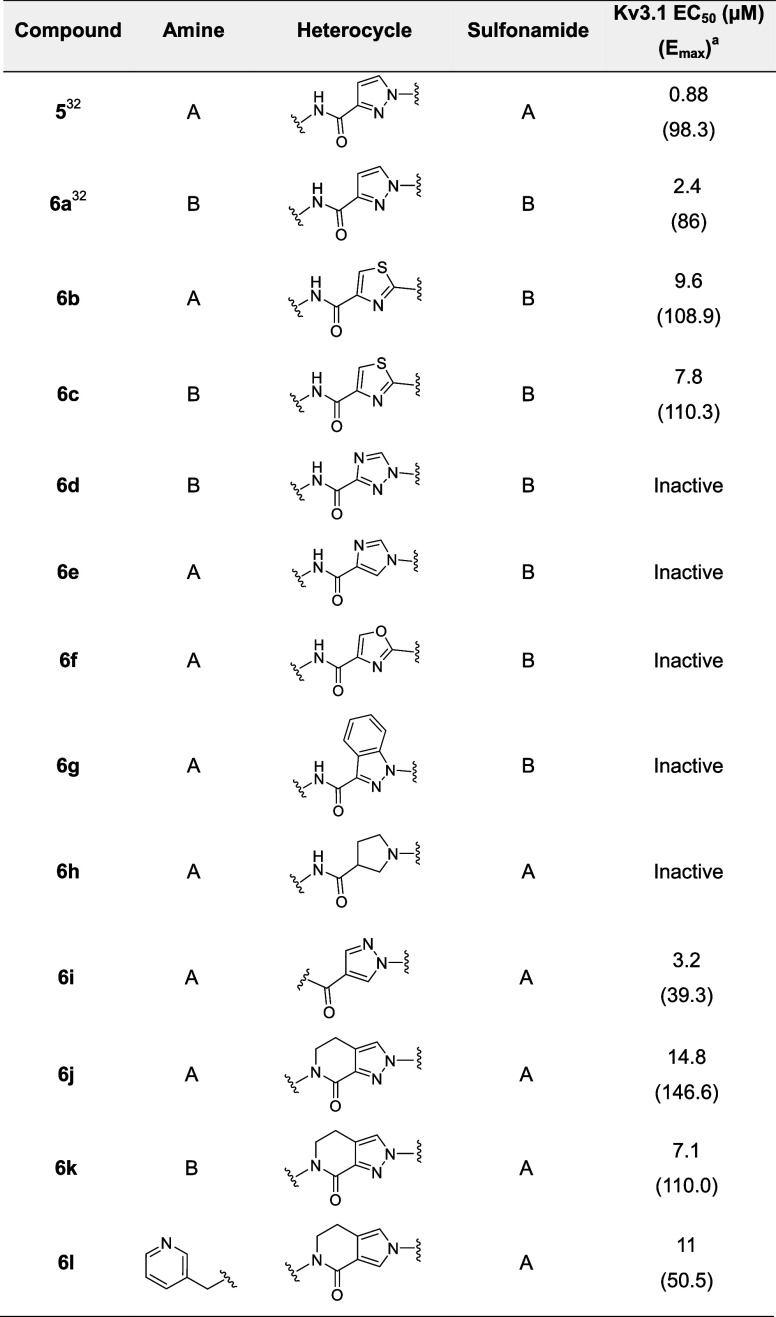
Investigation of Central Heterocycle Alternatives

a Determined in Tl^+^ flux assays. EC_50_ values represent a mean of three replicates. *E*_max_ values represent the maximal fluorescence response normalized to 15 μM **5** (set as 100% activity, with DMSO set as 0% activity). Inactive = EC_50_ could not be derived due to no activation or minimal activity observed at concentrations from 3 nM to 30 μM.

Previously reported **5** ^32^ was found to display a Kv3.1 activation EC_50_ of just under 1 μM in the thallium flux assay (Table 1). Application of an alternative amine/sulfonamide pair led to **6a**,^32^ with 3-fold reduced potency. These two molecules served as a baseline for additional changes. To compare both the potency and overall activation of additional analogues, **5** was included in all assay runs. The average activation *E*_max_ of all analogues was thus determined as a percentage of the activation observed with 15 μM **5**.

We first evaluated changes to the central pyrazole using the amines and sulfonamides from **5** and **6a** (Table 1). In general, most changes produced molecules with a lower Kv3.1 activation. For example, thiazoles **6b** and **6c** showed reduced potency of 10- and 3-fold compared to **5** and **6a**, respectively. Other heterocyclic cores fared worse; triazole **6d**, imidazole **6e**, and oxazole **6f** were inactive. Even the rather conservative addition of an aromatic ring to indazole **6g** was not tolerated. Further, saturated analogue **6h** was inactive, as were 6-membered rings (not shown). Pyrazole regiomer **6i**, however, retained activity, albeit with a 3-fold lower EC_50_ and a dramatically reduced maximal effect compared to those of **5**. On the basis of these results, it seems clear that the geometric and electronic requirements for activity are quite specific. Given this narrow SAR, we postulated that we might enforce geometrical constraints on the compounds to restrict rotational freedom and thereby increase activity. Analysis of the reported structure of **4**/Lu A00563 bound to Kv3.1 ^31^ led us to attempt to replace the amide proton with a tether to the core heterocycle (yellow arrow, Figure 3). However, the constrained analogues, including tetrahydropyrazolopyridinones **6j** and **6k** as well as tetrahydropyrrolopyridinone **6l**, all displayed sharply reduced activation of Kv3.1. On the basis of these accumulated results, we moved forward with a SAR study on the peripheral portions of the scaffold, retaining the pyrazole core.

**Figure 3:**
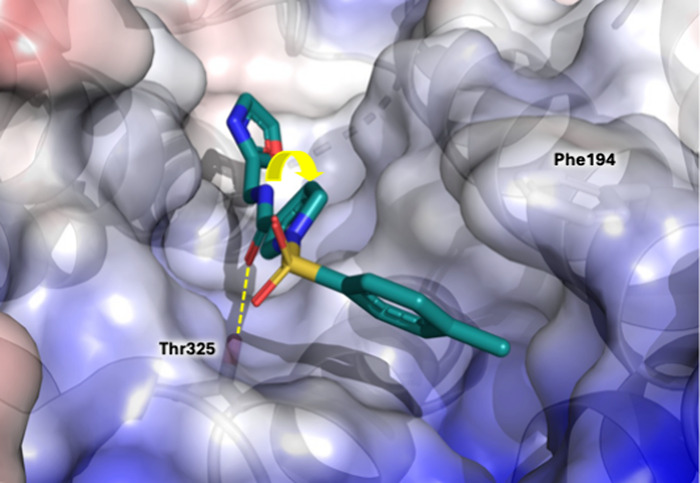
Structure of Lu AG00563 bound to Kv3.1 (7PQU) inspired rigidification of the compound around the central heterocyclic core.

Crossover analogues **7a** and **8a** (Table 2) were synthesized and evaluated. The activity of these analogues demonstrates that a pyridine-based amine shows only a minor drop in potency compared to a diazine (compare **7a** to **5** and **6a** to **8a**). Likewise, a small decrease in potency is observed with the use of methylsulfonamide vs methylmethoxysulfonamide (compare **5** to **8a** and **7a** to **6a**). Within this SAR context, changes to the C-3 position of the scaffold were evaluated (Table 2), with changes made using either 2-methyl, 4-methoxyphenyl sulfonamide (analogues **7**) and/or 4-methylphenyl sulfonamide (analogues **8**).

**Table 2:**
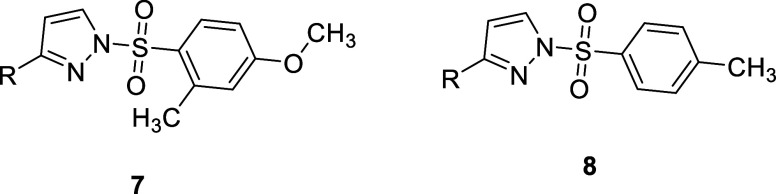

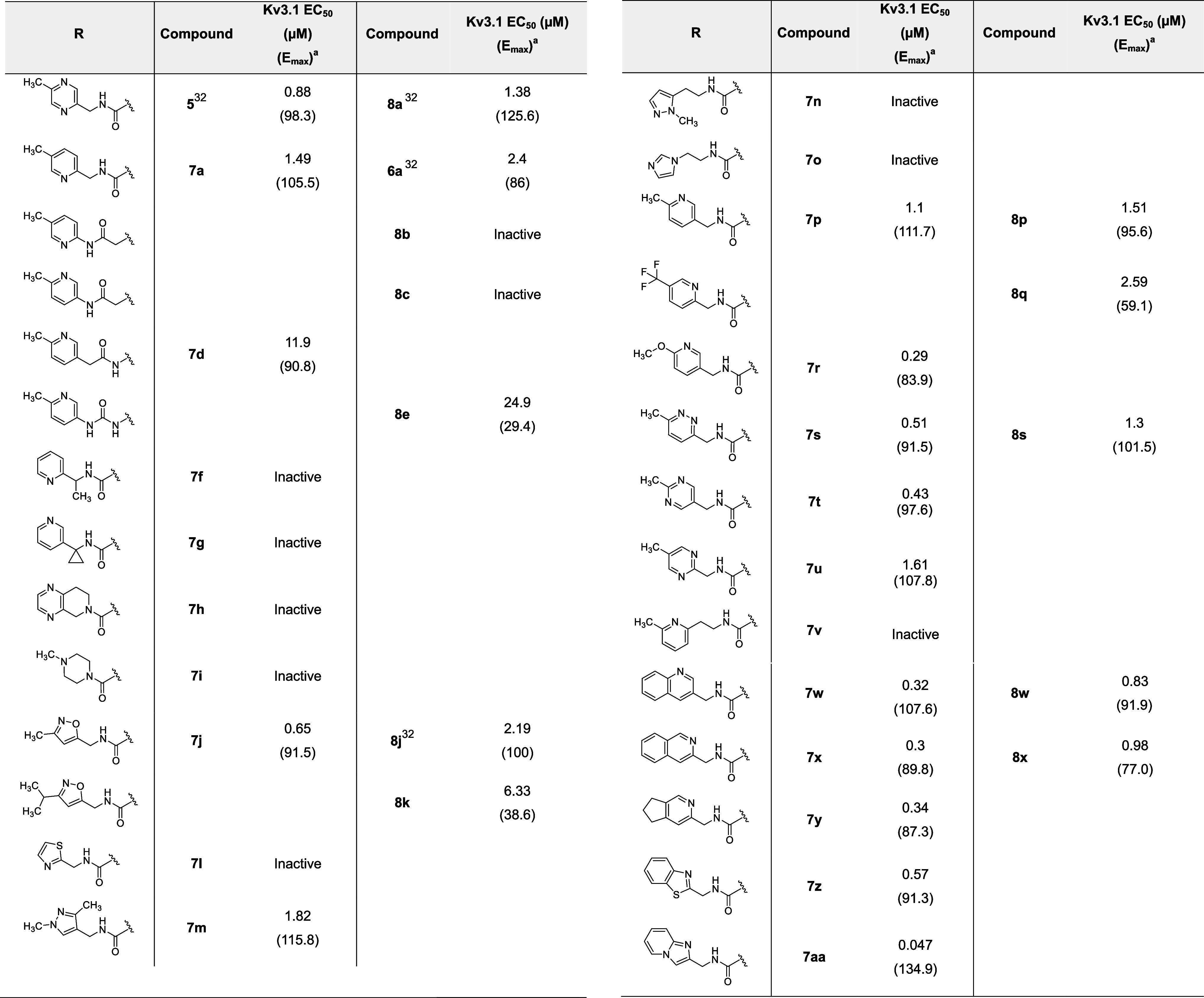
Structure–Activity Relationships at the C-3 Pyrazole Position

a Determined Tl^+^ flux assays. EC_50_ values represent a mean of three replicates. *E*_max_ values represent the maximal fluorescence response normalized to 15 μM **5** (set as 100% activity, with DMSO set as 0% activity). Inactive = EC_50_ could not be derived due to no activation or minimal activity observed at concentrations from 3 nM to 30 μM.

The configuration of C-3 amide was first investigated. Moving amide one methylene distal from pyrazole (**8b** and **8c**) was not successful. Inverting amide (**7d**) retained Kv3.1 activation but with only moderate potency, which was further eroded in urea **8e**. Substitution on carbon between the amide and the aromatic ring was not tolerated (**7f** and **7g**) nor were any tertiary amide (**7h** and **7i**) rings. On the basis of these results, the amide architecture of the parent molecules appeared to be necessary; we thus evaluated the suitability of additional terminal aromatic rings. Isoxazole-based rings similar to that on **4**/LuAG00563 proved to be successful, particularly in the context of 2-methyl, 4-methoxy sulfonamide (e.g., **7j**). While the methyl group of **7j** and **8j** ^32^ produced molecules of comparable potency to **5** and **8a**, respectively, further expansion of the alkyl group reduced Kv3.1 activation (**8k**). SAR preferences around the 5-membered heterocycles were found to be narrow, with compounds such as thiazole **7l** inactive and pyrazole **7m** showing reduced potency relative to **7j**. Furthermore, extension of the alkyl chain typically proved ineffectual (**7n** and **7o**).

Much better success was obtained with 6-membered heterocycles; 4-methyl 3-pyridine (**7p** and **8p**) proved comparable to the established methylated pyrazine (**5** and **7a**) and 2-pyridine (**7a** and **6a**) exemplars. The inclusion of an electron-withdrawing group (**8q**) had a minimal effect on activation, but 4-methoxy analogue **7r** was found to improve Kv3.1 activation potency by more than 5-fold compared to **7a**. Further, we identified good tolerance for changes to the position of the nitrogen atoms. Pyridazines **7s** and **8s** as well as pyrimidine **7t** showed superior potency to either **5** or **8a**, respectively, whereas pyrimidine regiomer **7u** was comparable to **8a**. As with the 5-membered heterocycles, extensions of the carbon linker (e.g., **7v**) led to inactive molecules. Together, these results suggest that the nitrogen atom at the 3 position of the aromatic ring (relative to the methyleneamide substitution) may be a critical determinant for high potency in the 6-membered heterocycles.

We also evaluated the heterobicyclic rings. Despite the SAR preference noted for nitrogen placement in the 6-membered rings, both quinolines **7w**/**8w** and isoquinolines **7x**/**8x** showed very good Kv3.1 activation potency. This tolerance extended to the saturated ring of **7y**. Pseudoisosteric benzothiazole **7z** showed only a minor drop in activation potency, and, surprisingly, imidazopyridine **7aa** showed activation potency nearly 10-fold better than any other analogue evaluated in the SAR study of the amide substitutions. Together, the Kv3.1 activation induced by these heterobicyclic analogues suggests that such moieties may be preferred over monocyclic rings in this template.

Fixing 4-methyl-2-pyridine of **6a** and **7a** in place, we evaluated the ability of changes in the sulfonamide end of the pyrazole template to affect changes in Kv3.1 activation (Table 3). Simple inversion of the 2- and 4-position substitutions of **7a** produced an inactive analogue (**9a**). Some activity could be recovered with 4-methyl using a 2-chloro substitution (**9b**), indicating a likely preference for lipophilic substitutions at this position. 2,4-Dichlorophenyl sulfonamide (**9c**) showed a reduced activity relative to **9b**. Electron-withdrawing groups (**9d** and **9e**) were poorly tolerated, as were heterocyclics (**9f**) and alkyl sulfonamides (**9g** and **9h**). The 4-position oxygen of sulfonamide in **7a** appeared superior to other changes; we thus experimented with the use of bicyclic sulfonamides that retained this atom. Design goals included the introduction of additional heteroatoms that might positively influence compound solubility and other physicochemical properties as well as balancing the aromatic ring-heavy nature of the scaffold with the addition of sp^3^-hybridized atoms. Analogues **9i** and **9j** were active, albeit with a reduction in potency compared to **7a**. With the reinforcement of the importance of 4-oxygen and lack of tolerance for 2-position oxygen, **9k** was inactive. However, quinoline **9l** displayed a surprisingly potent activation of the channel. Other heteroaromatic bicyclics, such as **9m**, were inactive. In contrast, dihydrobenzofuran analogue **9n** represents minimally substituted sulfonamide that roughly matches the potency of lead **7a**. While permethylation of this moiety (**9o**) showed reduced 3-fold potency relative to **9n**, the maximal activation shown by this analogue remained high.

**Table 3:**
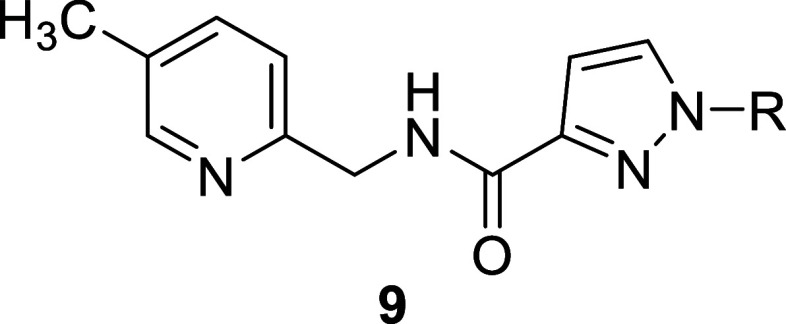

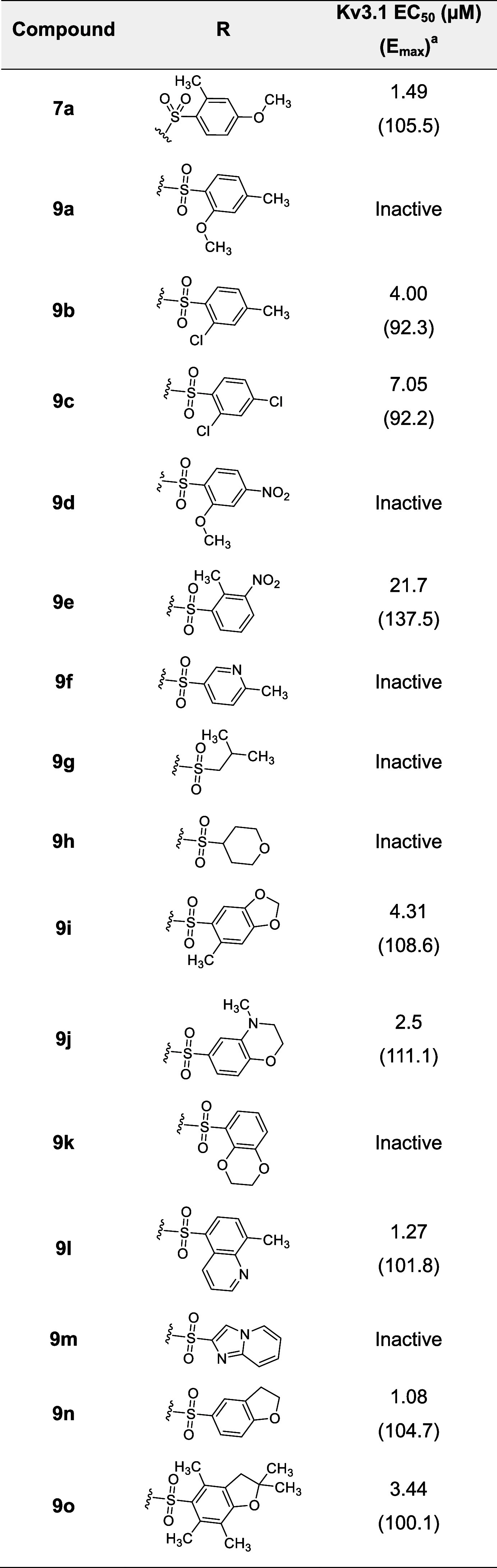
Structure–Activity Relationships at N-1 Sulfonamide

a Determined in Tl^+^ flux assays. EC_50_ values represent a mean of three replicates. *E*_max_ values represent the maximal fluorescence response normalized to 15 μM **5** (set as 100% activity, with DMSO set as 0% activity). Inactive = EC_50_ could not be derived due to no activation or minimal activity observed at concentrations from 3 nM to 30 μM.

Seeking synergies between substitutions on both ends of the central pyrazole, we evaluated a number of C-3 position amides using amines that had shown potent and high activation of Kv3.1 with dihydrobenzofuran sulfonamide of **9n** and the related methylated version of **9o** (Table 4). Interestingly, the use of other monocyclic heterocycles with methylated dihydrobenzofuran sulfonamide consistently produced analogues with higher potency on Kv3.1 than with unsubstituted dihydrobenzofuran (compare analogues **10a**–**10c** and **11a**–**11c**). Indeed, analogue **11c** displayed very potent channel activation and very high maximal activation, reflecting the increase in activity seen with pyridazine in **7s** vs **5**. While this SAR was consistent, the high potency of methoxypyridine or isoxazole-bearing amines (Table 2, **7r** and **7j**, respectively) did not translate to further improvements with dihydrobenzofuran. As heterobicyclic amines had shown good potencies in other contexts (Table 2), we also evaluated these with dihydrobenzofurans. However, quinolines **10f** and **10g** showed reduced potency compared to the prior analogues with 2-methyl, 4-methoxyphenyl sulfonamide, as did quinoline isosteres, such as **10h** and **10i**. Imidazopyridine **7aa** conferred the highest potency of the amines evaluated, and in the context of dihydrobenzofuran **10j**, this moiety also showed better potency than other compounds in this series, along with extremely high maximal activation. Heterobicyclic amines did not combine well with methylated dihydrobenzofuran. Analogue **11f**, for example, showed 2-fold reduced potency relative to **10f**, and **11j** showed poor potency of nearly 5 μM, despite the high potency observed with imidazopyridine in other contexts.

**Table 4:**
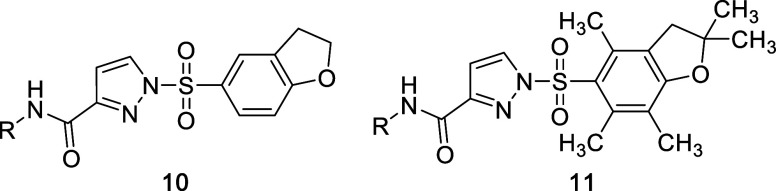

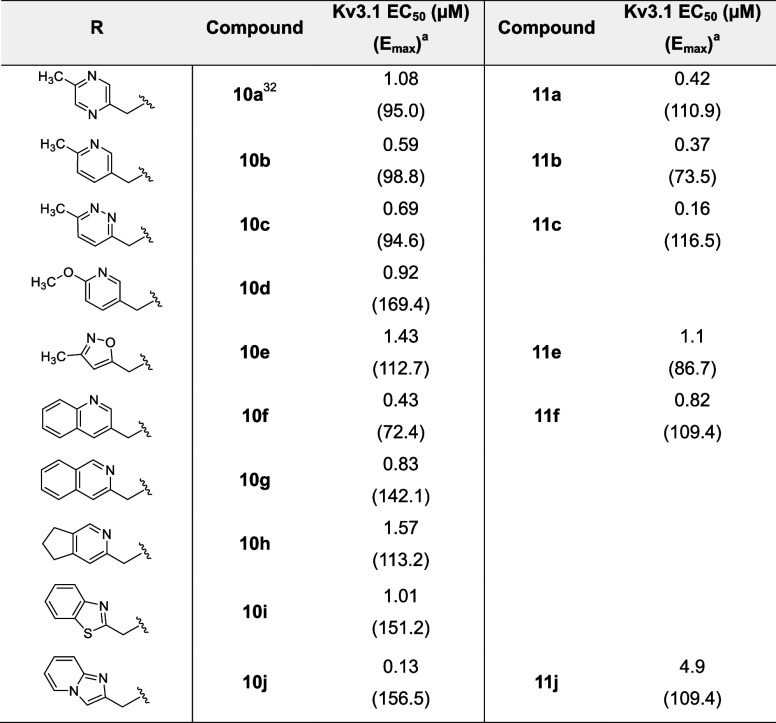
Evaluation of Dihydrobenzofuran Sulfonamides with C-3 Amides

a Determined in Tl^+^ flux assays. EC_50_ values represent a mean of three replicates. *E*_max_ values represent the maximal fluorescence response normalized to 15 μM **5** (set as 100% activity, with DMSO set as 0% activity).

## Patch-Clamp Characterization

Based on the potency and efficacy shown in Tl^+^ assays, several activators were selected for further profiling in an automated patch-clamp (APC) electrophysiology assay.^33^ Similar to Tl^+^ assays, the analogues activated Kv3.1 channels in a concentration-dependent manner. However, at concentrations of ≥3 μM, most analogues inhibited Kv3.1 currents (Figure 4). The SAR shown in the APC assay was not perfectly correlated with that observed in thallium assays, highlighting the need for validation using gold-standard voltage-clamp techniques (Table 5). Reported compound **5**, for example, showed a Tl^+^ EC_50_ of 880 nM but demonstrated higher potency of 120 nM in the APC setting. Several compounds (for example **7r**, **7w**, **7y**, and **10j**) did not match this APC potency, despite showing somewhat superior results in Tl^+^ assays. However, higher channel activation compared to **5** was often seen. For instance, compounds **7j**, **7s**, and **10a** showed nearly 2-fold higher activation over that attained by **5** in the APC assay. While **10c** and **10j** were the most potent compounds in the APC assay, analogue **7aa**, which has the best Tl^+^ assay potency in the set evaluated, shows perhaps the best overall APC profile, with potency essentially matching **5** and an activation level significantly exceeding that benchmark.

**Figure 4:**
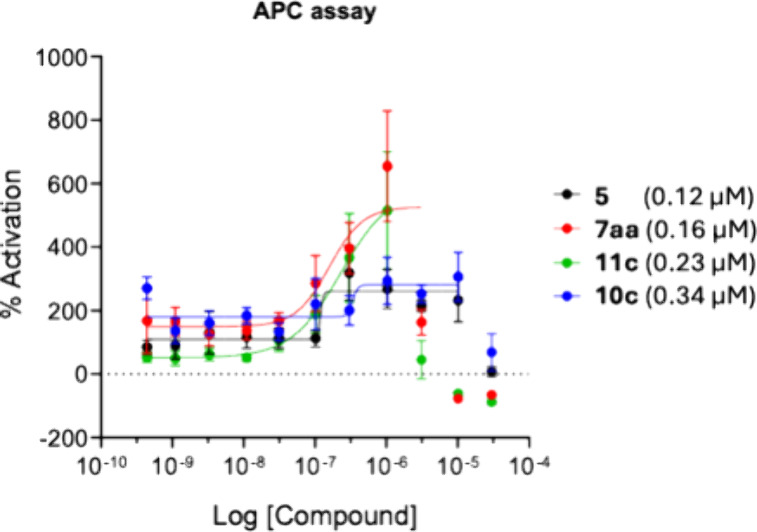
Overlay of representative compound dose–response curves in the APC assay.

**Table 5:** APC Electrophysiology Results Compared to the Corresponding Tl^+^ Assay Results

compound	APC assay^a^ EC_50_ (μM)	APC assay^a^ % *E*_max_	Tl^+^ assay^b^ EC_50_ (μM)	Tl^+^ assay^b^ % *E*_max_
**5**	0.12	292	0.88	98
**7j**	0.40	570	0.65	92
**7r**	0.36	302	0.29	84
**7s**	0.25	626	0.51	92
**7t**	0.31	303	0.43	98
**7w**	0.33	424	0.32	108
**7x**	0.38	353	0.30	90
**7y**	0.33	155	0.34	87
**7aa**	**0.16**	**527**	**0.047**	**135**
**10a**	1.70	624	1.08	95
**10b**	0.33	291	0.59	99
**10c**	**0.089**	**226**	0.69	95
**10f**	0.32	310	0.43	72
**10j**	**0.050**	**184**	0.13	157
**11a**	0.67	301	0.42	111
**11b**	0.13	105	0.37	74
**11c**	0.23	588	0.16	117

a Values are the mean of at least three replicates. *E*_max_ is the % activation of the baseline of the Kv3.1 current at −30 mV after compound addition and normalized to the full block of current with TEAC.

b Values are the mean of at least three replicates. *E*_max_ values represent the maximal fluorescence response with 15 μM **5** set as 100% activity and DMSO as 0% activity.

## Chemical Synthesis

Most reported analogues were prepared using variations on the general set of procedures shown in Scheme 1. No unexpected or unusually high safety hazards were encountered during the synthesis of the analogues. Pyrazole **12** reacted with the desired sulfonyl chloride using cesium carbonate or an organic amine base in a polar aprotic solvent. After saponification of ester **13** using standard conditions (aqueous lithium hydroxide), the final analogues **15** were prepared using amide coupling from acid **14** using either propanephosphonic acid anhydride (T3P) or carbodiimide as the activating agent. Variations on this scheme that were used to prepare additional compounds are described in the Supporting Information.

**Scheme 1:**
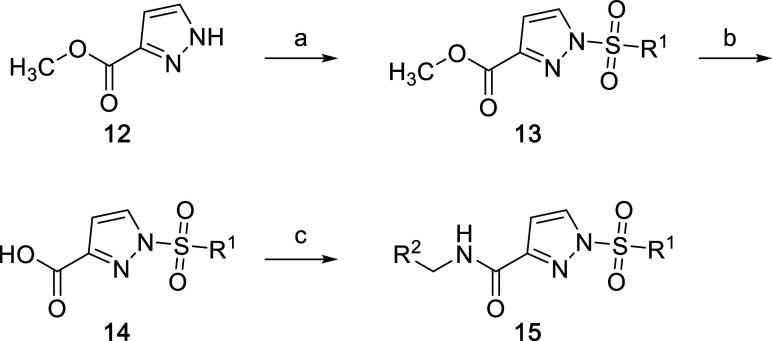
General Scheme for the Preparation of Analogues^a^

a Reagents and conditions: (a) appropriate sulfonyl chloride, Cs_2_CO_3_, DMF or DIPEA, ACN, 5 h; (b) 1 M LiOH, THF, 0 °C, 2 h; and (c) T3P, DIPEA, DMF, 12 h or EDC, HOBt, DIEPA, DMF.

## Conclusions

Our survey of SAR around a series of reported pyrazole-based activators of Kv3.1 succeeded in identifying analogues with superior potency and efficacy. Minimal tolerance for changes to the central portion of the molecule was observed, but we determined that several alternative heterocycles could be appended onto the C-3 amide side of the system, as long as the basic molecular architecture was retained. Notably, bicyclic moieties were very well-tolerated, leading to imidazopyridine **7aa**, the most potent Kv3.1 activator identified in this study.

Limited tolerance for changes around N-1 phenyl sulfonamide was also observed. However, selected examples, including dihydrobenzofuran **9n**, produced channel activation similar to that of previously reported analogue **5**, and a methylated dihydrobenzofuran derivative allowed for potent and effective activation in combination with several of the amines.

The potency relationships in the Tl^+^ assay did not translate perfectly to the more-precise automated patch-clamp setting. However, most tested analogues retained sub-micromolar potency and high efficacy within a range largely consistent with their performance in the Tl^+^ assay. Indeed, compound **7aa** displayed high potency and efficacy in both settings with both it and closely related analogue **10j** among the top performers.

The work further validates the presence of tractable SAR in this series of pyrazole-based Kv3.1 activators and, thus, indicates that this series has potential as a lead series for future discovery of CNS therapies. Further work to profile the *in vitro* characteristics of this series and other activator chemotypes will be reported in due course.

## Supplementary Material



## Notes

Experimental protocols for the molecular pharmacology assays (Tl^+^ flux and manual and automated patch-clamp functional assays), experimental protocols for compound synthesis, including compound characterization details, and ^1^H NMR and LC spectra for analogues (PDF)

## Abbreviations Used

Kv3.1: potassium voltage-gated channel subfamily C member 1
CNS: central nervous system
T-REx-HEK-293: tetracycline-regulated expression HEK-293 cells
*I*–*V*: current to voltage
Tl^+^: thallium
TEAC: tetraethylammonium chloride
APC: automated patch-clamp
DMF: dimethylformamide
DIPEA: *N*,*N*-diisoproylethylamine
ACN: acetonitrile
THF: tetrahydrofuran
EDC: 1-ethyl-3-(3-(dimethylamino)­propyl)­carbodiimide hydrochloride
HOBt: 1-hydroxybenzotriazole hydrate
