# Supplementary material for: Optimization of Pyrazole-Based Activators of Kv3.1
Source: ACS Med Chem Lett. 2026 Jun 25;17(7):1655–63. doi: 10.1021/acsmedchemlett.6c00272 (PMC13358987; doi:10.1021/acsmedchemlett.6c00272)

Supporting Information

Optimization of pyrazole-based activators of Kv3.1

Paul K. Spearing[1,‡], Vaishali Satpute Janve[2,‡], Ian M. Romaine[3], Navoda Jayakodiarachchi[1], Benjamin W. Guttentag[3], Somnath Jana[1,3], Kwangho Kim[1,3,4], Jerod S. Denton[2,3,4], Craig W. Lindsley[1,3,4,5], Alex G. Waterson[3,4,5,*]

Author affiliations:

[1] Warren Center for Neuroscience Drug Discovery, Vanderbilt University, Nashville, Tennessee 37232, United States

[2] Department of Anesthesiology, Vanderbilt University Medical Center, Nashville, Tennessee 37232, United States

[3] Vanderbilt Institute of Chemical Biology, Vanderbilt University, Nashville, Tennessee 37232, United States

[4] Department of Pharmacology, Vanderbilt University School of Medicine, Nashville, Tennessee 37232, United States

[5]Department of Chemistry, Vanderbilt University, Nashville, Tennessee 37232, United States

[‡] These authors contributed equally to this work.

[*] Corresponding author. Email: a.waterson@vanderbilt.edu

Contents:

Materials and methods for molecular pharmacology experiments *Pg S2*

Compound synthetic procedures and characterization data *Pg S6*

Spectral data for analogs *Pg S35*

Materials and methods for molecular pharmacology experiments

Generation of Kv3.1 monoclonal cell line

Monoclonal cell line expressing wild type (WT) Kv3.1b channels was generated by using the T-Rex-HEK293 cells (Thermo Fisher Scientific) and the pcDNA5/TO-hKv3.1b plasmid vector (synthesized by GenScript) encoding the human *KCNC1* isoform variant 2 (NM_004976.4). T-Rex-HEK293 cells were transfected with pcDNA5/TO-hKv3.1b plasmid vector using Lipofectamine LTX PLUS reagent (ThermoFisher Scientific, Catalog # A12621) following the manufacturer's instructions. In T-Rex-HEK293 cells Blasticidin resistance is conferred by the pcDNA6/TR plasmid while the transfected pcDNA5/TO-hKv3.1b plasmid confers Hygromycin resistance. Thus, transfected cells were selected for Kv3.1b expression by subjecting to selection with both antibiotics 48 hrs after transfection (Blasticidin HCl 5 μg/mL and Hygromycin B 250 μg/mL). Cells viable after antibiotic selection were amplified and then subjected to serial dilution to obtain ~1 cell per well in a 96 well plate. Wells with single isolated cells were identified and after amplification further expanded to establish stable monoclonal cell lines. Kv3.1 channel expression was induced using 1 μg/mL tetracycline 24 hours prior to all experiments. Monoclonal cell lines were then tested for Kv3.1 expression with Thallium assay and manual patch clamp recordings. One clone with robust Kv3.1 channel expression as seen with strong fluorescent signal in Tl assay and large (>5nA) current at +120mV with voltage clamp recording was then chosen for all subsequent experiments.

Quantitative Kv3.1 Thallium assay

Kv3.1 monoclonal cells were cultured as monolayers in Dulbecco's Modified Eagle Medium (DMEM; Gibco, 11965-092) supplemented with Tet-tested and heat-inactivated fetal bovine serum (10%, R&D Systems S10350H, 10%), Penicillin-Streptomycin (1%, Gibco, 15140-122, 2mM), Blasticidin HCl (5 μg/mL, Gibco, A11139–03, 10 mg/mL), and Hygromycin B (250 μg/mL, Invitrogen, Carlsbad, CA 10687–010, 50 mg/mL) at 37°C in a humidified atmosphere containing 95% air/5% CO_2. Kv3.1 expression was induced by treating cells

with 1 μg/mL tetracycline approximately 18 hours prior to the assay. On the day of the assay, cells were rinsed with assay buffer (Hanks' Balanced Salt Solution supplemented with 20 mM HEPES) and incubated for 1 hour at room temperature in the dark with Brilliant Thallos AM dye (0.2% v/v, Ion Bioscience, San Marcos, TX, Catalog #1381), a thallium-sensitive fluorescent indicator. Excess dye was removed by washing the cells with assay buffer. Cells were then plated into polyamine-coated, black-walled, clear-bottom 384-well plates (BD Biosciences, Bedford, MA) at a density of 20,000 cells per well in 20 μL of assay buffer. Fluorescence imaging was performed using a Panoptic Kinetic Imaging Plate Reader (Wavefront Bioscience, Franklin, TN). Baseline fluorescence measurements were acquired at 1 Hz (482 nm excitation/538 nm emission) prior to compound treatment. A ten-point concentration series from 1 nM to 30 μM was generated using the Echo liquid handler 650 (Labcyte, San Jose, CA) dissolved in assay buffer. Final DMSO concentration, 0.3% (v/v) in the assay was constant across all compound concentrations. Compounds were prepared SyncroPatch at 2x assay concentration and added to each well, 20 μL/well, followed by 10-minute incubation. Subsequently, 10 μL stimulus buffer containing 12.5 mM Tl_2SO_4 (Ion Bioscience, 7040S), 1 mM $MgSO_4$, 1.8 mM $CaSO_4{\cdot}H_2O$, 5 mM D-Glucose, 10 mM HEPES, and 125 mM $NaHCO_3$ was simultaneously added to each well of the plate with robotic liquid hander. The final Tl_2SO_4 concentration in the cell plate was 2.5 mM. Fluorescence measurements were acquired for 2 minutes to monitor thallium influx via Kv3.1 channels. The thallium induced fluorescence changes for each well were calculated by subtracting the baseline from the maximum steady-state response. The fluorescence responses were fitted to a four-parameter logistic equation using GraphPad Prism 10 software to determine potency and efficacy values.

Manual patch-clamps assay

Monoclonal cells expressing WT Kv3.1 channels were plated onto tissue culture-treated 35-mm polystyrene dishes at a density of 0.2-0.4 x 10^6 cells. Cells were cultured overnight with 1 μg/mL tetracycline to induce Kv3.1 channel expression in culture media in a 5% CO_2 incubator at 37°C. The next day cells were dissociated and plated on Matrigel–coated coverslips and allowed to recover for at least 30 minutes at 37°C with 5% CO_2 in an incubator

before the experiments. Patch electrodes (1.5 to 3 MΩ) were filled with an intracellular solution containing 130 mM KCl, 2 mM $MgCl_2$, 1 mM EGTA, 20 mM HEPES-free acid, and 2 mM Na_2ATP, pH 7.3, titrated with KOH, and osmolarity of 280±3 mOsmol/kg (adjusted with sucrose). The extracellular bath solution contained 135 mM NaCl, 5 mM KCl, 2 mM $CaCl_2$, 1 mM $MgCl_2$, 5 mM glucose, and 10 mM HEPES, and titrated with NaOH to get pH 7.4 and osmolarity of 300±3 mOsmol/kg. TEAC stock was made in extracellular solution. Macroscopic currents were recorded under voltage-clamp conditions using an Axopatch 200B Amplifier (Molecular Devices, Sunnyvale, CA). The voltage-clamp protocol consisted of a 1-s step to -120 mV from a -80 mV holding potential, followed by a 2-s ramp to +40 mV, repeated every 10 s. Data was collected at 10 kHz and filtered at 1 kHz. A step protocol was used to obtain IV and GV relationships, and dose response curves. Cells were voltage-clamped at a holding potential of −80 mV and then stepped every 1.5 seconds from −120 mV to 40 mV for 500 ms in 10-mV increments. Data acquisition and analysis were performed using the pClamp 10.7 software suite (Molecular Devices).

Automated patch-clamps assay

Kv3.1 currents were recorded in voltage clamp mode using a SyncroPatch 384 automated patch clamp instrument at room temperature in a perforated patch configuration using four-hole, medium-resistance NPC-384 chips (Nanion, 08 3007). Standard methods with KF-based recording solutions were used.[1] The intracellular recording solution contained (in mM) 100 KF, 10 KCl, 10 NaCl, 10 EGTA (pH 7.2). The reference NMDG60 solution contained (in mM) 140 NMDG-Cl, 80 NaCl, 4 KCl, 2 $CaCl_2$, 1 mL $MgCl_2$, 5 Glucose, 10 HEPES (pH7.4). The divalent-free solution was Ca2+ and Mg2+ free NMDG60 solution. The seal enhancer solution was prepared by adding 8 mM $CaCl_2$ to the NMDG60 solution. Cells were suspended in standard physiological solution containing (in mM) 140 NaCl, 4 KCl, 2 $CaCl_2$, 1 $MgCl_2$, 5 Glucose, 10 HEPES (pH 7.4). The seal enhancer solution contained (in mM) 140 NaCl, 4 KCl, 5 $CaCl_2$, 0.5 mL $MgCl_2$, 5 Glucose, 10 HEPES (pH7.3).

KF chip well were filled with divalent-free solution and the internal solution was introduced from the bottom of the chip, filling the apertures. Then 20 µL of cell suspension (~1-1.5 x10^6

cells/mL) was transferred to each well from the cell reservoir. Negative pressure (150 or 200 mBar) was applied to promote cell capture followed by addition of seal enhancer solution, which rapidly increases the seal resistance and tight seal formation. Membrane perforation was achieved by adding 5 μM escin (Sigma Millipore, E1378) to the internal buffer. Whole-cell access was assessed by monitoring membrane capacitance and series resistance. After seal formation, the cells were washed two times with reference NMDG60 solution. Then Kv3.1 currents were evoked by voltage clamping cells at a holding potential of -90 mV, then stepping the membrane potential (V_m) in 10mV increments from -100 mV to 60 mV for 500 msec, then stepping V_m back to -90 mV. This protocol was repeated every 20 sec. Currents in the control solution were recorded for 2 min, in Kv3.1 activator for 5 min, and in 12.5 mM TEAC for 2.5 min. Data analysis was performed using DataControl384 software (Nanion Technologies) where each well was inspected to meet the inclusion criterion of ≥-1 nA baseline holding current at -80 mV, stable drug response over time, and inhibition with TEAC. Each well received only one dose of activator. For each well % activation was calculated from the baseline and normalized to TEAC mediated inhibition. Top analogs had peak activation at -30mV, thus for concentration-response relationships, percent activation from baseline was calculated at -30mV for each well. The 50% activation concentration (EC_{50}) were derived from single-site, four-parameter logistical fits to dose-response data in GraphPad Prism 10 (GraphPad Software). Data are presented as means ± SEM and n represent the number of wells. Statistical comparisons of current amplitudes were performed using an unpaired t-test or ANOVA, with p-value of less than 0.05 was considered as statistically significant.

Reference

1. Li, K.; Janve, V. S.; Denton, J. S. "Automated patch clamp analysis of heterologously expressed Kir6.2/SUR1 and Kir6.1/SUR2B KATP currents" *Am. J. Physiol. Cell Physiol.* **2025**, *329*, C82-C92, DOI: https://doi.org/10.1152/ajpcell.00266.2025

Synthesis and characterization of compounds

General experimental information: No unexpected or unusually high safety hazards were encountered. Solvents were obtained from commercial sources. Commercial reagents were used as received. Compounds were purified by Teledyne ISCO normal phase column chromatograph system using RediSep Silver Silica Gel Disposable Flash Columns. Where necessary, preparative reverse phase HPLC was conducted on a Gilson HPLC system using a Phenomenex Luna column (100 Å, 50 x 21.20 mm, 5 μm C18) or a Phenomenex Kinetex EVO C18 column (100 x 30 mm, 5 μM) with UV/Vis detection. ^{1}H NMR spectra were recorded on Bruker 400 MHz spectrometer and are reported relative to deuterated solvent signals. Data for ^{1}H NMR spectra are reported as follows: chemical shift (δ ppm), multiplicity (s = singlet, d = doublet, t = triplet, q = quartet, quint = quintet, m = multiplet, br = broad, app = apparent), coupling constants (Hz), and integration. ^{13}C NMR spectra were recorded on Bruker 100 MHz spectrometer and are reported relative to deuterated solvent signals. LCMS was performed using Agilent 1260 series system with UV detection at 214 and 254 nm, and Accucore 2.6 *μ*m C18 80 Å LC column (50 mm x 2.1mm); a gradient of 2%−95% MeCN in H2O, and 0.1% TFA, over 1.5 min, then hold at 95% for 0.4 min with mass detection using an Agilent 6140 mass spectrometer with electrospray ionization (ESI) or on a Waters QDa / Acquity I-Class LCMS (Column: Phenomenex Kinetex EVO C18 (1.0x50 mm, 1.7um)) operating in ESI (+) ionization mode. Flow Rate: 0.4 mL/min, Acquire Time: 1.5 min, Wavelength: UV 215 & 254, Oven Temp.: 55°C.

Synthesis of 1-((2,3-dihydrobenzofuran-5-yl)sulfonyl)-N-((5-methylpyrazin-2-yl)methyl)-1H-pyrazole-3-carboxamide (6i)

Methyl 1-((4-methoxy-2-methylphenyl)sulfonyl)-1H-pyrazole-3-carboxylate: To a solution of methyl 1H-pyrazole-4-carboxylate (75.0 mg, 1 Eq, 595 μmol) in DMF (3 mL, 0.2 M) was added 4-methoxy-2-methylbenzenesulfonyl chloride (197 mg, 1.5 Eq, 892 μmol) and Cs_2CO_3 (581 mg, 3 Eq, 1.78 mmol). The reaction mixture was allowed to stir at rt for 3 h, then satd. aqueous. NH_4Cl (10 mL) was added, followed by EtOAc (15 mL). The organic phase was washed with water, dried over $MgSO_4$, and filtered. The filter cake was washed

with EtOAc and the combined organic layers were concentrated under reduced pressure. The crude product was purified by ISCO column chromatography eluting with 0 to 65% EtOAc in Hexane to afford Methyl 1-((4-methoxy-2-methylphenyl)sulfonyl)-1H-pyrazole-3-carboxylate (180 mg, 97%). ^{1}H NMR (400 MHz, $CDCl_3$) δ 8.63 (s, 1H), 8.15 (d, *J* = 9.0 Hz, 1H), 8.03 (s, 1H), 6.89 (dd, *J* = 2.4, 9.0 Hz, 1H), 6.80 (d, *J* = 2.4 Hz, 3H), 3.87 (s, 3H), 3.86 (s, 1H), 2.54 (s, 3H). LCMS *m/z* = 310.9 $[M+H]^+$.

1-((4-Methoxy-2-methylphenyl)sulfonyl)-1H-pyrazole-3-carboxylic acid: A solution of methyl 1-((4-methoxy-2-methylphenyl)sulfonyl)-1H-pyrazole-3-carboxylate (180.0 mg, 1 Eq, 580 µmol) in THF (4 mL, 0.1 M) was cooled to 0°C. LiOH (1.74 mL, 1M (aq), 3 Eq, 1.74 mmol)) was added and the reaction mixture was allowed to stir at 0°C for 1 h. The reaction mixture was quenched by the addition of 1N HCl, then was extracted with EtOAc (3 x 15 mL). The combined organic phase was washed with water, dried over $MgSO_4$, and filtered. The filter cake was washed with EtOAc and the combined organic layers were concentrated under reduced pressure. The crude product was taken on to the next step without further purification. LCMS *m/z* = 296.9 $[M+H]^+$.

*1-((2,3-Dihydrobenzofuran-5-yl)sulfonyl)-N-((5-methylpyrazin-2-yl)methyl)-1H-pyrazole-3-carboxamide (**6i**)***:** To a solution of methyl 1-((4-methoxy-2-methylphenyl)sulfonyl)-1H-pyrazole-4-carboxylic acid (20.7 mg, 1 Eq, 69.9 µmol) in DMF (1.5 mL, 0.05 M) was added (5-methylpyrazin-2-yl)methanamine (10.3 mg, 9.46 µL, 1.2 Eq, 83.8 µmol), followed by addition of propylphosphonic anhydride (61.7 µL, 50% Wt, 1.5 Eq, 105 µmol). The reaction mixture was allowed to stir at rt for 18 h, then water was added and the mixture was extracted EtOAc (3 x 15 mL). The combined organic phase was then dried over $MgSO_4$ and filtered. The crude product was purified by ISCO column chromatography eluting with 0 to 90% EtOAc in DCM to afford **6i** (5.3 mg, 19%). ^{1}H NMR (400 MHz, $CDCl_3$) δ 8.58 (s, 1H), 8.52 (s, 1H), 8.39 (s, 1H), 8.13 (d, *J* = 8.9 Hz, 1H), 7.98 (s, 1H), 6.99 (t, *J* = 4.5 Hz, 1H) 6.87 (dd, *J* = 2.6 Hz and 8.9 Hz, 1H), 6.78 (d, *J* = 2.4 Hz, 1H), 4.70 (d, *J* = 5.0 Hz, 2H), 3.85 (s, 3H), 2.57 (s, 3H), 2.53 (s, 3H). LCMS *m/z* = 402.0 $[M+H]^+$.

***N*-((5-Methylpyrazin-2-yl)methyl)-1-tosyl-1*H*-indazole-3-carboxamide (6g):** Synthesized in a similar manner as **6i** starting from 4-methylbenzenesulfonyl chloride, methyl 1H-indazole-3-carboxylate and (5-methylpyrazin-2-yl)methanamine to obtain 21 mg (32%) of **6g**. LCMS *m/z* = 422.4 $[M+H]^+$.

1-((4-Methoxy-2-methylphenyl)sulfonyl)-N-((5-methylpyrazin-2-yl)methyl)pyrrolidine-3-carboxamide (6h): Synthesized in a similar manner as **6i** starting from 4-methoxy-2-methylbenzenesulfonyl chloride, methyl pyrrolidine-3-carboxylate and (5-methylpyrazin-2-yl)methanamine to obtain 8 mg (41%) of **6h**. ^{1}H NMR (400 MHz, $CDCl_3$) δ 8.49 (d, *J* = 1.5 Hz, 1H), 8.41 (d, *J* = 1.4 Hz, 1H), 7.90 (d, *J* = 8.6 Hz, 1H), 6.84 – 6.64 (m, 3H), 4.57 (d, *J* = 5.2 Hz, 2H), 3.86 (s, 3H), 3.64 (dd, *J* = 10.0, 7.8 Hz, 1H), 3.45 – 3.29 (m, 3H), 3.04 (p, *J* = 7.8 Hz, 1H), 2.61 (s, 3H), 2.60 (s, 3H), 2.21 (td, *J* = 7.5, 5.2 Hz, 2H). LCMS *m/z* = 405.3 $[M+H]^+$.

2-((4-Methoxy-2-methylphenyl)sulfonyl)-6-((5-methylpyridin-2-yl)methyl)-2,4,5,6-tetrahydro-7H-pyrazolo[3,4-c]pyridin-7-one (6k): Synthesized by a set of procedures similar to those used in the preparation of **6j**, except reacting **24** with (5-methyl-2-pyridyl)methanamine. 2.2 mg (18%) of (**6k**) was obtained. ^{1}H NMR (400 MHz, $CDCl_3$) δ 8.26 (s, 1H), 8.09 (d, *J* = 8.9 Hz, 1H), 7.91 (s, 1H), 7.39 (d, *J* = 2.5, 8.1 Hz, 1H), 7.23 (d, *J* = 8.1 Hz, 1H), 6.78 (dd, *J* = 2.6, 9.0 Hz, 1H), 6.69 (d, *J* = 2.3 Hz, 1H), 4.75 (s, 2H), 3.78 (s, 3H), 3.57 (t, *J* = 6.6 Hz, 2H), 2.75 (t, *J* = 6.4 Hz, 1H), 2.49 (s, 3H), 2.24 (s, 3H). LCMS *m/z* = 427.0 $[M+H]^+$.

***1-((4-Methoxy-2-methylphenyl)sulfonyl)-N-((5-methylpyridin-2-yl)methyl)-1H-pyrazole-3-carboxamide (7a)*:** Synthesized in a similar manner as **6i** starting from 4-methoxy-2-methylbenzenesulfonyl chloride and (5-methylpyridin-2-yl)methanamine to obtain 1.2 mg (4%) of **7a**. LCMS *m/z* = 401.2 $[M+H]^+$.

***1-((4-Methoxy-2-methylphenyl)sulfonyl)-N-(1-(pyridin-2-yl)ethyl)-1H-pyrazole-3-carboxamide (7f)*:** Synthesized in a similar manner as **6i** starting from 4-methoxy-2-methylbenzenesulfonyl chloride and 1-(pyridin-2-yl)ethan-1-amine to obtain 3.1 mg (15%) of **7f**. ^{1}H NMR (400 MHz, $CDCl_3$) δ 8.64 (d, *J* = 5.0 Hz, 1H), 8.19 – 8.13 (m, 2H), 8.08 (d, *J* = 7.1

Hz, 1H), 7.96 – 7.90 (m, 1H), 7.58 – 7.51 (m, 1H), 7.46 – 7.41 (m, 1H), 6.92 (p, *J* = 2.2 Hz, 2H), 6.82 (d, *J* = 2.5 Hz, 1H), 5.38 (t, *J* = 7.1 Hz, 1H), 3.89 (s, 3H), 2.65 (s, 3H), 1.71 (d, *J* = 6.5 Hz, 3H). LCMS *m/z* = 401.2 $[M+H]^+$.

***1-((4-Methoxy-2-methylphenyl)sulfonyl)-N-(1-(pyridin-3-yl)cyclopropyl)-1H-pyrazole-3-carboxamide (7g)*:** Synthesized in a similar manner as **6i** starting from 4-methoxy-2-methylbenzenesulfonyl chloride and 1-(pyridin-2-yl)cyclopropan-1-amine to obtain 3.7 mg (25%) of **7g**. ^{1}H NMR (400 MHz, $CDCl_3$) δ 8.60 (d, *J* = 2.3 Hz, 1H), 8.51 (dd, *J* = 5.2, 1.4 Hz, 1H), 8.19 (d, *J* = 2.8 Hz, 1H), 8.14 (d, *J* = 8.9 Hz, 1H), 7.94 – 7.89 (m, 1H), 7.55 – 7.40 (m, 2H), 6.91 (dd, *J* = 8.8, 2.7 Hz, 2H), 6.83 (d, *J* = 2.7 Hz, 1H), 3.90 (s, 3H), 2.64 (s, 3H), 2.57 (s, 3H). LCMS *m/z* = 413.2 $[M+H]^+$.

***(7,8-Dihydropyrido[3,4-b]pyrazin-6(5H)-yl)(1-((4-methoxy-2-methylphenyl)sulfonyl)-1H-pyrazol-3-yl)methanone (7h)*:** Synthesized in a similar manner as **6i** starting from 4-methoxy-2-methylbenzenesulfonyl chloride and 5,6,7,8-tetrahydropyrido[3,4-b]pyrazine to obtain 4 mg (21%) of **7h**. LCMS *m/z* = 414.2 $[M+H]^+$.

***(1-((4-Methoxy-2-methylphenyl)sulfonyl)-1H-pyrazol-3-yl)(4-methylpiperazin-1-yl)methanone (7i)*:** Synthesized in a similar manner as **6i** starting from 4-methoxy-2-methylbenzenesulfonyl chloride and 1-methylpiperazine to obtain 2.0 mg (14%) of **7i**. ^{1}H NMR (400 MHz, $CDCl_3$) δ 8.19 (d, *J* = 2.7 Hz, 1H), 8.09 (d, *J* = 8.9 Hz, 1H), 6.90 (dd, *J* = 10.5, 2.6 Hz, 2H), 6.82 (d, *J* = 2.5 Hz, 1H), 5.03 (d, *J* = 14.8 Hz, 1H), 4.82 (d, *J* = 14.6 Hz, 1H), 3.97 – 3.87 (m, 5H), 3.58 – 3.50 (m, 2H), 2.90 – 2.84 (m ,2H), 2.64 (s, 3H), 2.55 (s, 3H). LCMS *m/z* = 379.1 $[M+H]^+$.

***1-((4-Methoxy-2-methylphenyl)sulfonyl)-N-((3-methylisoxazol-5-yl)methyl)-1H-pyrazole-3-carboxamide (7j)*:** Synthesized in a similar manner as **6i** starting from 4-methoxy-2-methylbenzenesulfonyl chloride and 1-methylpiperazine to obtain 4 mg (14%) of **7j**. LCMS *m/z* = 391.2 $[M+H]^+$.

1-((4-Methoxy-2-methylphenyl)sulfonyl)-N-(thiazol-2-ylmethyl)-1H-pyrazole-3-carboxamide (7l)**:** Synthesized in a similar manner as **6i** starting from 4-methoxy-2-methylbenzenesulfonyl chloride and thiazol-2-ylmethanamine to obtain 4.0 mg (20%) of **7l**. LCMS *m/z* = 393.1 $[M+H]^+$.

N-((1,3-Dimethyl-1H-pyrazol-4-yl)methyl)-1-((4-methoxy-2-methylphenyl)sulfonyl)-1H-pyrazole-3-carboxamide (7m)**:** Synthesized in a similar manner as **6i** starting from 4-methoxy-2-methylbenzenesulfonyl chloride and (1,3-dimethyl-1H-pyrazol-4-yl)methanamine to obtain 5 mg (22%) of **7m**. ^{1}H NMR (400 MHz, $CDCl_3$) δ 8.16 (d, *J* = 2.8 Hz, 1H), 8.10 (d, *J* = 8.9 Hz, 1H), 7.32 (s, 1H), 6.96 – 6.85 (m, 3H), 6.80 (d, *J* = 2.6 Hz, 1H), 4.38 (d, *J* = 5.7 Hz, 2H), 3.88 (s, 3H), 3.84 (s, 3H), 2.53 (s, 3H), 2.25 (s, 3H). LCMS *m/z* = 404.3 $[M+H]^+$.

1-((4-Methoxy-2-methylphenyl)sulfonyl)-N-(2-(1-methyl-1H-pyrazol-5-yl)ethyl)-1H-pyrazole-3-carboxamide (7n)**:** Synthesized in a similar manner as **6i** starting from 4-methoxy-2-methylbenzenesulfonyl chloride and 2-(1-methyl-1H-pyrazol-5-yl)ethan-1-amine to obtain 5 mg (18%) of **7n**. LCMS *m/z* = 404.2 $[M+H]^+$.

N-(2-(1H-Imidazol-1-yl)ethyl)-1-((4-methoxy-2-methylphenyl)sulfonyl)-1H-pyrazole-3-carboxamide (7o)**:** Synthesized in a similar manner as **6i** starting from 4-methoxy-2-methylbenzenesulfonyl chloride and 2-(1H-imidazol-1-yl)ethan-1-amine to obtain 2.5 mg (9%) of **7o**. ^{1}H NMR (400 MHz, $CDCl_3$) δ 8.44 (s, 1H), 8.15 (d, *J* = 2.8 Hz, 1H), 8.10 (d, *J* = 8.9 Hz, 1H), 7.51 (t, *J* = 6.2 Hz, 1H), 7.21 (d, *J* = 1.6 Hz, 1H), 7.09 (d, *J* = 1.5 Hz, 1H), 6.95 (d, *J* = 2.7 Hz, 1H), 6.91 (dd, *J* = 9.0, 2.6 Hz, 1H), 6.82 (d, *J* = 3.3 Hz, 1H), 4.45 – 4.33 (m, 2H), 3.87 (s, 3H), 3.86 – 3.81 (m, 2H), 2.55 (s, 3H). LCMS *m/z* = 390.2 $[M+H]^+$.

(4-Methoxy-2-methylphenyl)sulfonyl)-N-((6-methylpyridin-3-yl)methyl)-1H-pyrazole-3-carboxamide (7p)**:** Synthesized in a similar manner as **6i** starting from 4-methoxy-2-

methylbenzenesulfonyl chloride and (6-methylpyridin-3-yl)methanamine to obtain 7.0 mg (30%) of **7p**. ^{1}H NMR (400 MHz, $CDCl_3$) δ 8.20 (bs, 1H), 7.84 (d, *J* = 8.6 Hz, 1H), 7.39 (d, *J* = 8.1 Hz, 1H), 7.0 (d, *J* = 6.7 Hz, 1H), 6.74-6.67 (m, 2H), 4.79 (bs, 1H), 4.01 (d, *J* = 5.8 Hz, 2H), 3.78 (s, 3H), 5.52 (s, 3H), 2.44 (s, 2H). LCMS *m/z* = 401.0 $[M+H]^+$.

1-((4-Methoxy-2-methylphenyl)sulfonyl)-N-((6-methoxypyridin-3-yl)methyl)-1H-pyrazole-3-carboxamide (7r): Synthesized in a similar manner as **6i** starting from 4-methoxy-2-methylbenzenesulfonyl chloride and (6-methoxypyridin-3-yl)methanamine to obtain 39.8 mg (70%) of **7r**. ^{1}H NMR (400 MHz, $CDCl_3$) δ 8.17 (d, *J* = 2.7 Hz, 1H), 8.11 (s, 1H), 8.09 (d, *J* = 7.2 Hz, 1H), 7.58 (dd, *J* = 2.4, 8.5 Hz, 1H), 7.11 (t, *J* = 5.5 Hz, 1H), 6.94 (d, *J* = 2.7 Hz, 1H), 6.87 (dd, *J* = 2.6, 9.0 Hz, 1H), 6.79 (d, *J* = 2.3 Hz, 1H), 6.72 (d, *J* = 8.5 Hz, 1H), 4.50 (d, *J* = 6.0 Hz, 2H), 3.93 (s, 3H), 3.87 (s, 3H), 2.52 (s, 3H). ^{13}C NMR (100 MHz, $CDCl_3$) δ 164.58, 163.71, 160.37, 150.65, 146.14, 141.50, 138.85, 133.54, 132.66, 126.18, 125.75, 118.39, 111.68, 110.93, 108.24, 55.62, 53.39, 40.15, 20.61. LCMS *m/z* = 402.0 $[M+H]^+$.

1-((4-Methoxy-2-methylphenyl)sulfonyl)-N-((6-methylpyridazin-3-yl)methyl)-1H-pyrazole-3-carboxamide (7s): Synthesized in a similar manner as **6i** starting from 4-methoxy-2-methylbenzenesulfonyl chloride and (6-methylpyridazin-3-yl)methanamine to obtain 16mg (59%) of **7s**. ^{1}H NMR (400 MHz, $CDCl_3$) δ 8.10 (d, *J* = 2.7 Hz, 1H), 8.05 (d, *J* = 9.0 Hz, 1H), 7.59 (bs, 1H), 7.39 (d, *J* = 8.4 Hz, 1H), 7.23 (d, *J* = 8.4 Hz, 1H), 6.84 (d, *J* = 2.7 Hz, 1H), 6.81 (dd, *J* = 9.0, 2.5 Hz, 1H), 6.71 (d, *J* = 2.3 Hz, 1H), 4.76 (d, *J* = 6.0 Hz, 2H), 3.80 (s, 3H), 2.63 (s, 3H), 2.46 (s, 3H). LCMS *m/z* = 402.0 $[M+H]^+$.

1-((4-Methoxy-2-methylphenyl)sulfonyl)-N-((2-methylpyrimidin-5-yl)methyl)-1H-pyrazole-3-carboxamide (7t): Synthesized in a similar manner as **6i** starting from 4-methoxy-2-methylbenzenesulfonyl chloride and (2-methylpyrimidin-5-yl)methanamine to obtain 26.5 mg (38%) of **7t**. ^{1}H NMR (400 MHz, $CDCl_3$) δ 8.64 (s, 2H), 8.19 (d, *J* = 2.7 Hz, 1H), 8.11 (d, *J* = 8.9 Hz, 1H), 7.21 (t, *J* = 6.2 Hz, 1H), 6.95 (d, *J* = 2.7 Hz, 1H), 6.89 (dd, *J* = 2.5, 8.9 Hz, 1H), 6.80 (d, *J* = 2.5 Hz, 1H), 4.55 (d, *J* = 6.2 Hz, 2H), 3.89 (s, 3H), 2.74 (s, 3H), 2.53 (s,

3H). ^{13}C NMR (100 MHz, $CDCl_3$) δ 164.65, 156.63, 150.23, 141.52, 133.56, 132.78, 127.99, 118.42, 111.73, 108.25, 55.64, 38.28, 25.65, 20.63. LCMS *m/z* = 402.0 $[M+H]^+$.

1-((4-Methoxy-2-methylphenyl)sulfonyl)-N-((5-methylpyrimidin-2-yl)methyl)-1H-pyrazole-3-carboxamide (7u): Synthesized in a similar manner as **6i** starting from 4-methoxy-2-methylbenzenesulfonyl chloride and (5-methylpyrimidin-2-yl)methanamine to obtain 38.0 mg (55%) of **7u**. ^{1}H NMR (400 MHz, $CDCl_3$) δ 8.56 (s, 2H), 8.17 (d, *J* = 2.7 Hz, 1H), 8.13 (d, *J* = 8.9 Hz, 1H), 7.91 (t, *J* = 6.2 Hz, 1H), 6.95 (d, *J* = 2.7 Hz, 1H), 6.88 (dd, *J* = 2.5, 8.9 Hz, 1H), 6.81 (d, *J* = 2.5 Hz, 1H), 4.81 (d, *J* = 5.2 Hz, 2H), 3.88 (s, 3H), 2.64 (s, 3H), 2.34 (s, 3H). ^{13}C NMR (100 MHz, $CDCl_3$) δ 164.48, 162.94, 160.48, 157.26, 150.88, 141.82, 133.44, 132.35, 128.78, 126.10, 118.34, 111.66, 108.25, 55.61, 44.77, 20.79, 15.32. LCMS *m/z* = 402.0 $[M+H]^+$.

***1-((4-Methoxy-2-methylphenyl)sulfonyl)-N-(2-(6-methylpyridin-2-yl)ethyl)-1H-pyrazole-3-carboxamide* (7v):** Synthesized in a similar manner as **6i** starting from 4-methoxy-2-methylbenzenesulfonyl chloride and 2-(6-methylpyridin-2-yl)ethan-1-amine to obtain 1.6 mg (6%) of **7v**. LCMS *m/z* = 415.2 $[M+H]^+$.

1-((4-Methoxy-2-methylphenyl)sulfonyl)-N-(quinolin-3-ylmethyl)-1H-pyrazole-3-carboxamide (7w): Synthesized in a similar manner as **6i** starting from 4-methoxy-2-methylbenzenesulfonyl chloride and quinolin-3-ylmethanamine to obtain 23.7 mg (84%) of **7w**. ^{1}H NMR (400 MHz, $CDCl_3$) δ 8.88 (d, *J* =2.1 Hz. 1H), 8.17 (d, *J* = 2.8 Hz, 1H), 8.12-8.08 (m, 2H), 8.07 (d, *J* = 8.9 Hz, 1H), 7.78 (d, *J* = 8.1 Hz, 1H), 7.70 (t, *J* = 7.7 Hz, 1H), 7.55 (t, *J* = 7.7 Hz, 1H, 7.33 (t, *J* = 5.7 Hz, 1H), 6.95 (d, *J* = 2.7 Hz, 1H), 6.83 (dd, *J* = 2.7 and 8.9 Hz, 1H), 6.75 (d, *J* = 2.4 Hz, 1H), 4.76 (d, *J* = 6.3 Hz, 2H), 3.83 (s, 3H), 2.50 (s, 3H). LCMS *m/z* = 437.0 $[M+H]^+$.

***N-(Isoquinolin-3-ylmethyl)-1-((4-methoxy-2-methylphenyl)sulfonyl)-1H-pyrazole-3-carboxamide (7x)*:** Synthesized in a similar manner as **6i** starting from 4-methoxy-2-

methylbenzenesulfonyl chloride and isoquinolin-3-ylmethanamine to obtain 32.7 mg (55%) of **7x**. ^{1}H NMR (400 MHz, $CDCl_3$) δ 9.13 (s, 1H), 8.08 (d, *J* = 2.7 Hz, 1H), 8.03 (d, *J* = 8.9 Hz, 1H), 7.89 (d, *J* = 8.1 Hz, 1H), 7.70 (d, *J* = 8.3 Hz, 1H), 7.63-7.59 (m, 3H), 7.53-7.49 (m, 3H), 6.86 (d, *J* = 2.7 Hz, 1H), 6.78 (dd, *J* = 2.5, 8.9 Hz, 1H), 6.70 (d, *J* = 2.3 Hz, 1H), 4.76 (d, *J* = 5.9 Hz, 2H), 3.78 (s, 3H), 2.48 (s, 3H). ^{13}C NMR (100 MHz, $CDCl_3$) δ 164.53, 160.54, 152.33, 150.86, 150.03, 141.65, 136.27, 133.59, 132.45, 130.56, 127.75, 127.45, 127.12, 126.47, 125.85, 118.39, 118.16, 111.65, 108.20, 55.61, 44.60, 20.70. LCMS *m/z* = 437.0 $[M+H]^+$.

***N-((6,7-Dihydro-5H-cyclopenta[b]pyridin-3-yl)methyl)-1-((4-methoxy-2-methylphenyl)sulfonyl)-1H-pyrazole-3-carboxamide (7y)*:** Synthesized in a similar manner as **6i** starting from 4-methoxy-2-methylbenzenesulfonyl chloride and (6,7-dihydro-5H-cyclopenta[b]pyridin-3-yl)methanamine to obtain 38.0 mg (66%) of **7y**. ^{1}H NMR (400 MHz, $CDCl_3$) δ 8.27 (s, 1H), 8.17 (d, *J* = 2.7 Hz, 1H), 8.09 (d, *J* = 9.0 Hz, 1H), 7.50 (s, 1H), 7.17 (t, *J* = 5.7 Hz, 1H), 6.94 (d, *J* = 2.7 Hz, 1H), 6.87 (dd, *J* = 2.5, 8.9 Hz, 1H), 6.78 (d, *J* = 2.3 Hz, 1H), 4.54 (d, *J* = 6.2 Hz, 2H), 3.87 (s, 3H), 2.99 (t, *J* = 7.7 Hz, 2H), 2.91 (t, *J* = 7.4 Hz, 2H), 2.52 (s, 3H), 2.13 (p, *J* = 7.6 Hz, 2H). ^{13}C NMR (100 MHz, $CDCl_3$) δ 165.19, 164.59, 160.41, 150.62, 146.88, 141.50, 137.23, 133.55, 132.66, 132.07, 130.78, 125.72, 118.39, 111.68, 108.25, 55.62, 40.72, 33.76, 30.45, 23.19, 20.62. LCMS *m/z* = 427.0 $[M+H]^+$.

N-(Benzo[d]thiazol-2-ylmethyl)-1-((4-methoxy-2-methylphenyl)sulfonyl)-1H-pyrazole-3-carboxamide (7z): Synthesized in a similar manner as **6i** starting from 4-methoxy-2-methylbenzenesulfonyl chloride and benzo[d]thiazol-2-ylmethanamine to obtain 45.4 mg (76%) of **7z**. ^{1}H NMR (400 MHz, $CDCl_3$) δ 8.10 (d, *J* = 2.7 Hz, 1H), 8.03 (d, *J* = 9.0 Hz, 1H), 7.90 (d, *J* = 8.2 Hz, 1H), 7.76 (d, *J* = 7.9 Hz, 1H), 7.56 (t, *J* = 5.9 Hz, 1H), 7.43-7.37 (m 1H), 7.33-7.28 (m, 1H), 6.88 (d, *J* = 2.7 Hz, 1H), 6.79 (dd, *J* = 2.5, 8.9 Hz, 1H), 6.70 (d, *J* = 2.4 Hz, 1H), 4.91 (d, *J* = 6.1 Hz, 2H), 3.78 (s, 3H), 2.48 (s, 3H). ^{13}C NMR (100 MHz, $CDCl_3$) δ 167.74, 164.62, 160.56, 152.74, 150.10, 141.67, 135.25, 133.66, 132.59, 126.11, 125.66, 125.22, 122.88, 121.66, 118.44, 111.68, 108.27, 55.62, 41.39, 20.72. LCMS *m/z* = 442.9 $[M+H]^+$.

***N*-(Imidazo[1,2-a]pyridin-2-ylmethyl)-1-((4-methoxy-2-methylphenyl)sulfonyl)-1*H*-pyrazole-3-carboxamide (7aa):** Synthesized in a similar manner as **6i** starting from 4-methoxy-2-methylbenzenesulfonyl chloride and imidazo[1,2-a]pyridin-2-ylmethanamine to obtain 25.1 mg (44%) of **7aa**. ^{1}H NMR (400 MHz, $CDCl_3$) δ 8.16 (d, *J* = 2.7 Hz, 1H), 8.11 (d, *J* = 9.0 Hz, 1H), 8.06 (d, *J* = 6.8 Hz, 1H), 7.57 (s, 1H), 7.54 (d, *J* = 9.0 Hz, 1H), 7.45 (t, *J* = 5.2 Hz, 1H), 7.20-7.15 (m 1H), 6.92 (d, *J* = 2.7 Hz, 1H), 6.88 (dd, *J* = 2.5, 8.8 Hz, 1H), 6.84-6.76 (m, 2H), 4.72 (d, *J* = 5.9 Hz, 2H), 3.88 (s, 3H), 2.53 (s, 3H). ^{13}C NMR (100 MHz, $CDCl_3$) δ 164.55, 160.42, 150.79, 145.04, 143.08, 141.55, 133.69, 132.41, 125.71, 125.64, 124.66, 118.40, 117.22, 112.34, 111.65, 110.22, 108.06, 55.61, 37.55, 20.65. LCMS *m/z* = 425.9 $[M+H]^+$.

***N*-((5-Methylpyrazin-2-yl)methyl)-1-tosyl-1*H*-pyrazole-3-carboxamide (8a):** Synthesized in a similar manner as **6i** starting from 4-methylbenzenesulfonyl chloride to obtain 7.0 mg (39%) of **8a**. ^{1}H NMR (400 MHz, $CDCl_3$) δ 8.43 (s, 1H), 8.34 (s, 1H), 8.04 (d, *J* = 2.6 Hz, 1H), 7.82 (d, *J* = 8.4 Hz, 2H), 7.55 (bs, 1H), 7.29 (d, *J* = 8.2 Hz, 2H), 6.84 (d, *J* = 2.8 Hz, 1H), 4.63 (d, *J* = 5.9 Hz, 2H), 2.50 (s, 3H), 2.37 (s, 3H). LCMS *m/z* = 372.0 $[M+H]^+$.

***N*-(5-Methylpyridin-2-yl)-2-(1-tosyl-1*H*-pyrazol-3-yl)acetamide (8b):** Synthesized in a similar manner as **6i** starting from ethyl 2-(1H-pyrazol-3-yl)acetate **30** and utilizing 2-amino-5-methylpyridine to obtain 7.1 mg (37%) of **6e**. ^{1}H NMR (400 MHz, $CDCl_3$) δ 9.24 (s, 1H), 8.16 – 8.07 (m, 3H), 7.95 (d, *J* = 8.5 Hz, 2H), 7.60 (dd, *J* = 8.6, 2.3 Hz, 1H), 7.33 (d, *J* = 8.5 Hz, 2H), 6.47 (d, *J* = 2.7 Hz, 1H), 3.83 (s, 2H), 2.42 (s, 3H), 2.34 (s, 3H).LCMS *m/z* = 371.3 $[M+H]^+$.

***N*-(6-Methylpyridin-3-yl)-2-(1-tosyl-1*H*-pyrazol-3-yl)acetamide (8c):** Synthesized in a similar manner as **6i** starting from ethyl 2-(1H-pyrazol-3-yl)acetate **30** and utilizing 5-amino-2-methylpyridine to obtain 6.4 mg (33%) of **6e**. LCMS *m/z* = 371.3 $[M+H]^+$.

***N*-((3-Methylisoxazol-5-yl)methyl)-1-tosyl-1*H*-pyrazole-3-carboxamide (8j):** Synthesized in a similar manner as **6i** starting from 4-methylbenzenesulfonyl chloride and (3-methylisoxazol-5-yl)methanamine to obtain 5 mg (22%) of **8j**. LCMS *m/z* = 361.2 $[M+H]^+$.

***N*-((3-Isopropylisoxazol-5-yl)methyl)-1-tosyl-1*H*-pyrazole-3-carboxamide (8k):** Synthesized in a similar manner as **6i** starting from 4-methylbenzenesulfonyl chloride and (3-isopropylisoxazol-5-yl)methanamine to obtain 6 mg (23%) of **8k**. ^{1}H NMR (400 MHz, $CDCl_3$) δ 8.15 (d, *J* = 2.7 Hz, 1H), 7.92 (d, *J* = 8.4 Hz, 2H), 7.39 (d, *J* = 7.9 Hz, 2H), 6.93 (d, *J* = 2.8 Hz, 1H), 6.11 (s, 1H), 4.67 (dd, *J* = 6.2 Hz, 2H), 3.05 (dt, *J* = 13.9, 7.0 Hz, 1H), 2.47 (s, 3H), 1.29 (d, *J* = 7.0 Hz, 6H). LCMS *m/z* = 389.3 $[M+H]^+$.

***N*-((6-Methylpyridin-3-yl)methyl)-1-tosyl-1*H*-pyrazole-3-carboxamide (8p):** Synthesized in a similar manner as **6i** starting from 4-methylbenzenesulfonyl chloride and (6-methylpyridin-3-yl)methanamine to obtain 3.0 mg (11%) of **8p**. LCMS *m/z* = 371.2 $[M+H]^+$.

1-Tosyl-N-((5-(trifluoromethyl)pyridin-2-yl)methyl)-1H-pyrazole-3-carboxamide (8q): Synthesized in a similar manner as **6i** starting from 4-methylbenzenesulfonyl chloride and (5-(trifluoromethyl)pyridin-2-yl)methanamine to obtain 3.2 mg (12%) of **8q**. ^{1}H NMR (400 MHz, $CDCl_3$) δ 8.93 – 8.86 (m, 1H), 8.14 (d, *J* = 2.8 Hz, 1H), 8.05 (dd, *J* = 8.3, 2.3 Hz, 1H), 7.96 (d, *J* = 8.4 Hz, 2H), 7.64 (d, *J* = 8.1 Hz, 1H), 7.39 (d, *J* = 8.2 Hz, 2H), 6.92 (d, *J* = 2.7 Hz, 1H), 4.86 (d, *J* = 5.8 Hz, 2H), 2.46 (s, 3H). LCMS *m/z* = 425.2 $[M+H]^+$.

***N*-((6-Methylpyridazin-3-yl)methyl)-1-tosyl-1*H*-pyrazole-3-carboxamide (8s):** Synthesized in a similar manner as **6i** starting from 4-methylbenzenesulfonyl chloride and (6-methylpyridazin-3-yl)methanamine to obtain 8.4 mg (34%) of **8s**. ^{1}H NMR (400 MHz, $CDCl_3$) δ 8.14 (d, *J* = 2.8 Hz, 1H), 7.93 (d, *J* = 8.4 Hz, 2H), 7.82 (t, *J* = 6.1 Hz, 1H), 7.56 (d, *J* = 8.6 Hz, 1H), 7.41–7.38 (m, 3H), 6.92 (d, *J* = 2.7 Hz, 1H), 4.89 (d, *J* = 6.0 Hz, 2H), 2.77 (s, 3H), 2.46 (s, 3H). LCMS *m/z* = 372.3 $[M+H]^+$.

N-(Quinolin-3-ylmethyl)-1-tosyl-1H-pyrazole-3-carboxamide (8w): Synthesized in a similar manner as **6i** starting from 4-methylbenzenesulfonyl chloride and quinolin-3-ylmethanamine to obtain 2.7 mg (10%) of **8w**. ^{1}H NMR (400 MHz, $CDCl_3$) δ 9.07 (s, 1H), 8.48 (s, 1H), 8.42 (d, *J* = 8.6 Hz, 1H), 8.15 (d, *J* = 2.7 Hz, 1H), 7.96 – 7.79 (m, 4H), 7.78 – 7.64 (m, 2H), 7.37 (d, *J* = 8.0 Hz, 2H), 6.95 (d, *J* = 2.7 Hz, 1H), 4.86 (d, *J* = 5.9 Hz, 2H), 2.64 (s, 3H). LCMS *m/z* = 407.3 $[M+H]^+$.

N-(Isoquinolin-3-ylmethyl)-1-tosyl-1H-pyrazole-3-carboxamide (8x): Synthesized in a similar manner as **6i** starting from 4-methylbenzenesulfonyl chloride and isoquinolin-3-ylmethanamine to obtain 3.5 mg (15%) of **8x**. LCMS *m/z* = 407.3 $[M+H]^+$.

1-((4-Methoxy-2-methylphenyl)sulfonyl)-N-((5-methylpyridin-2-yl)methyl)-1H-pyrazole-3-carboxamide (9a): Synthesized in a similar manner as **6i** starting from 4-methoxy-2-methylbenzenesulfonyl chloride and (5-methylpyridin-2-yl)methanamine to obtain 15 mg (54%) of **9a**. H NMR (400 MHz, $CDCl_3$) δ 8.39 – 8.35 (m, 1H), 8.25 (d, *J* = 2.7 Hz, 1H), 8.01 (d, *J* = 8.1 Hz, 1H), 7.68 (t, *J* = 5.9 Hz, 1H), 7.53 (dd, *J* = 7.9, 2.1 Hz, 1H), 7.29 (d, *J* = 8.1 Hz, 1H), 6.96 (dt, *J* = 8.3, 1.1 Hz, 1H), 6.91 (d, *J* = 2.7 Hz, 1H), 6.79 – 6.70 (s, 1H), 4.69 (d, *J* = 5.8 Hz, 2H), 3.79 (s, 3H), 2.44 (s, 3H), 2.35 (s, 3H). LCMS *m/z* = 401.2 $[M+H]^+$.

1-((2-Chloro-4-methylphenyl)sulfonyl)-N-((5-methylpyridin-2-yl)methyl)-1H-pyrazole-3-carboxamide (9b): Synthesized in a similar manner as **6i** starting from 2-chloro-4-methylbenzenesulfonyl chloride and (5-methylpyridin-2-yl)methanamine to obtain 24 mg (86%) of **9b**. LCMS *m/z* = 405.2 $[M+H]^+$.

1-((2,4-Dichlorophenyl)sulfonyl)-N-((5-methylpyridin-2-yl)methyl)-1H-pyrazole-3-carboxamide (9c): Synthesized in a similar manner as **6i** starting from 2,4-dichlorobenzenesulfonyl chloride and (5-methylpyridin-2-yl)methanamine to obtain 6 mg (21%) of **9c**. ^{1}H NMR (400 MHz, $CDCl_3$) δ 8.40 (s, 1H), 8.35 – 8.25 (m, 2H), 7.79 (t, *J* = 6.0 Hz,

1H), 7.66 – 7.48 (m, 3H), 7.36 (d, *J* = 8.0 Hz, 1H), 6.97 (d, *J* = 2.8 Hz, 1H), 4.72 (d, *J* = 5.8 Hz, 2H), 2.64 (s, 3H), 2.38 (s, 3H). LCMS *m/z* = 425.1 $[M+H]^+$.

1-((2-Methoxy-4-nitrophenyl)sulfonyl)-N-((5-methylpyridin-2-yl)methyl)-1H-pyrazole-3-carboxamide (9d): Synthesized in a similar manner as **6i** starting from 2-methoxy-4-nitrobenzenesulfonyl chloride and (5-methylpyridin-2-yl)methanamine to obtain 14 mg (47%) of **9d**. LCMS *m/z* = 432.2 $[M+H]^+$.

1-((2-Methyl-3-nitrophenyl)sulfonyl)-N-((5-methylpyridin-2-yl)methyl)-1H-pyrazole-3-carboxamide (9e): Synthesized in a similar manner as **6i** starting from 2-methyl-3-nitrobenzenesulfonyl chloride and (5-methylpyridin-2-yl)methanamine to obtain 2.2 mg (8%) of **9e**. ^{1}H NMR (400 MHz, $CDCl_3$) δ 8.51 – 8.41 (m, 2H), 8.28 – 8.17 (m, 2H), 8.01 (dd, *J* = 8.1, 1.4 Hz, 1H), 7.86 (d, *J* = 8.1 Hz, 1H), 7.71 – 7.60 (m, 2H), 6.99 (d, *J* = 2.7 Hz, 1H), 4.85 (d, *J* = 5.9 Hz, 2H), 2.71 (s, 3H), 2.47 (s, 3H). LCMS *m/z* = 416.2 $[M+H]^+$.

N-((5-Methylpyridin-2-yl)methyl)-1-((6-methylpyridin-3-yl)sulfonyl)-1H-pyrazole-3-carboxamide (9f): Synthesized in a similar manner as **6i** starting from 6-methylpyridine-3-sulfonyl chloride and (5-methylpyridin-2-yl)methanamine to obtain 2.4 mg (9%) of **9f**. LCMS *m/z* = 372.3 $[M+H]^+$.

1-(Isobutylsulfonyl)-N-((5-methylpyridin-2-yl)methyl)-1H-pyrazole-3-carboxamide (9g): Synthesized in a similar manner as **6i** starting from 2-methylpropane-1-sulfonyl chloride and (5-methylpyridin-2-yl)methanamine to obtain 2.2 mg (9%) of **9g**. LCMS *m/z* = 337.2 $[M+H]^+$.

N-((5-Methylpyridin-2-yl)methyl)-1-((tetrahydro-2H-pyran-4-yl)sulfonyl)-1H-pyrazole-3-carboxamide (9h): Synthesized in a similar manner as **6i** starting from tetrahydro-2H-pyran-4-sulfonyl chloride and (5-methylpyridin-2-yl)methanamine to obtain 3.7 mg (15%) of **9h**. ^{1}H NMR (400 MHz, $CDCl_3$) δ 8.47 (d, *J* = 2.1 Hz, 1H), 8.38 (s, 1H), 8.06 (d, *J* = 2.7 Hz, 1H),

7.87 (d, *J* = 8.0 Hz, 1H), 7.62 (d, *J* = 8.0 Hz, 1H), 6.97 (d, *J* = 2.7 Hz, 1H), 4.89 (d, *J* = 5.9 Hz, 2H), 4.17 – 3.98 (m, 3H), 3.48 (td, *J* = 11.8, 2.2 Hz, 2H), 2.47 (s, 3H), 2.07 – 1.89 (m, 2H), 1.88 – 1.83 (m, 2H). LCMS *m/z* = 365.3 $[M+H]^+$.

1-((6-Methylbenzo[d][1,3]dioxol-5-yl)sulfonyl)-N-((5-methylpyridin-2-yl)methyl)-1H-pyrazole-3-carboxamide (9i): Synthesized in a similar manner as **6i** starting from 6-methylbenzo[d][1,3]dioxole-5-sulfonyl chloride and (5-methylpyridin-2-yl)methanamine to obtain 2.0 mg (7%) of **9i**. LCMS *m/z* = 415.2 $[M+H]^+$.

1-((4-Methyl-3,4-dihydro-2H-benzo[b][1,4]oxazin-6-yl)sulfonyl)-N-((5-methylpyridin-2-yl)methyl)-1H-pyrazole-3-carboxamide (9j): Synthesized in a similar manner as **6i** starting from 4-methyl-3,4-dihydro-2H-benzo[b][1,4]oxazine-6-sulfonyl chloride and (5-methylpyridin-2-yl)methanamine to obtain 2.2 mg (9%) of **9j**. ^{1}H NMR (400 MHz, $CDCl_3$) δ 8.47 (s, 1H), 8.14 – 8.03 (m, 2H), 7.89 (d, *J* = 8.1 Hz, 1H), 7.68 (d, *J* = 8.0 Hz, 1H), 7.34 (dd, *J* = 8.5, 2.3 Hz, 1H), 7.25 (d, *J* = 2.3 Hz, 1H), 6.89 – 6.85 (m, 2H), 4.94 (d, *J* = 6.0 Hz, 2H), 4.38 – 4.32 (m, 2H), 3.39 – 3.30 (m, 2H), 3.01 (s, 3H), 2.48 (s, 3H). LCMS *m/z* = 428.2 $[M+H]^+$.

1-((2,3-Dihydrobenzo[b][1,4]dioxin-5-yl)sulfonyl)-N-((5-methylpyridin-2-yl)methyl)-1H-pyrazole-3-carboxamide (9k): Synthesized in a similar manner as **6i** starting from 2,3-dihydrobenzo[b][1,4]dioxine-5-sulfonyl chloride and (5-methylpyridin-2-yl)methanamine to obtain 9 mg (32%) of **9k**. LCMS *m/z* = 415.2 $[M+H]^+$.

N-((5-Methylpyridin-2-yl)methyl)-1-((8-methylquinolin-5-yl)sulfonyl)-1H-pyrazole-3-carboxamide (9l): Synthesized in a similar manner as **6i** starting from 8-methylquinoline-5-sulfonyl chloride and (5-methylpyridin-2-yl)methanamine to obtain 18 mg (62%) of **9l**. ^{1}H NMR (400 MHz, $CDCl_3$) δ 9.32 (dd, *J* = 8.8, 1.7 Hz, 1H), 9.04 (dd, *J* = 4.2, 1.7 Hz, 1H), 8.50 – 8.40 (m, 2H), 8.21 (d, *J* = 2.7 Hz, 1H), 7.92 (t, *J* = 5.7 Hz, 1H), 7.73 (dd, *J* = 7.8, 1.1 Hz, 1H), 7.68 (dd, *J* = 8.8, 4.1 Hz, 1H), 7.63 – 7.54 (m, 1H), 7.34 (d, *J* = 8.0 Hz, 1H), 6.91 (d, *J* = 2.7 Hz, 1H), 4.70 (d, *J* = 5.7 Hz, 2H), 2.91 (s, 3H), 2.40 (s, 3H). LCMS *m/z* = 422.3 $[M+H]^+$.

1-(Imidazo[1,2-a]pyridin-2-ylsulfonyl)-N-((5-methylpyridin-2-yl)methyl)-1H-pyrazole-3-carboxamide (9m): Synthesized in a similar manner as **6i** starting from imidazo[1,2-a]pyridine-2-sulfonyl chloride and (5-methylpyridin-2-yl)methanamine to obtain 2.7 mg (9%) of **9m**. LCMS *m/z* = 397.2 $[M+H]^+$.

1-((2,3-Dihydrobenzofuran-5-yl)sulfonyl)-N-((5-methylpyridin-2-yl)methyl)-1H-pyrazole-3-carboxamide (9n): Synthesized in a similar manner as **6i** starting from 2,3-dihydrobenzofuran-5-sulfonyl chloride and (5-methylpyridin-2-yl)methanamine to obtain 6.8 mg (40%) of **9n**. ^{1}H NMR (400 MHz, $CDCl_3$) δ 8.41 (s, 1H), 8.11 (d, *J* = 2.7 Hz, 1H), 7.87-7.83 (m, 2H), 7.75 (t, *J* = 5.7 Hz, 1H), 7.49 (dd, *J* = 1.7, 7.9 Hz, 1H),7.24 (d, *J* = 7.9 Hz, 1H), 6.92 (d, *J* = 2.7 Hz, 1H), 6.89 (d, *J* = 8.2 Hz, 1H), 4.72 (t, *J* = 8.9 Hz, 2H), 4.68 (d, *J* = 5.7 Hz, 2H), 3.30 (t, *J* = 8.9 Hz, 2H), 2.35 (s, 3H). LCMS *m/z* = 399.0 $[M+H]^+$.

N-((5-Methylpyridin-2-yl)methyl)-1-((2,2,4,6,7-pentamethyl-2,3-dihydrobenzofuran-5-yl)sulfonyl)-1H-pyrazole-3-carboxamide (9o): Synthesized in a similar manner as **6i** starting from 2,2,4,6,7-pentamethyl-2,3-dihydrobenzofuran-5-sulfonyl chloride and (5-methylpyridin-2-yl)methanamine to obtain 7.0 mg (15%) of **9o**. ^{1}H NMR (400 MHz, $CDCl_3$) δ 8.28 (s, 1H), 8.09 (d, *J* = 2.7 Hz, 1H), 7.66 (t, *J* = 5.3 Hz, 1H), 7.39 (dd, *J* = 1.9, 7.9 Hz, 1H), 7.16 (d, *J* = 7.9 Hz, 1H), 6.83 (d, *J* = 2.7 Hz, 1H), 4.57 (d, *J* = 5.6 Hz, 2H), 2.94 (s, 2H), 2.48 (s, 3H), 2.45 (s, 3H), 2.25 (s, 3H), 2.04 (s, 3H), 1.42 (s, 6H). LCMS *m/z* = 469.0 $[M+H]^+$.

1-((2,3-Dihydrobenzofuran-5-yl)sulfonyl)-N-((5-methylpyrazin-2-yl)methyl)-1H-pyrazole-3-carboxamide (10a): Synthesized in a similar manner as **6i** starting from 2,3-dihydrobenzofuran-5-sulfonyl chloride and (5-methylpyrazin-2-yl)methanamine to obtain 23.3 mg (82%) of **10a**. ^{1}H NMR (400 MHz, $CDCl_3$) δ 8.51 (s, 1H), 8.40 (s, 1H), 8.10 (d, *J* = 2.7 Hz, 1H), 7.85-7.77 (m, 2H), 7.66-7.60 (m, 1H), 6.90 (d, *J* = 2.7 Hz, 1H), 6.86 (d, *J* = 6.8 Hz), 4.73-4.65 (m, 4H), 3.27 (t, *J* = 8.9 Hz, 2H), 2.55 (s, 1H). LCMS *m/z* = 400.0 $[M+H]^+$.

1-((2,3-Dihydrobenzofuran-5-yl)sulfonyl)-N-((6-methylpyridin-3-yl)methyl)-1H-pyrazole-3-carboxamide (10b): Synthesized in a similar manner as **6i** starting from 2,3-dihydrobenzofuran-5-sulfonyl chloride and (6-methylpyridin-3-yl)methanamine to obtain 11.1 mg (60%) of **10b**. ^{1}H NMR (400 MHz, $CDCl_3$) δ 8.45 (d, *J* = 1.7 Hz, 1H), 8.09 (d, *J* = 2.7 Hz, 1H), 7.81-7.75 (m, 2H), 7.57 (dd, *J* = 2.4 and 7.8 Hz, 1H), 7.21 (t, *J* = 6.0 Hz 1H), 7.12(d, *J* = 8.0 Hz, 1H), 6.91 (d, *J* = 2.7 Hz, 1H), 6.85 (d, *J* = 8.5 Hz, 1H), 4.70 (t, *J* = 8.7 Hz, 2H), 4.55 (d, *J* = 6.2 Hz, 2H), 3.26 (t, *J* = 8.7 Hz, 2H), 2.54 (s, 1H). LCMS *m/z* = 398.9 $[M+H]^+$.

1-((2,3-Dihydrobenzofuran-5-yl)sulfonyl)-N-((6-methylpyridazin-3-yl)methyl)-1H-pyrazole-3-carboxamide (10c): Synthesized in a similar manner as **6i** starting from 2,3-dihydrobenzofuran-5-sulfonyl chloride and (6-methylpyridazin-3-yl)methanamine to obtain 8.8 mg (52%) of **10c**. ^{1}H NMR (400 MHz, $CDCl_3$) δ 8.10 (d, *J* = 2.7 Hz, 1H), 7.84-7.80 (m, 2H), 7.77 (t, *J* = 5.6 Hz 1H) 7.37 (dd, *J* = 8.5 and 63.0 Hz, 2H), 6.89 (d, *J* = 2.7 Hz, 1H), 6.86 (d, *J* = 8.5 Hz, 1H), 4.84 (d, *J* = 6.0 Hz, 2H), 4.70 (t, *J* = 8.8 Hz, 2H), 3.29 (t, *J* = 8.8 Hz, 2H), 2.70 (s, 1H). LCMS *m/z* = 400.0 $[M+H]^+$.

1-((2,3-Dihydrobenzofuran-5-yl)sulfonyl)-N-((6-methoxypyridin-3-yl)methyl)-1H-pyrazole-3-carboxamide (10d): Synthesized in a similar manner as **6i** starting from 2,3-dihydrobenzofuran-5-sulfonyl chloride and (6-methoxypyridin-3-yl)methanamine to obtain 27.6 mg (39%) of (**10d**). ^{1}H NMR (400 MHz, $CDCl_3$) δ 8.43 (bs, 1H), 8.09 (d, *J* = 8.7 Hz, 1H), 8.04 (d, *J* = 2.7 Hz, 1H), 7.76-7.71 (m, 2H), 7.58-7.42 (m, 5H), 6.96 (d, *J* = 8.7 Hz, 1H), 6.82 (d, *J* = 2.7 Hz, 1H), 6.79 (d, *J* = 8.9 Hz, 1H), 4.63 (t, *J* = 8.9 Hz, 2H), 4.53 (d, *J* = 5.8 Hz, 2H), 4.04 (s, 3H), 3.20 (t, *J* = 8.7 Hz, 2H). LCMS *m/z* = 415.0 $[M+H]^+$.

1-((2,3-Dihydrobenzofuran-5-yl)sulfonyl)-N-((3-methylisoxazol-5-yl)methyl)-1H-pyrazole-3-carboxamide (10e): Synthesized in a similar manner as **6i** starting from 2,3-dihydrobenzofuran-5-sulfonyl chloride and (3-methylisoxazol-5-yl)methanamine to obtain 17.6 mg (66%) of **10e**. ^{1}H NMR (400 MHz, $CDCl_3$) δ 8.10 (d, *J* =2.7 Hz. 1H), 7.84-7.78 (m, 2H), 7.70 (t, 1H), 7.30 (t, *J* = 5.8 Hz, 1H), 6.89(d, *J* = 2.7 Hz, 1H), 6.87 (d, *J* = 8.4 Hz, 1H), 6.06 (s,

1H), 4.71 (t, *J* = 8.8 Hz, 2H), 4.64 (d, *J* = 6.0 Hz, 2H), 3.29 (t, *J* = 8.8 Hz, 2H), 2.26 (s,1H). LCMS *m/z* = 389.0 $[M+H]^+$.

1-((2,3-Dihydrobenzofuran-5-yl)sulfonyl)-N-(quinolin-3-ylmethyl)-1H-pyrazole-3-carboxamide (10f): Synthesized in a similar manner as **6i** starting from 2,3-dihydrobenzofuran-5-sulfonyl chloride and quinolin-3-ylmethanamine to obtain 26.3 mg (74%) of **10f**. ^{1}H NMR (400 MHz, $CDCl_3$) δ 8.89 (d, *J* =2.1 Hz. 1H), 8.14-8.08 (m, 3H), 7.78 (t, *J* =8.6 Hz, 2H), 7.71 (t, *J* =7.7 Hz, 1H), 7.55 (t, *J* =7.7 Hz, 1H), 7.33 (t, *J* =6.1 Hz, 1H), 6.94 (d, *J* =2.7 Hz, 1H, 6.85 (d, *J* =8.6 Hz, 1H), 4.78 (d, *J* = 6.3 Hz, 2H), 4.69 (t, *J* = 8.8 Hz, 2H), 3.24 (t, *J* = 8.6 Hz, 2H). LCMS *m/z* = 435.0 $[M+H]^+$.

***1-((2,3-Dihydrobenzofuran-5-yl)sulfonyl)-N-(isoquinolin-3-ylmethyl)-1H-pyrazole-3-carboxamide (10g*)**:** Synthesized in a similar manner as **6i** starting from 2,3-dihydrobenzofuran-5-sulfonyl chloride and quinolin-3-ylmethanamine to obtain 43 mg (59%) of **10g**. ^{1}H NMR (400 MHz, $CDCl_3$) δ 9.54 (s, 1H), 8.62 (bt, *J* = 6.2 Hz, 1H), 8.26 (s, 1H), 8.23 (d, *J* = 8.4 Hz, 1H), 8.04-8.00 (m, 3H), 7.88-7.83 (m, 2H), 7.79 (dd, *J* = 2.0, 8.6 Hz, 1H), 6.80 (d, *J* = 8.6 Hz, 1H), 6.76 (d, *J* = 2.7 Hz, 1H), 4.95 (d, *J* = 6.3 Hz, 2H), 4.63 (t, *J* = 8.8 Hz, 1H), 3.22 (t, *J* = 8.9 Hz, 1H). ^{13}C NMR (100 MHz, $CDCl_3$) δ 166.10, 161.75, 149.65, 147.20, 142.60, 138.86, 136.44, 132.07, 130.81, 130.72, 129.85, 129.30, 127.46, 126.66, 126.62, 126.11, 1254.81, 110.05, 108.10, 72.75, 40.43, 28.60. LCMS *m/z* = 434.9 $[M+H]^+$.

N-((6,7-Dihydro-5H-cyclopenta[b]pyridin-3-yl)methyl)-1-((2,3-dihydrobenzofuran-5-yl)sulfonyl)-1H-pyrazole-3-carboxamide (10h): Synthesized in a similar manner as **6i** starting from 2,3-dihydrobenzofuran-5-sulfonyl chloride and quinolin-3-ylmethanamine to obtain 42 mg (58%) of **10h**. ^{1}H NMR (400 MHz, $CDCl_3$) δ 8.54 (s, 1H), 8.10 (s, 1H), 8.05 (d, *J* = 2.7 Hz, 1H), 7.76-7.73 (m, 2H), 7.55 (bt, *J* = 6.2 Hz, 1H), 6.82 (d, *J* = 2.7 Hz, 1H), 6.81-6.79 (m, 1H), 4.64 (t, *J* = 8.8 Hz, 1H), 4.59 (d, *J* = 6.3 Hz, 2H), 3.26 (t, *J* = 7.8 Hz, 1H), 3.21 (t, *J* = 8.8 Hz, 1H), 3.03 (t, *J* = 7.5 Hz, 1H), 2.26 (p, *J* = 7.6 Hz, 1H). ^{13}C NMR (100 MHz, $CDCl_3$) δ 166.15,

161.18, 159.61, 149.92, 143.56, 140.25, 138.32, 135.79, 132.46, 130.64, 129.37, 126.72, 125.63, 110.17, 108.61, 72.76, 40.02, 30.82, 30.32, 28.65, 23.32. LCMS *m/z* = 425.0 $[M+H]^+$.

N-(Benzo[d]thiazol-2-ylmethyl)-1-((2,3-dihydrobenzofuran-5-yl)sulfonyl)-1H-pyrazole-3-carboxamide (10i): Synthesized in a similar manner as **6i** starting from 2,3-dihydrobenzofuran-5-sulfonyl chloride and benzo[d]thiazol-2-ylmethanamine to obtain 37.4 mg (50%) of **10i** . ^{1}H NMR (400 MHz, $CDCl_3$) δ 8.05 (d, *J* = 2.7 Hz, 1H), 7.95 (d, *J* = 8.2 Hz, 1H), 7.81-7.75 (m, 3H), 7.72 (bt, *J* = 6.2 Hz, 1H), 7.45 (t, *J* = 7.6 Hz, 1H), 7.35 (t, *J* = 7.8 Hz, 1H), 6.86 (d, *J* = 2.7 Hz, 1H), 6.82 (d, *J* = 8.2 Hz, 1H), 4.96 (d, *J* = 6.3 Hz, 2H), 4.63 (t, *J* = 8.8 Hz, 2H), 3.21 (t, *J* = 8.8 Hz, 1H), 3.21 (t, *J* = 8.8 Hz, 1H). ^{13}C NMR (100 MHz, $CDCl_3$) δ 166.01, 160.77, 151.61, 150.16, 132.33, 130.72, 129.18, 126.93, 126.53, 125.71, 125.65, 122.45, 121.77, 110.11, 108.65, 72.69, 40.86, 28.69. LCMS *m/z* = 440.9 $[M+H]^+$.

1-((2,3-Dihydrobenzofuran-5-yl)sulfonyl)-N-(imidazo[1,2-a]pyridin-2-ylmethyl)-1H-pyrazole-3-carboxamide (10j): Synthesized in a similar manner as **6i** starting from 2,3-dihydrobenzofuran-5-sulfonyl chloride and imidazo[1,2-a]pyridin-2-ylmethanamine to obtain 34.0 mg (47%) of **10j**. ^{1}H NMR (400 MHz, $CDCl_3$) δ 8.61 (bs, 1H), 8.25 (d, *J* = 6.2 Hz, 1H), 8.01 (d, *J* = 2.2 Hz, 1H), 7.97 (bd, *J* = 7.6 Hz, 1H), 7.84 (d, *J* = 9.7 Hz, 2H), 7.77 (d, *J* = 8.3 Hz, 1H), 7.71 (t, *J* = 7.2 Hz, 1H), 7.25 (t, *J* = 6.8 Hz, 1H), 6.78 (d, *J* = 8.5 Hz, 2H), 4.78 (d, *J* = 4.4 Hz, 2H), 4.62 (t, *J* = 8.7 Hz, 2H), 3.21 (t, *J* = 8.9 Hz, 2H). ^{13}C NMR (100 MHz, $CDCl_3$) δ 166.11, 135.30, 132.61, 132.06, 130.77, 129.33, 127.19, 126.57, 126.11, 117.23, 113.65, 113.01, 110.03, 108.06, 72.76, 33.65, 28.57. LCMS *m/z* = 423.9 $[M+H]^+$.

N-((5-Methylpyrazin-2-yl)methyl)-1-((2,2,4,6,7-pentamethyl-2,3-dihydrobenzofuran-5-yl)sulfonyl)-1H-pyrazole-3-carboxamide (11a): Synthesized in a similar manner as **6i** starting from 2,2,4,6,7-pentamethyl-2,3-dihydrobenzofuran-5-sulfonyl chloride and (5-methylpyrazin-2-yl)methanamine to obtain 22 mg (47%) of **11a**. ^{1}H NMR (400 MHz, $CDCl_3$) δ 8.45 (s, 1H), 8.30 (s, 1H), 8.09 (d, *J* = 2.7 Hz, 1H), 7.52 (bs, 1H), 6.83 (d, *J* = 2.7 Hz, 1H), 4.61

(d, J = 5.9 Hz, 2H), 2.94 (s, 2H), 2.48 (s, 3H), 2.47 (s, 3H), 2.45 (s, 3H), 2.04 (s, 3H) 1.42 (s, 6H). LCMS m/z = 470.0 $[M+H]^+$.

***N*-((6-Methylpyridin-3-yl)methyl)-1-((2,2,4,6,7-pentamethyl-2,3-dihydrobenzofuran-5-yl)sulfonyl)-1*H*-pyrazole-3-carboxamide (11b):** Synthesized in a similar manner as **6i** starting from 2,3-dihydrobenzofuran-5-sulfonyl chloride and (6-methylpyridin-3-yl)methanamine to obtain 13.0 mg (34%) of **11b**. ^{1}H NMR (400 MHz, $CDCl_3$) δ 8.37 (d, J = 2.0 Hz, 1H), 8.08 (d, J = 2.8 Hz, 1H), 7.51 (dd, J = 2.2, 7.9 Hz, 1H), 7.06-7.01 (m, 2H), 6.85 (d, J = 2.7 Hz, 1H), 4.48 (d, J = 6.2 Hz, 2H), 2.92 (s, 2H), 2.47 (s, 3H), 2.43 (s, 3H), 2.41 (s, 3H), 2.02 (s, 3H) 1.41 (s, 6H). LCMS m/z = 469.0 $[M+H]^+$.

***N*-((6-Methylpyridazin-3-yl)methyl)-1-((2,2,4,6,7-pentamethyl-2,3-dihydrobenzofuran-5-yl)sulfonyl)-1*H*-pyrazole-3-carboxamide (11c):** Synthesized in a similar manner as **6i** starting from 2,2,4,6,7-pentamethyl-2,3-dihydrobenzofuran-5-sulfonyl chloride and (6-methylpyridazin-3-yl)methanamine to obtain 10.0 mg (26%) of **11c**. ^{1}H NMR (400 MHz, $CDCl_3$) δ 8.11 (d, J = 2.7 Hz, 1H), 7.54 (bt, J = 5.9 Hz, 1H), 7.42 (d, J = 8.6 Hz, 1H), 7.22 (d, J = 8.5 Hz, 1H), 6.83 (d, J = 2.7 Hz, 1H), 4.77 (d, J = 6.2 Hz, 2H), 2.96 (s, 2H), 2.62 (s, 3H), 2.47 (s, 3H), 2.41 (s, 3H), 2.03 (s, 3H) 1.42 (s, 6H). LCMS m/z = 470.0 $[M+H]^+$.

***N*-((3-Methylisoxazol-5-yl)methyl)-1-((2,2,4,6,7-pentamethyl-2,3-dihydrobenzofuran-5-yl)sulfonyl)-1*H*-pyrazole-3-carboxamide (11e):** Synthesized in a similar manner as **6i** starting from 2,2,4,6,7-pentamethyl-2,3-dihydrobenzofuran-5-sulfonyl chloride and (3-methylisoxazol-5-yl)methanamine to obtain 16.0 mg (42%) of **11e**. ^{1}H NMR (400 MHz, $CDCl_3$) δ 8.10 (d, J = 2.7 Hz, 1H), 7.11 (bt, J = 6.1 Hz, 1H), 6.83 (d, J = 2.7 Hz, 1H), 5.99 (s, 1H), 4.57 (d, J = 6.2 Hz, 2H), 2.95 (s, 2H), 2.46 (s, 3H), 2.42 (s, 3H), 2.19 (s, 3H), 2.04 (s, 3H) 1.43 (s, 6H).

1-((2,2,4,6,7-Pentamethyl-2,3-dihydrobenzofuran-5-yl)sulfonyl)-N-(quinolin-3-ylmethyl)-1H-pyrazole-3-carboxamide (11f): Synthesized in a similar manner as **6i**

starting from 2,2,4,6,7-pentamethyl-2,3-dihydrobenzofuran-5-sulfonyl chloride and quinolin-3-ylmethanamine to obtain 18.6 mg (62%) of **11f**. ^{1}H NMR (400 MHz, $CDCl_3$) δ 8.87 (d, *J* = 2.1 Hz. 1H), 8.17 (d, *J* = 2.8 Hz, 1H), 8.12-8.06 (m, 2H), 7.78 (d, *J* = 8.1 Hz, 1H), 7.70 (t, *J* = 7.6 Hz, 1H), 7.55 (t, *J* = 7.6 Hz, 1H), 7.33 (t, 1H), 6.95 (d, *J* = 2.7 Hz, 1H), 4.80-4.76 (m, 2H), 2.97 (s, 2H), 2.50 (s, 3H). 2.48 (s, 3H), 2.08 (s, 3H), 1.46 (s, 6H). LCMS *m/z* = 505.0 $[M+H]^+$.

N-(Imidazo[1,2-a]pyridin-2-ylmethyl)-1-((2,2,4,6,7-pentamethyl-2,3-dihydrobenzofuran-5-yl)sulfonyl)-1H-pyrazole-3-carboxamide (11j): Synthesized in a similar manner as **6i** starting from 2,2,4,6,7-pentamethyl-2,3-dihydrobenzofuran-5-sulfonyl chloride and imidazo[1,2-a]pyridin-2-ylmethanamine to obtain 32.3 mg (48%) of **11j**. ^{1}H NMR (400 MHz, MeOD) δ 8.76 (d, *J* = 6.7 Hz, 1H), 8.16 (s, 1H), 7.99-7.94 (m, 1H), 7.89 (d, *J* = 8.9 Hz, 1H), 7.76 (s, 1H), 7.48 (dt, *J* = 1.0, 6.7 Hz, 1H), 6.84, (s, 1H), 6.43 (s, 1H). 4.84 (s, 2H), 2.90 (s, 2H), 2.16 (s, 3H), 2.12 (s, 3H), 2.02 (s, 3H), 1.44 (s, 6H). LCMS *m/z* = 494.0 $[M+H]^+$.

Scheme S1. Preparation of analogs **6b**, **6c**, and **6f**. [a]

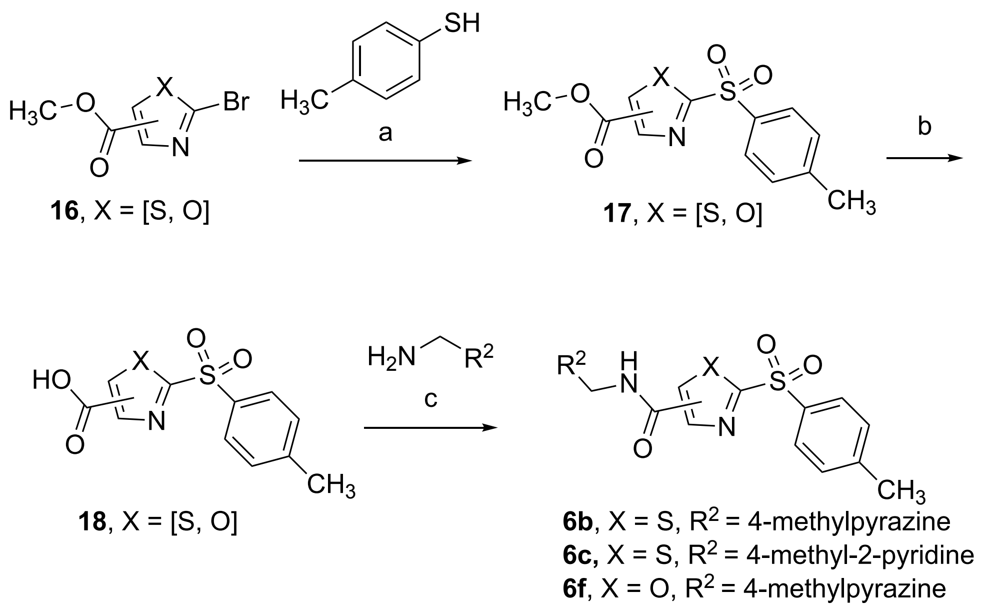

[a]*Reagents and Conditions*: a) Cs_2CO_3, dioxane, H_2O, 6h, then Oxone,16h, 34-71%; b) LiOH, dioxane, H_2O, 16h, 88%; c) HATU, DMF, 30 min, 63-72%.

Synthesis of N-((5-Methylpyrazin-2-yl)methyl)-2-tosylthiazole-4-carboxamide (6b)

Methyl 2-(4-methylphenyl)sulfonyl-1,3-thiazole-4-carboxylate: methyl 2-bromothiazole-4-carboxylate (268 mg, 1 Eq,1.21 mmol), p-thiocresol (150 mg, 1 Eq, 1.21 mmol) and Cs_2CO_3 (1.19g, 3 Eq, 3.62 mmol) in 1,4-dioxane (6 mL) was stirred at RT for 6 hr. The mixture was filtered then 1,4-dioxane (1 mL), H_2O (1 mL) and potassium peroxymonosulfate (1.87 g, 2.5 Eq, 3.02 mmol) were added and the mixture stirred at RT for 16 hr. The mixture was filtered and the filtrate was purified by flash chromatography eluting with 0-80% EtOAc in hexanes to afford methyl 2-(4-methylphenyl)sulfonyl-1,3-thiazole-4-carboxylate (255mg, 71% yield.) LCMS *m/z* = 312.1 $[M+H]^+$.

Lithium 2-(4-methylphenyl)sulfonyl-1,3-thiazole-4-carboxylate: methyl 2-(4-methylphenyl)sulfonyl-1,3-thiazole-4-carboxylate (255 mg, 1 Eq, 0.86 mmol) and lithium hydroxide (42 mg, 2 Eq, 1.72 mmol) in 1,4-dioxane (2.5 mL) and H_2O (0.5 mL) was stirred at RT for 16 hr. The suspension was diluted with dioxane (4 mL) then the solids were collected by suction filtration, washed with dioxane then dried. The crude product was taken on to the next step without further purification. LCMS *m/z* = 284.1 $[M+H]^+$.

N-((5-Methylpyrazin-((5-methylpyridin-2-yl)methyl)-2-tosylthiazole-4-carboxamiden-2-yl)methyl)-2-tosyl-3H-pyrrole-5-carboxamide ***(6b):*** 2-(aminomethyl)-5-methylpyrazine (8.5 mg, 1 Eq, 0.07 mmol), lithium 2-(4-methylphenyl)sulfonyl-1,3-thiazole-4-carboxylate (20 mg, 0.07 mmol), DIEA (24 μL, 2 Eq, 0.14 mmol) and HATU (39 mg, 1.5 Eq, 0.1 mmol) in DMF (0.25 mL) was stirred at RT for 30 min. The solution was purified by semi-prep HPLC (eluting with 2-55% MeCN in 0.05% NH_4OH (aq)) to afford **6b** (19 mg, 71%.) ^1H NMR (400 MHz, $CDCl_3$) δ 8.55 (d, *J* = 1.5 Hz, 1H), 8.50 (d, *J* = 1.6 Hz, 1H), 8.41 (s, 1H), 8.01 – 7.96 (m, 3H), 7.45 – 7.38 (d, *J* = 7.8 Hz, 1H), 4.77 (d, *J* = 5.9 Hz, 2H), 2.64 (s, 2H). LCMS *m/z* = 389.1 $[M+H]^+$.

N-((5-Methylpyridin-2-yl)methyl)-2-tosylthiazole-4-carboxamide (6c): Synthesized in a similar manner as **6b** utilizing (5-methylpyridin-2-yl)methanamine to obtain 19 mg (71%) of **6c**. LCMS *m/z* = 389.1 $[M+H]^+$.

Scheme S2. Preparation of analogs **6d** and **6e**.

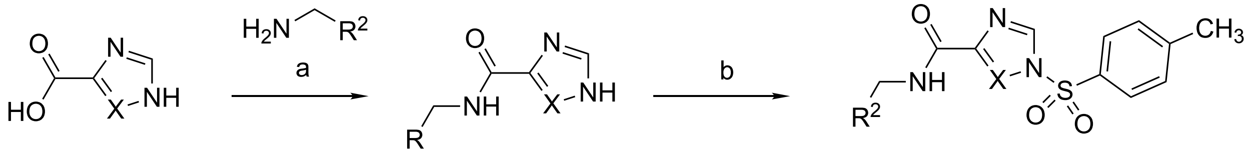

19, X= [CH, N]

20, X= [CH, N]

6d, X= N, R^2 = 4-methyl-2-pyridyl
6e, X= CH, R^2 = 4-methylpyrazinyl

Reagents and Conditions: a) EDCI, HOBT, DIEA, DMF, 1h, 31-50%; b) tosyl chloride,

Synthesis of N-((5-methylpyridin-2-yl)methyl)-2-tosylthiazole-4-carboxamide (6d)

N-[(5-Methyl-2-pyridinyl)methyl]-1H-1,2,4-triazole-3-carboxamide: (5-methylpyridin-2-yl)methanamine (54 mg, 1 Eq, 0.44 mmol), 1-(3-dimethylaminopropyl)-3-ethylcarbodiimide hydrochloride (127 mg, 0.66 mmol), 1-hydroxbenzotriazole hydrate (101 mg, 1.5 Eq, 0.66 mmol), 1,2,4-triazole-3-carboxylic acid (50 mg, 1 Eq, 0.44 mmol), and DIEA (0.15 mL, 2 Eq, 0.88 mmol) in DMF (1.5 mL) was stirred at RT for 16 hr. The solution was then purified by semi-prep HPLC (eluting with 0-30% MeCN in 0.05% NH_4OH (aq)) to afford *N-[(5-methyl-2-pyridinyl)methyl]-1H-1,2,4-triazole-3-carboxamide* (62 mg, 65%.) LCMS *m/z* = 218.2 $[M+H]^+$.
N-((5-Methylpyridin-2-yl)methyl)-2-tosylthiazole-4-carboxamide (**6d**): Tosyl chloride (22 mg, 1.0 Eq, 0.12 mmol) was added to N-[(5-methyl-2-pyridinyl)methyl]-1H-1,2,4-triazole-3-carboxamide (25 mg, 1 Eq, 0.12 mmol) and DIEA (40 μL, 2 Eq, 0.23 mmol) in DMF (0.5 mL) then the solutions were stirred at RT for 30 min. The solution was purified by semi-prep HPLC (eluting with 2-60% MeCN in 0.05% NH_4OH (aq)) to afford **6d** (13 mg, 30% yield.) ^1H NMR (400 MHz, DMSO) δ 9.34 (s, 1H), 8.64 (s, 1H), 8.22 – 8.15 (m, 1H), 7.72 (d, *J* = 8.3 Hz, 1H), 7.48 (d, *J* = 8.1 Hz, 2H), 7.13 (d, *J* = 8.0 Hz, 2H), 4.71 (d, *J* = 6.0 Hz, 2H), 2.55 (s, 3H), 2.29 (s, 3H). LCMS *m/z* = 372.2 $[M+H]^+$.

N-((5-Methylpyrazin-2-yl)methyl)-1-tosyl-1H-imidazole-4-carboxamide (6e): Synthesized in a similar manner as **6d** starting from imidazole-4-carboxylic acid and utilizing (5-methylpyrazin-2-yl)methanamine to obtain 21 mg (50%) of **6e**. LCMS *m/z* = 372.3 $[M+H]^+$.

N-((5-Methylpyrazin-2-yl)methyl)-2-tosyloxazole-5-carboxamide (6f): Synthesized in a similar manner as **6b** starting from methyl 2-bromooxazole-5-carboxylate and utilizing (5-methylpyrazin-2-yl)methanamine to obtain 2.2 mg (8%) of **6f**. ^{1}H NMR (400 MHz, $CDCl_3$) δ 8.55 (d, *J* = 10.0 Hz, 2H), 8.31 (s, 1H), 8.03 – 7.93 (m, 2H), 7.77 – 7.71 (m, 1H), 7.44 (d, *J* = 8.0 Hz, 2H), 4.76 (d, *J* = 5.6 Hz, 2H), 2.66 (s, 3H), 2.50 (s, 3H).
LCMS *m/z* = 373.2 $[M+H]^+$.

Scheme S3.[a] Preparation of pyrazololactam **6j**.

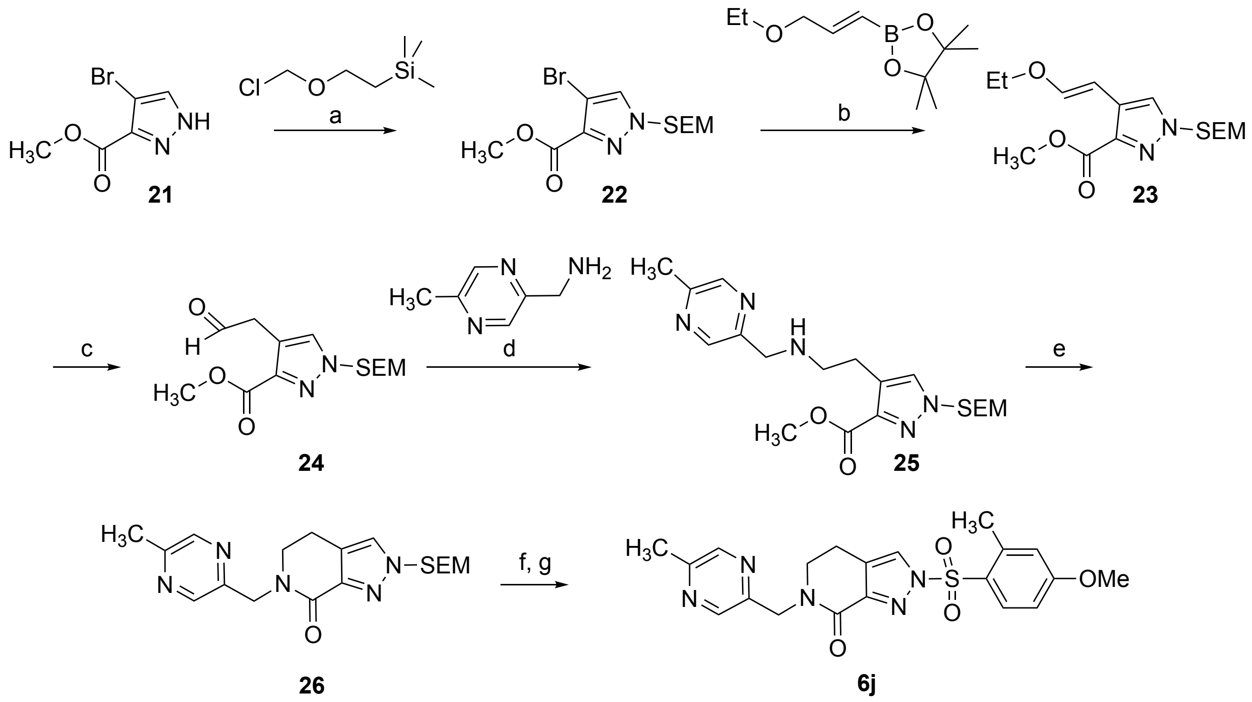

[a]*Reagents and Conditions*: a) NaH, THF, 84%; b) $Pd_2(dba)_3$, XPhos, K_2CO_3, dioxane, H_2O, 80°C, 3h, 82%; c) 4M HCl, THF, 6h, 16%; d) $NaBH(OAc)_3$, AcOH, MeOH, 3h, 23 %; e) Cs_2CO_3, MeOH, 20h, 60%; f) TFA, TES, DCE, 50 °C, 1h; g) 4-methoxy-2-methylbenzenesulfonyl chloride, Cs_2CO_3, DMF, rt, 18h, 22%.

Synthesis of 2-((4-methoxy-2-methylphenyl)sulfonyl)-6-((5-methylpyrazin-2-yl)methyl)-2,4,5,6-tetrahydro-7H-pyrazolo[3,4-c]pyridin-7-one (6j)

*Methyl 4-bromo-1-((2-(trimethylsilyl)ethoxy)methyl)-1H-pyrazole-3-carboxylate (**22**):* A solution of 4-bromo-1H-pyrazole-3-carboxylic acid methyl ester (1.00 g, 1 Eq, 4.88 mmol) in THF (15 mL) was cooled to 0 °C. At this temperature NaH (293 mg, 60% Wt, 1.5 Eq, 7.32 mmol) was added and the reaction mixture was kept cold while stirring for 30 min. At this time, 2-(chloromethoxyethyl)trimethyl silane (1.22 g, 1.29 mL, 1.5 Eq, 7.32 mmol) was added. The reaction mixture was allowed to warm to rt and stirred at 23 °C for 16 h, then quenched by the addition of satd. aq. NH_4Cl mL, followed by EtOAc (15 mL). The organic phase was separated, washed with water, dried over $MgSO_4$, and filtered. The filter cake was washed with EtOAc and the combined organics were concentrated under reduced pressure. The crude product was purified by ISCO column chromatography eluting with 0 to 15% EtOAc in Hexane to afford **22** (1.38 g, 84%). ^{1}H NMR (400 MHz, $CDCl_3$) δ 7.71 (s, 1H), 5.48 (s, 2H), 3.97 (s, 3H), 3.61-3.56 (m, 2H), 0.95-0.91 (m, 2H), 0.00 (s, 9H). LCMS *m/z* = 334.9 $[M+H]^+$.

*Methyl (E)-4-(2-ethoxyvinyl)-1-((2-(trimethylsilyl)ethoxy)methyl)-1H-pyrazole-3-carboxylate (**23**):* To a solution of methyl 4-bromo-1-((2-(trimethylsilyl)ethoxy)methyl)-1H-pyrazole-3-carboxylate (500.00 mg, 1 Eq, 1.4913 mmol) in 1,4-dioxane (10 mL) and water (2.5 mL) was added 2-(dicyclohexylphosphanyl)-2',4',6'-tris(isopropyl)biphenyl (213.29 mg, 0.3 Eq, 447.40 µmol), tris((1E,4E)-1,5-diphenylpenta-1,4-dien-3-one) dipalladium (136.57 mg, 0.1 Eq, 149.13 µmol), and K_2CO_3 (618.31 mg, 3 Eq, 4.4740 mmol) at rt. The mixture was degassed by allowing Ar gas to bubble through the solution for 20 min, then heated at 80 °C for 3 hours. Water and brine were added and the mixture was extracted with EtOAc (3x). The combined organic phases were dried over Na_2SO_4 and filtered, then concentrated under reduced pressure. The crude product was purified by ISCO column chromatography eluting with 0 to 13% EtOAc in Hexane to afford **23** (399.0 mg, 82%). LCMS *m/z* = 327.1 $[M+H]^+$.

*Methyl 4-(2-oxoethyl)-1-((2-(trimethylsilyl)ethoxy)methyl)-1H-pyrazole-3-carboxylate (**24**):* To a solution of methyl (E)-4-(2-ethoxyvinyl)-1-((2-(trimethylsilyl)ethoxy)methyl)-1H-pyrazole-3-carboxylate (180.0 mg, 1 Eq, 551.4 µmol) in THF (2 mL) was slowly added HCl (201.0 mg, 1.378 mL, 4 molar, 10 Eq, 5.514 mmol). The reaction mixture was allowed to stir at rt for 1 hour. The reaction mixture was then slowly added to a stirring satd. aq. $NaHCO_3$

solution. The mixture was extracted with EtOAc (3x) and the combined organic phases were dried over Na_2SO_4, filtered, and concentrated under reduced pressure to afford **24** (165 mg,). ^{1}H NMR (400 MHz, $CDCl_3$) δ 9.79 (t, *J* = 1.3 Hz, 1H), 7.68 (s, 1H), 5.50 (s, 2H), 3.96-3.94 (m, 5H), 3.72 (s, 3H), 3.63-3.58 (m, 2H), 0.96-0.91 (m, 2H), 0.00 (s, 9H). LCMS *m/z* = 299.0 $[M+H]^+$.

*Methyl 4-(2-(((5-methylpyrazin-2-yl)methyl)amino)ethyl)-1-((2-(trimethylsilyl)ethoxy)methyl)-1H-pyrazole-3-carboxylate (**25**):* To a solution of methyl 4-(2-oxoethyl)-1-((2-(trimethylsilyl)ethoxy)methyl)-1H-pyrazole-3-carboxylate (112.0 mg, 1 Eq, 375.3 μmol) in MeOH (2 mL) was added (5-Methylpyrazin-2-yl)methanamine (50.85 mg, 46.56 μL, 1.1 Eq, 412.9 μmol), AcOH (67.61 mg, 64.46 μL, 3 Eq, 1.126 mmol) and sodium triacetoxyborohydride (238.6 mg, 3 Eq, 1.126 mmol). The reaction mixture was allowed to stir at rt for 3 hours, then slowly added to a stirring satd. aq. $NaHCO_3$ solution. The mixture was extracted with EtOAc (3x) and the combined organic phases were dried over Na_2SO_4, filtered, and concentrated under reduced pressure. The crude product was purified by ISCO column chromatography eluting with 0 to 40% EtOAc in Hexane to afford **25** (36.0 mg, 23%). ^{1}H NMR (400 MHz, MeOD) δ 8.52 (d, *J* = 5.0 Hz, 2H), 7.80 (s, 1H), 5.47 (s, 2H), 3.97 (s, 2H), 3.91 (s, 3H), 3.65-3.60 (m, 2H), 3.02 (d, *J* = 7.2 Hz, 2H), 2.90 (d, *J* = 7.2 Hz, 2H), 2.58 (s, 3H), 0.94-0.89 (m, 2H), 0.00 (s, 9H). LCMS *m/z* = 406.1 $[M+H]^+$.

*6-((5-Methylpyrazin-2-yl)methyl)-2-((2-(trimethylsilyl)ethoxy)methyl)-2,4,5,6-tetrahydro-7H-pyrazolo[3,4-c]pyridin-7-one (**26**):* To a solution of methyl 4-(2-(((5-methylpyrazin-2-yl)methyl)amino)ethyl)-1-((2-(trimethylsilyl)ethoxy)methyl)-1H-pyrazole-3-carboxylate (36.00 mg, 1 Eq, 88.76 μmol) in MeOH (1.5 mL) was slowly added Cs_2CO_3 (115.7 mg, 4 Eq, 355.1 μmol). The reaction mixture was allowed to stir at rt for 18 hours, then concentrated under reduced pressure and purified by ISCO column chromatography eluting with 0 to 2% MeOH (10% in DCM) in DCM to afford **26** (20 mg, 60%). ^{1}H NMR (400 MHz, $CDCl_3$) δ 8.62 (d, *J* = 1.1 Hz, 1H), 8.39 (d, *J* = 1.1 Hz, 1H), 7.43 (s, 1H), 5.50 (s, 2H), 4.91 (s, 2H), 3.69 (t, *J* = 6.6 Hz, 2H), 3.63-3.58 (m, 2H), 2.87 (d, *J* = 6.6 Hz, 2H), 2.57 (s, 3H), 0.95-0.90 (m, 2H), 0.00 (s, 9H). LCMS *m/z* = 374.1 $[M+H]^+$.

6-((5-Methylpyrazin-2-yl)methyl)-2,4,5,6-tetrahydro-7H-pyrazolo[3,4-c]pyridin-7-one: To a solution of 6-((5-methylpyrazin-2-yl)methyl)-2-((2-(trimethylsilyl)ethoxy)methyl)-2,4,5,6-tetrahydro-7H-pyrazolo[3,4-c]pyridin-7-one (20.0 mg, 1 Eq, 53.5 μmol) in 1,2-dichloroethane (0.5 mL) was slowly added TFA (0.7 g, 0.5 mL, 100 Eq, 6 mmol) and triethylsilane (0.1 g, 0.2 mL, 20 Eq, 1 mmol). This reaction mixture was allowed to heat at 50°C for 1 hour, then concentrated under reduced pressure. The residue was further diluted with DCM (3x) and concentrated to remove any excess TFA to afford *6-((5-methylpyrazin-2-yl)methyl)-2,4,5,6-tetrahydro-7H-pyrazolo[3,4-c]pyridin-7-one* which was used directly in the next step with no further purification.

*2-((4-Methoxy-2-methylphenyl)sulfonyl)-6-((5-methylpyrazin-2-yl)methyl)-2,4,5,6-tetrahydro-7H-pyrazolo[3,4-c]pyridin-7-one (**6j**)***:** To a solution of 6-((5-methylpyrazin-2-yl)methyl)-2,4,5,6-tetrahydro-7H-pyrazolo[3,4-c]pyridin-7-one (13.00 mg, 1 Eq, 53 μmol) in DMF (1.5 mL) was slowly added Cs_2CO_3 (52 mg, 3 Eq, 160 μmol) followed by (4-methoxy-2-methylbenzene sulfonyl chloride (14.15 mg, 14 μL, 1.2 Eq, 64 μmol). The reaction mixture was allowed to stir at rt for 18 hours. Water and brine were added, and the mixture was extracted with EtOAc (3x). The combined organic phases were dried over Na_2SO_4, filtered, concentrated under reduced pressure. The crude product was purified by ISCO column chromatography eluting with 0 to 2% MeOH (10% in DCM) in DCM to afford 5.0 mg (22%) of **6j**. ^{1}H NMR (400 MHz, $CDCl_3$) δ 8.48 (s, 1H), 8.28 (s, 1H), 8.08 (d, *J* = 9.0 Hz, 1H), 7.92 (s, 1H), 6.78 (dd, *J* = 2.5, 9.0 Hz, 1H), 6.69 (d, *J* = 2.4 Hz, 1H), 4.76 (s, 2H), 3.78 (s, 3H), 3.62 (t, *J* = 6.6 Hz, 2H), 2.78 (t, *J* = 6.6 Hz, 1H), 2.49 (s, 3H), 2.47 (s, 3H). LCMS *m/z* = 428.0 $[M+H]^+$.

Scheme S4.[a] Preparation of pyrrololactam **6l**.

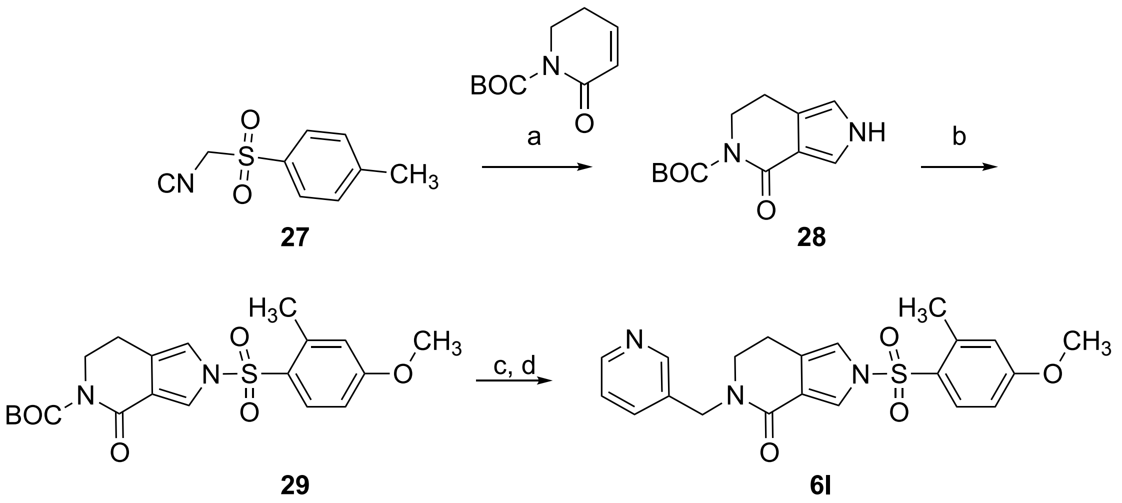

[a]*Reagents and Conditions*: a) DBU, THF, 57 %; b) Cs_2CO_3, DMF, 31%; c) TFA, DCM, 98 %; d) NaHMDS, THF, 38°C, 32%.

Synthesis of 2-((4-methoxy-2-methylphenyl)sulfonyl)-5-(pyridin-3-ylmethyl)-2,5,6,7-tetrahydro-4H-pyrrolo[3,4-c]pyridin-4-one (6l)

*tert-Butyl 4-oxo-2,4,6,7-tetrahydro-5H-pyrrolo[3,4-c]pyridine-5-carboxylate (**28**):* To a solution of tosylmethyl isocyanide (272 mg, 1.1 Eq, 1.39 mmol) in THF (4 mL) was added DBU (212 mg, 210 µL, 1.1 Eq, 1.39 mmol). The reaction mixture was allowed to stir at rt for 15 min, at which time *tert*-butyl 2-oxo-5,6-dihydropyridine-1(2H)-carboxylate (250 mg, 1 Eq, 1.27 mmol) was. The reaction mixture was allowed to stir at rt for 12 hours. Brine was added and the mixture was extracted with EtOAc (3x). The combined organic phases were concentrated under reduced pressure and concentrated under reduced pressure. The crude product was purified by ISCO column chromatography eluting with 0 to 75% EtOAc in Hexane to afford 170 mg (57%) of **28**. ^{1}H NMR (400 MHz, DMSO-d_6) δ 11.38 (br s, 1H), 7.34 (m, 1H), 6.61 (s, 1H), 3.81 (t, J = 6.0 Hz, 2H), 2.67 (t, J = 6.0 Hz, 2H), 1.44 (s, 9H). No ionization by LCMS, MS APCI neg. 235 [M^+-I].

*tert-Butyl 2-((4-methoxy-2-methylphenyl)sulfonyl)-4-oxo-2,4,6,7-tetrahydro-5H-pyrrolo[3,4-c]pyridine-5-carboxylate (**29**):* To a solution of tert-butyl 4-oxo-2,4,6,7-tetrahydro-5H-pyrrolo[3,4-c]pyridine-5-carboxylate (50 mg, 1 Eq, 0.21 mmol) in DMF (1 mL) at rt was

added Cs_2CO_3 (0.21 g, 3 Eq, 0.63 mmol), followed by 4-methoxy-2-methylbenzene sulfonyl chloride (51 mg, 1.1 Eq, 0.23 mmol). The reaction mixture heated to 60 °C and stirred at this temperature for 12 hours. The mixture was allowed to cool to rt and satd. aq. NH_4Cl was added, followed by EtOAc (15 mL). The combined organic phases were dried over Na_2SO_4, filtered, and concentrated under reduced pressure. The crude product was purified by ISCO column chromatography eluting with 0 to 45% EtOAc in Hexane to afford **29** (28 mg, 31%). ^1H NMR (400 MHz, $CDCl_3$) δ 7.91 (d, *J* = 8.9 Hz, 1H), 7.66 (d, *J* = 2.2 Hz, 1H), 6.81-6.79 (m, 1H), 6.77 (dd, *J* = 2.5, 8.9 Hz, 1H), 6.70 (d, *J* = 2.3 Hz, 1H), 3.88 (t, *J* = 6.1 Hz, 1H), 3.79 (s, 3H), 2.67 (t, *J* = 5.9 Hz, 2H), 2.41 (s, 3H), 1.47 (s, 9H). LCMS *m/z* = 364.9 $[M+H]^+$.

2-((4-Methoxy-2-methylphenyl)sulfonyl)-2,5,6,7-tetrahydro-4H-pyrrolo[3,4-c]pyridin-4-one: To a solution of tert-butyl 2-((4-methoxy-2-methylphenyl)sulfonyl)-4-oxo-2,4,6,7-tetrahydro-5H-pyrrolo[3,4-c]pyridine-5-carboxylate (28 mg, 1 Eq, 67 μmol) in DCM (1 mL) was added TFA (76 mg, 51 μL, 10 Eq, 0.67 mmol) at rt. The reaction mixture was allowed to stir at rt for 2 hours. Satd. aq. NH_4Cl was added, followed by EtOAc (15 mL). The mixture was extracted with EtOAc and the combined organic phases were dried over Na_2SO_4, filtered, and concentrated under reduced pressure. The crude product was purified by ISCO column chromatography eluting with 0 to 40% MeOH in DCM to afford 2-((4-Methoxy-2-methylphenyl)sulfonyl)-2,5,6,7-tetrahydro-4H-pyrrolo[3,4-c]pyridin-4-one (21 mg, 98%). ^1H NMR (400 MHz, $CDCl_3$) δ 8.01 (d, *J* = 8.9 Hz, 1H), 7.67 (d, *J* = 2.1 Hz, 1H), 7.05-7.02 (m, 1H), 6.98 (dd, *J* = 2.6, 8.9 Hz, 1H), 6.93 (d, *J* = 2.2 Hz, 1H), 3.88 (s, 3H), 3.45 (t, *J* = 6.5 Hz, 2H), 2.76-2.72 (m, 2H), 2.51 (s, 3H). LCMS *m/z* = 321.0 $[M+H]^+$.

*2-((4-Methoxy-2-methylphenyl)sulfonyl)-5-(pyridin-3-ylmethyl)-2,5,6,7-tetrahydro-4H-pyrrolo[3,4-c]pyridin-4-one (**6l**)*:To a solution of 2-((4-methoxy-2-methylphenyl)sulfonyl)-2,5,6,7-tetrahydro-4H-pyrrolo[3,4-c]pyridin-4-one (20 mg, 1 Eq, 62 μmol) in THF (1 mL) was added NaHMDS (69 mg, 0.37 mL, 1 molar in THF, 6 Eq, 0.37 mmol) at 0°C. The reaction mixture was stirred at this temperature for 10 min, then 3-(bromomethyl)pyridine hydrobromide (39 mg, 2.5 Eq, 0.16 mmol) was added. The reaction mixture was heated to 38 °C and stirred at this temperature for 12 hours. The reaction mixture was allowed to cool to rt and satd. aq. NH_4Cl was added, followed by EtOAc (15 mL). The mixture was extracted

with EtOAc and the combined organic phases were dried over Na_2SO_4, filtered, and concentrated under reduced pressure. The crude product was purified by reverse phase ISCO column chromatography eluting with 0 to 40% AcCN in DCM to afford 10 mg (32%) of **6l**. ^{1}H NMR (400 MHz, MeOD) δ 8.77 (d, *J* = 25.4 Hz, 2H), 8.48 (d, *J* = 8.3 Hz, 1H), 8.04 (d, *J* = 8.8 Hz, 1H), 7.97 (bt, *J* = 6.3 Hz, 1H), 7.71 (d, *J* = 2.1 Hz, 1H), 7.07-7.04 (m, 1H), 6.99 (dd, *J* = 2.5, 8.8 Hz, 1H), 6.94 (d, *J* = 2.5 Hz, 1H), 3.89 (s, 3H), 3.67 (t, *J* = 6.6 Hz, 2H), 2.86 (d, *J* = 6.4 Hz, 2H), 2.52 (s, 3H). LCMS *m/z* = 412.0 $[M+H]^+$.

Scheme S5. [a] Preparation of analog **7d** and **8e**.

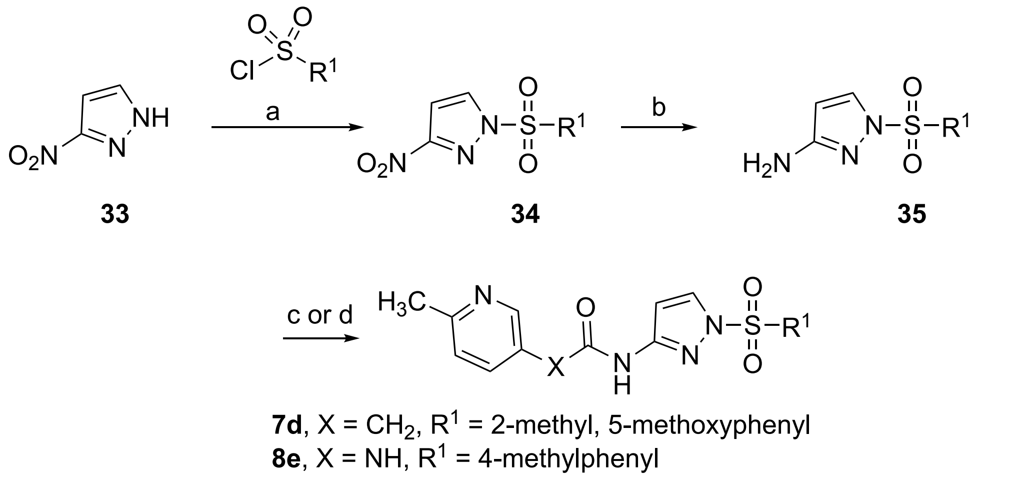

[a]*Reagents and Conditions*: a) DIPEA, DMF, rt, b) Pd/C, H_2, EtOH, rt, c) For **7d:** 2-(6-methylpyridin-3-yl)acetic acid, HATU, DMF, rt, 48%, d) for **8e:** p-nitrophenyl chloroformate, pyridine, rt, 9%.

N-(1-((4-Methoxy-2-methylphenyl)sulfonyl)-1H-pyrazol-3-yl)-2-(6-methylpyridin-3-yl)acetamide (7d):

1-((4-methoxy-2-methylphenyl)sulfonyl)-3-nitro-1H-pyrazole: 4-methoxy-2-methylbenzene-1-sulfonyl chloride (195 mg, 1.0 Eq, 0.88 mmol) was added to 3-nitro-1H-pyrazole **33** (100 mg, 1.0 Eq., 0.88 mmol) and DIEA (0.39 mL, 3.0 Eq, 2.21 mmol) in DMF (3 mL) and the solution was stirred at RT for 18 hr. The solution was diluted with EtOAc, washed with H_2O (2x) and brine, dried over Na_2SO_4 , and concentrated. The residue was purified by flash chromatography (eluting with 0-40% EtOAc in hexanes) to give 1-(4-methoxy-2-methylphenyl)sulfonyl-3-nitropyrazole (150 mg, 57% yield). ^{1}H NMR (400 MHz, $CDCl_3$) δ

8.24 (d, *J* = 2.8 Hz, 1H), 8.20 (d, *J* = 9.0 Hz, 1H), 6.99 (d, *J* = 2.8 Hz, 1H), 6.92 (dd, *J* = 9.0, 2.6 Hz, 1H), 6.85 (dd, *J* = 2.6, 0.8 Hz, 1H), 3.90 (s, 3H), 2.64 (s, 3H).

1-(4-methoxy-2-methylphenyl)sulfonylpyrazol-3-amine: 1-(4-methoxy-2-methylphenyl)sulfonyl-3-nitropyrazole (150 mg, 1.0 Eq, 0.5 mmol) and Pd/C (80 mg, 0.2 Eq, 0.08 mmol) in Ethanol (4 mL) was stirred vigorously under H_2 (1 atm) at RT for 3 hr. The mixture was filtered through a pad of Celite then concentrated to give 1-(4-methoxy-2-methylphenyl)sulfonylpyrazol-3-amine (98 mg, 72% yield), which was used without further purification. LCMS *m/z* = 268.3 $[M+H]^+$.

N-(1-((4-methoxy-2-methylphenyl)sulfonyl)-1H-pyrazol-3-yl)-2-(6-methylpyridin-3-yl)acetamide ***(7d):*** HATU (15.6 mg,1.1 Eq, 0.04 mmol) was added to 2-(6-methylpyridin-3-yl)acetic acid (5.6 mg, 1.0 Eq, 0.04 mmol), 1-(4-methoxy-2-methylphenyl)sulfonylpyrazol-3-amine (10 mg, 1.1 Eq, 0.04 mmol) and N,N-diisopropylethylamine (0.01 mL, 2.0 Eq, 0.07mmol) in DMF (0.15 mL) then stirred at RT for 16 hr. The solution was purified by semi-prep HPLC (eluting with 5-70% MeCN in 0.05% NH_4OH (aq)) to obtain 7.2 mg (48 yield) of **7d**. ^1H NMR (400 MHz, $CDCl_3$) δ 9.27 (s, 1H), 8.85 (s, 1H), 8.05 – 7.89 (m, 3H), 7.41 (d, *J* = 8.1 Hz, 1H), 6.92 (d, *J* = 2.8 Hz, 1H), 6.82 (dd, *J* = 8.9, 2.6 Hz, 1H), 6.74 (d, *J* = 2.6 Hz, 1H), 3.92 (s, 2H), 3.86 (s, 3H), 2.79 (s, 3H), 2.50 (s, 3H). LCMS *m/z* = 401.2 $[M+H]^+$.

1-(6-Methylpyridin-3-yl)-3-(1-tosyl-1H-pyrazol-3-yl)urea (8e):

1-Tosyl-1H-pyrazol-3-amine: Synthesized in a similar manner as **7d** starting from 4-methylbenzenesulfonyl chloride to obtain 98 mg (73% yield) of 1-tosyl-1H-pyrazol-3-amine. LCMS *m/z* = 268.2 $[M+H]^+$.

1-(6-Methylpyridin-3-yl)-3-(1-tosyl-1H-pyrazol-3-yl)urea ***(8e):*** 4-nitrophenyl chloroformate (17 mg, 2.0 Eq, 0.08 mmol) was added to 1-(4-methylphenyl)sulfonylpyrazol-3-amine (10 mg, 1.0 Eq, 0.04 mmol) in pyridine (0.25 mL) at RT then the solution was stirred at RT for 30 min. 5-Amino-2-methylpyridine (9.1 mg, 2.0 Eq, 0.08 mmol) was added and the solution was stirred at RT overnight. The solution was concentrated and the residue purified by semi-prep HPLC (eluting with 5-95% MeCN in 0.05% NH4OH (aq) to obtain 1.4 mg (9% yield) of **8e**. LCMS *m/z* = 372.2 $[M+H]^+$.

Spectral Data for Analogs

5

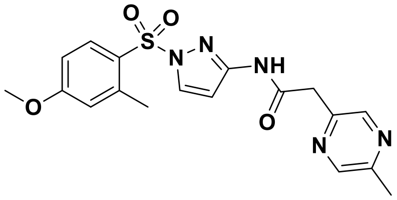

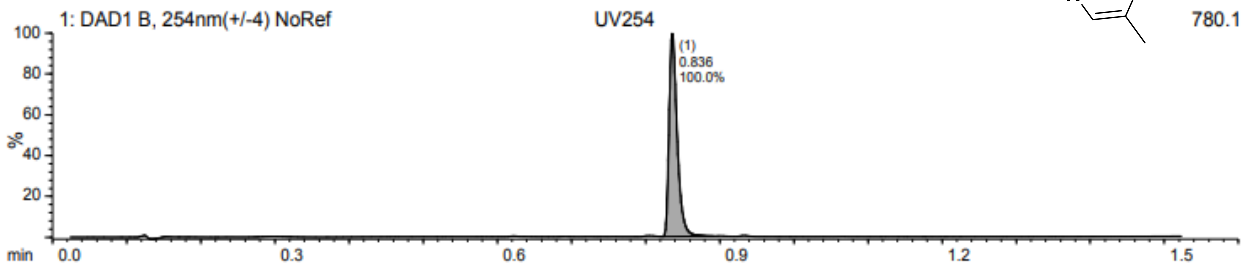

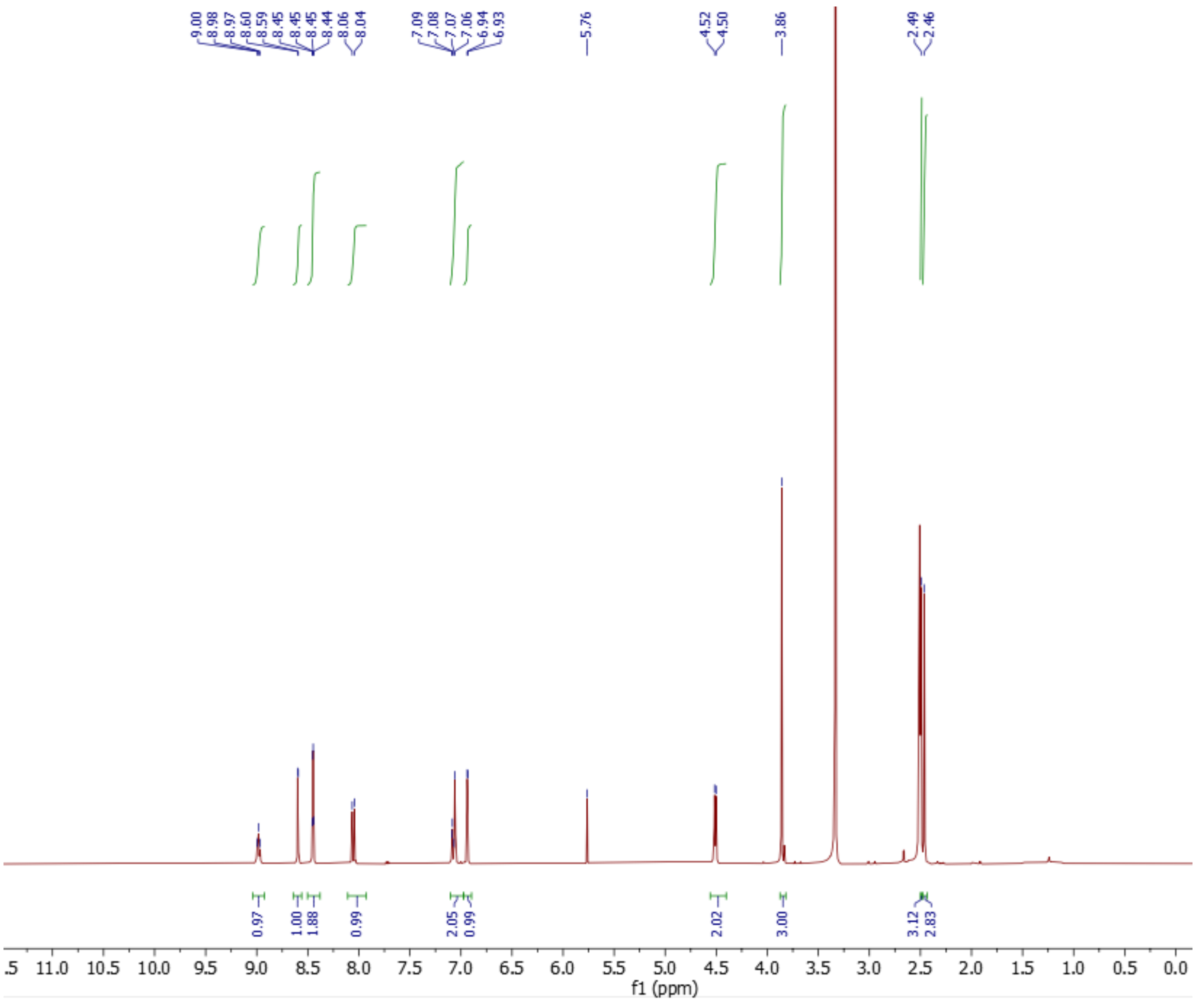

6a

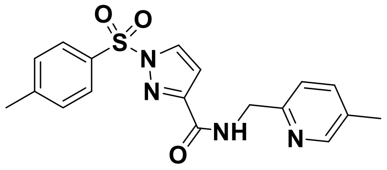

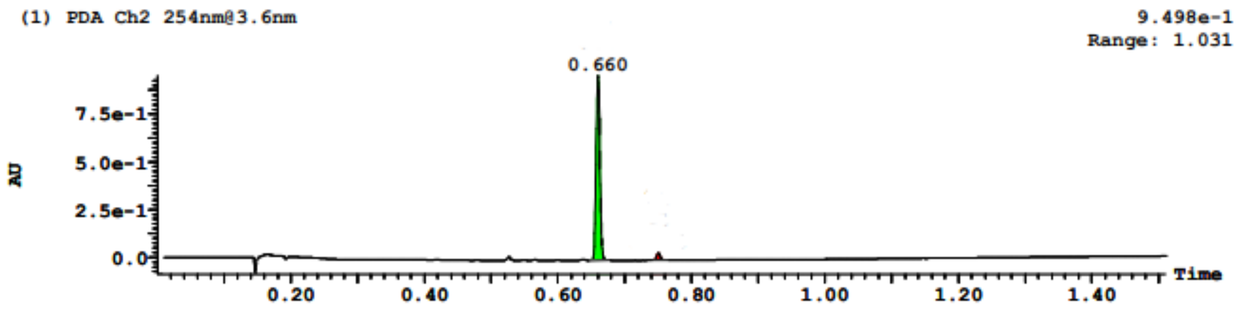

(1) PDA Ch2 254nm@3.6nm
9.498e-1
Range: 1.031
0.660
7.5e-1
5.0e-1
2.5e-1
0.0
AU
0.20
0.40
0.60
0.80
1.00
1.20
1.40
Time

6b

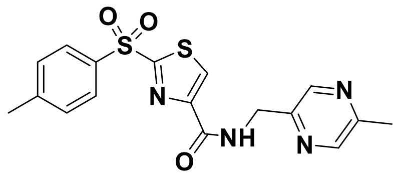
O
O
S
S
N
N
N
N
NH
O

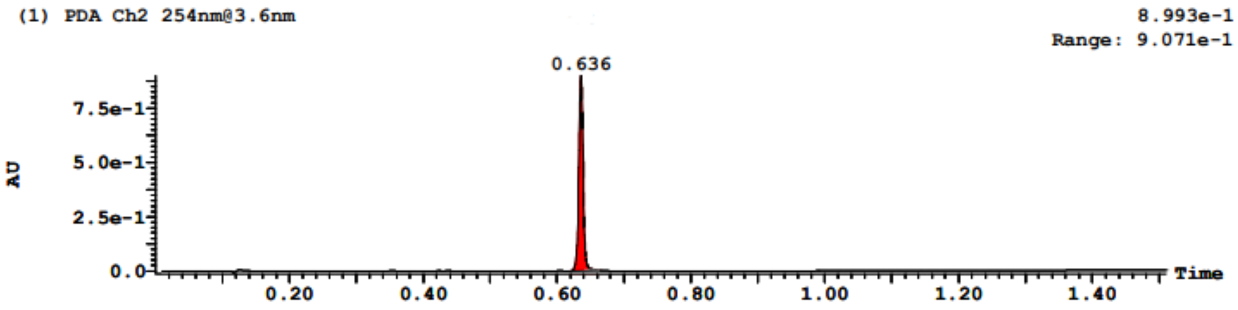
(1) PDA Ch2 254nm@3.6nm
8.993e-1
Range: 9.071e-1
0.636
AU
7.5e-1
5.0e-1
2.5e-1
0.0
Time
0.20
0.40
0.60
0.80
1.00
1.20
1.40

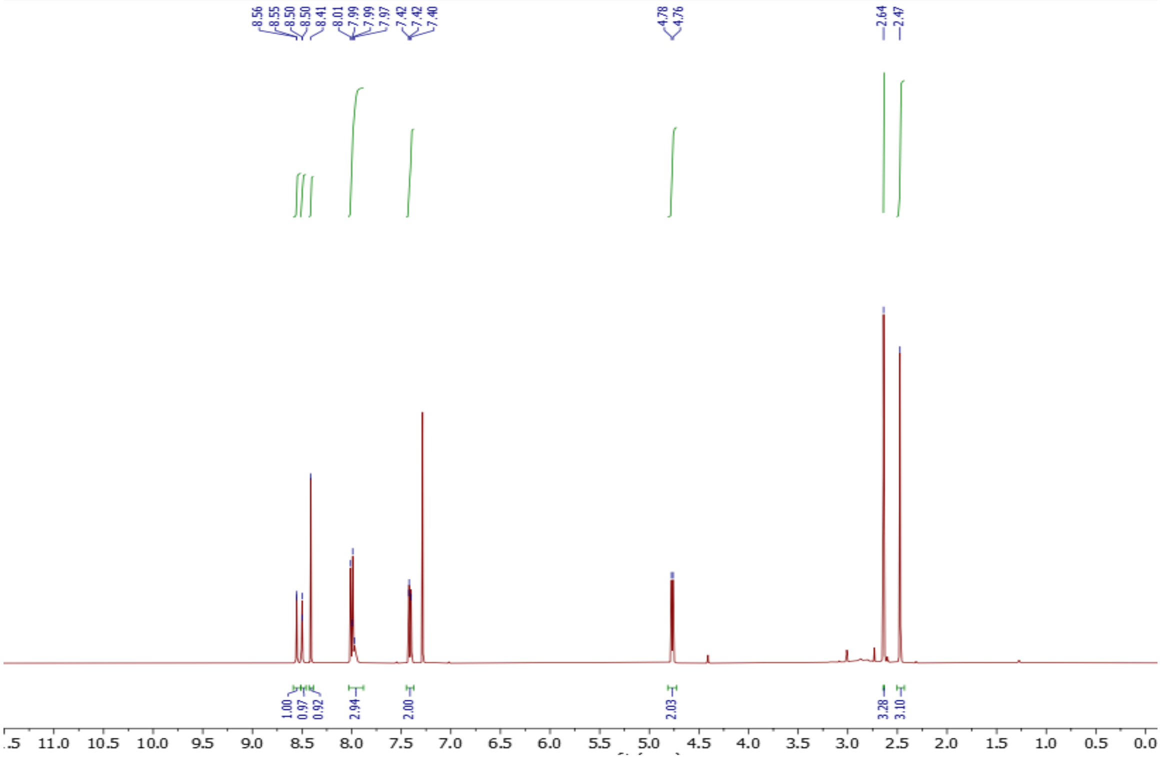
8.56
8.55
8.50
8.50
8.41
8.01
7.99
7.99
7.97
7.42
7.42
7.40
4.78
4.76
2.64
2.47
1.00
0.97
0.92
2.94
2.00
2.03
3.28
3.10
.5
11.0
10.5
10.0
9.5
9.0
8.5
8.0
7.5
7.0
6.5
6.0
5.5
5.0
4.5
4.0
3.5
3.0
2.5
2.0
1.5
1.0
0.5
0.0

6d

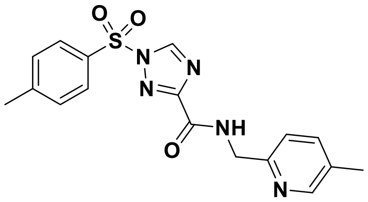
O
O
S
N
N
N
NH
O
N

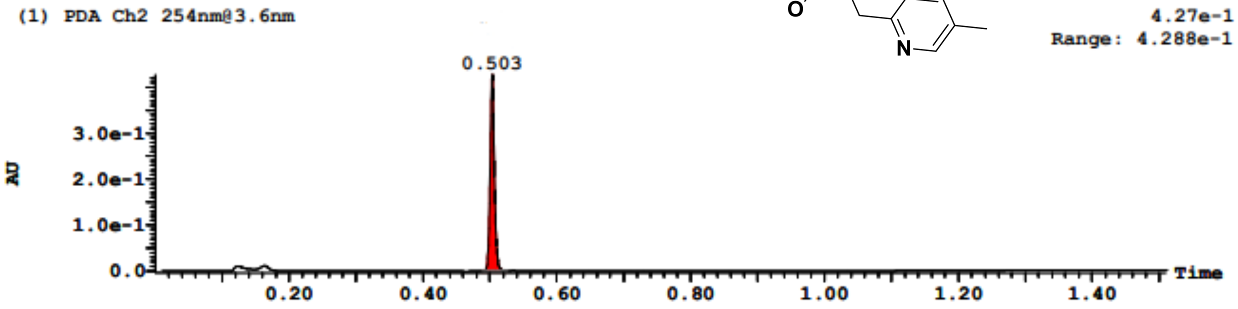
(1) PDA Ch2 254nm@3.6nm
4.27e-1
Range: 4.288e-1
0.503
AU
3.0e-1
2.0e-1
1.0e-1
0.0
0.20
0.40
0.60
0.80
1.00
1.20
1.40
Time

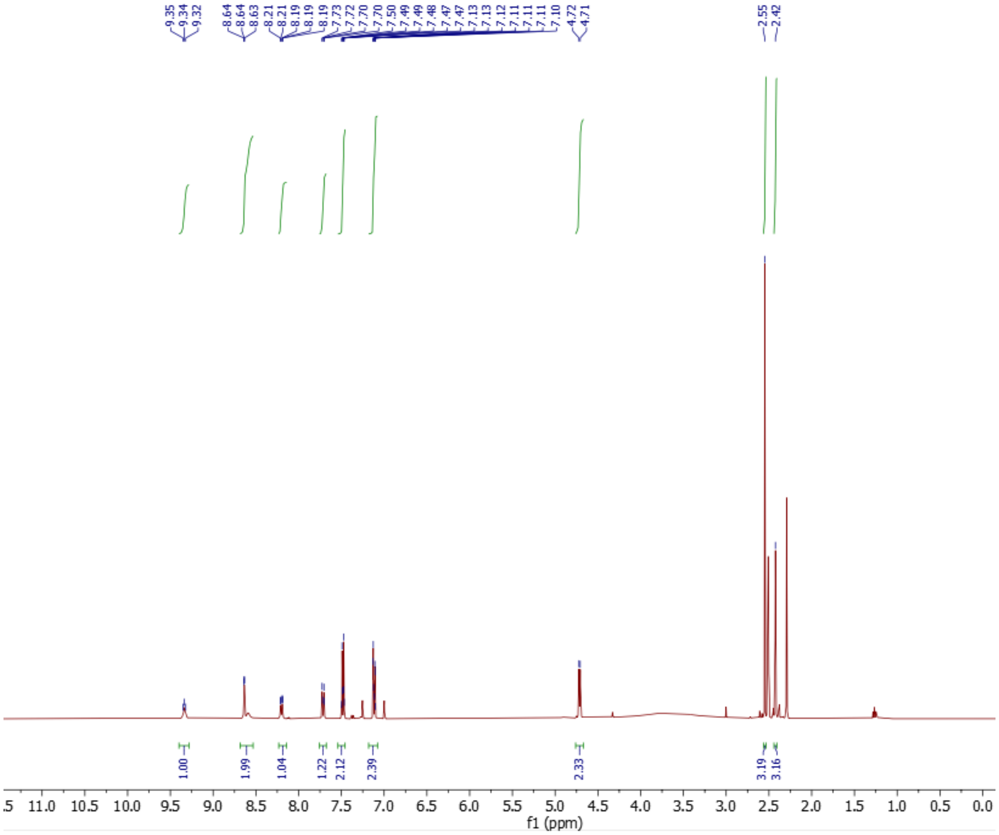
9.35 9.34 9.32 8.64 8.64 8.63 8.21 8.21 8.19 8.19 8.19 7.73 7.72 7.70 7.70 7.50 7.49 7.48 7.47 7.47 7.13 7.13 7.12 7.11 7.11 7.11 7.10 4.72 4.71 2.55 2.42
1.00 1.99 1.04 1.22 2.12 2.39 2.33 3.19 3.16
.5 11.0 10.5 10.0 9.5 9.0 8.5 8.0 7.5 7.0 6.5 6.0 5.5 5.0 4.5 4.0 3.5 3.0 2.5 2.0 1.5 1.0 0.5 0.0
f1 (ppm)

6f

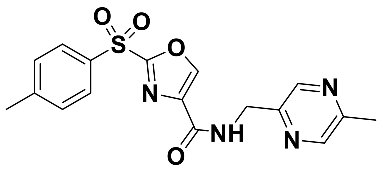

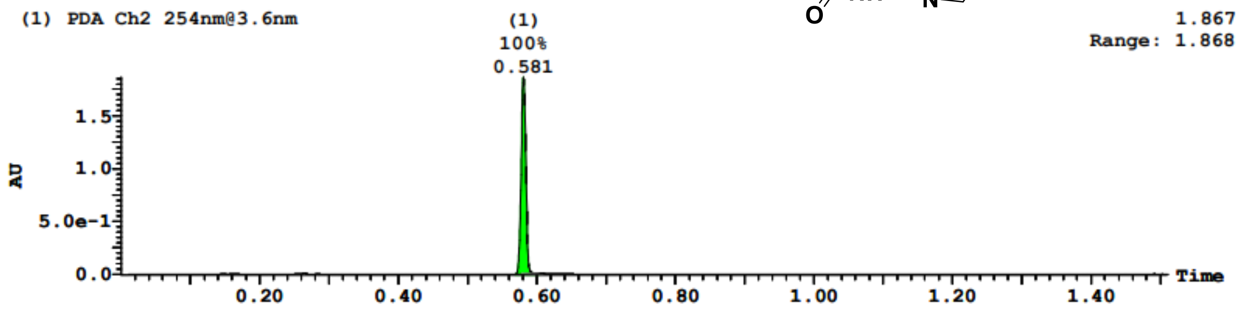
(1) PDA Ch2 254nm@3.6nm
(1)
100%
0.581
1.867
Range: 1.868
AU
1.5
1.0
5.0e-1
0.0
0.20
0.40
0.60
0.80
1.00
1.20
1.40
Time

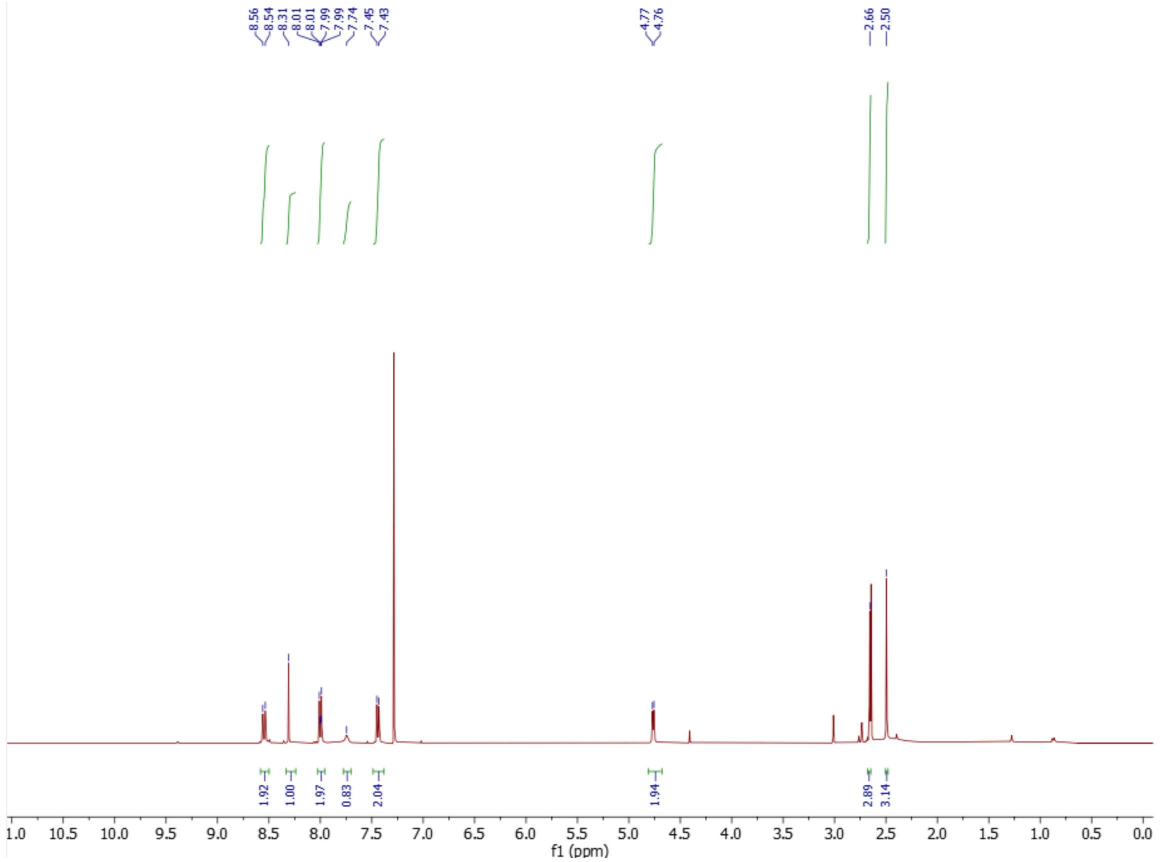
8.56
8.54
8.31
8.01
7.99
7.99
7.74
7.45
7.43
4.77
4.76
2.66
2.50
1.92
1.00
1.97
0.83
2.04
1.94
2.89
3.14
f1 (ppm)

6h

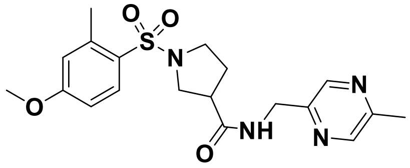

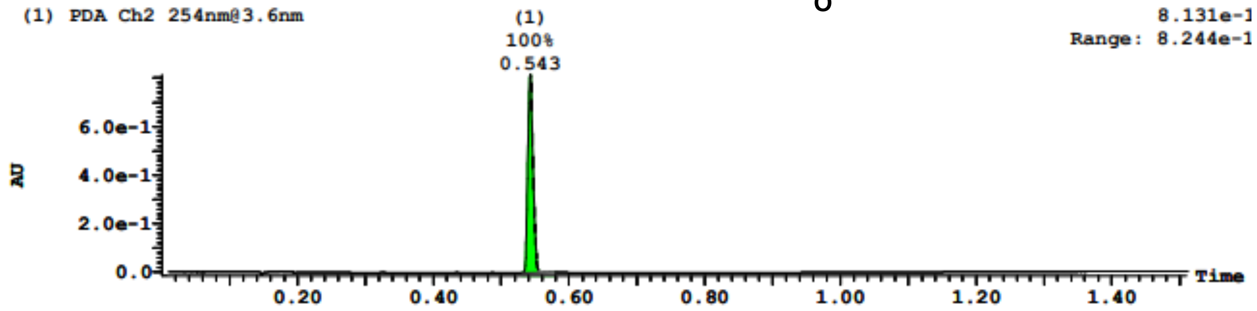
(1) PDA Ch2 254nm@3.6nm
(1)
100%
0.543
8.131e-1
Range: 8.244e-1
AU
Time

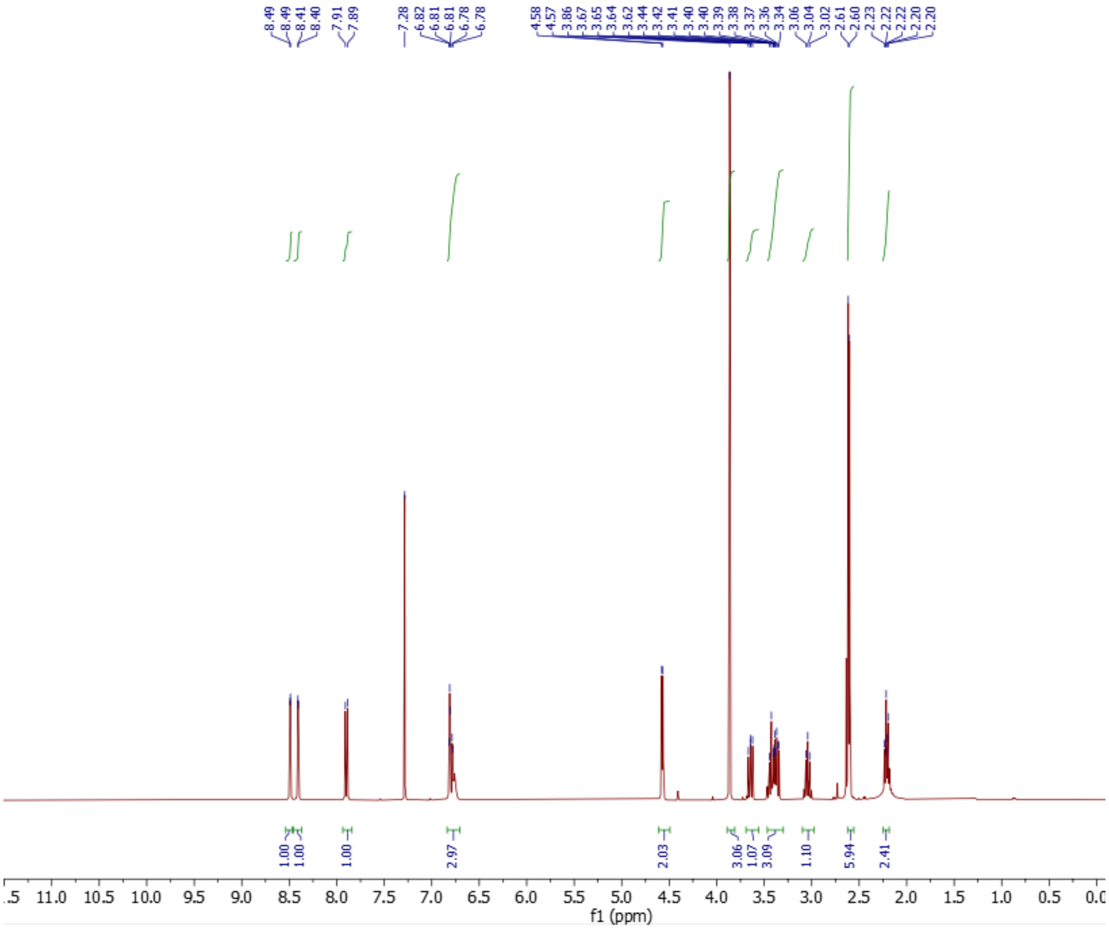
f1 (ppm)

(6i)

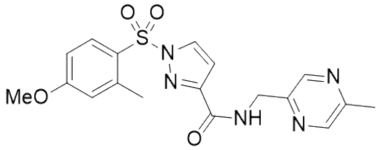

MeO
O
O
S
N
N
O
NH
N
N

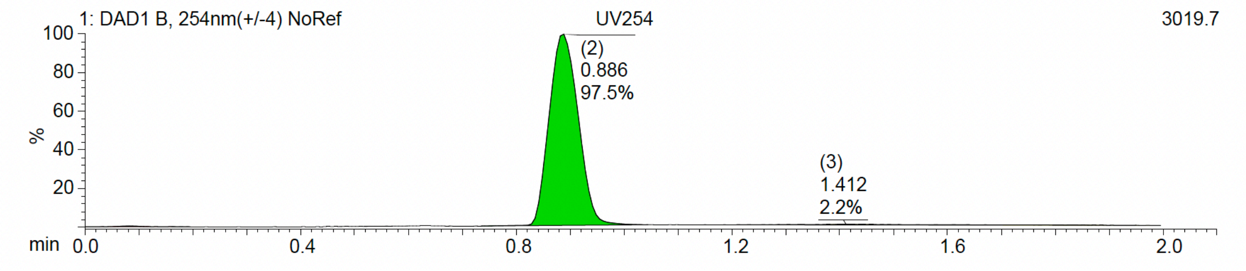

1: DAD1 B, 254nm(+/-4) NoRef
UV254
3019.7
(2)
0.886
97.5%
(3)
1.412
2.2%
%
min

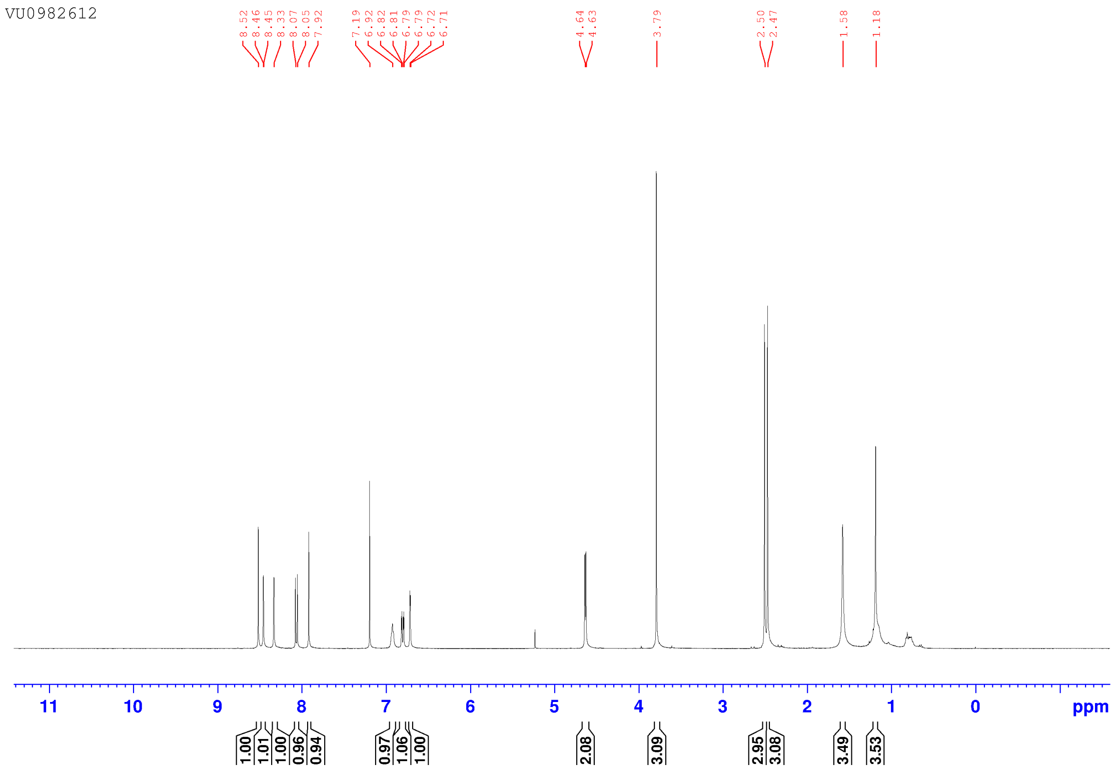

VU0982612
8.52
8.46
8.45
8.33
8.07
8.05
7.92
7.19
6.92
6.82
6.81
6.79
6.79
6.72
6.71
4.64
4.63
3.79
2.50
2.47
1.58
1.18
ppm
1.00
1.01
1.00
0.96
0.94
0.97
1.06
1.00
2.08
3.09
2.95
3.08
3.49
3.53

(6j)

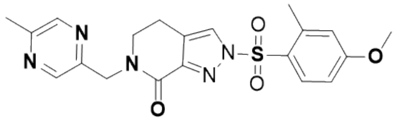
N
N
N
N
N
N
S
O
O
O
O

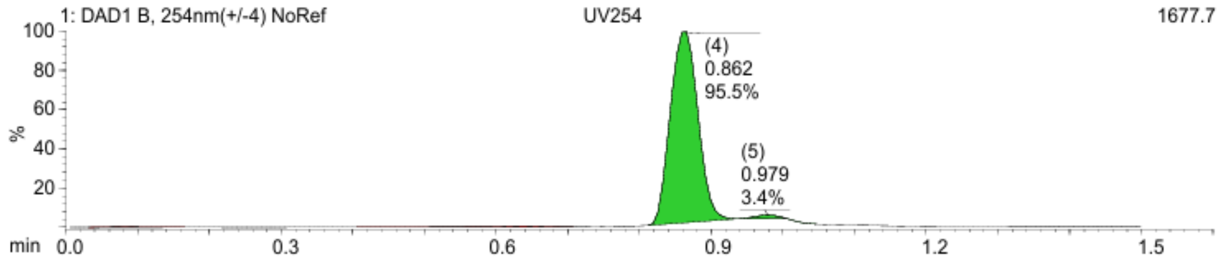
1: DAD1 B, 254nm(+/-4) NoRef
UV254
1677.7
(4)
0.862
95.5%
(5)
0.979
3.4%
%
min

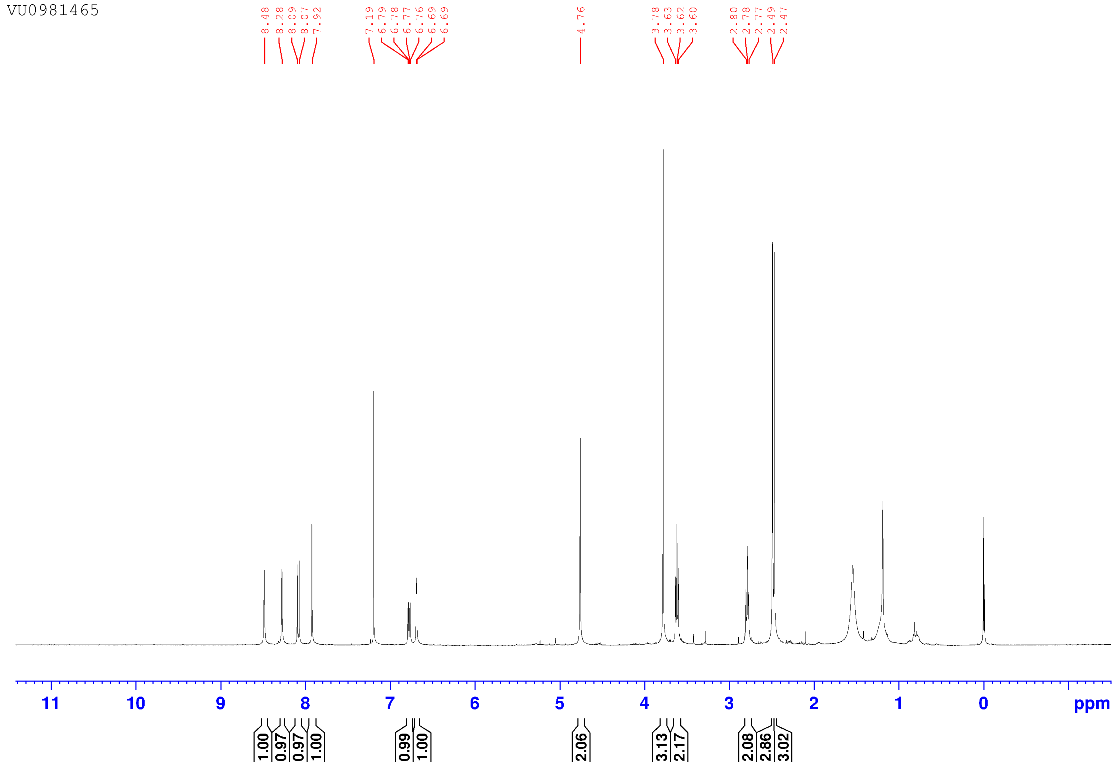
VU0981465
8.48
8.28
8.09
8.07
7.92
7.19
6.79
6.78
6.77
6.76
6.69
6.69
4.76
3.78
3.63
3.62
3.60
2.80
2.78
2.77
2.49
2.47
ppm
1.00
0.97
0.97
1.00
0.99
1.00
2.06
3.13
2.17
2.08
2.86
3.02

(6k)

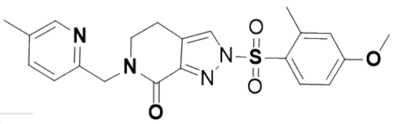

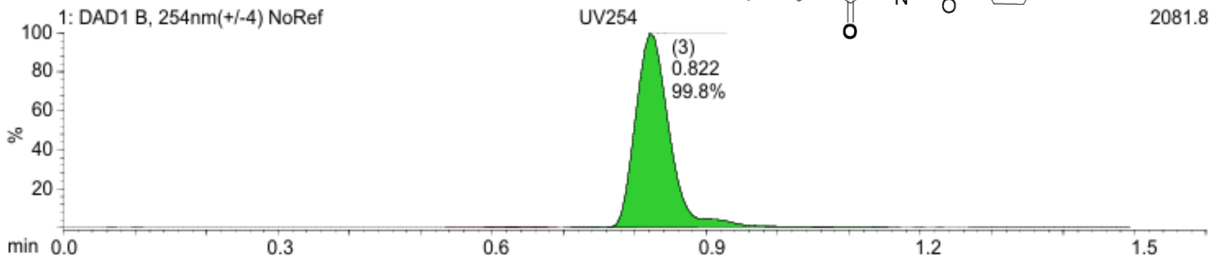

1: DAD1 B, 254nm(+/-4) NoRef
UV254
2081.8
(3)
0.822
99.8%
%
min

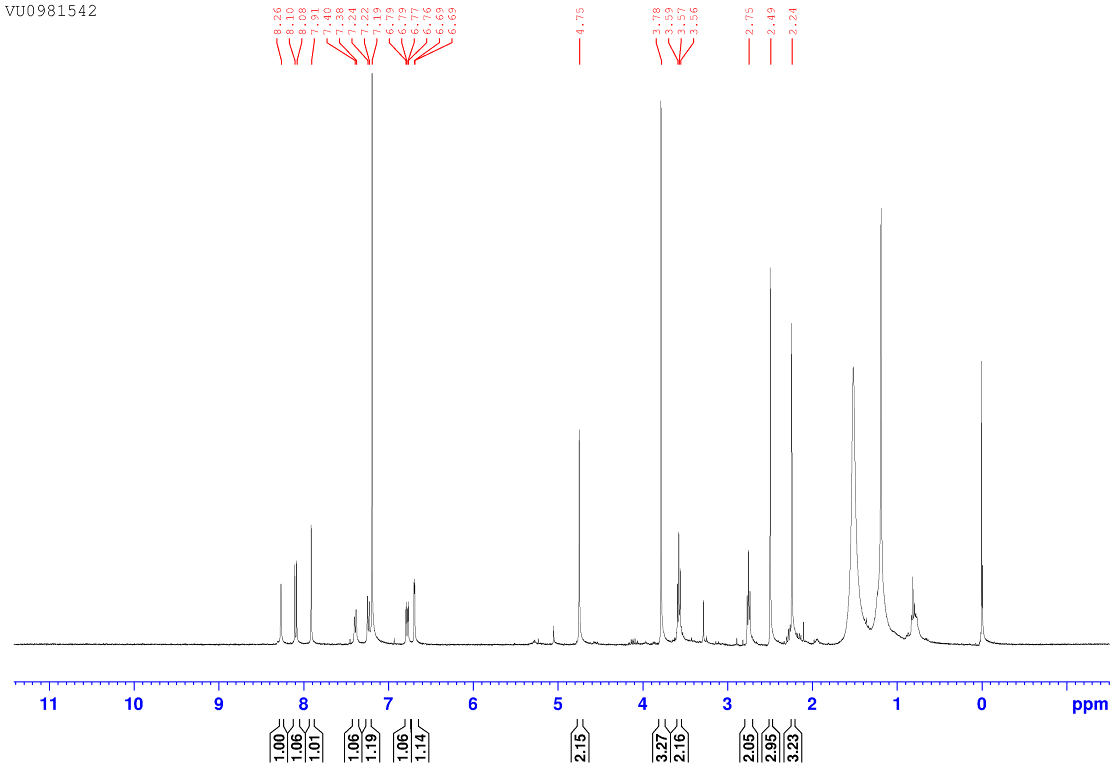

VU0981542
8.26
8.10
8.08
7.91
7.40
7.38
7.24
7.22
7.19
6.79
6.79
6.77
6.76
6.69
6.69
4.75
3.78
3.59
3.57
3.56
2.75
2.49
2.24
ppm
1.00
1.06
1.01
1.06
1.19
1.06
1.14
2.15
3.27
2.16
2.05
2.95
3.23

(6l)

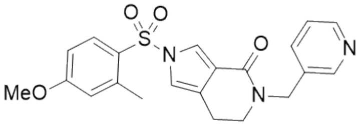

MeO
O
O
S
N
O
N
N

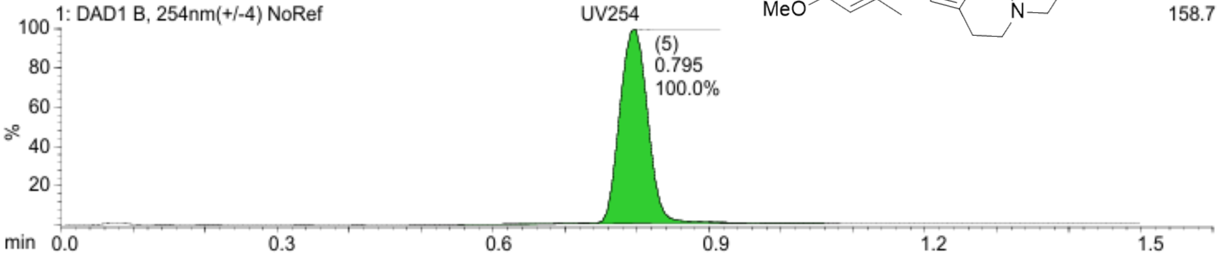

1: DAD1 B, 254nm(+/-4) NoRef
UV254
158.7
(5)
0.795
100.0%
100
80
60
40
20
%
min
0.0
0.3
0.6
0.9
1.2
1.5

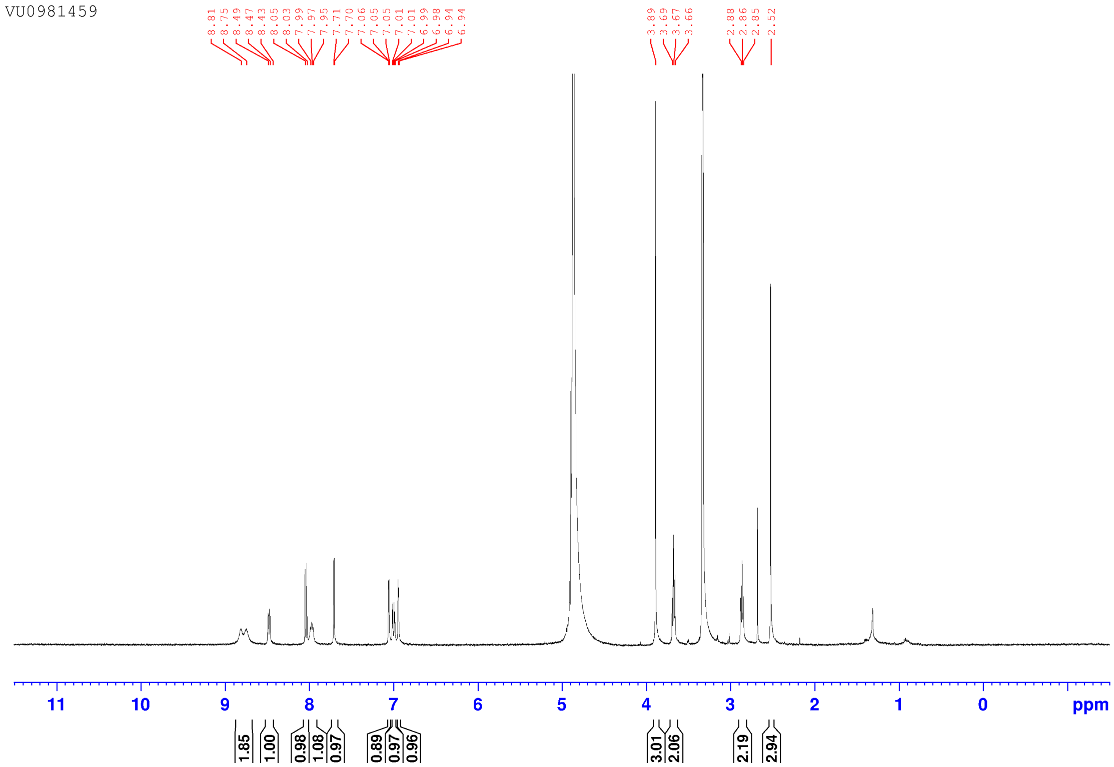

VU0981459
8.81 8.75 8.49 8.47 8.43 8.05 8.03 7.99 7.97 7.95 7.71 7.70 7.06 7.05 7.05 7.01 7.01 6.99 6.98 6.94 6.94
3.89 3.69 3.67 3.66
2.88 2.86 2.85 2.52
11
10
9
8
7
6
5
4
3
2
1
0
ppm
1.85 1.00 0.98 1.08 0.97 0.89 0.97 0.96 3.01 2.06 2.19 2.94

7d

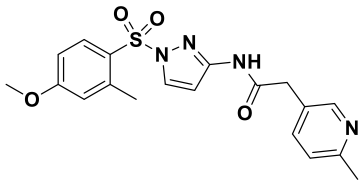
O
O
S
N
N
NH
O
O
N

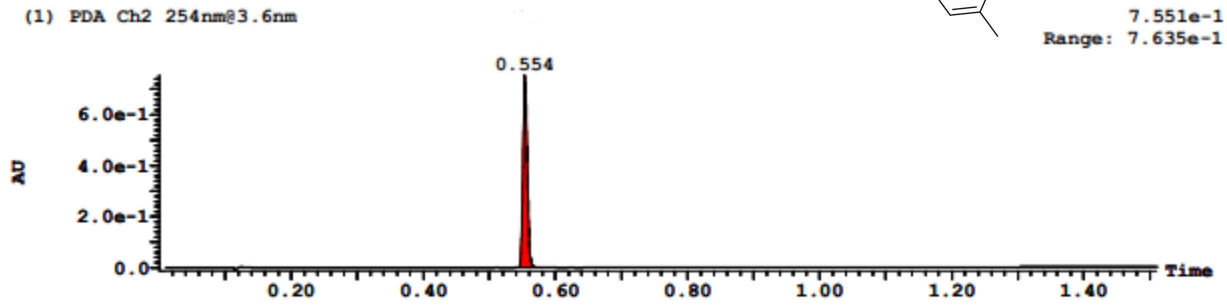
(1) PDA Ch2 254nm@3.6nm
7.551e-1
Range: 7.635e-1
0.554
AU
6.0e-1
4.0e-1
2.0e-1
0.0
0.20
0.40
0.60
0.80
1.00
1.20
1.40
Time

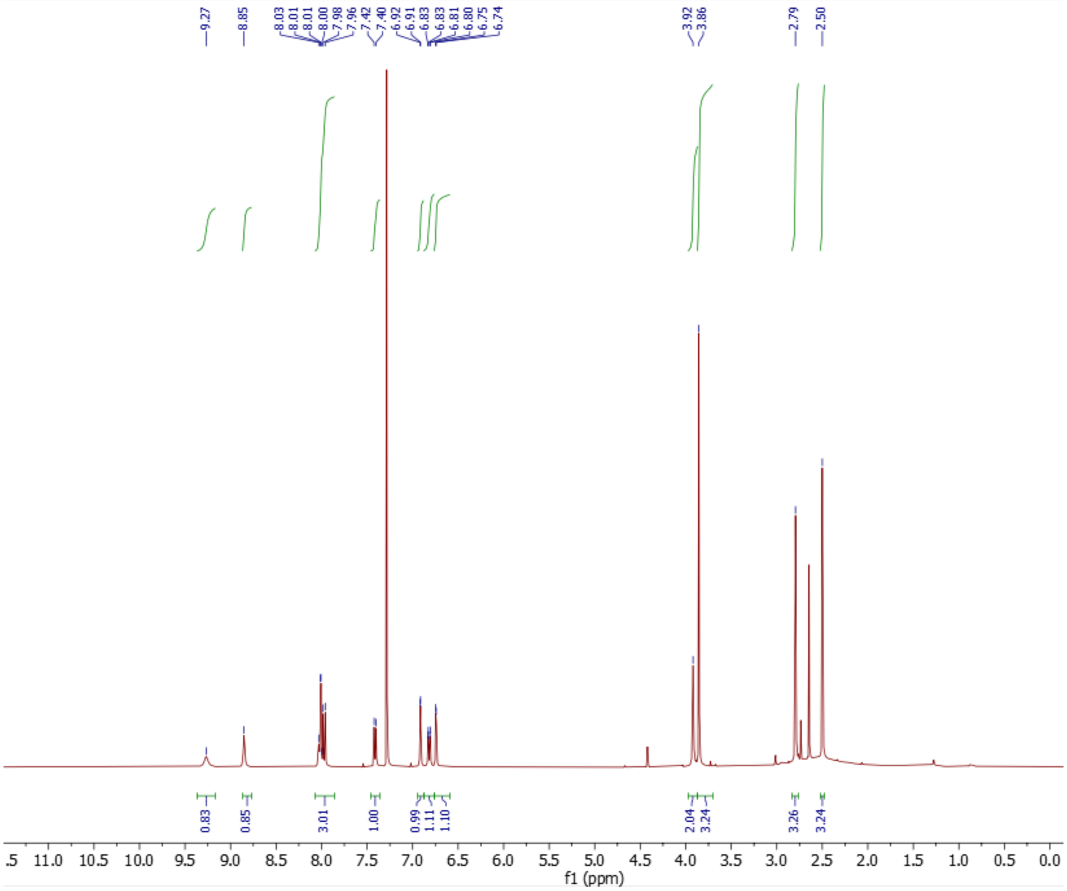
9.27
8.85
8.03
8.01
8.01
8.00
7.98
7.96
7.42
7.40
6.92
6.91
6.83
6.83
6.81
6.80
6.75
6.74
3.92
3.86
2.79
2.50
0.83
0.85
3.01
1.00
0.99
1.11
1.10
2.04
3.24
3.26
3.24
f1 (ppm)

7f

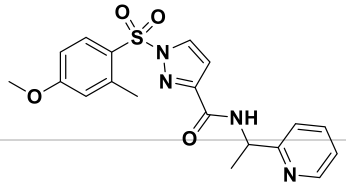
O
O
S
N
N
O
NH
O
N

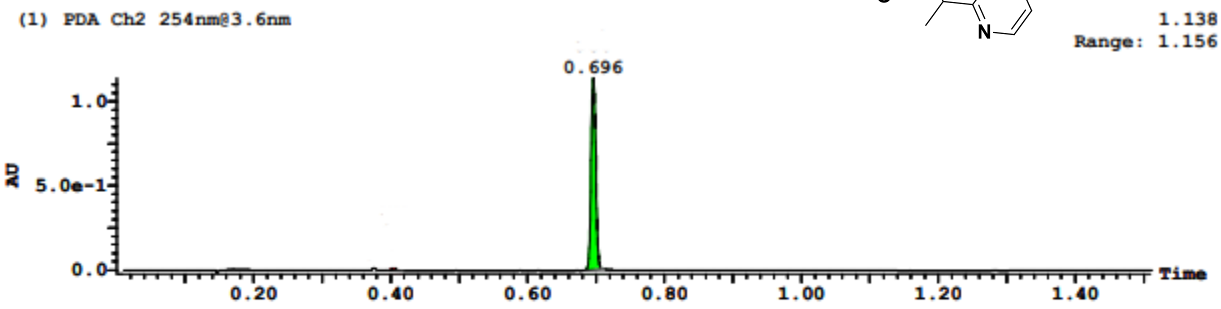
(1) PDA Ch2 254nm@3.6nm
1.138
Range: 1.156
0.696
AU
1.0
5.0e-1
0.0
0.20
0.40
0.60
0.80
1.00
1.20
1.40
Time

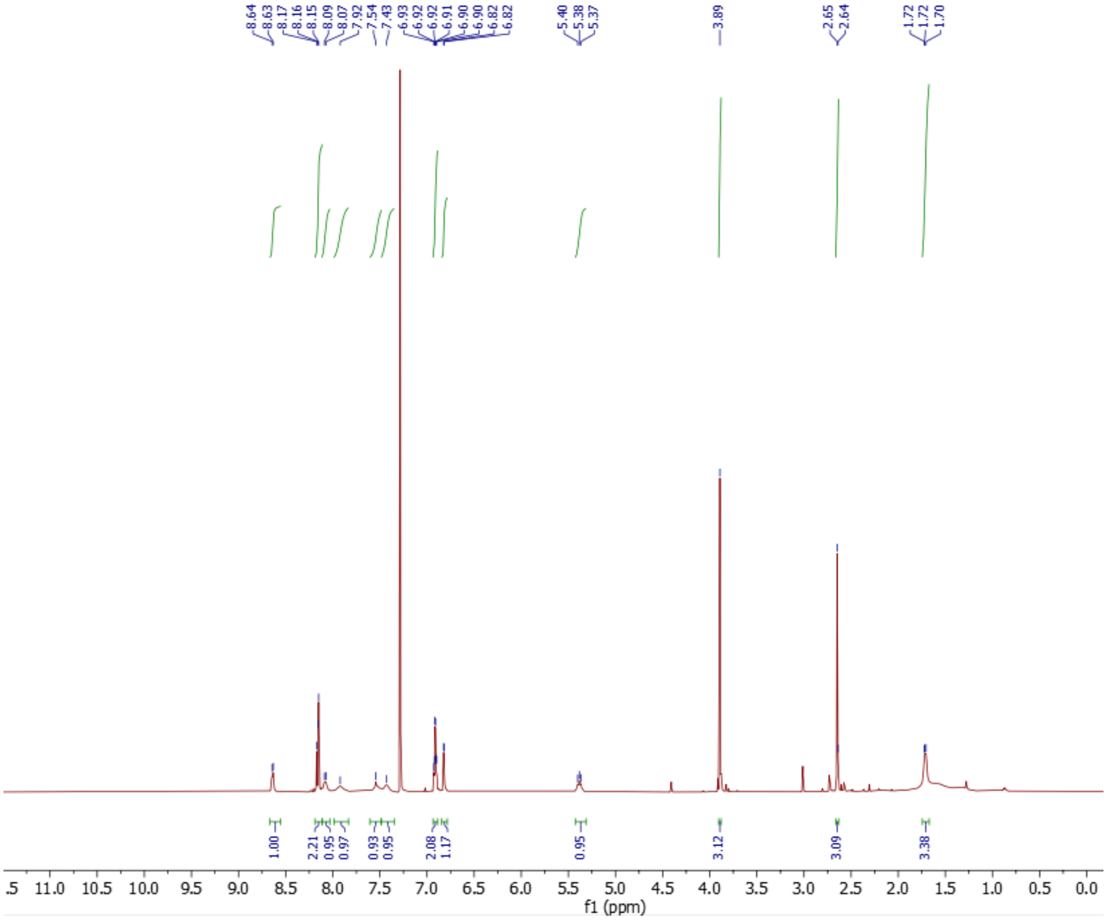
8.64
8.63
8.17
8.16
8.15
8.09
8.07
7.92
7.54
7.43
6.93
6.92
6.92
6.91
6.90
6.90
6.82
6.82
5.40
5.38
5.37
3.89
2.65
2.64
1.72
1.72
1.70
1.00
2.21
0.95
0.97
0.93
0.95
2.08
1.17
0.95
3.12
3.09
3.38
f1 (ppm)

7g

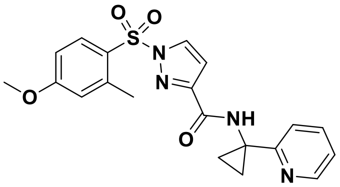

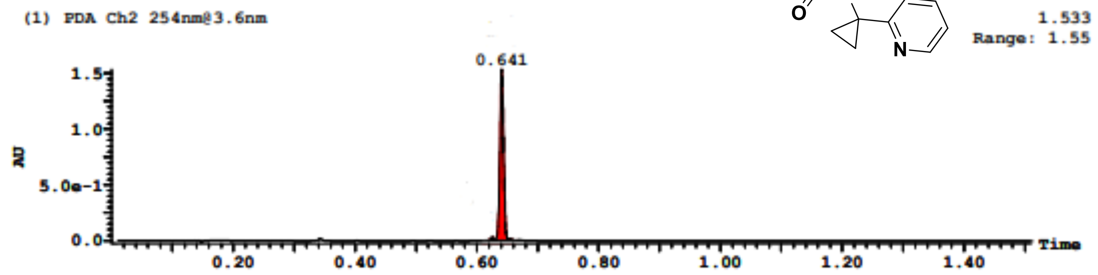

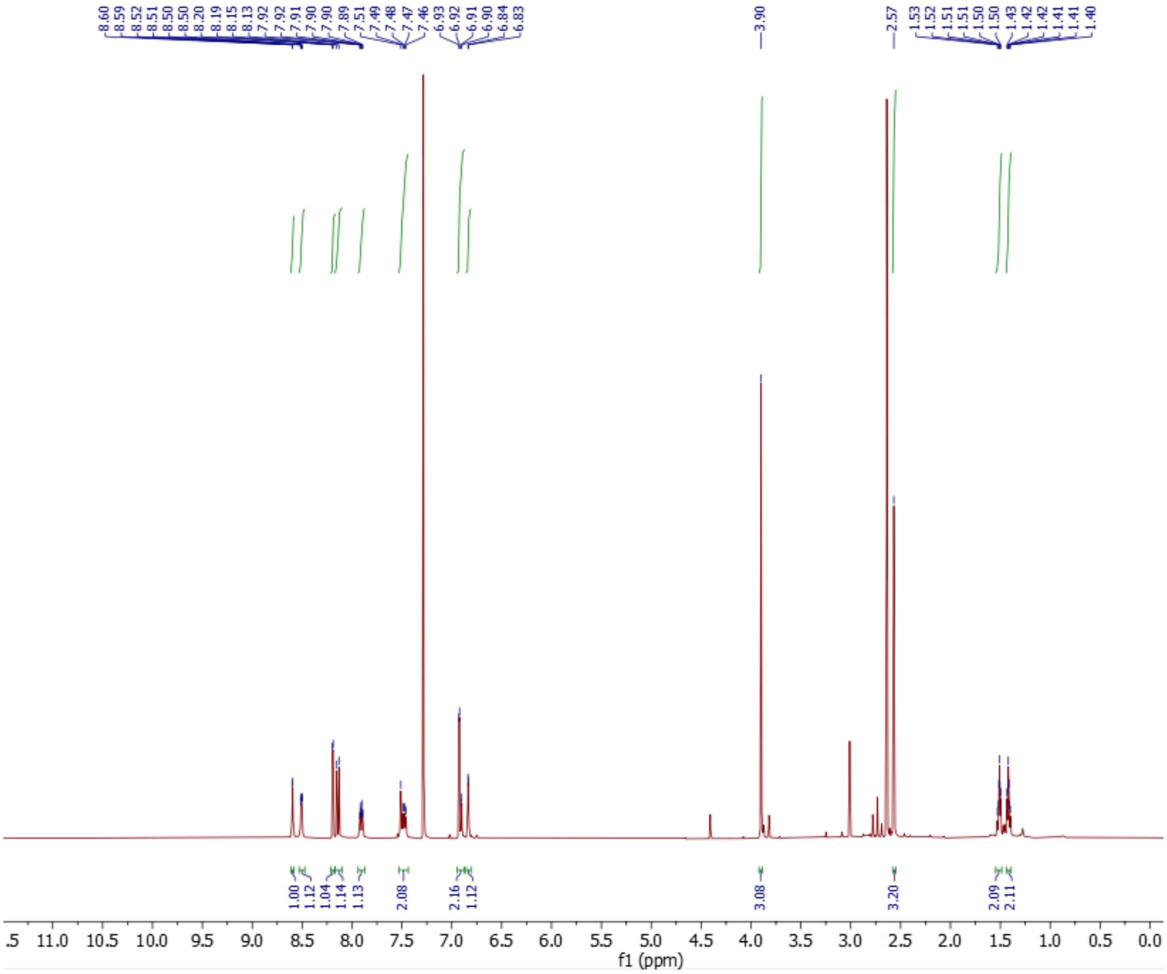

7i

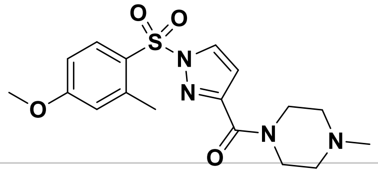

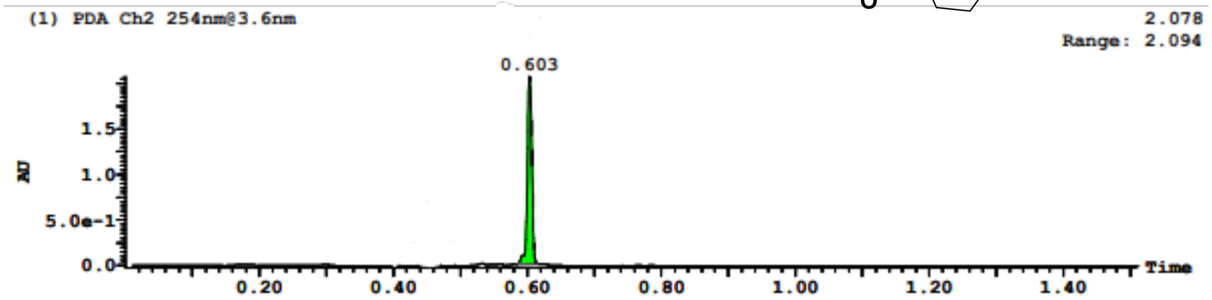

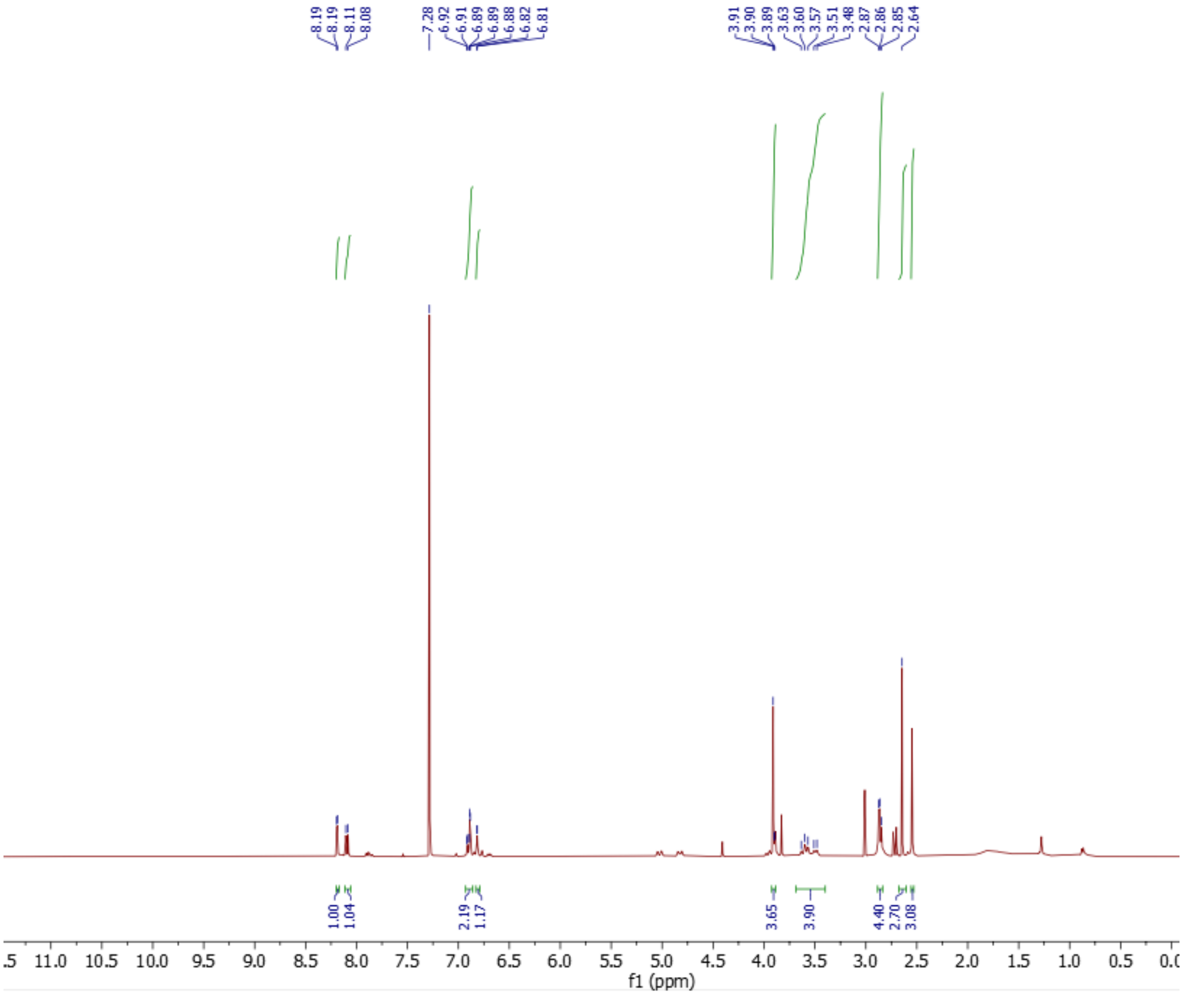

7j

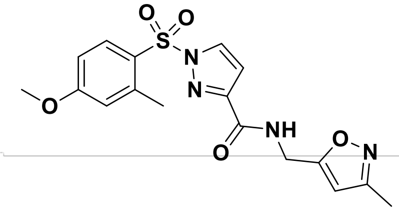

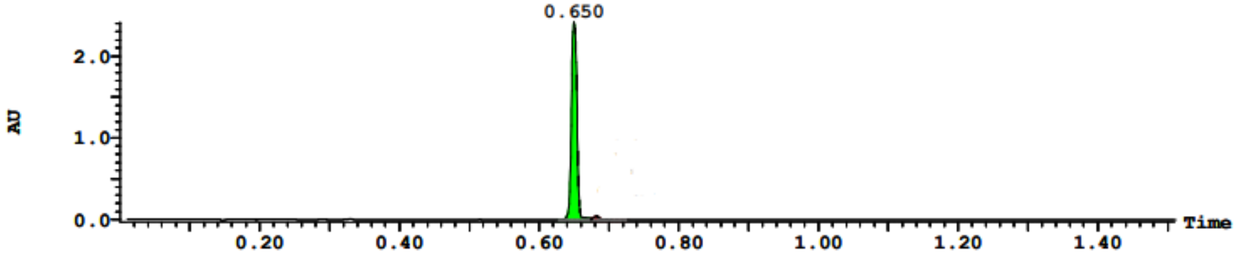

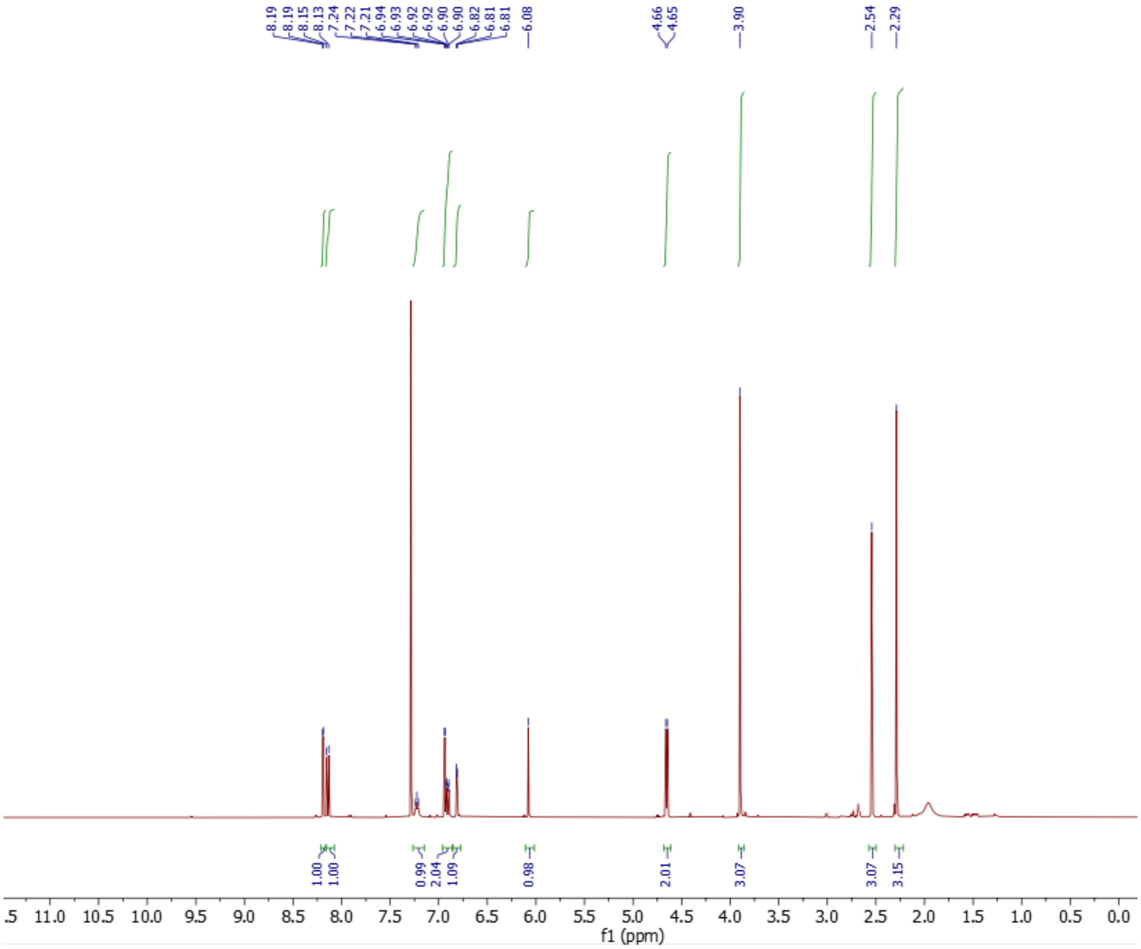

7m

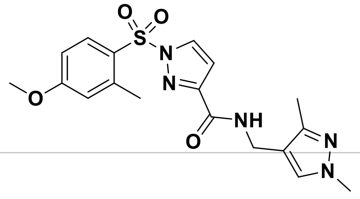

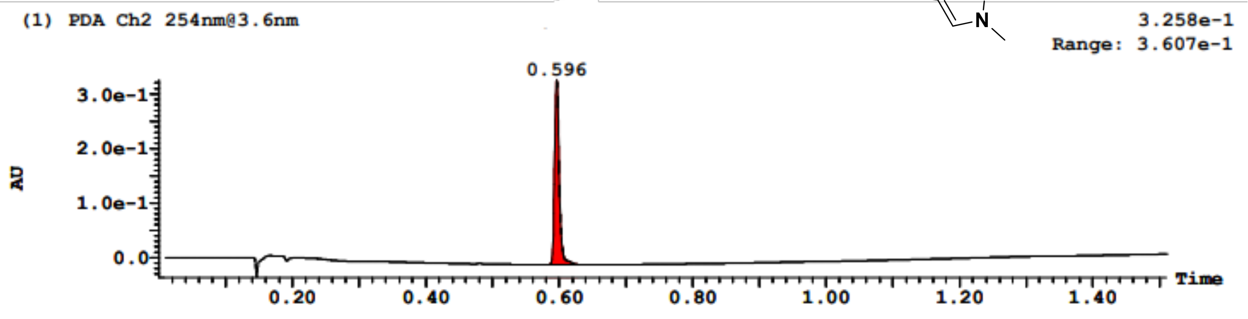

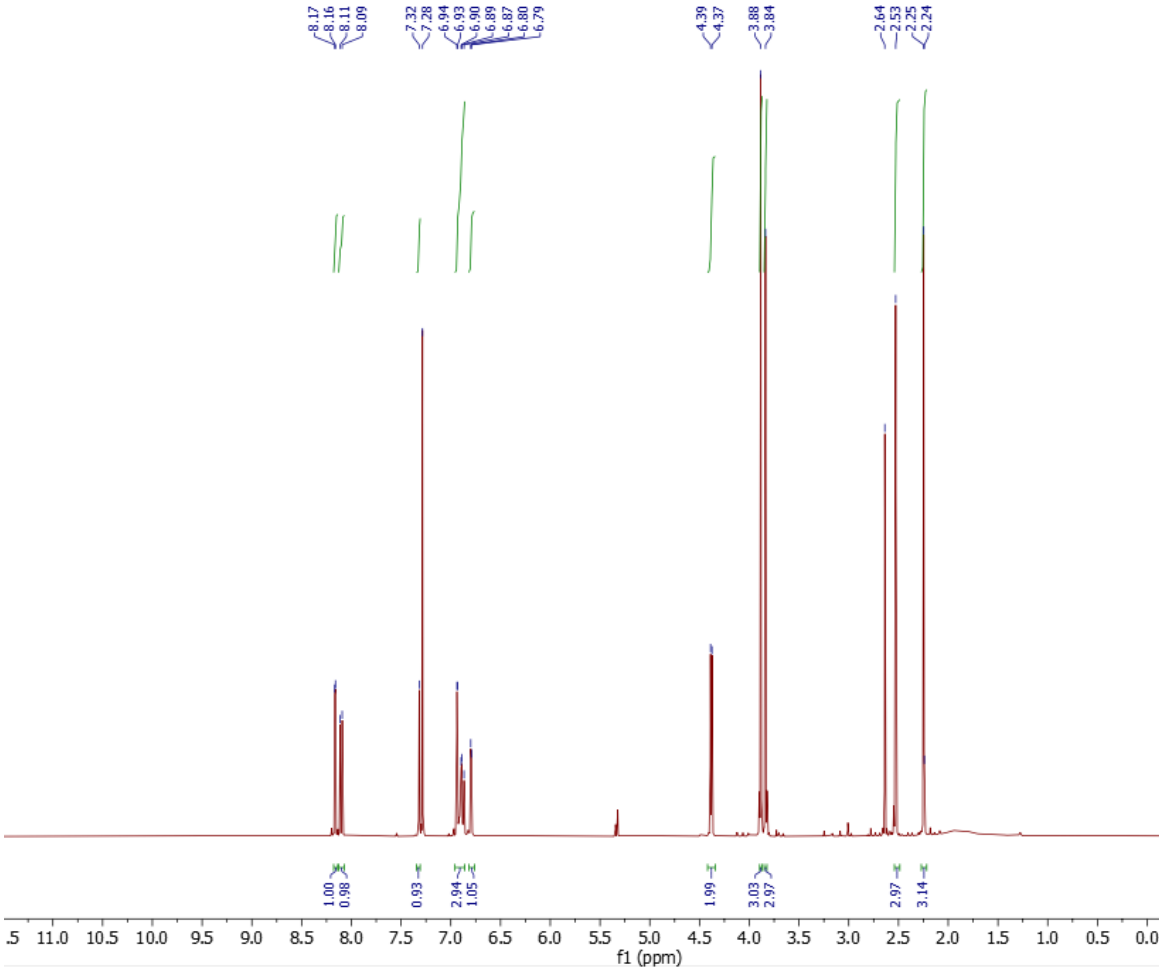

7o

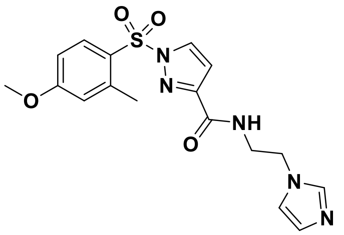

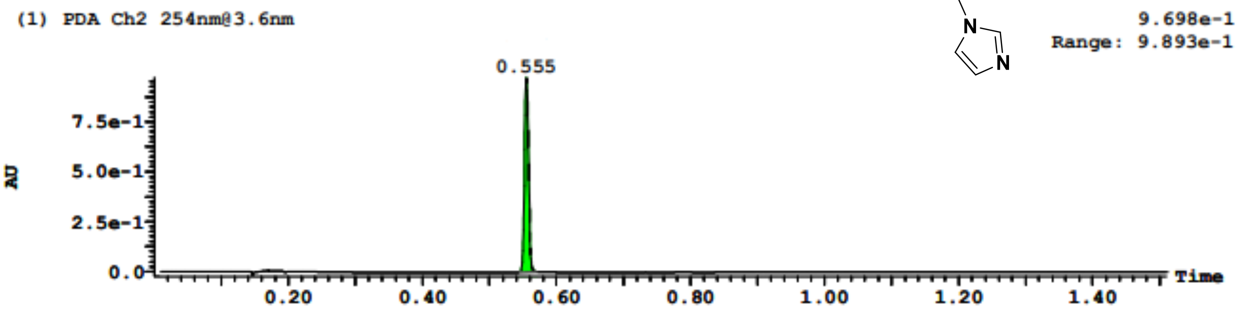

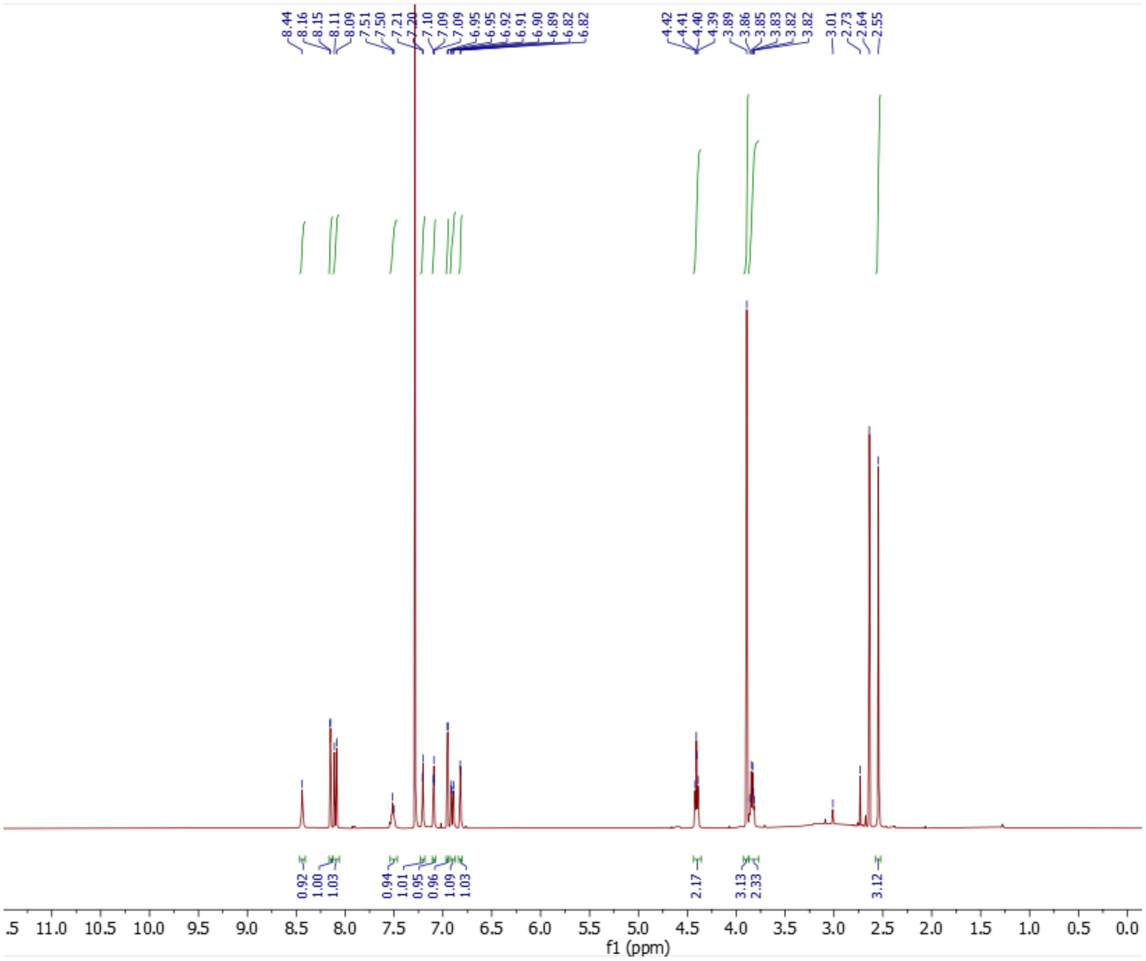

(7p)

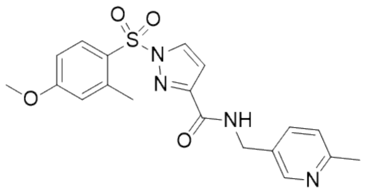
O
O
S
N
N
O
NH
O
N

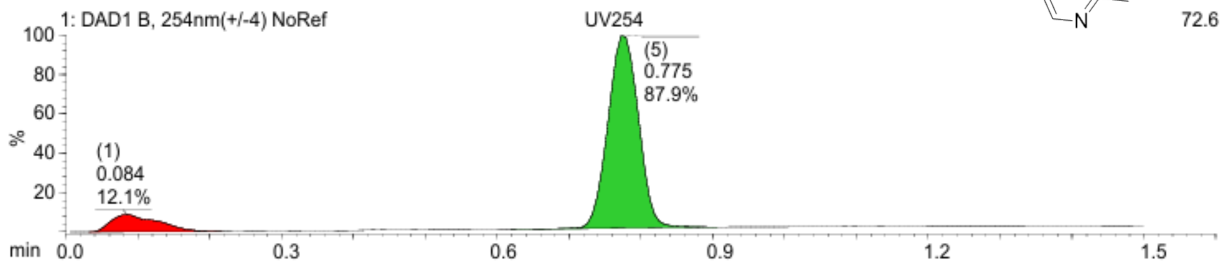
1: DAD1 B, 254nm(+/-4) NoRef
UV254
72.6
(5)
0.775
87.9%
(1)
0.084
12.1%
%
min
100
80
60
40
20
0.0
0.3
0.6
0.9
1.2
1.5

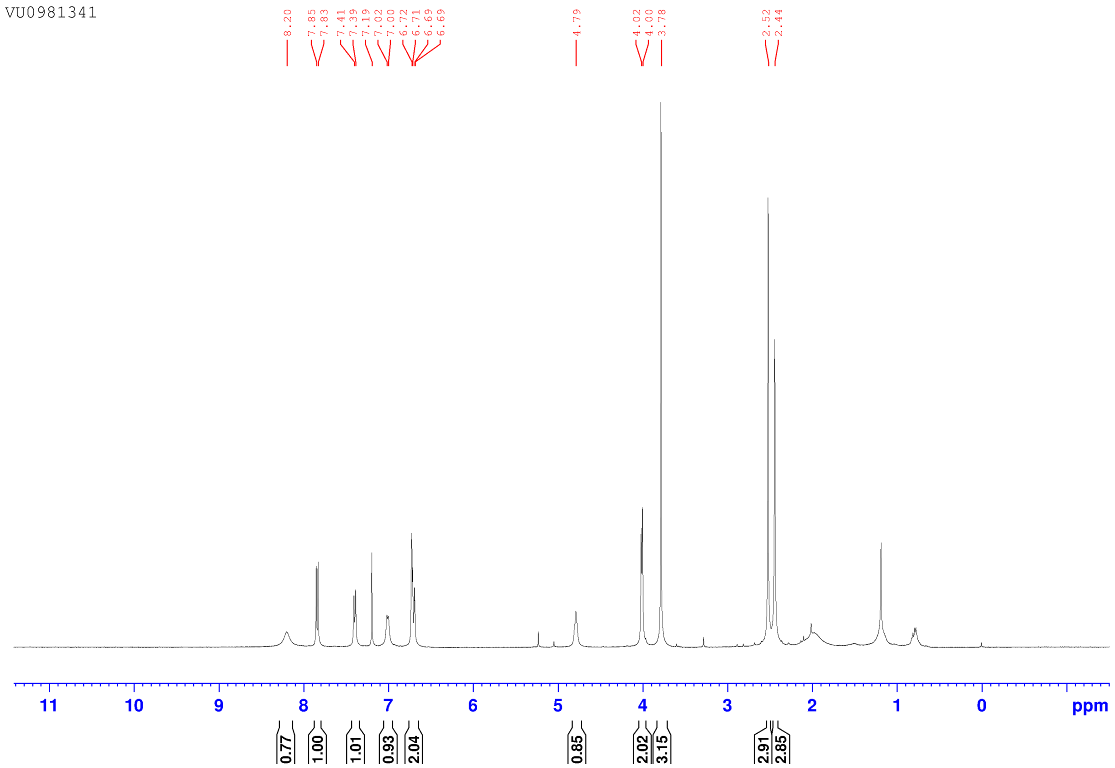
VU0981341
8.20
7.85
7.83
7.41
7.39
7.19
7.02
7.00
6.72
6.71
6.69
6.69
4.79
4.02
4.00
3.78
2.52
2.44
11
10
9
8
7
6
5
4
3
2
1
0
ppm
0.77
1.00
1.01
0.93
2.04
0.85
2.02
3.15
2.91
2.85

(7r)

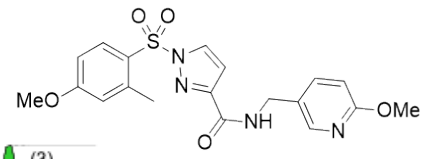

MeO
OMe
NH
N

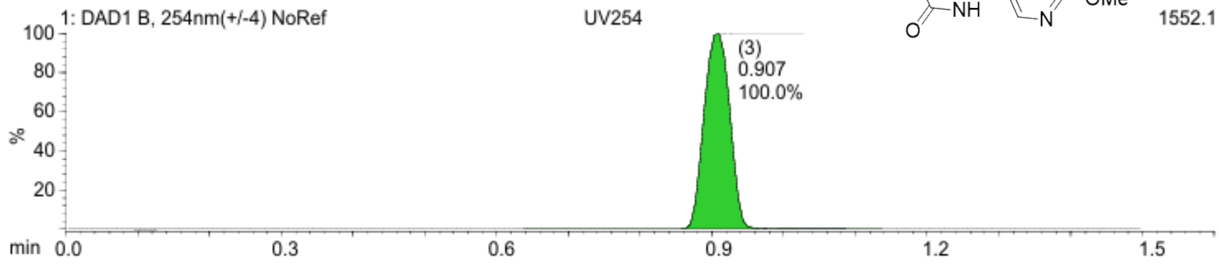

1: DAD1 B, 254nm(+/-4) NoRef
UV254
1552.1
(3)
0.907
100.0%
%
min

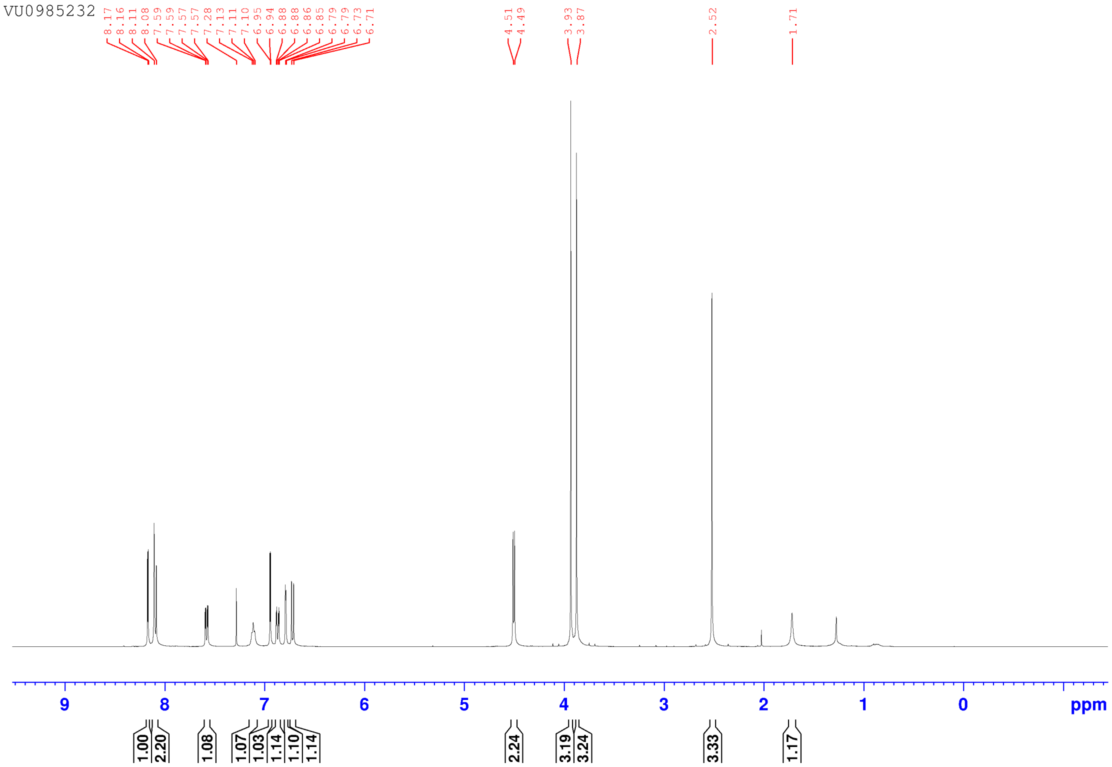

VU0985232
ppm

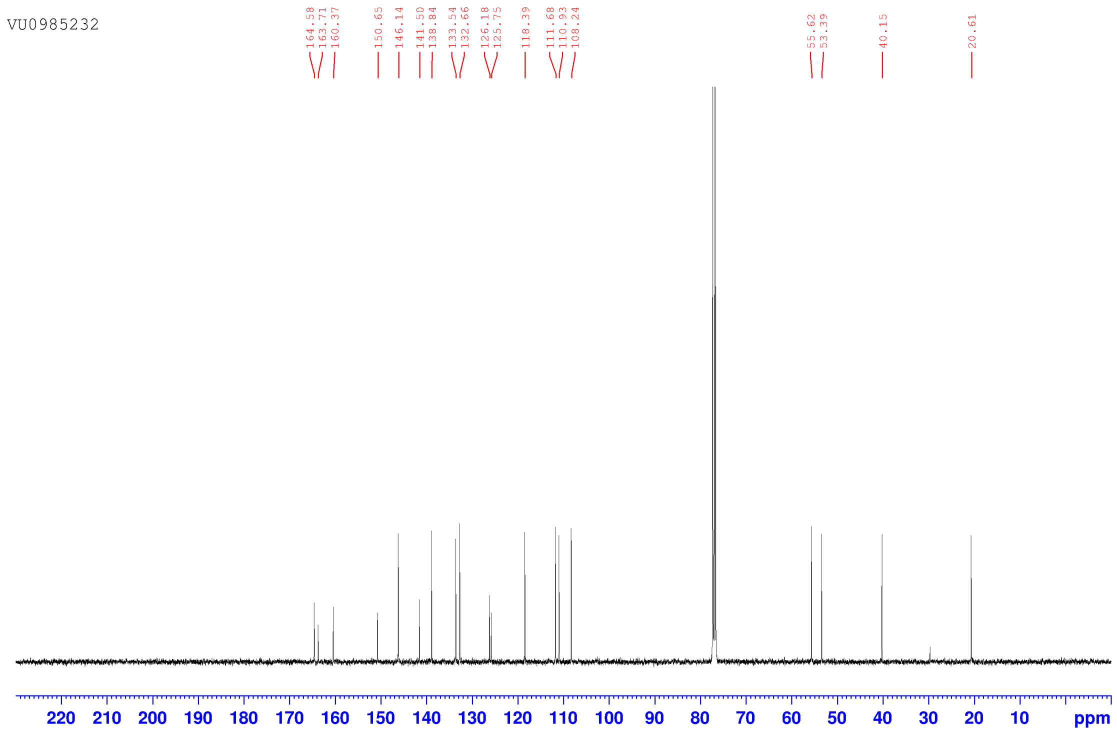

VU0985232
164.58
163.71
160.37
150.65
146.14
141.50
138.84
133.54
132.66
126.18
125.75
118.39
111.68
110.93
108.24
55.62
53.39
40.15
20.61
220 210 200 190 180 170 160 150 140 130 120 110 100 90 80 70 60 50 40 30 20 10 ppm

(7s)

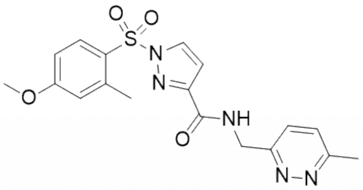

O
O
S
N
N
O
NH
O
N
N

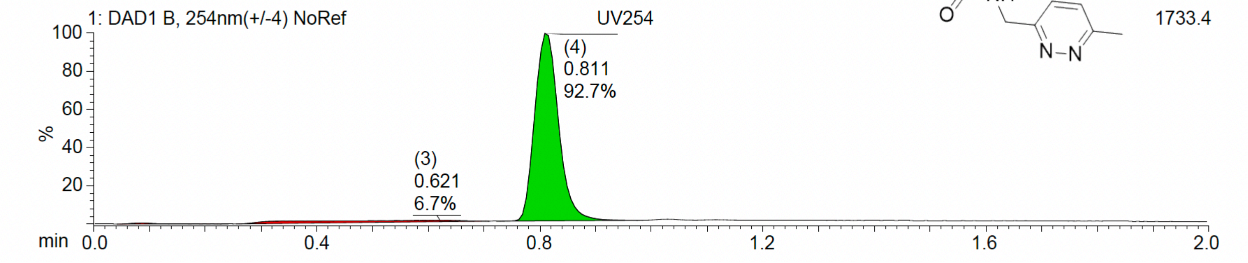

1: DAD1 B, 254nm(+/-4) NoRef
UV254
1733.4
(4)
0.811
92.7%
(3)
0.621
6.7%
%
min

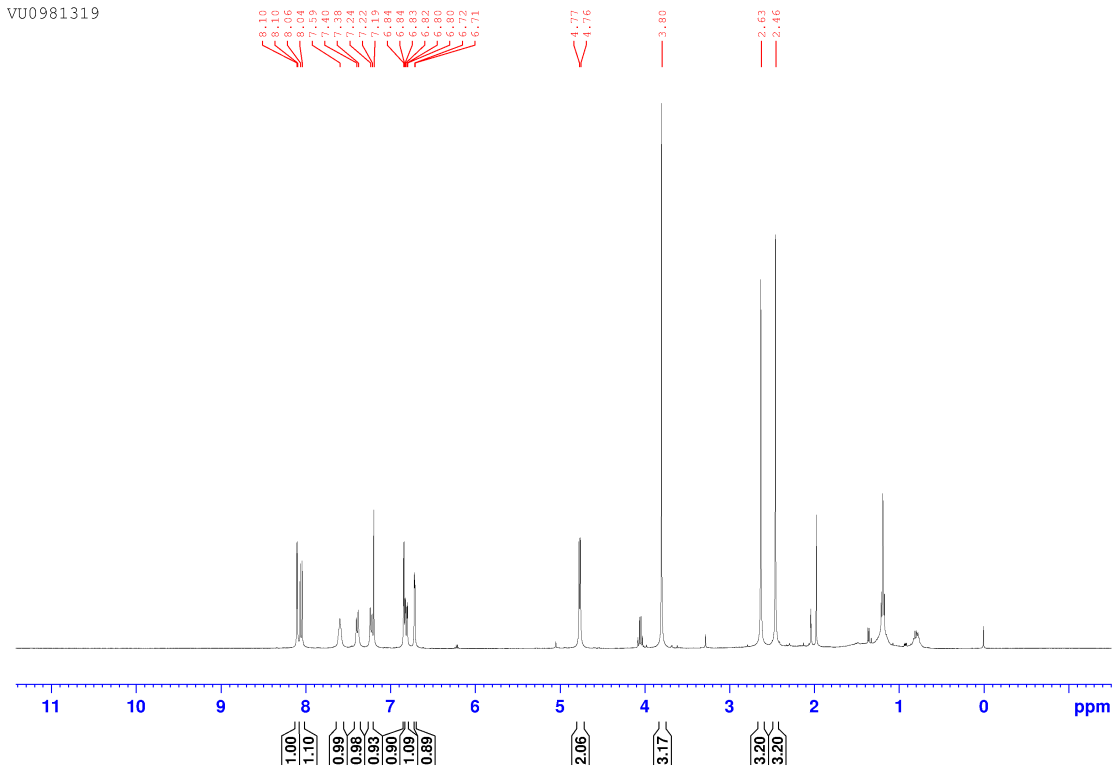

VU0981319
8.10 8.10 8.06 8.04 7.59 7.40 7.38 7.24 7.22 7.19 6.84 6.84 6.83 6.82 6.80 6.80 6.72 6.71
4.77 4.76
3.80
2.63 2.46
ppm
1.00 1.10 0.99 0.98 0.93 0.90 1.09 0.89 2.06 3.17 3.20 3.20

(7t)

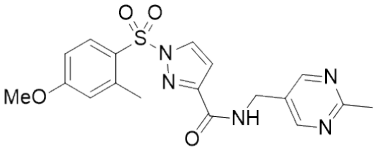
O
O
S
N
N
MeO
NH
O
N
N

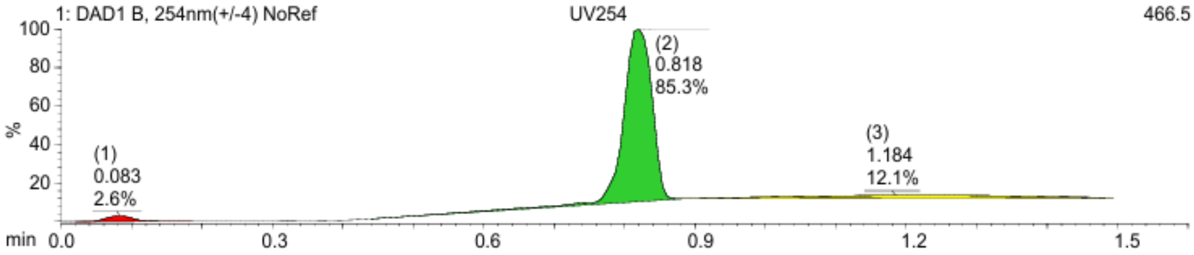
1: DAD1 B, 254nm(+/-4) NoRef
UV254
466.5
100
80
60
40
20
%
(1)
0.083
2.6%
(2)
0.818
85.3%
(3)
1.184
12.1%
min 0.0
0.3
0.6
0.9
1.2
1.5

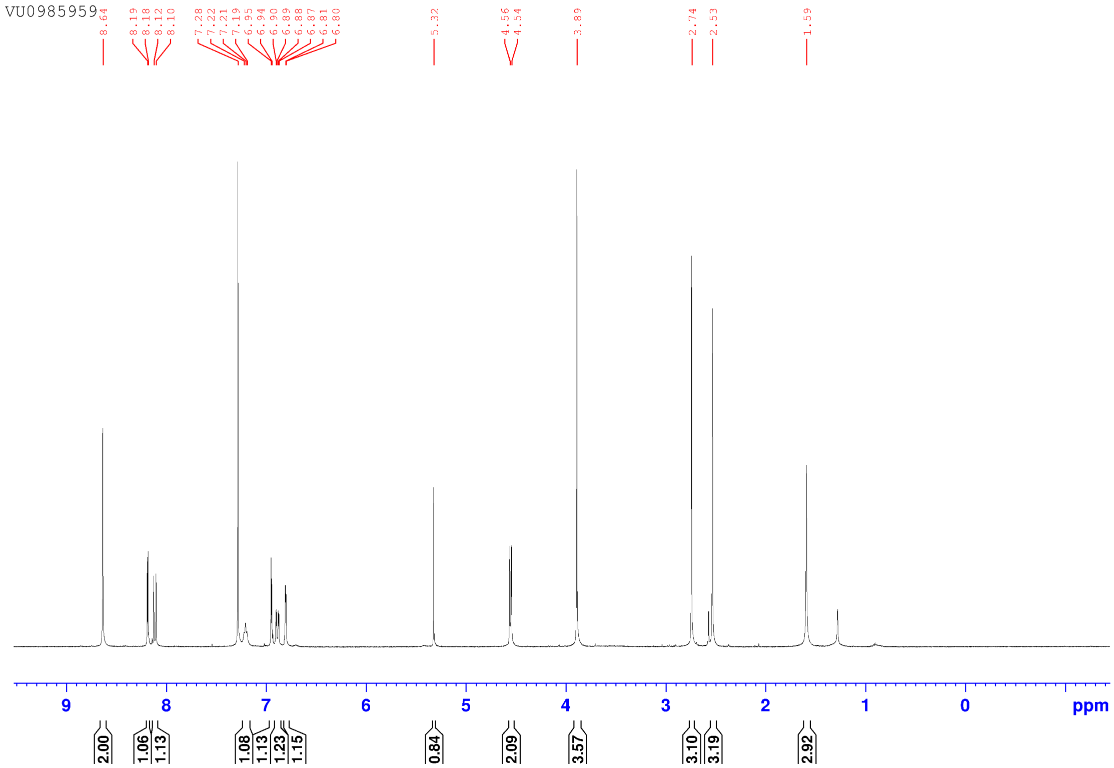
VU0985959
8.64
8.19
8.18
8.12
8.10
7.28
7.22
7.21
7.19
6.95
6.94
6.90
6.89
6.88
6.87
6.81
6.80
5.32
4.56
4.54
3.89
2.74
2.53
1.59
9
8
7
6
5
4
3
2
1
0
ppm
2.00
1.06
1.13
1.08
1.13
1.23
1.15
0.84
2.09
3.57
3.10
3.19
2.92

(7u)

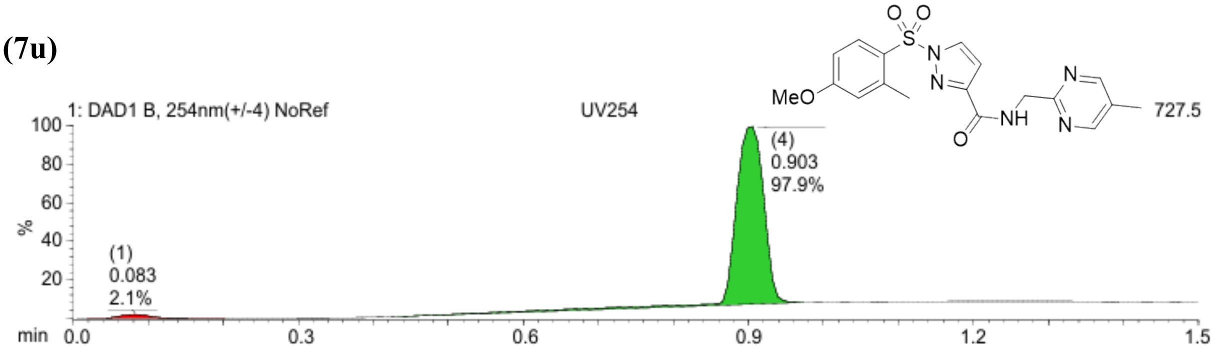

1: DAD1 B, 254nm(+/-4) NoRef
UV254
727.5
(1)
0.083
2.1%
(4)
0.903
97.9%
%
min
MeO
NH

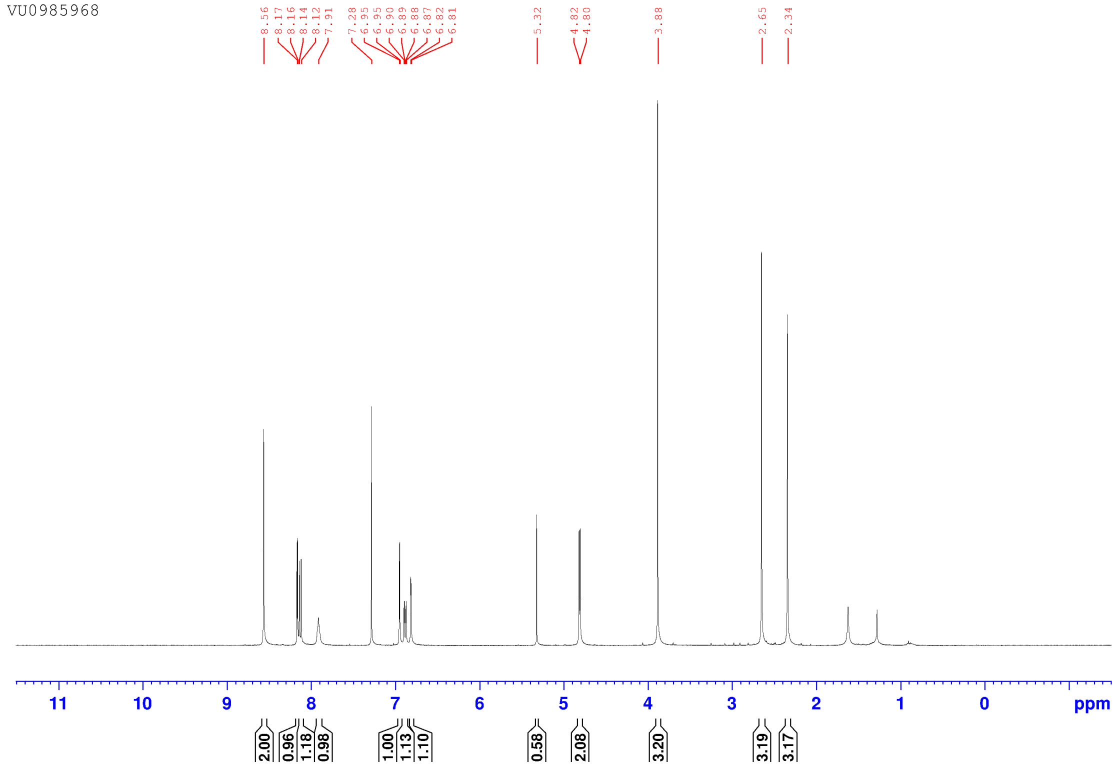

VU0985968
8.56 8.17 8.16 8.14 8.12 7.91 7.28 6.95 6.95 6.90 6.89 6.88 6.87 6.82 6.81 5.32 4.82 4.80 3.88 2.65 2.34
2.00 0.96 1.18 0.98 1.00 1.13 1.10 0.58 2.08 3.20 3.19 3.17
ppm

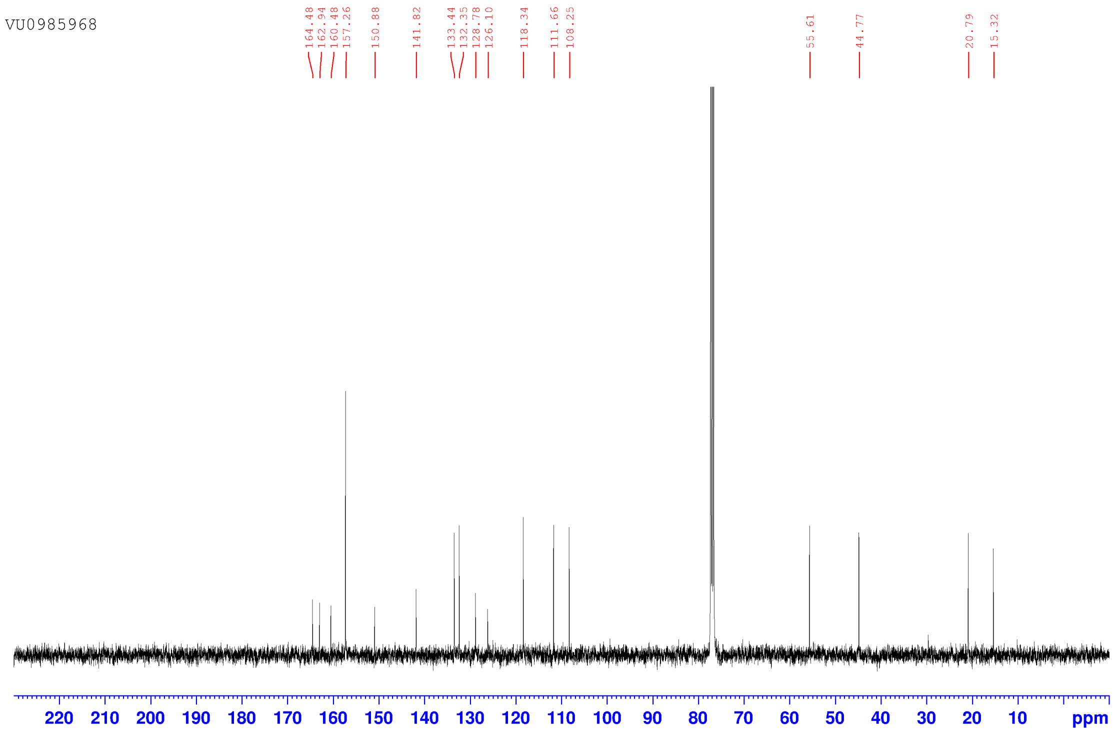
VU0985968
164.48
162.94
160.48
157.26
150.88
141.82
133.44
132.35
128.78
126.10
118.34
111.66
108.25
55.61
44.77
20.79
15.32
220 210 200 190 180 170 160 150 140 130 120 110 100 90 80 70 60 50 40 30 20 10 ppm

(7w)

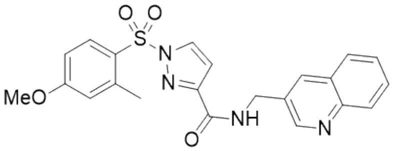
MeO
NH
N
O

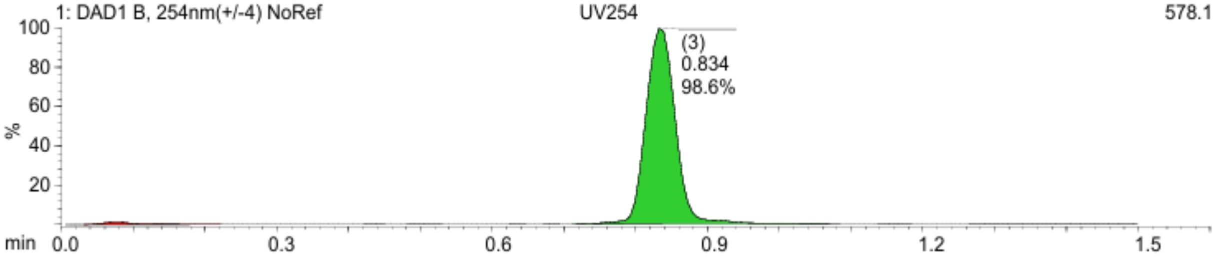
1: DAD1 B, 254nm(+/-4) NoRef
UV254
578.1
(3)
0.834
98.6%
%
min

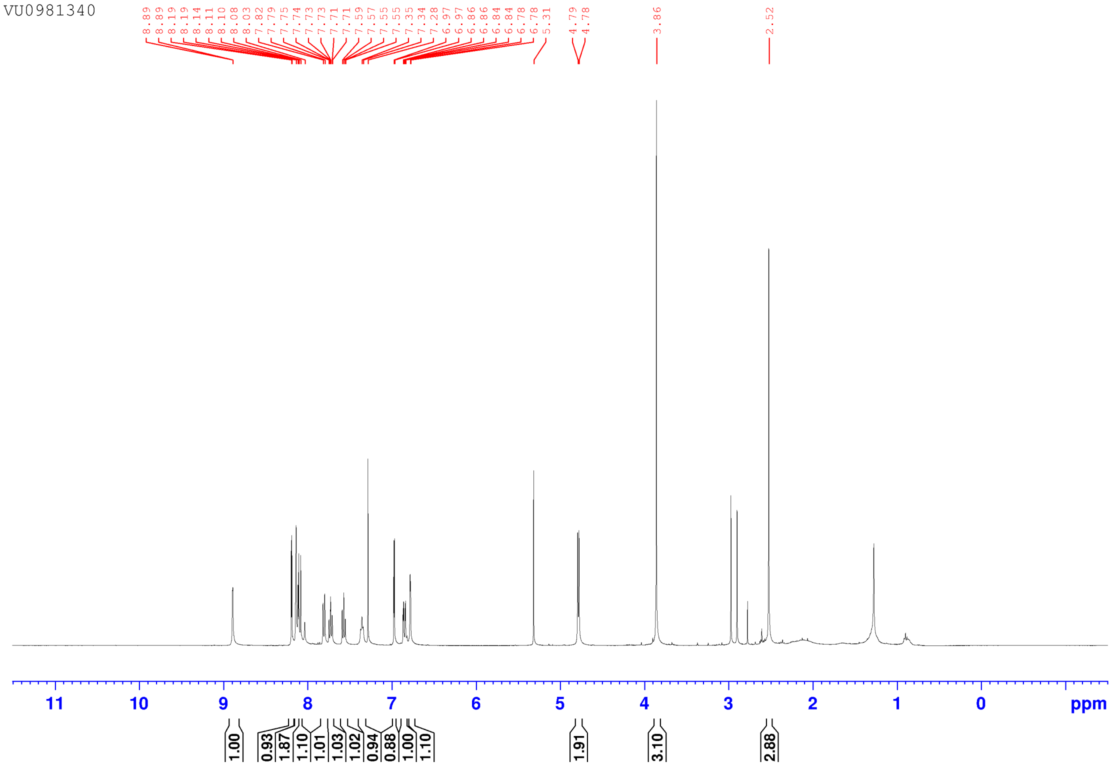
VU0981340
ppm

(7x)

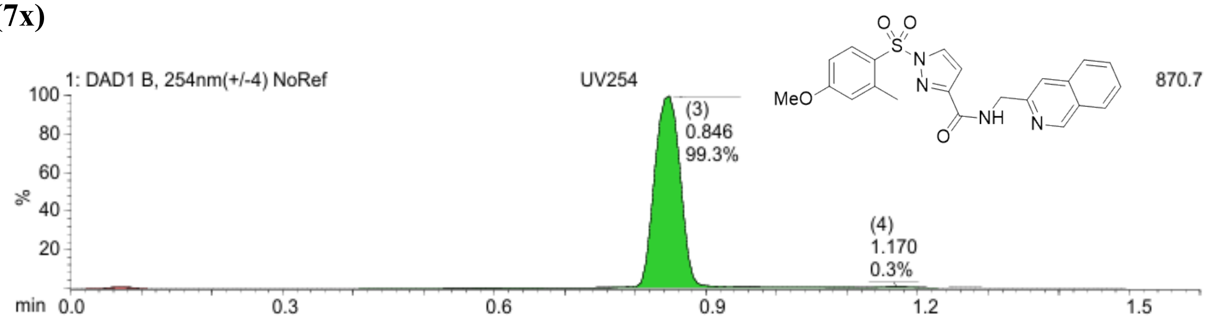
1: DAD1 B, 254nm(+/-4) NoRef
UV254
870.7
MeO
NH
O
N
(3)
0.846
99.3%
(4)
1.170
0.3%
%
min

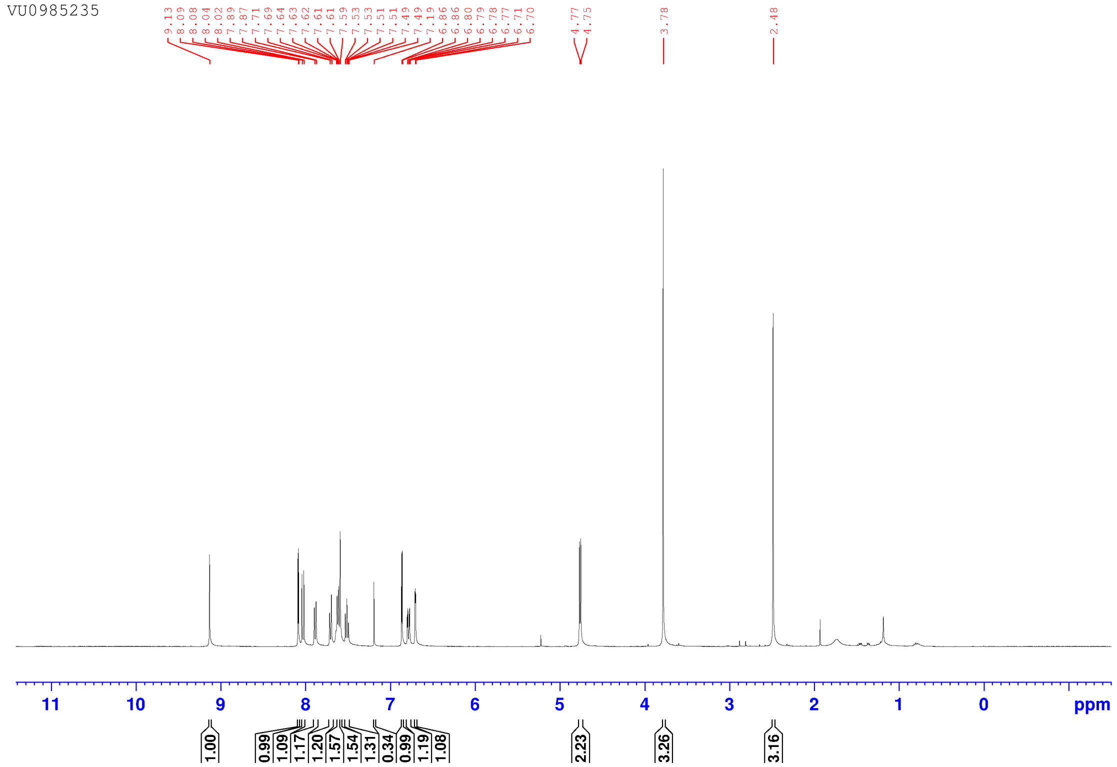
VU0985235
9.13
4.77
4.75
3.78
2.48
ppm
1.00
2.23
3.26
3.16

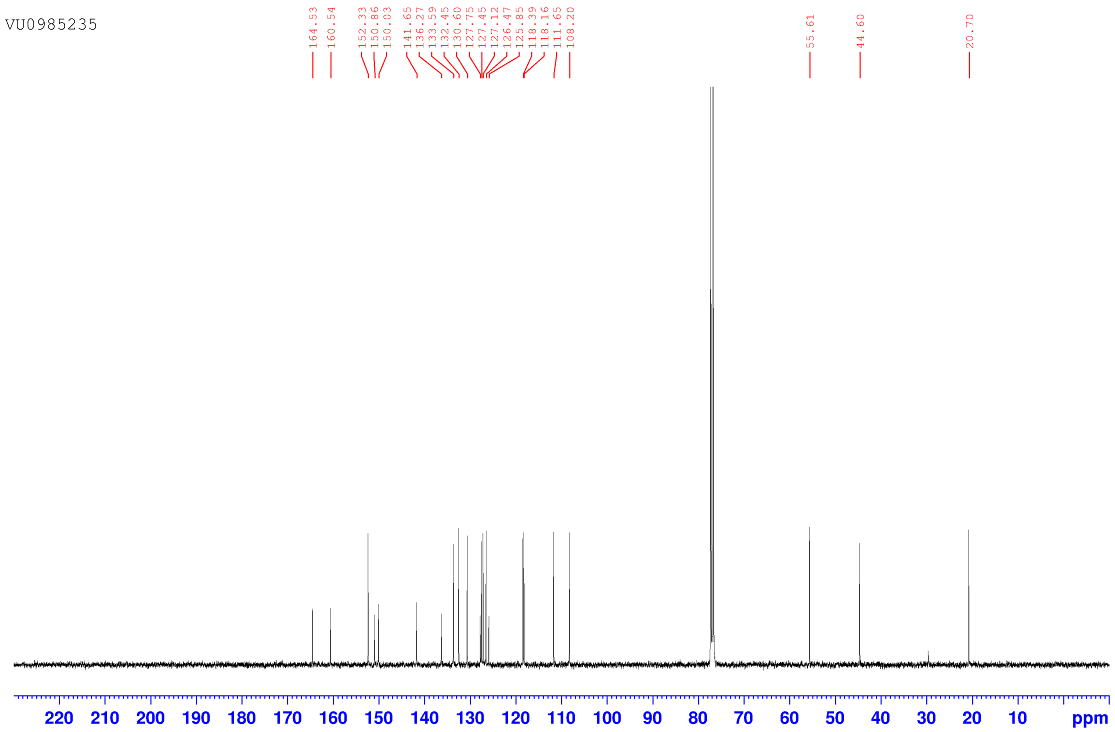
VU0985235
164.53
160.54
152.33
150.86
150.03
141.65
136.27
133.59
132.45
130.60
127.75
127.45
127.12
126.47
125.85
118.39
118.16
111.65
108.20
55.61
44.60
20.70
220 210 200 190 180 170 160 150 140 130 120 110 100 90 80 70 60 50 40 30 20 10 ppm

(7y)

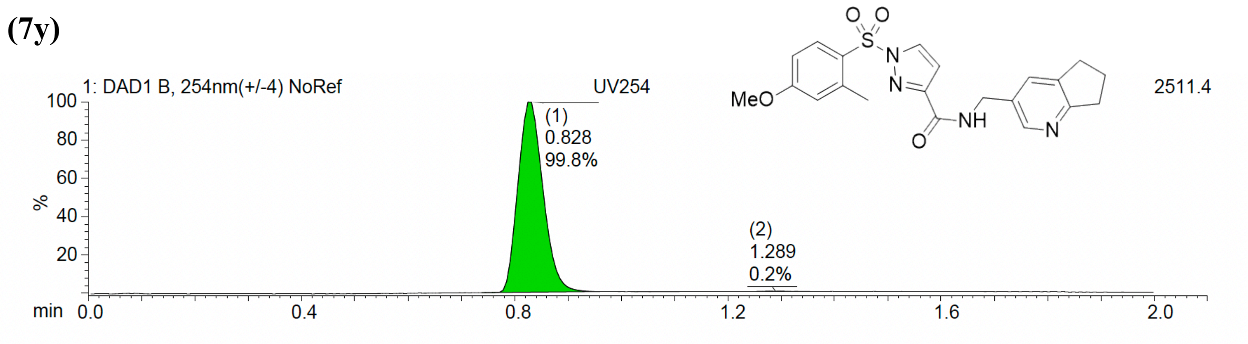
1: DAD1 B, 254nm(+/-4) NoRef
UV254
2511.4
(1)
0.828
99.8%
(2)
1.289
0.2%
%
min
MeO
NH
N
O

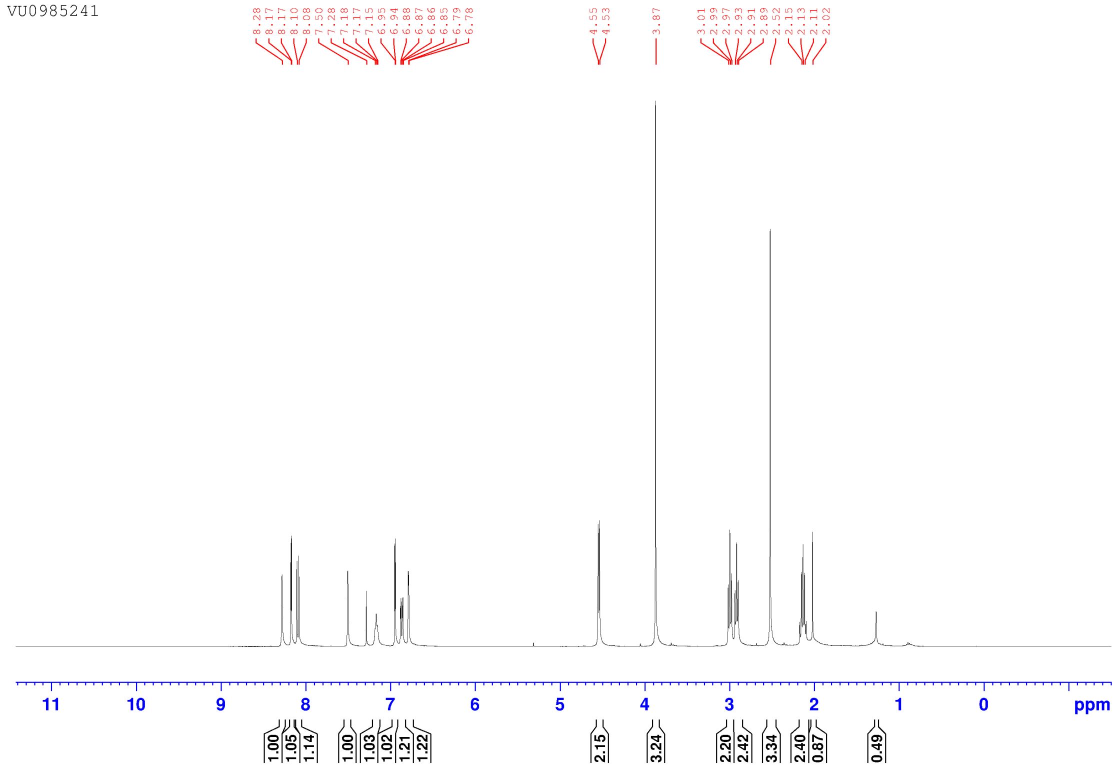
VU0985241
8.28
8.17
8.17
8.10
8.08
7.50
7.28
7.18
7.17
7.15
6.95
6.94
6.88
6.87
6.86
6.85
6.79
6.78
4.55
4.53
3.87
3.01
2.99
2.97
2.93
2.91
2.89
2.52
2.15
2.13
2.11
2.02
ppm
1.00
1.05
1.14
1.00
1.03
1.02
1.21
1.22
2.15
3.24
2.20
2.42
3.34
2.40
0.87
0.49

VU0985241

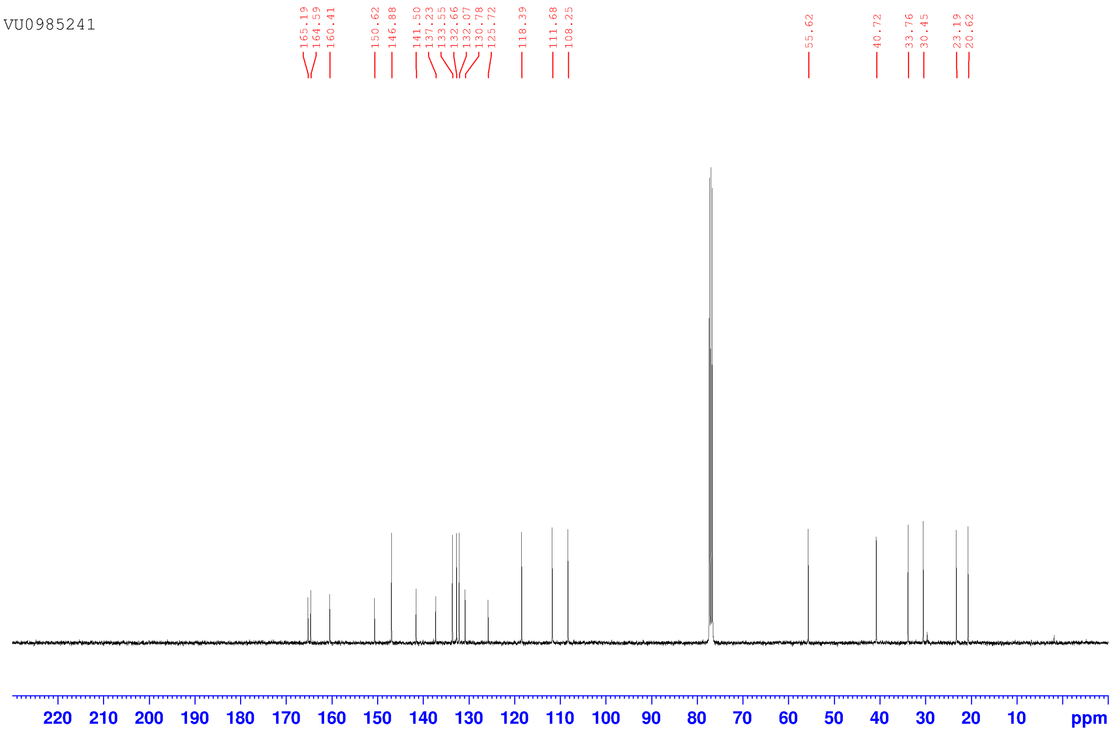

165.19
164.59
160.41
150.62
146.88
141.50
137.23
133.55
132.66
132.07
130.78
125.72
118.39
111.68
108.25
55.62
40.72
33.76
30.45
23.19
20.62
220 210 200 190 180 170 160 150 140 130 120 110 100 90 80 70 60 50 40 30 20 10 ppm

(7z)

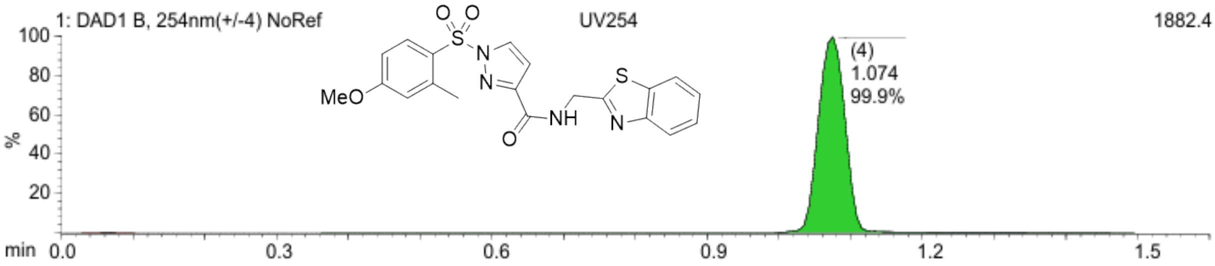
1: DAD1 B, 254nm(+/-4) NoRef
UV254
1882.4
(4)
1.074
99.9%
MeO
NH
min

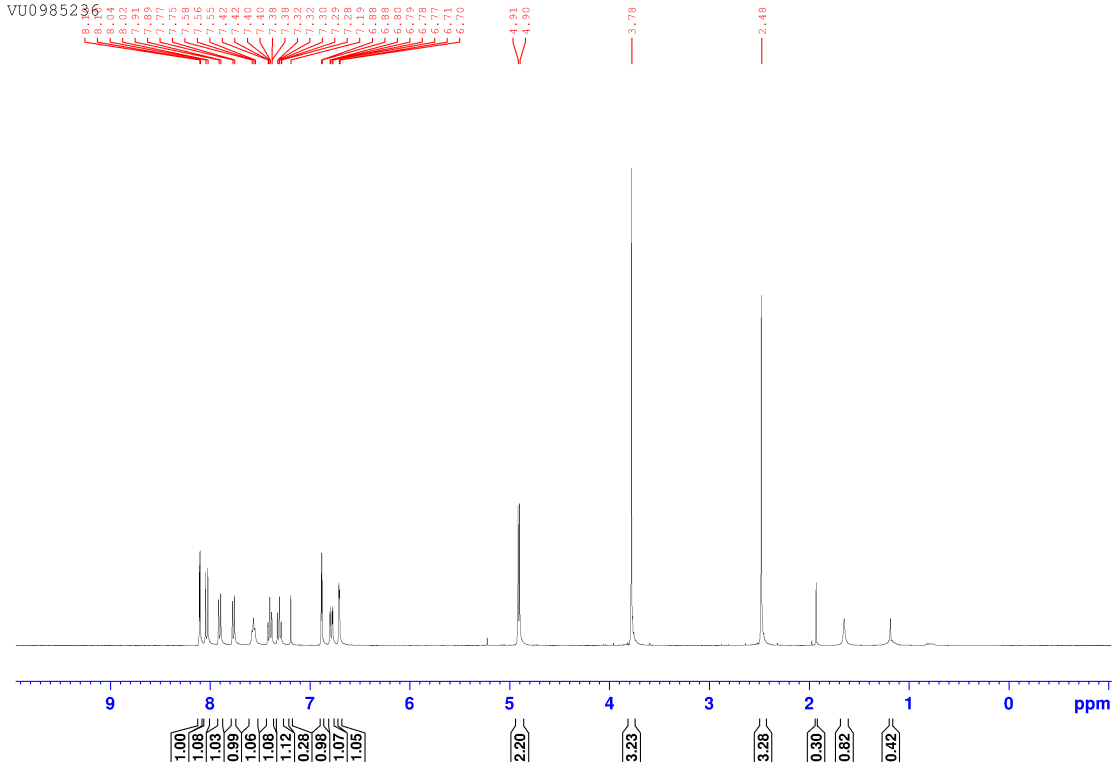
VU0985236
4.91
4.90
3.78
2.48
ppm
1.00
1.08
1.03
0.99
1.06
1.08
1.12
0.28
0.98
1.07
1.05
2.20
3.23
3.28
0.30
0.82
0.42

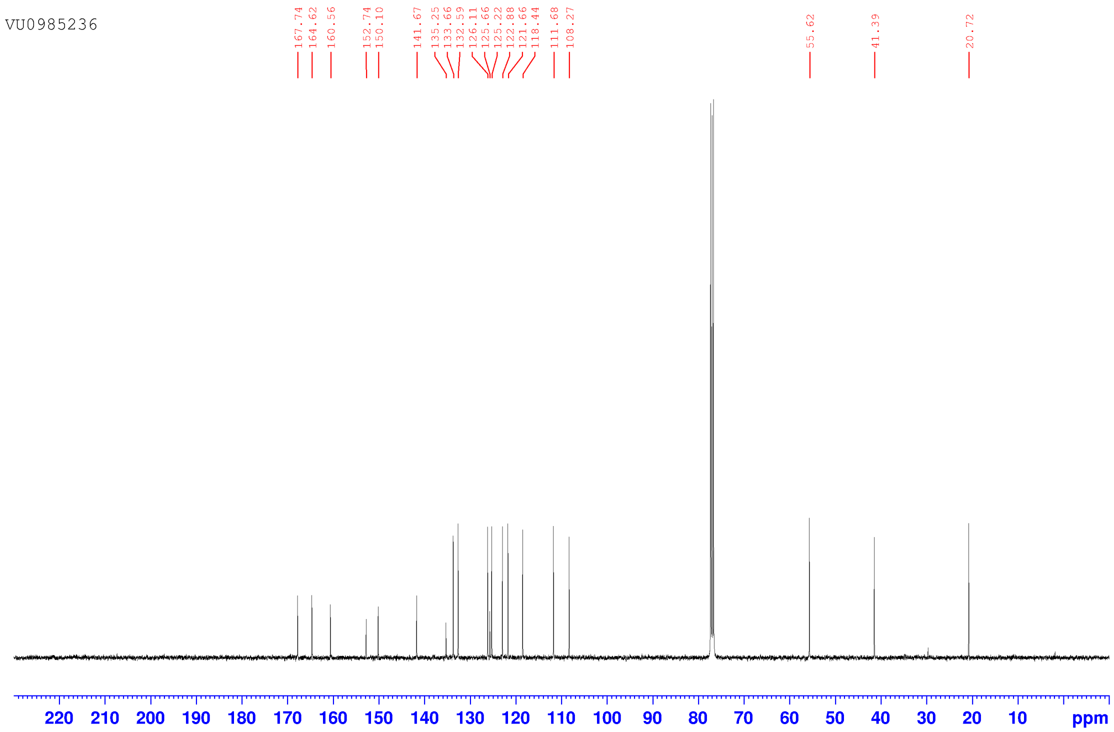
VU0985236
167.74
164.62
160.56
152.74
150.10
141.67
135.25
133.66
132.59
126.11
125.66
125.22
122.88
121.66
118.44
111.68
108.27
55.62
41.39
20.72
220 210 200 190 180 170 160 150 140 130 120 110 100 90 80 70 60 50 40 30 20 10 ppm

(7aa)

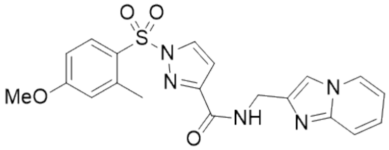
MeO
O
O
S
N
N
O
NH
N
N

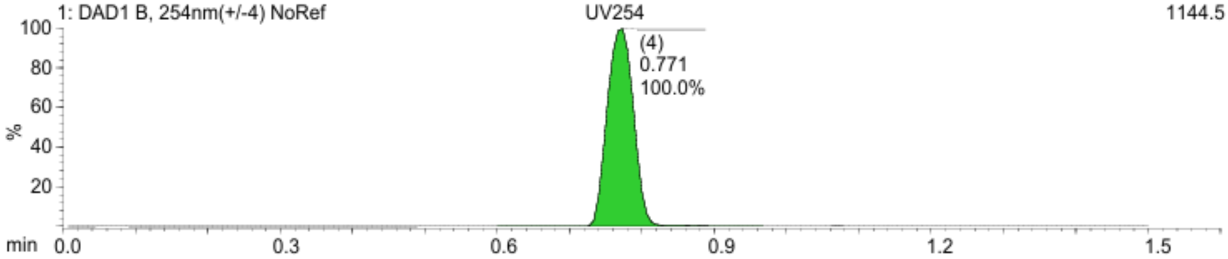
1: DAD1 B, 254nm(+/-4) NoRef
UV254
1144.5
(4)
0.771
100.0%
%
min

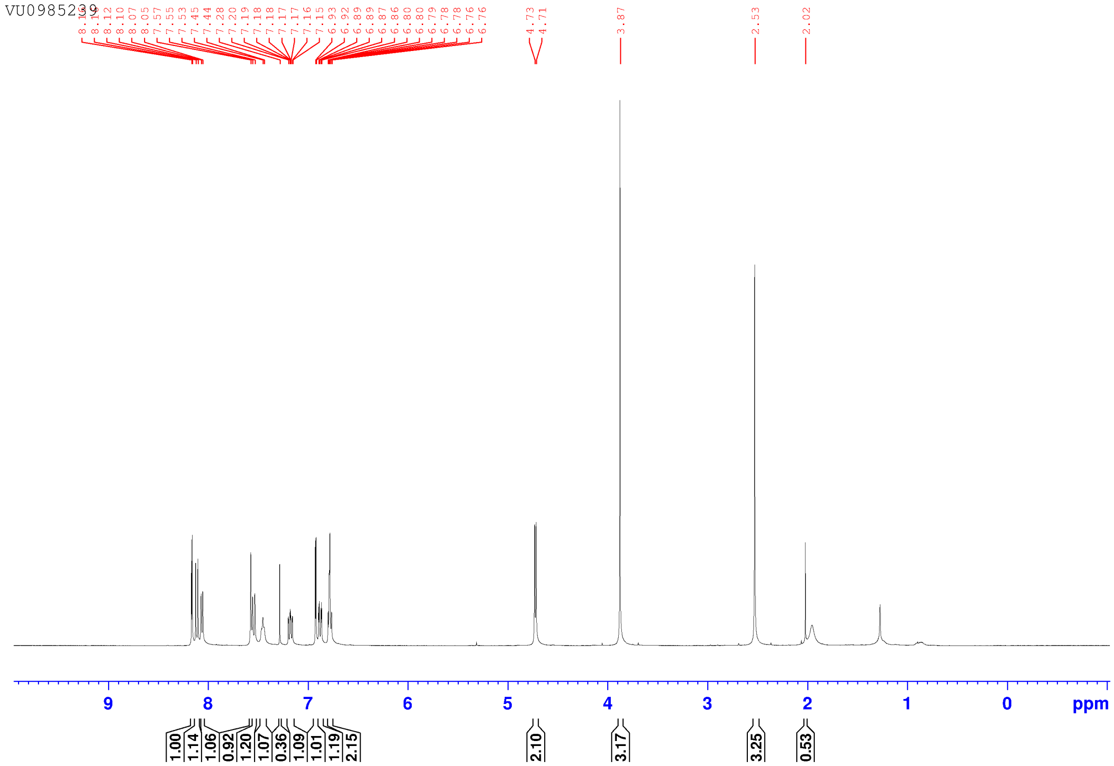
VU0985239
4.73
4.71
3.87
2.53
2.02
ppm
2.10
3.17
3.25
0.53

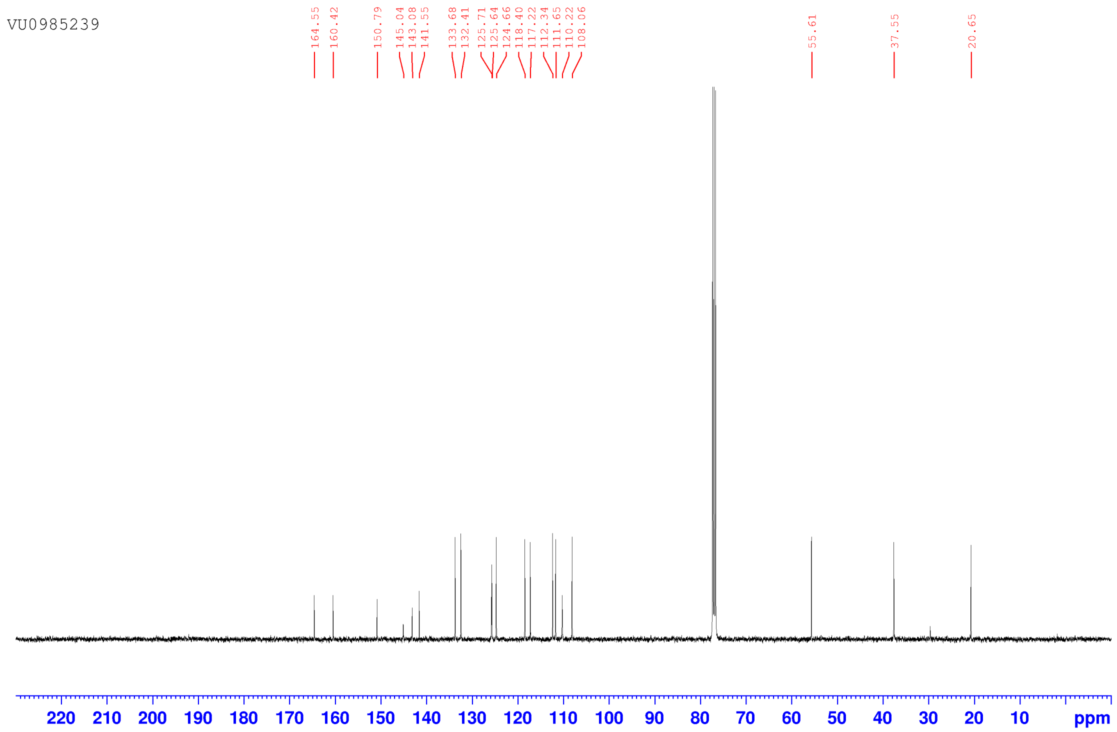

VU0985239
164.55
160.42
150.79
145.04
143.08
141.55
133.68
132.41
125.71
125.64
124.66
118.40
117.22
112.34
111.65
110.22
108.06
55.61
37.55
20.65
220 210 200 190 180 170 160 150 140 130 120 110 100 90 80 70 60 50 40 30 20 10 ppm

8a

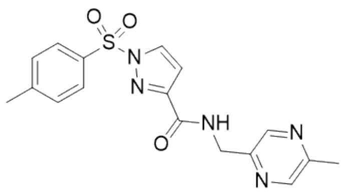

O
O
S
N
N
NH
O
N
N

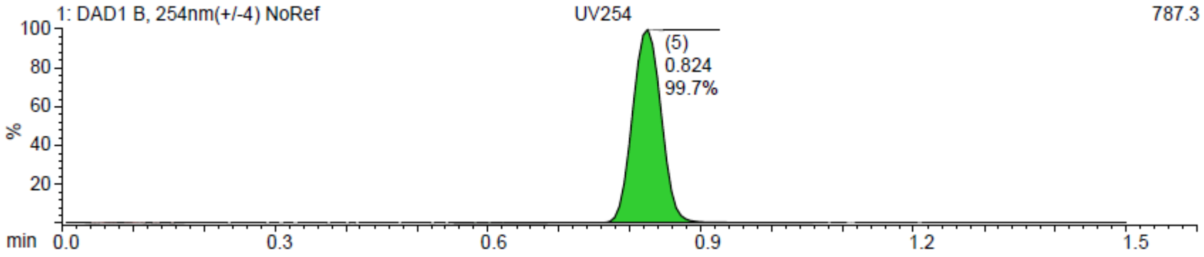

1: DAD1 B, 254nm(+/-4) NoRef
UV254
787.3
(5)
0.824
99.7%
%
min

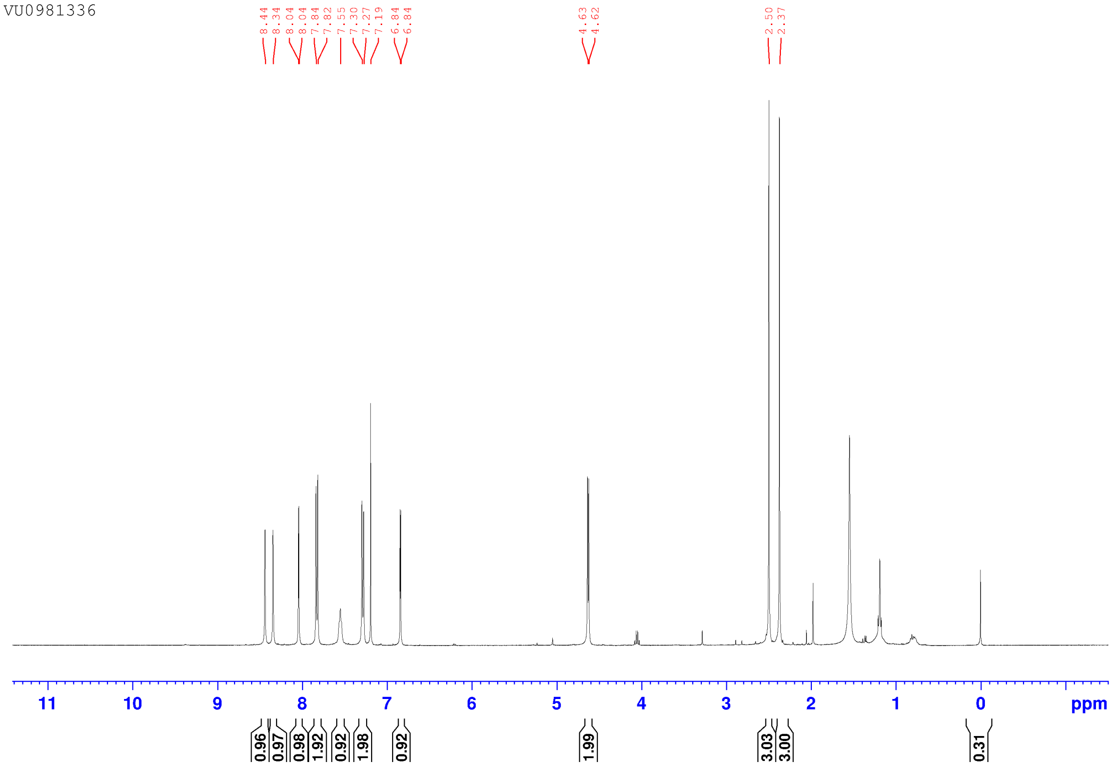

VU0981336
ppm

8b

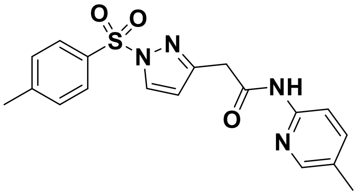

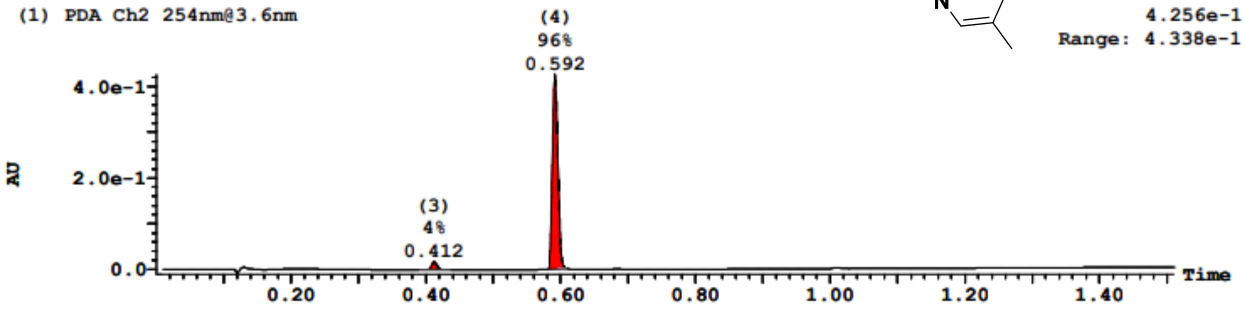

(1) PDA Ch2 254nm@3.6nm
4.256e-1
Range: 4.338e-1
(4)
96%
0.592
(3)
4%
0.412
AU
Time

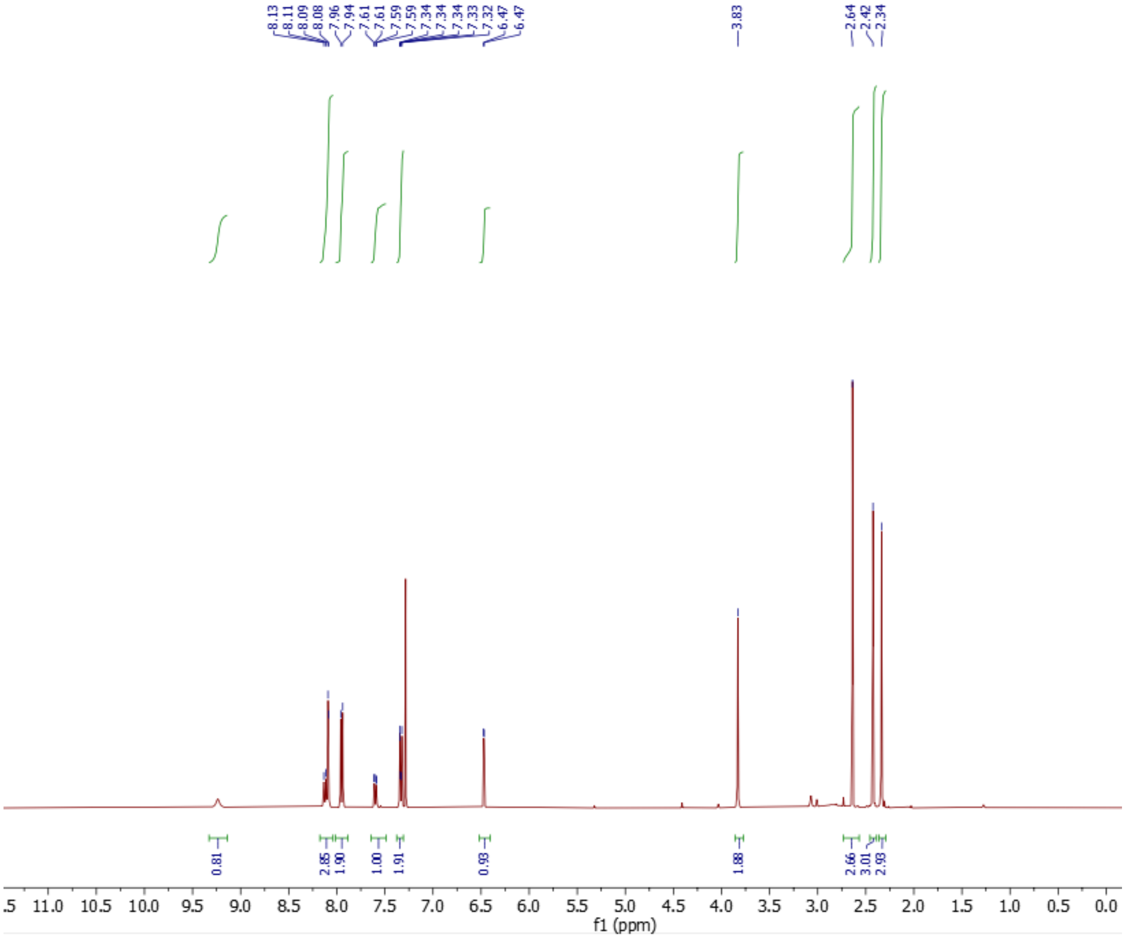

f1 (ppm)

8j

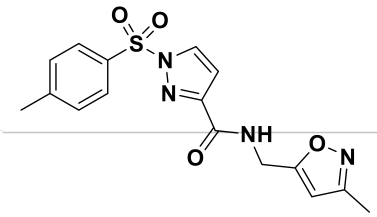

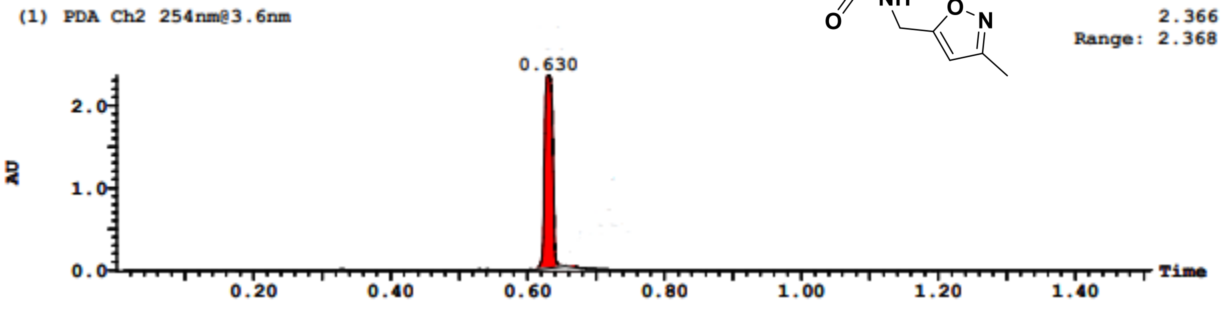

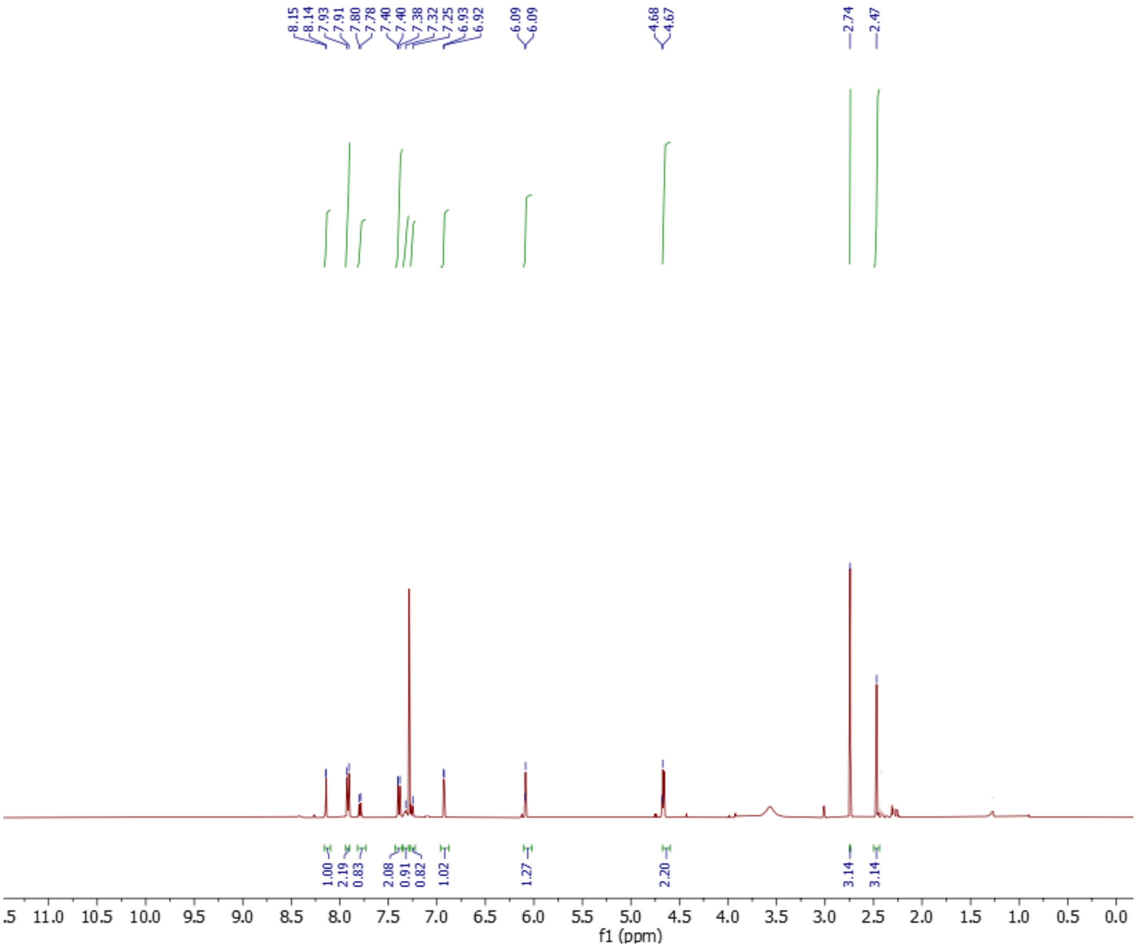

8k

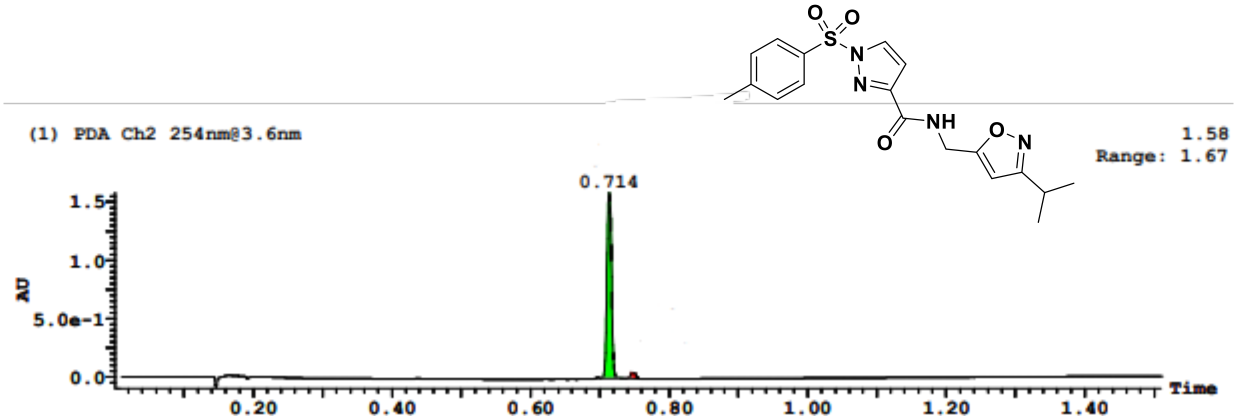

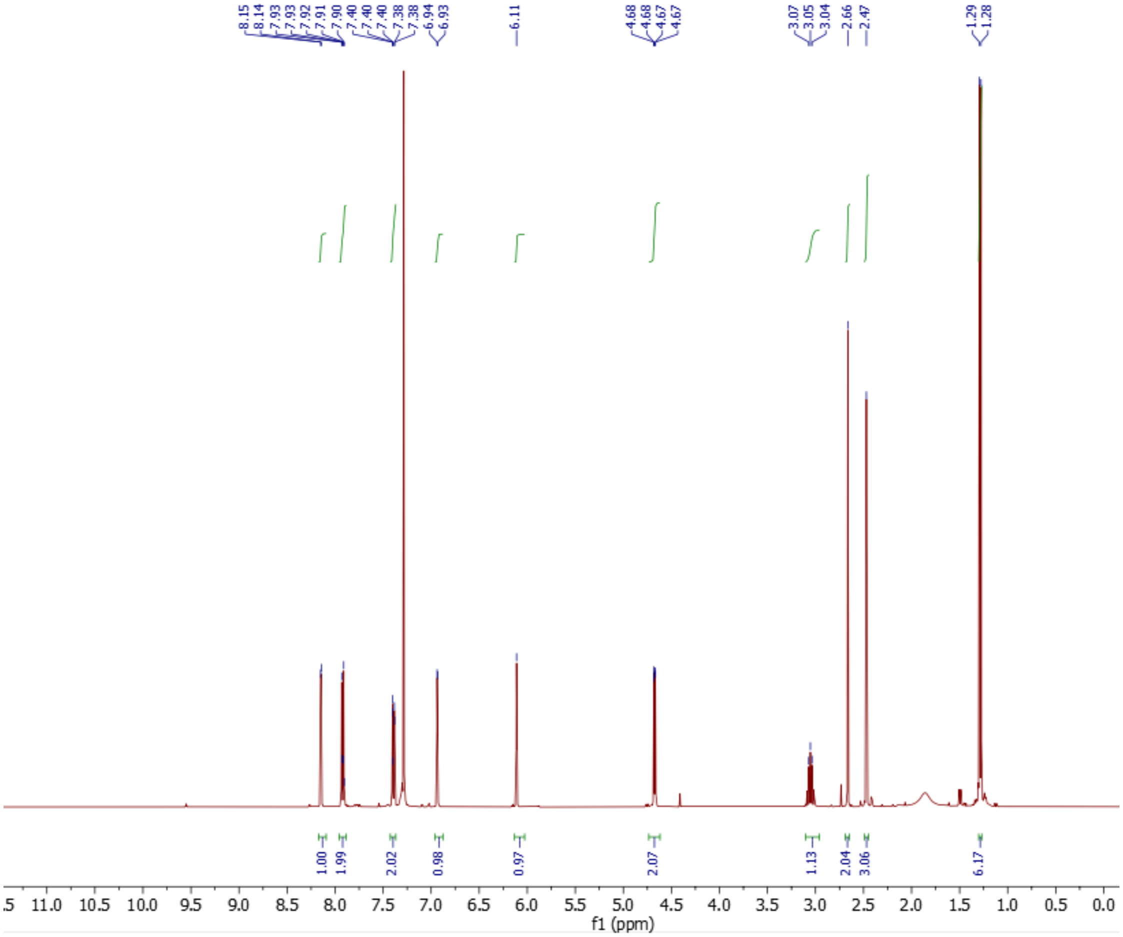

8q

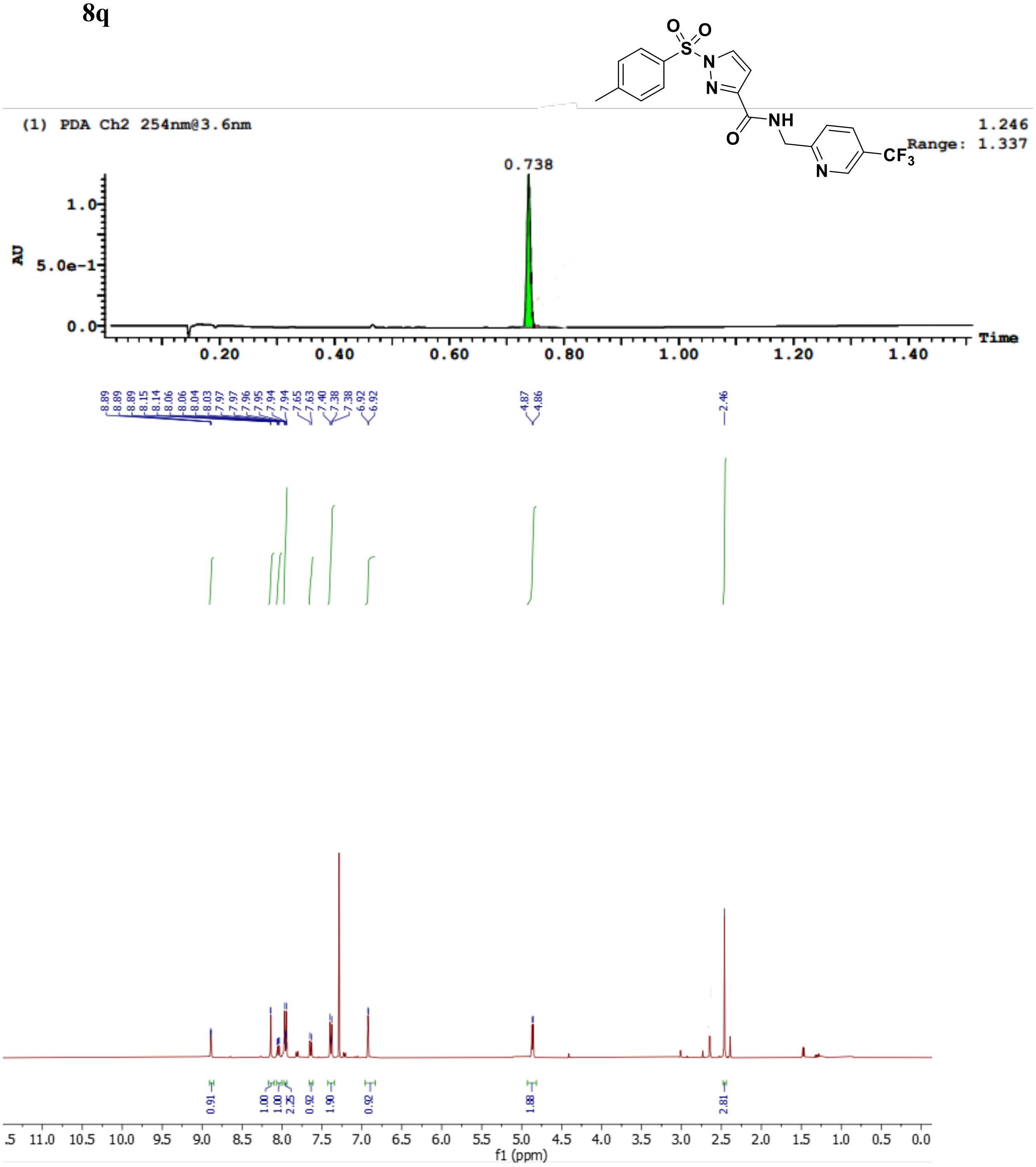

8s

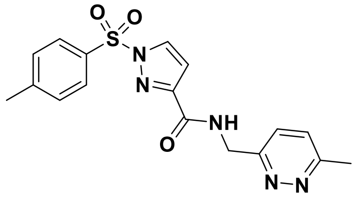

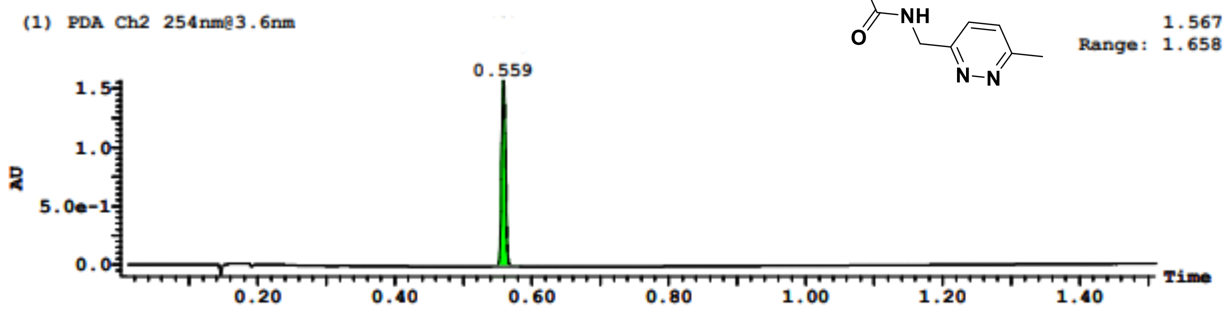

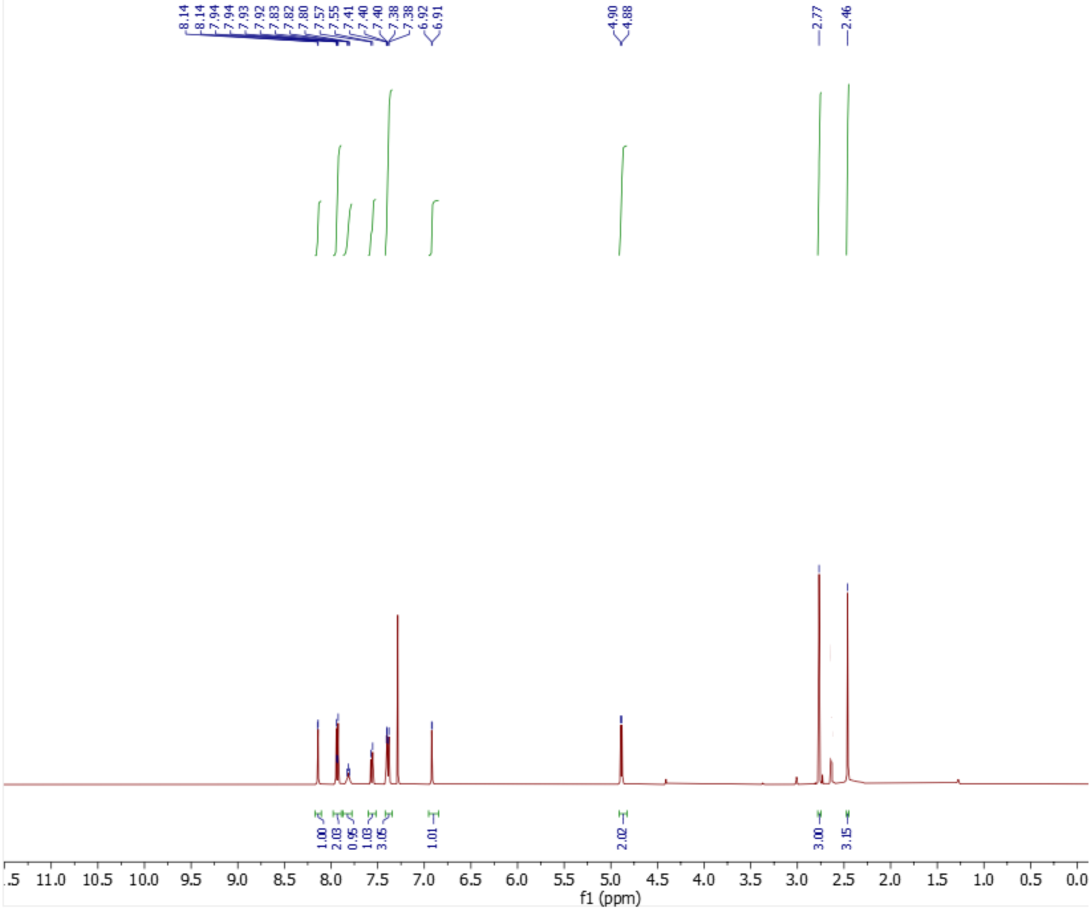

8w

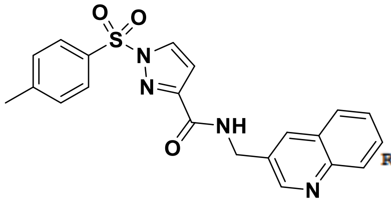
O
O
S
N
N
O
NH
N

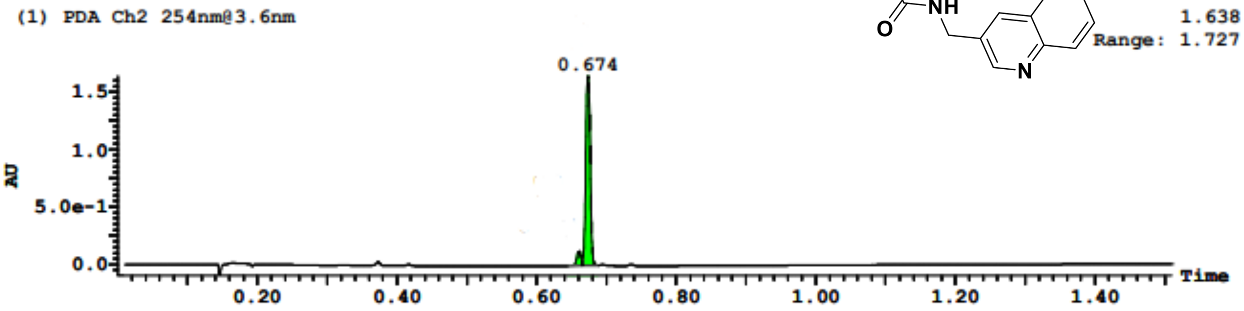
(1) PDA Ch2 254nm@3.6nm
1.638
Range: 1.727
0.674
AU
1.5
1.0
5.0e-1
0.0
0.20
0.40
0.60
0.80
1.00
1.20
1.40
Time

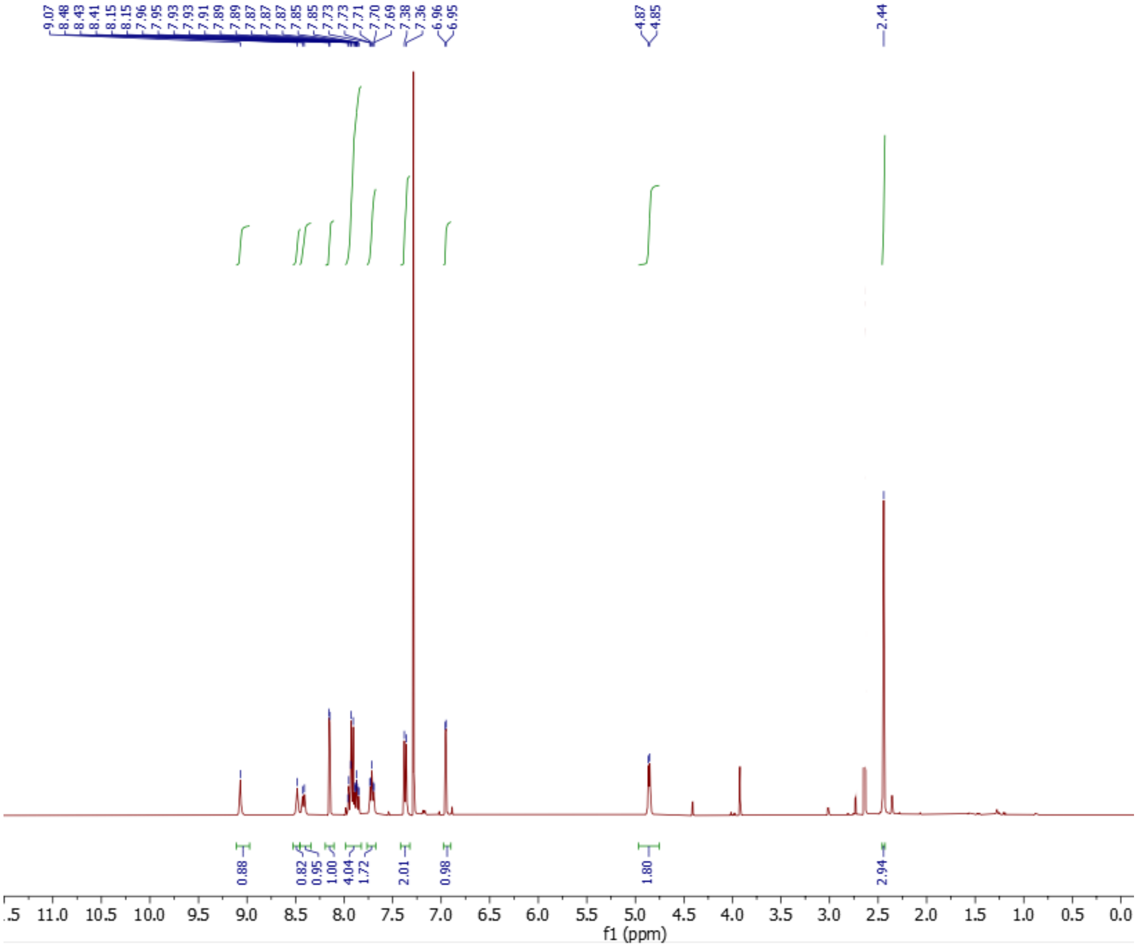
9.07
8.48
8.43
8.41
8.15
8.15
7.96
7.95
7.93
7.93
7.91
7.89
7.89
7.87
7.87
7.87
7.85
7.85
7.73
7.73
7.71
7.70
7.69
7.38
7.36
6.96
6.95
4.87
4.85
2.44
0.88
0.82
0.95
1.00
4.04
1.72
2.01
0.98
1.80
2.94
.5
11.0
10.5
10.0
9.5
9.0
8.5
8.0
7.5
7.0
6.5
6.0
5.5
5.0
4.5
4.0
3.5
3.0
2.5
2.0
1.5
1.0
0.5
0.0
f1 (ppm)

9a

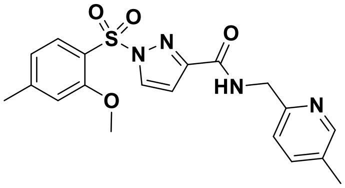

O
O
S
N
N
O
HN
N
O

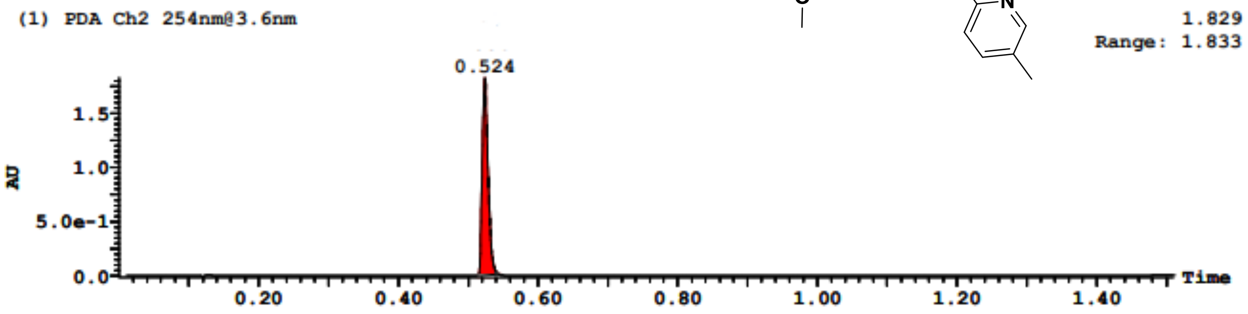

(1) PDA Ch2 254nm@3.6nm
1.829
Range: 1.833
0.524
AU
Time

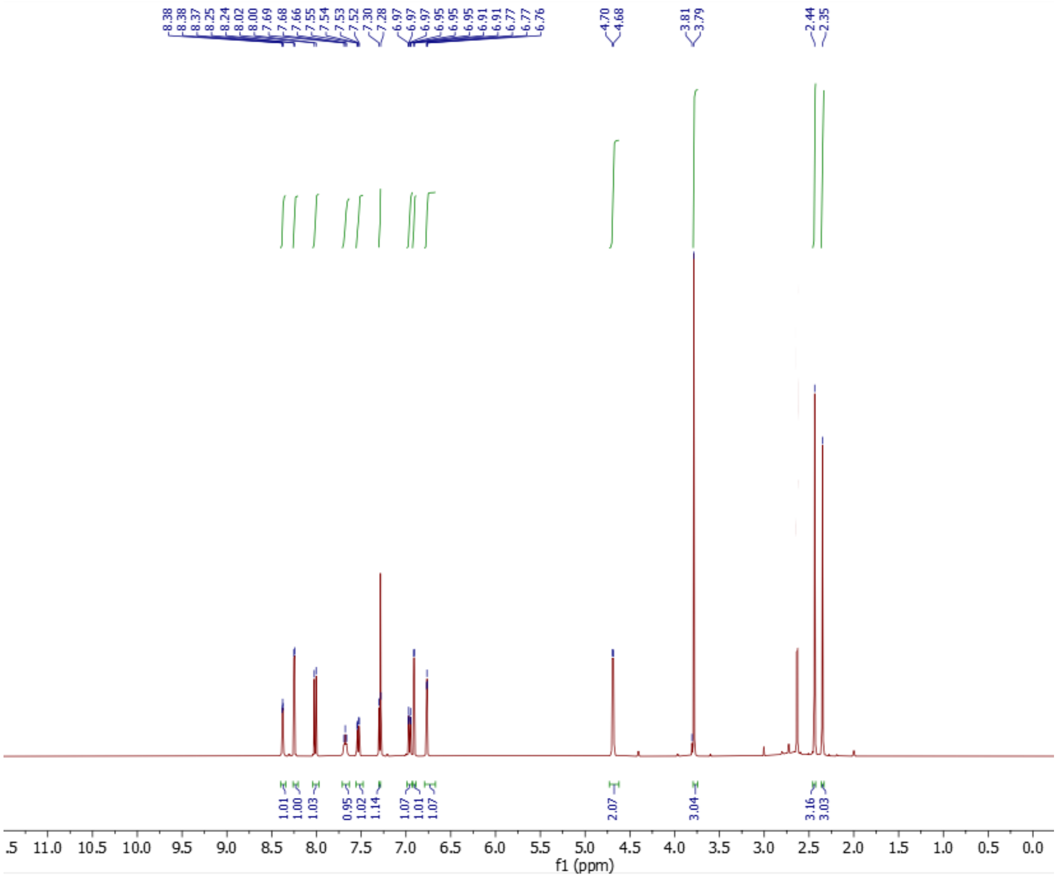

f1 (ppm)

9c

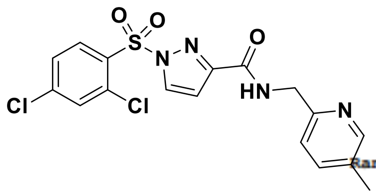

O
O
S
N
N
O
HN
N
Cl
Cl

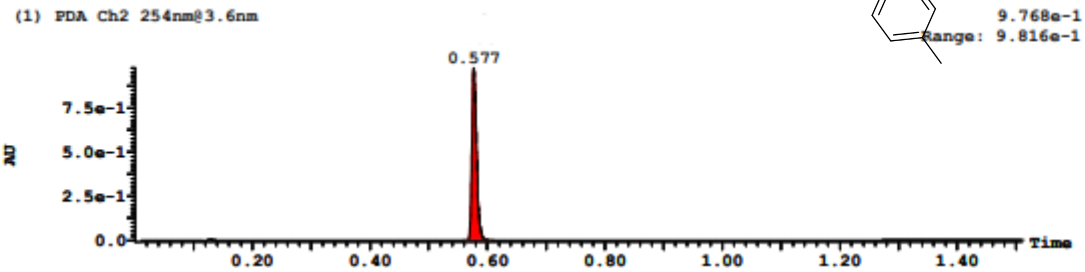

(1) PDA Ch2 254nm@3.6nm
9.768e-1
Range: 9.816e-1
0.577
AU
7.5e-1
5.0e-1
2.5e-1
0.0
0.20
0.40
0.60
0.80
1.00
1.20
1.40
Time

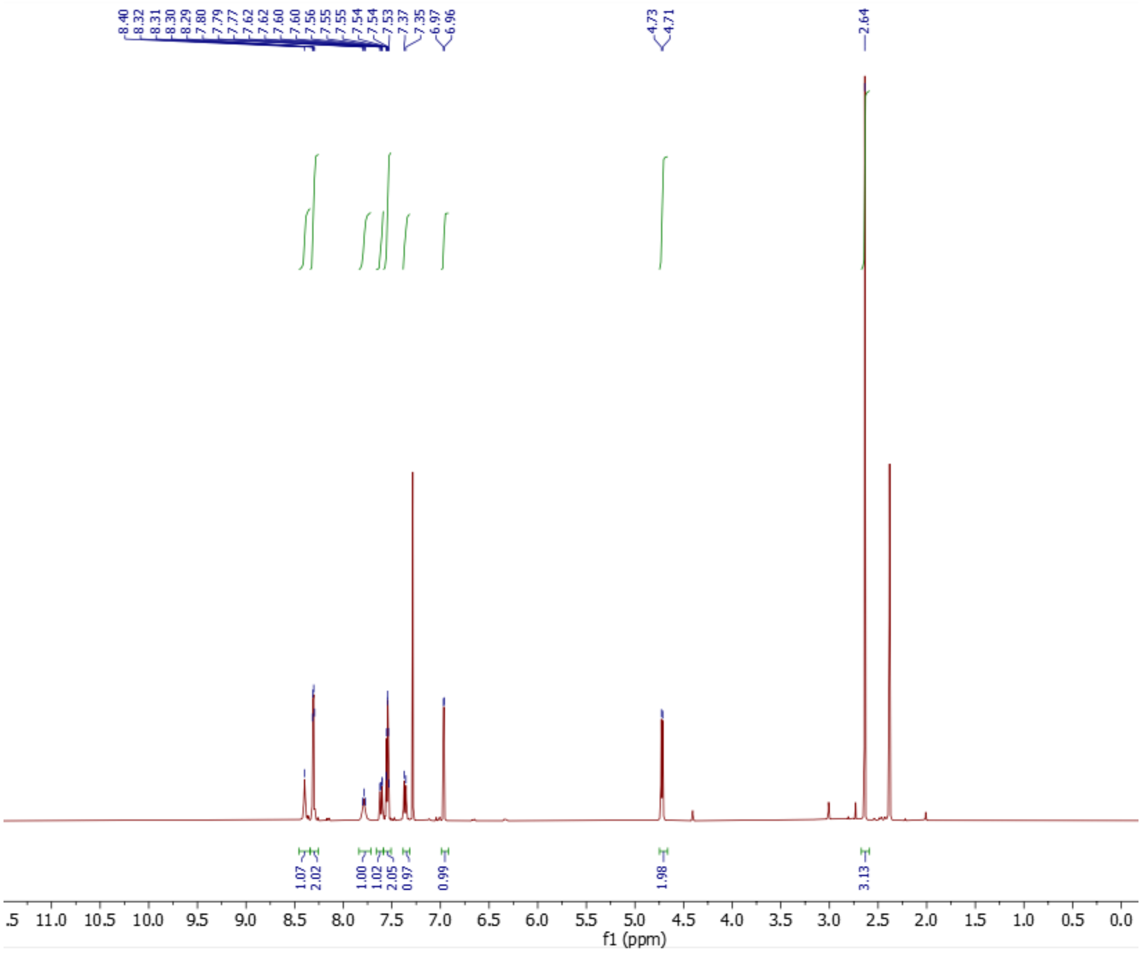

8.40
8.32
8.31
8.30
8.29
7.80
7.79
7.77
7.62
7.62
7.60
7.56
7.55
7.55
7.54
7.54
7.53
7.37
7.35
6.97
6.96
4.73
4.71
2.64
1.07
2.02
1.00
1.02
2.05
0.97
0.99
1.98
3.13
f1 (ppm)

9e

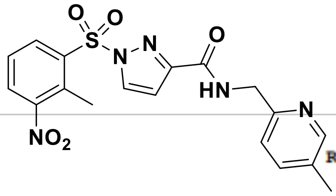
NO2
HN
N
O
N
N
S
O
O

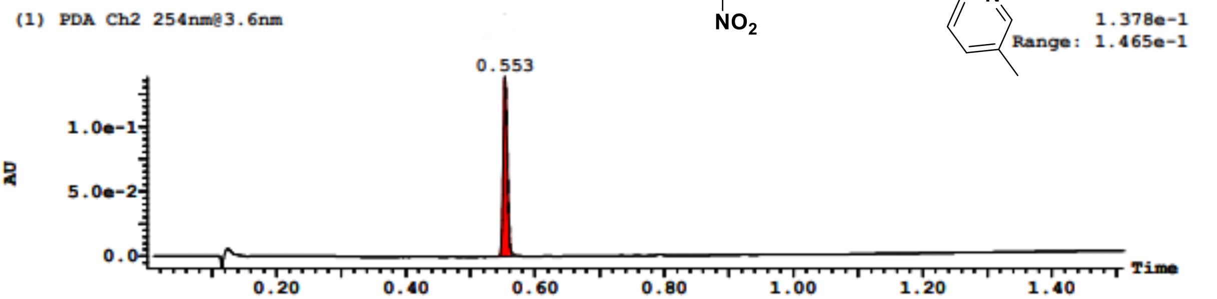
(1) PDA Ch2 254nm@3.6nm
1.378e-1
Range: 1.465e-1
0.553
AU
Time

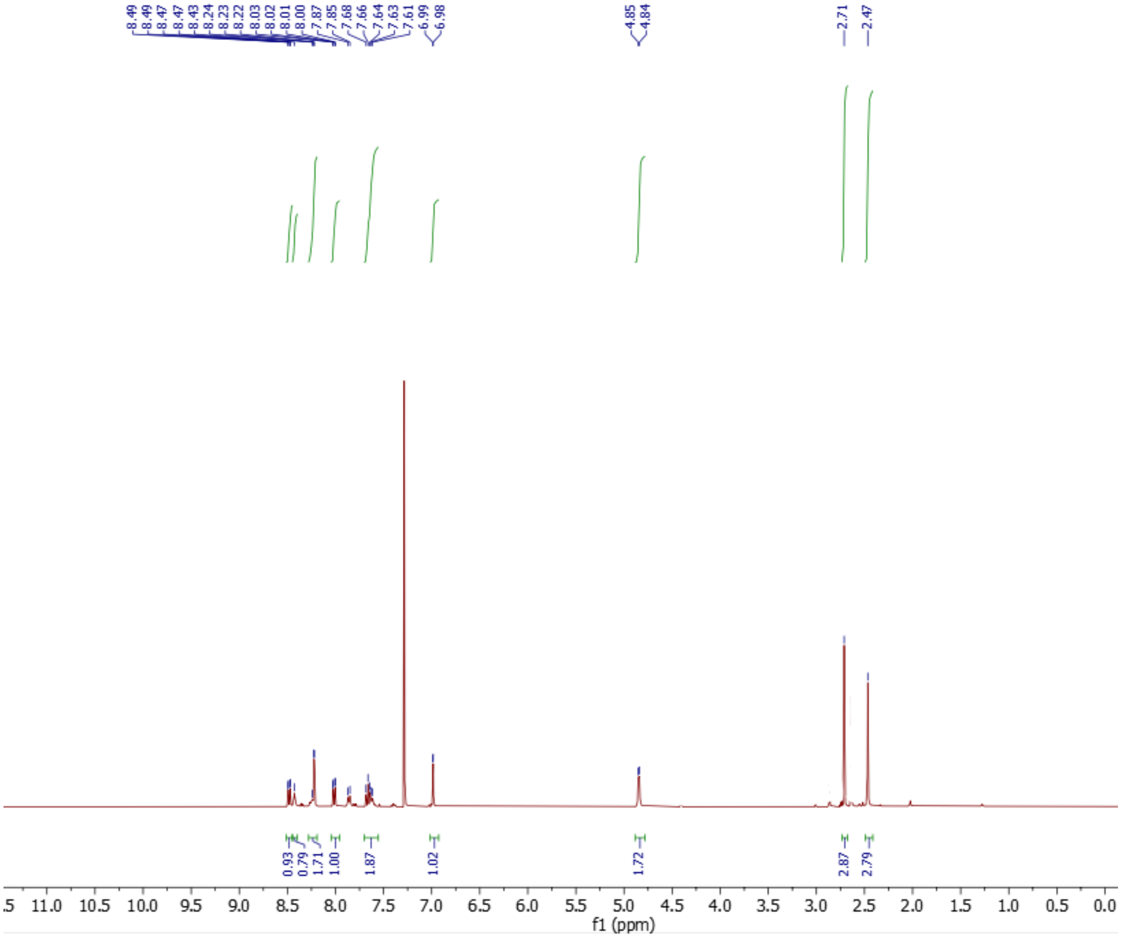
8.49 8.49 8.47 8.47 8.43 8.24 8.23 8.22 8.03 8.02 8.01 8.00 7.87 7.85 7.68 7.66 7.64 7.63 7.61 6.99 6.98
4.85 4.84
2.71
2.47
0.93 0.79 1.71 1.00 1.87 1.02 1.72 2.87 2.79
f1 (ppm)

9h

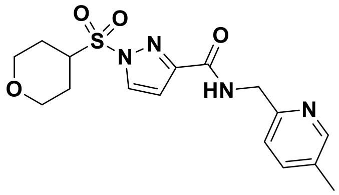

O
S
O
N
N
O
HN
N
O

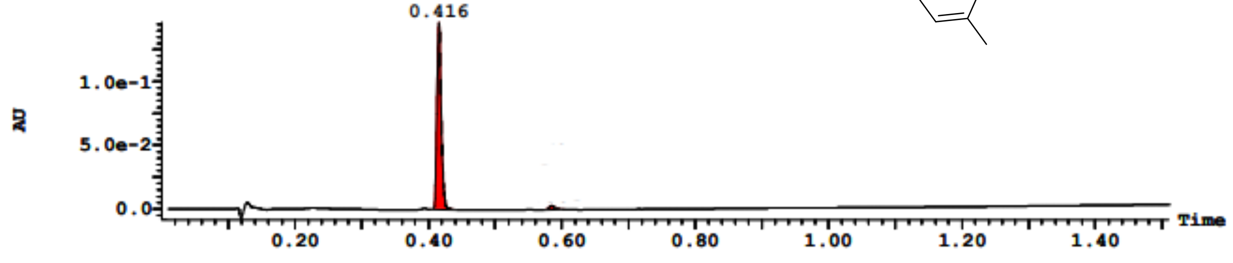

(1) PDA Ch2 254nm@3.6nm
1.464e-1
Range: 1.541e-1
0.416
AU
1.0e-1
5.0e-2
0.0
0.20
0.40
0.60
0.80
1.00
1.20
1.40
Time

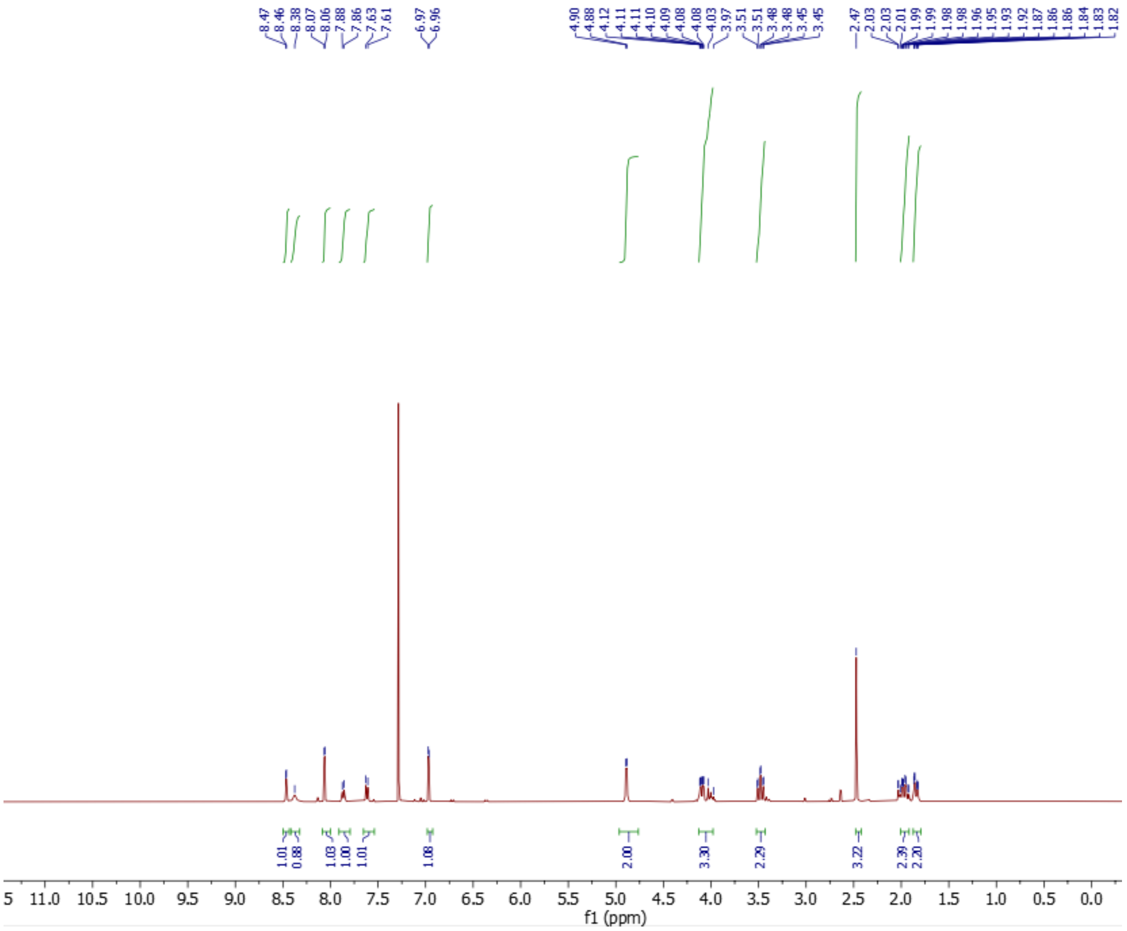

8.47
8.46
8.38
8.07
8.06
7.88
7.86
7.63
7.61
6.97
6.96
4.90
4.88
4.12
4.11
4.11
4.10
4.09
4.08
4.08
4.03
3.97
3.51
3.48
3.48
3.45
3.45
2.47
2.03
2.03
2.01
1.99
1.99
1.98
1.98
1.96
1.95
1.93
1.92
1.87
1.86
1.86
1.84
1.83
1.82
1.01
0.88
1.03
1.00
1.01
1.08
2.00
3.30
2.29
3.22
2.39
2.20
5
11.0
10.5
10.0
9.5
9.0
8.5
8.0
7.5
7.0
6.5
6.0
5.5
5.0
4.5
4.0
3.5
3.0
2.5
2.0
1.5
1.0
0.5
0.0
f1 (ppm)

9j

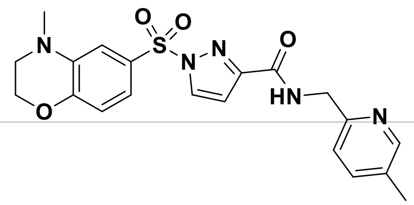

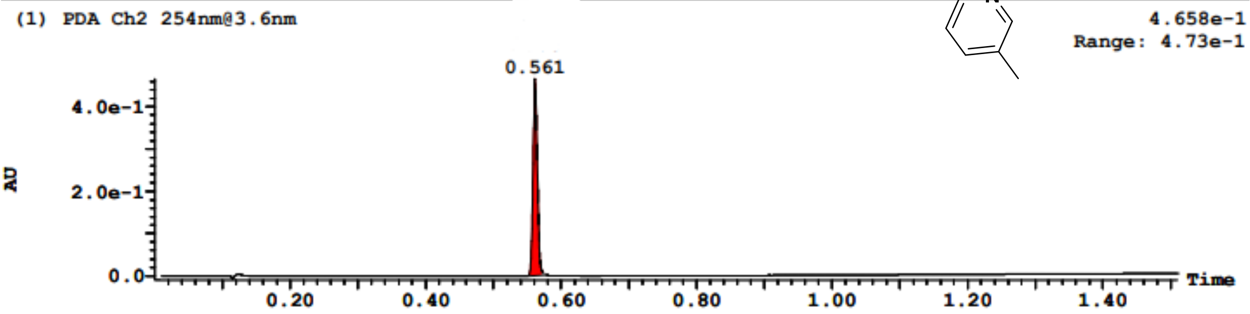

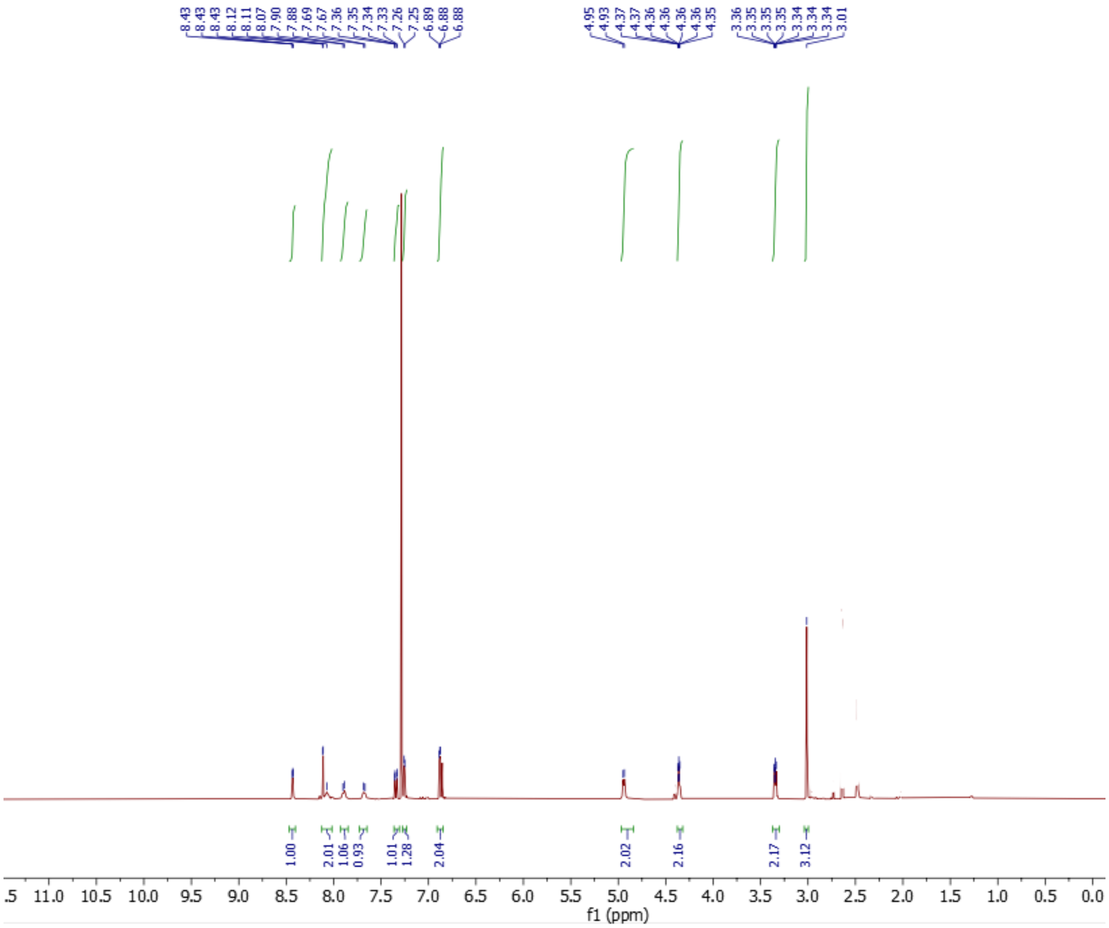

9l

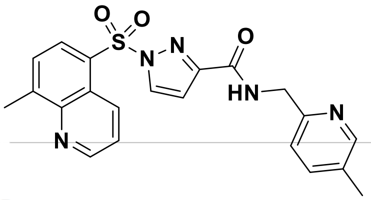

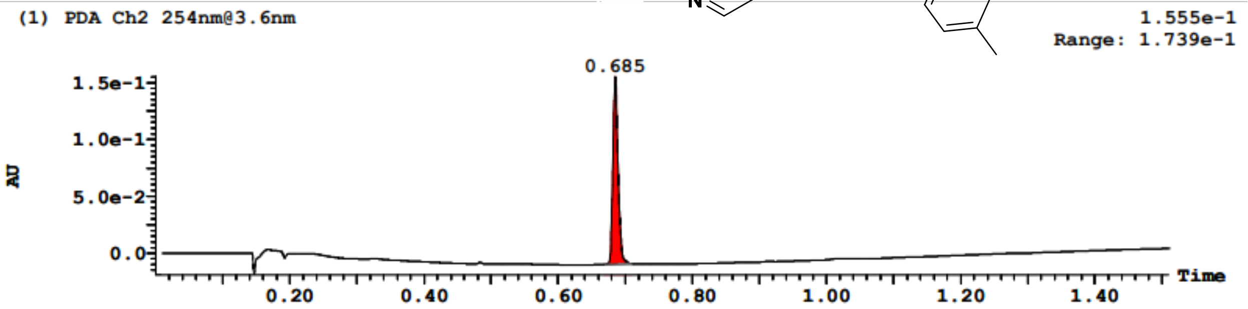

(1) PDA Ch2 254nm@3.6nm
1.555e-1
Range: 1.739e-1
0.685
1.5e-1
1.0e-1
5.0e-2
0.0
AU
0.20
0.40
0.60
0.80
1.00
1.20
1.40
Time

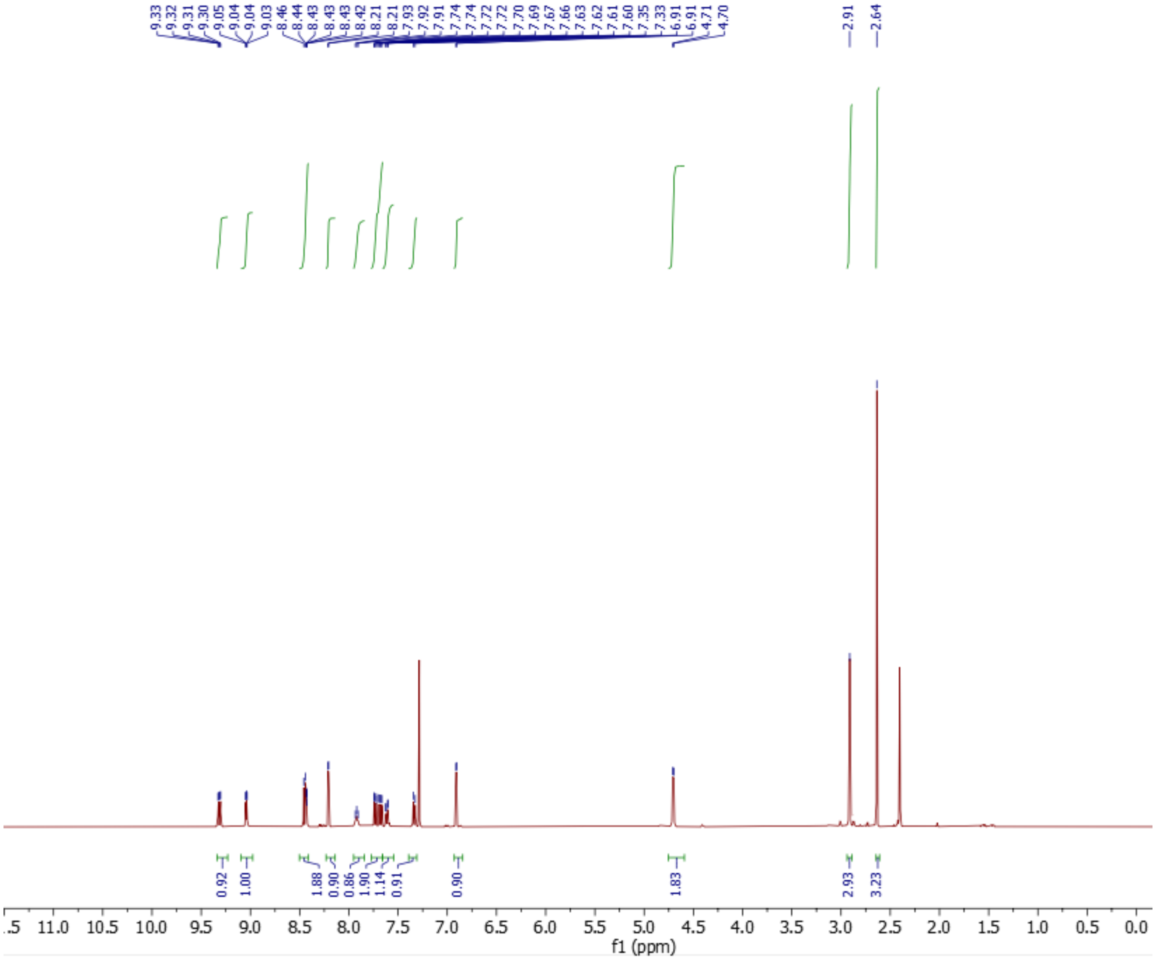

9.33 9.32 9.31 9.30 9.05 9.04 9.04 9.03 8.46 8.44 8.43 8.43 8.43 8.42 8.21 8.21 7.93 7.92 7.91 7.74 7.74 7.72 7.72 7.70 7.69 7.67 7.66 7.63 7.62 7.61 7.60 7.35 7.33 6.91 6.91 4.71 4.70
2.91
2.64
0.92
1.00
1.88
0.90
0.86
1.90
1.14
0.91
0.90
1.83
2.93
3.23
f1 (ppm)

(9n)

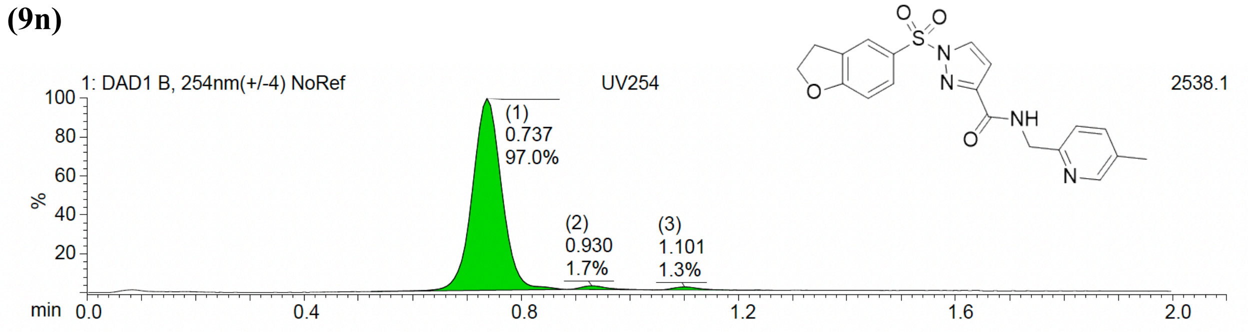
1: DAD1 B, 254nm(+/-4) NoRef
UV254
2538.1
(1)
0.737
97.0%
(2)
0.930
1.7%
(3)
1.101
1.3%
%
min

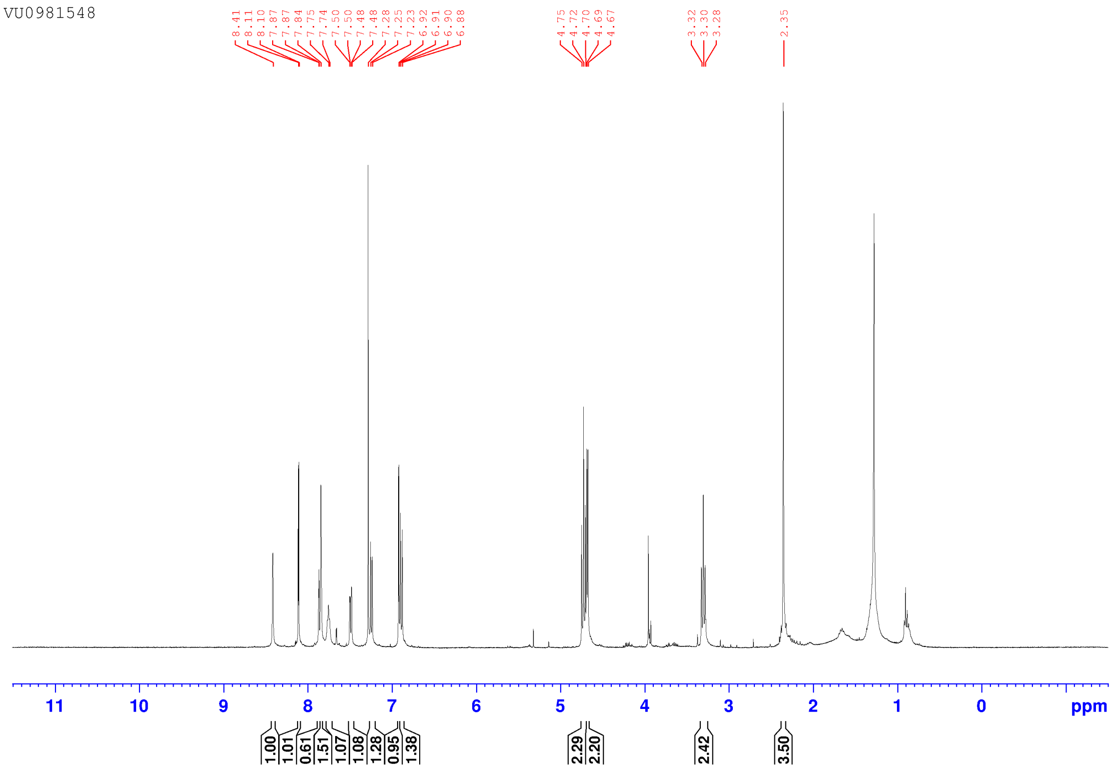
VU0981548
8.41 8.11 8.10 7.87 7.84 7.75 7.74 7.50 7.48 7.48 7.28 7.25 7.23 6.92 6.91 6.90 6.88
4.75 4.72 4.70 4.69 4.67
3.32 3.30 3.28
2.35
ppm
1.00 1.01 0.61 1.51 1.07 1.08 1.28 0.95 1.38
2.29 2.20
2.42
3.50

(9o)

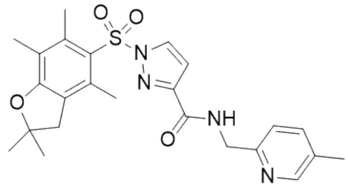

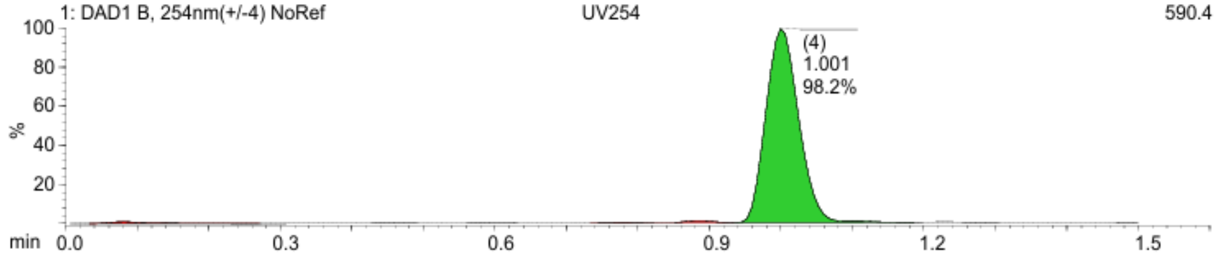

1: DAD1 B, 254nm(+/-4) NoRef
UV254
590.4
(4)
1.001
98.2%
%
min

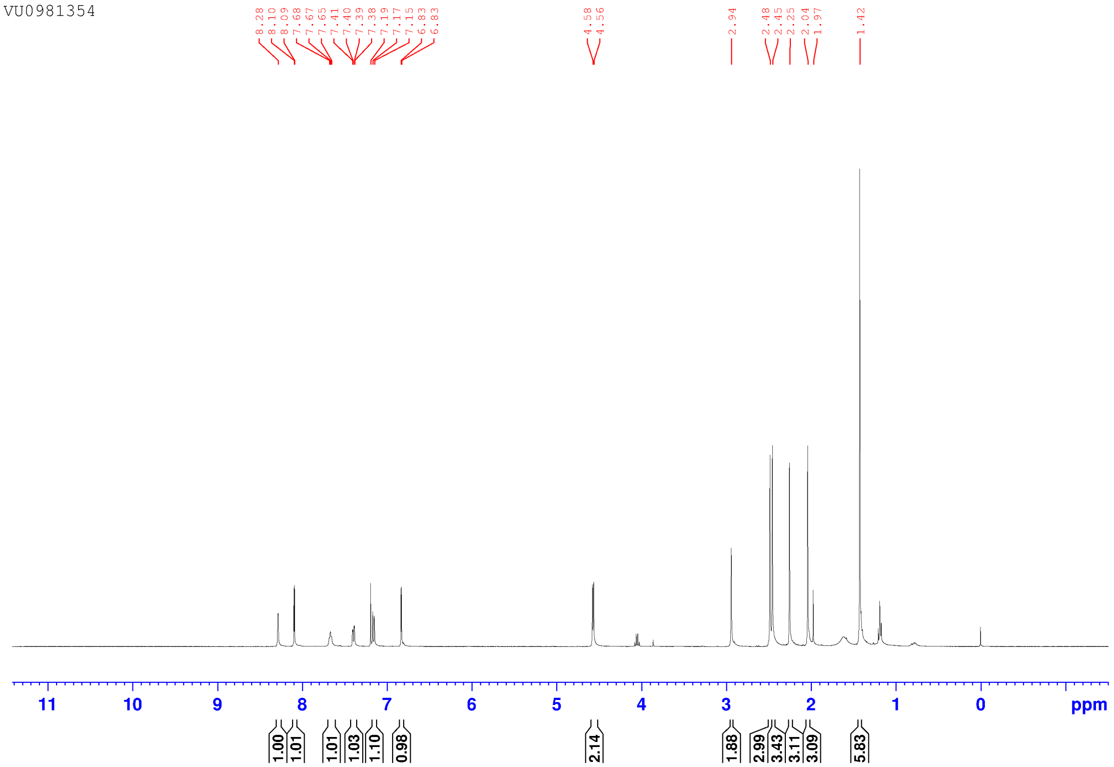

VU0981354
8.28 8.10 8.09 7.68 7.67 7.65 7.41 7.40 7.39 7.38 7.19 7.17 7.15 6.83 6.83
4.58 4.56
2.94
2.48 2.45 2.25 2.04 1.97
1.42
ppm
1.00 1.01 1.01 1.03 1.10 0.98 2.14 1.88 2.99 3.43 3.11 3.09 5.83

(10a)

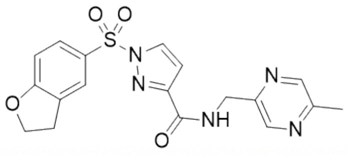
O
O
S
N
N
O
NH
N
N
O

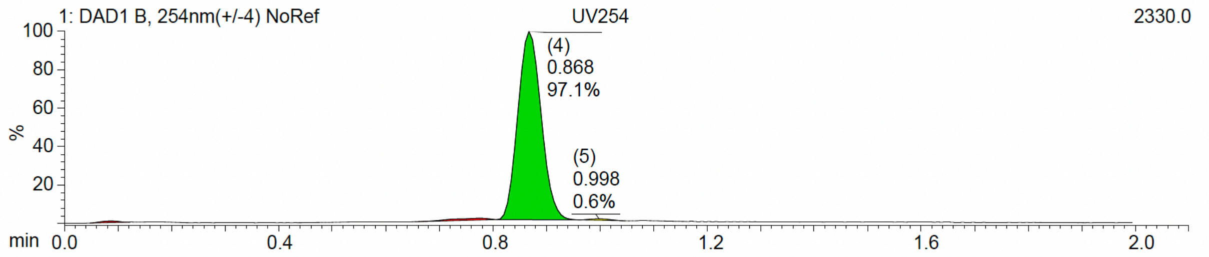
1: DAD1 B, 254nm(+/-4) NoRef
UV254
2330.0
(4)
0.868
97.1%
(5)
0.998
0.6%
%
min
100
80
60
40
20
0.0
0.4
0.8
1.2
1.6
2.0

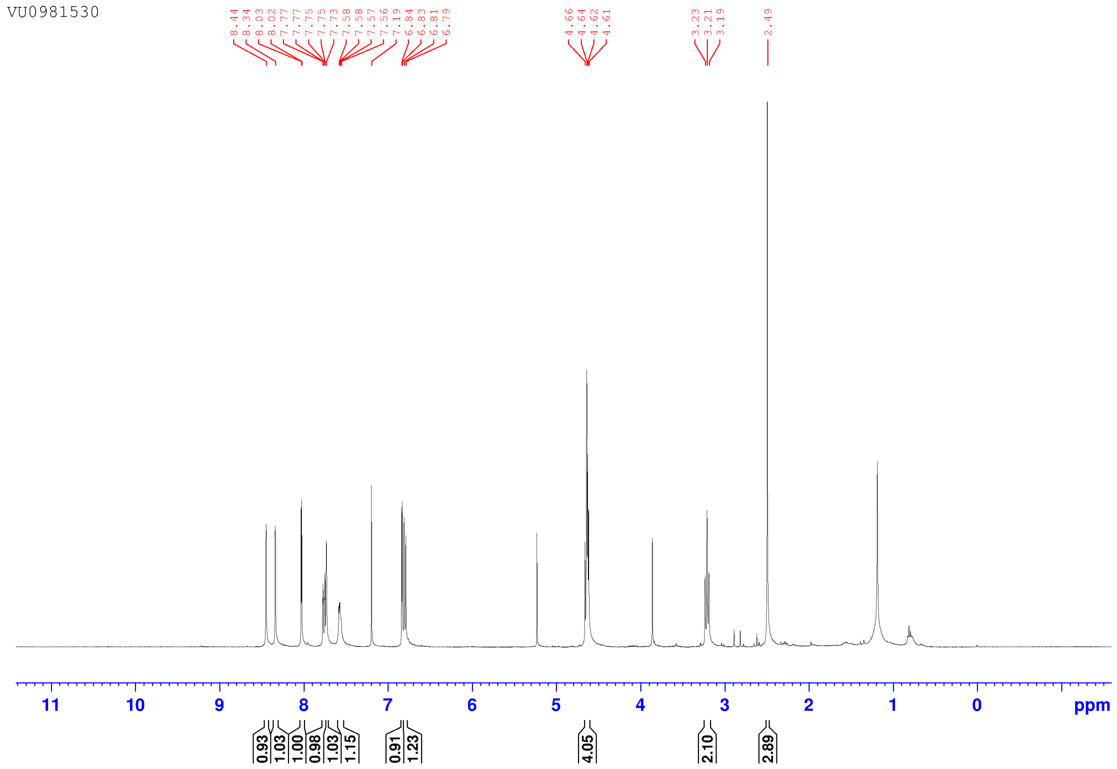
VU0981530
8.44
8.34
8.03
8.02
7.77
7.77
7.75
7.75
7.73
7.58
7.58
7.57
7.56
7.19
6.84
6.83
6.81
6.79
4.66
4.64
4.62
4.61
3.23
3.21
3.19
2.49
11
10
9
8
7
6
5
4
3
2
1
0
ppm
0.93
1.03
1.00
0.98
1.03
1.15
0.91
1.23
4.05
2.10
2.89

(10b)

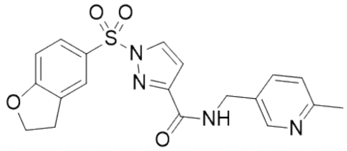

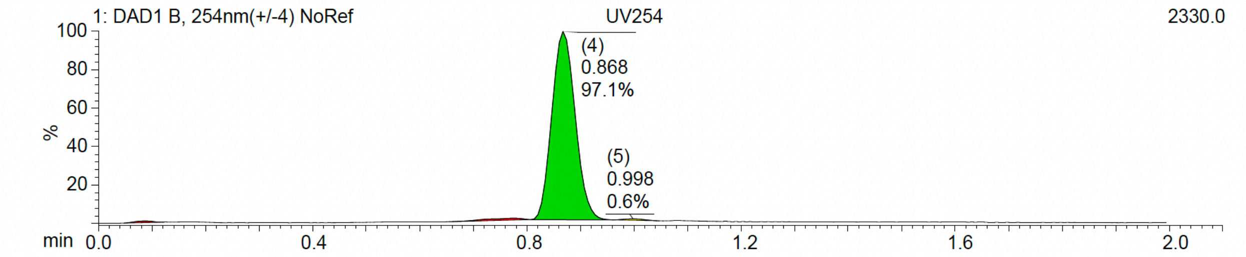
1: DAD1 B, 254nm(+/-4) NoRef
UV254
2330.0
(4)
0.868
97.1%
(5)
0.998
0.6%
%
min

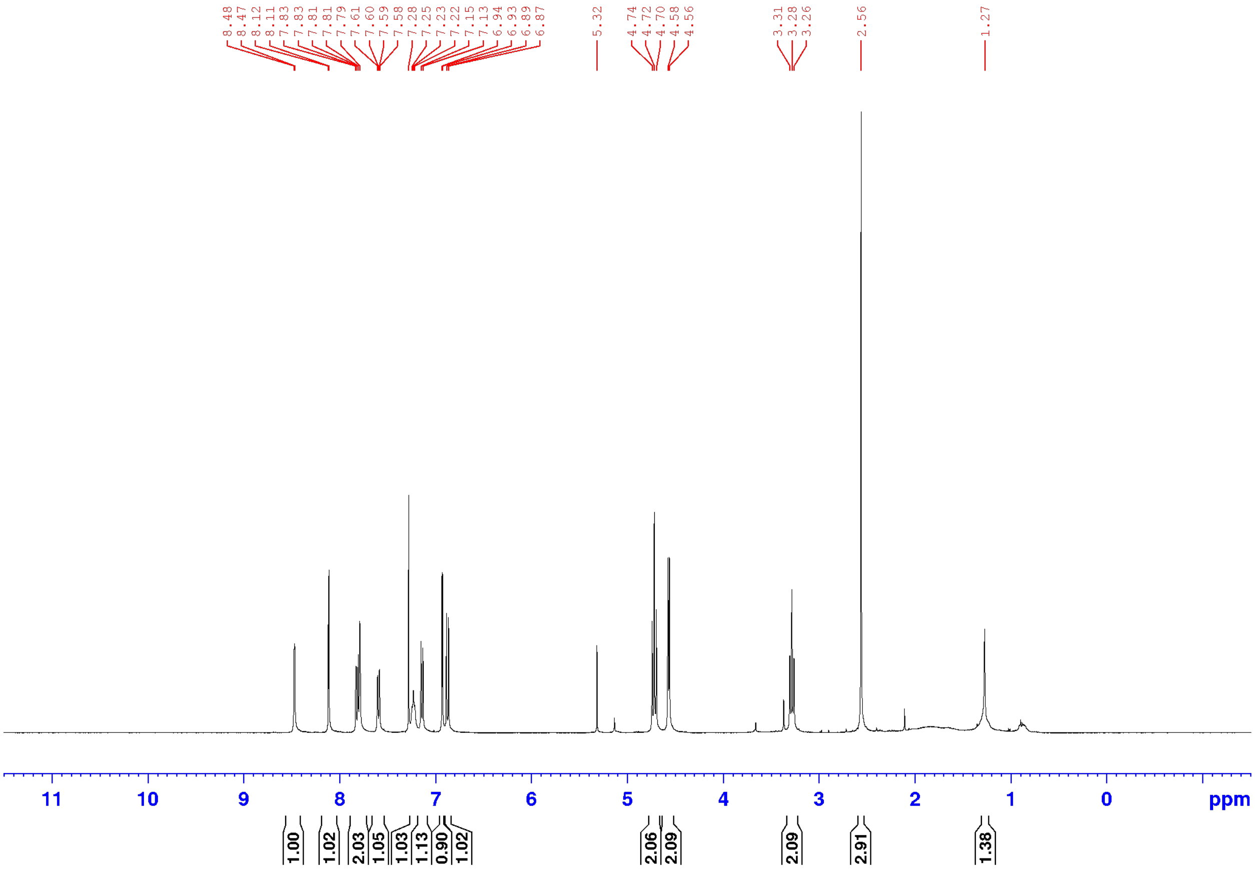
8.48
8.47
8.12
8.11
7.83
7.83
7.81
7.79
7.61
7.60
7.59
7.58
7.28
7.25
7.23
7.22
7.15
7.13
6.94
6.93
6.89
6.87
5.32
4.74
4.72
4.70
4.58
4.56
3.31
3.28
3.26
2.56
1.27
ppm
1.00
1.02
2.03
1.05
1.03
1.13
0.90
1.02
2.06
2.09
2.09
2.91
1.38

(10c)

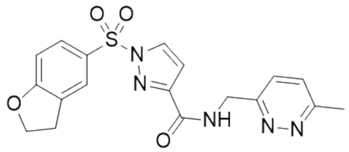
O
S
N
N
O
O
NH
O
N
N

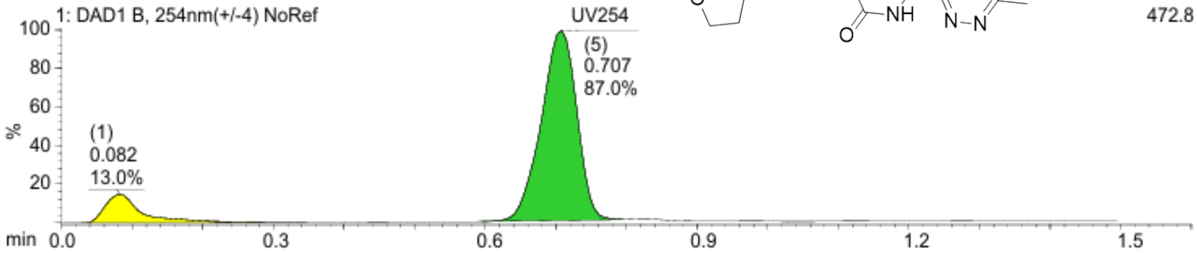
1: DAD1 B, 254nm(+/-4) NoRef
UV254
472.8
100
80
60
40
20
%
min
0.0
0.3
0.6
0.9
1.2
1.5
(1)
0.082
13.0%
(5)
0.707
87.0%

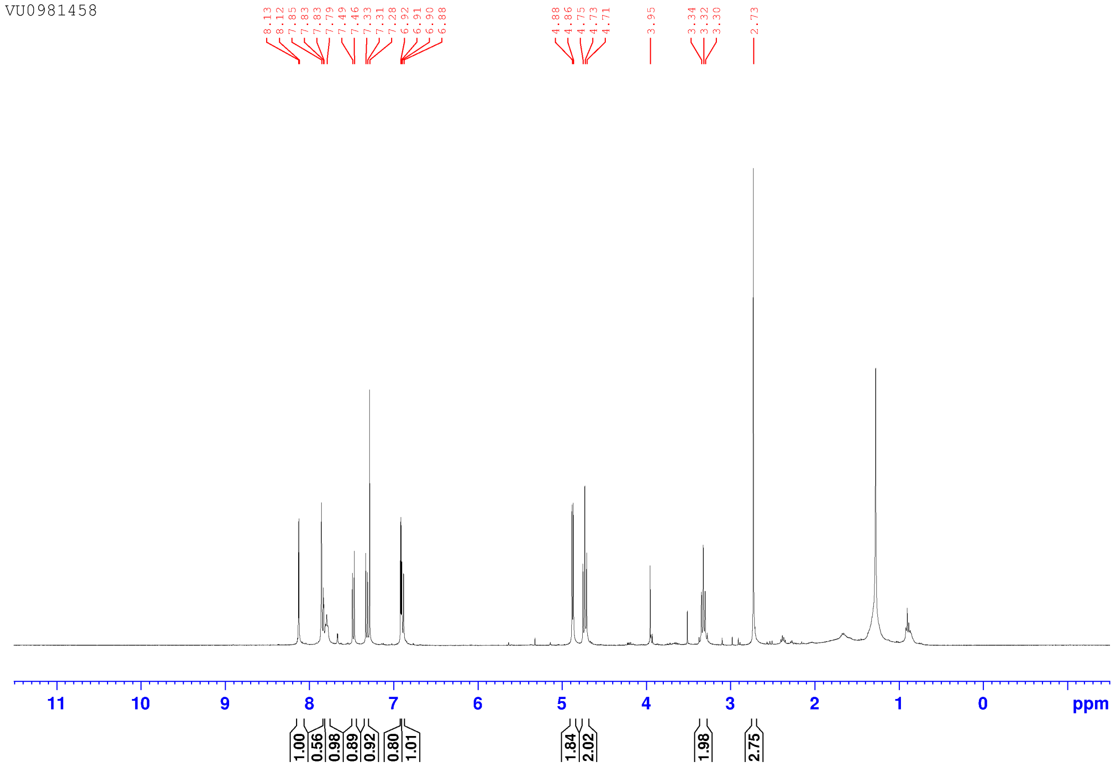
VU0981458
8.13
8.12
7.85
7.83
7.83
7.79
7.49
7.46
7.33
7.31
7.28
6.92
6.91
6.90
6.88
4.88
4.86
4.75
4.73
4.71
3.95
3.34
3.32
3.30
2.73
11
10
9
8
7
6
5
4
3
2
1
0
ppm
1.00
0.56
0.98
0.89
0.92
0.80
1.01
1.84
2.02
1.98
2.75

(10d)

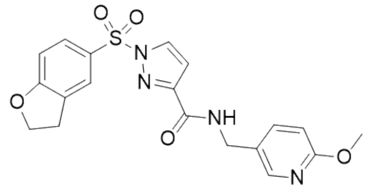

O
S
O
N
N
O
NH
O
N
O

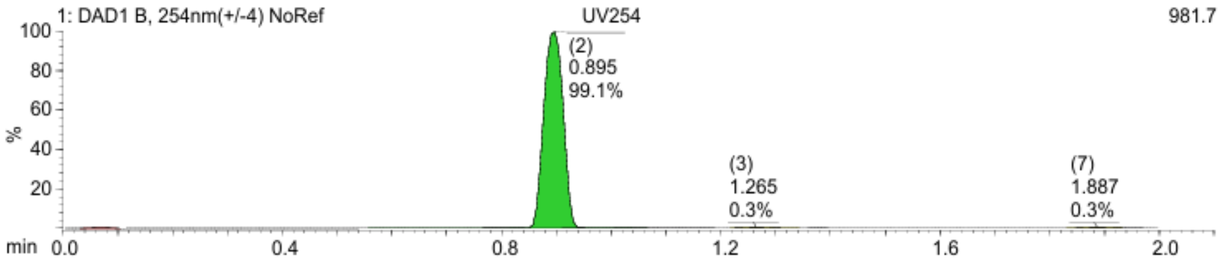

1: DAD1 B, 254nm(+/-4) NoRef
UV254
981.7
(2)
0.895
99.1%
(3)
1.265
0.3%
(7)
1.887
0.3%
%
min

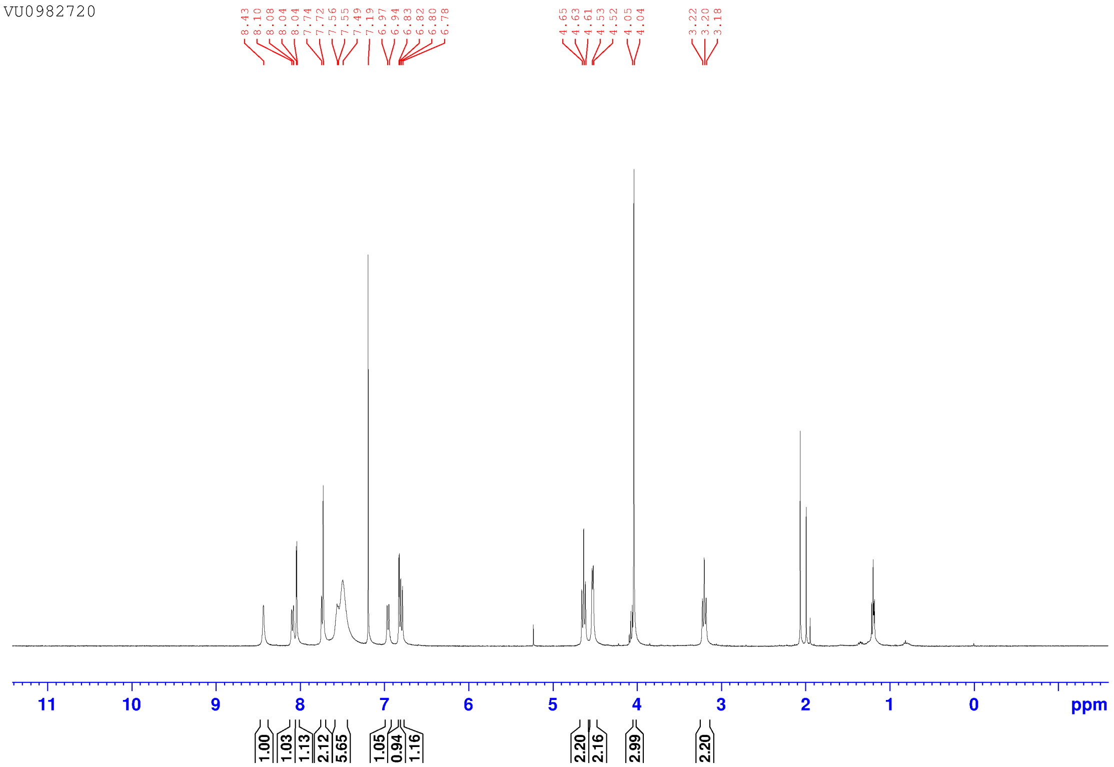

VU0982720
8.43 8.10 8.08 8.04 8.04 7.74 7.72 7.56 7.55 7.49 7.19 6.97 6.94 6.83 6.82 6.80 6.78
4.65 4.63 4.61 4.53 4.52 4.05 4.04
3.22 3.20 3.18
ppm
1.00 1.03 1.13 2.12 5.65 1.05 0.94 1.16 2.20 2.16 2.99 2.20

(10e)

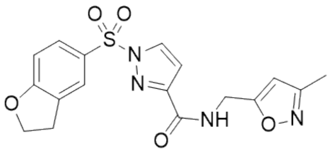

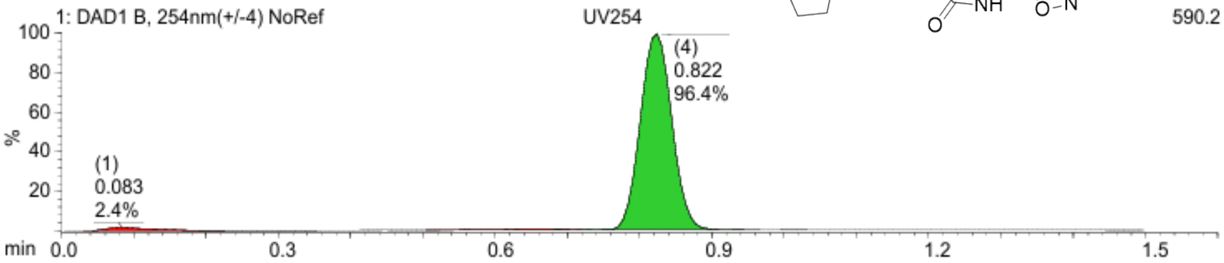
1: DAD1 B, 254nm(+/-4) NoRef
UV254
590.2
(1)
0.083
2.4%
(4)
0.822
96.4%
%
min
100
80
60
40
20
0.0
0.3
0.6
0.9
1.2
1.5

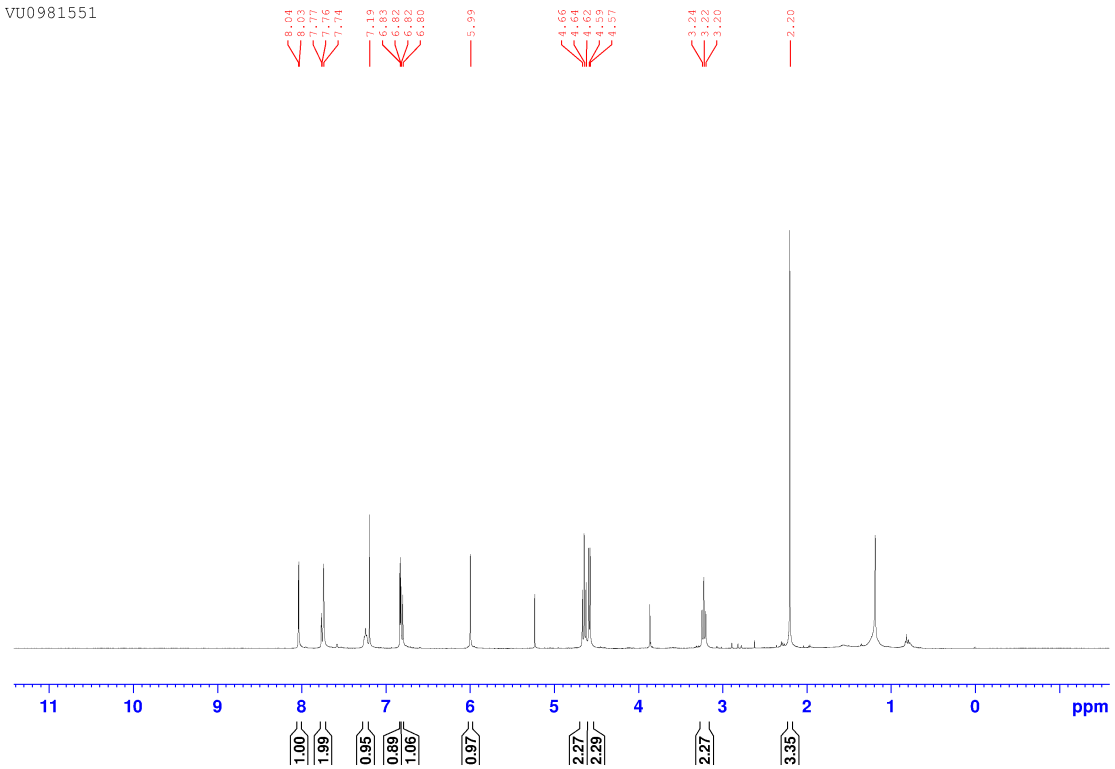
VU0981551
8.04
8.03
7.77
7.76
7.74
7.19
6.83
6.82
6.82
6.80
5.99
4.66
4.64
4.62
4.59
4.57
3.24
3.22
3.20
2.20
11
10
9
8
7
6
5
4
3
2
1
0
ppm
1.00
1.99
0.95
0.89
1.06
0.97
2.27
2.29
2.27
3.35

(10f)

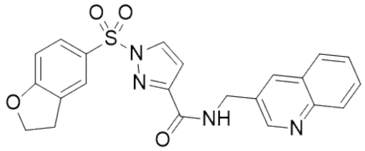

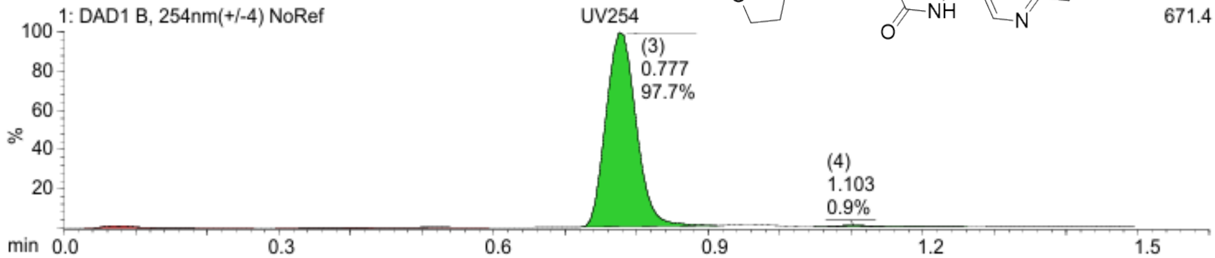
1: DAD1 B, 254nm(+/-4) NoRef
UV254
671.4
(3)
0.777
97.7%
(4)
1.103
0.9%
%
min

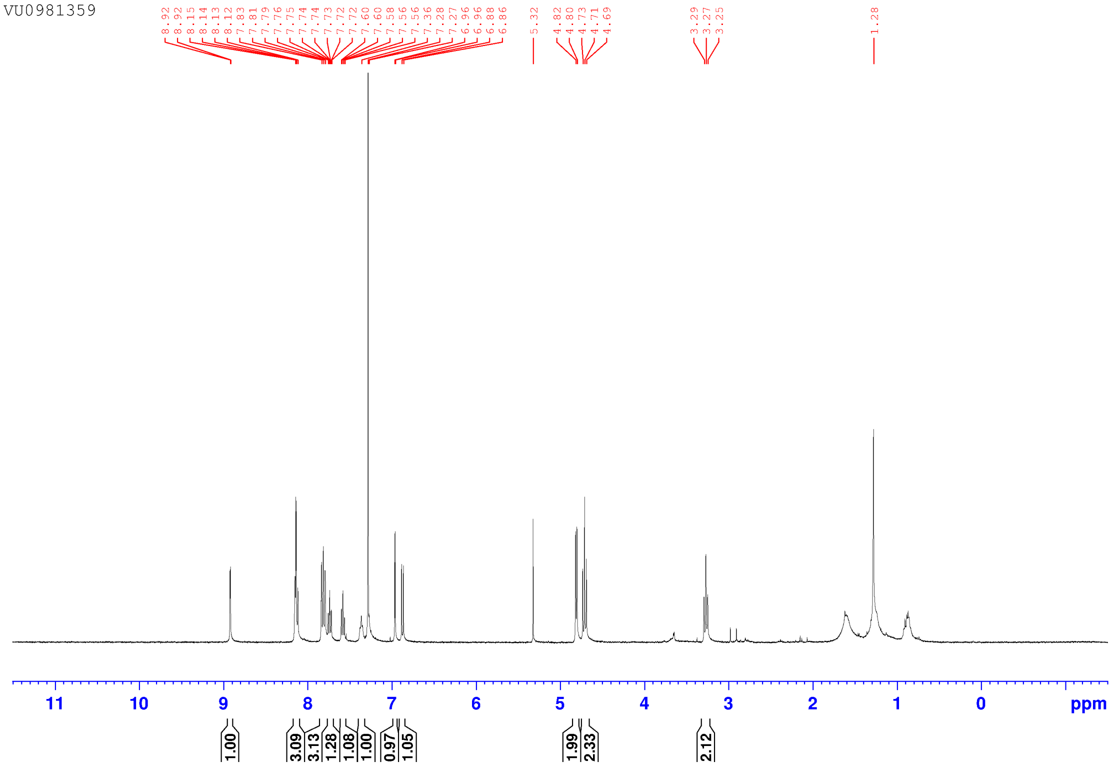
VU0981359
8.92 8.92 8.15 8.14 8.13 8.12 7.83 7.81 7.79 7.76 7.75 7.74 7.74 7.73 7.72 7.72 7.60 7.60 7.58 7.56 7.56 7.36 7.28 7.27 6.96 6.96 6.88 6.86
5.32
4.82 4.80 4.73 4.71 4.69
3.29 3.27 3.25
1.28
ppm
1.00
3.09
3.13
1.28
1.08
1.00
0.97
1.05
1.99
2.33
2.12

(10g)

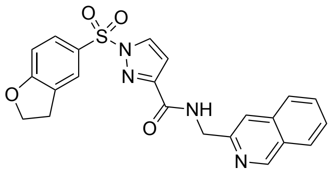

O
O
S
N
N
O
NH
O
N

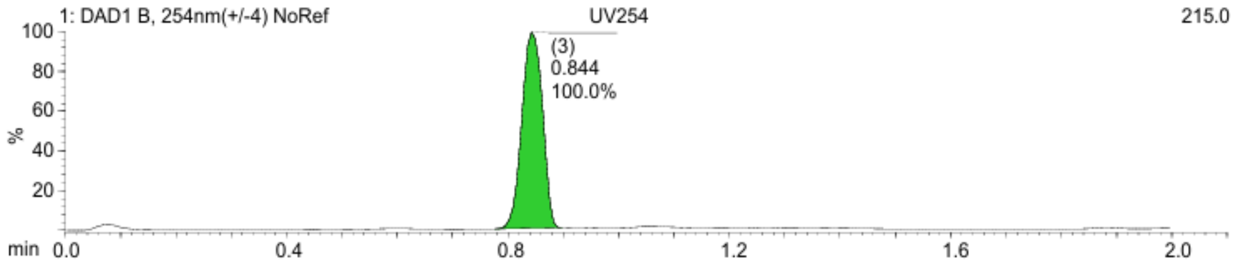

1: DAD1 B, 254nm(+/-4) NoRef
UV254
215.0
(3)
0.844
100.0%
%
min

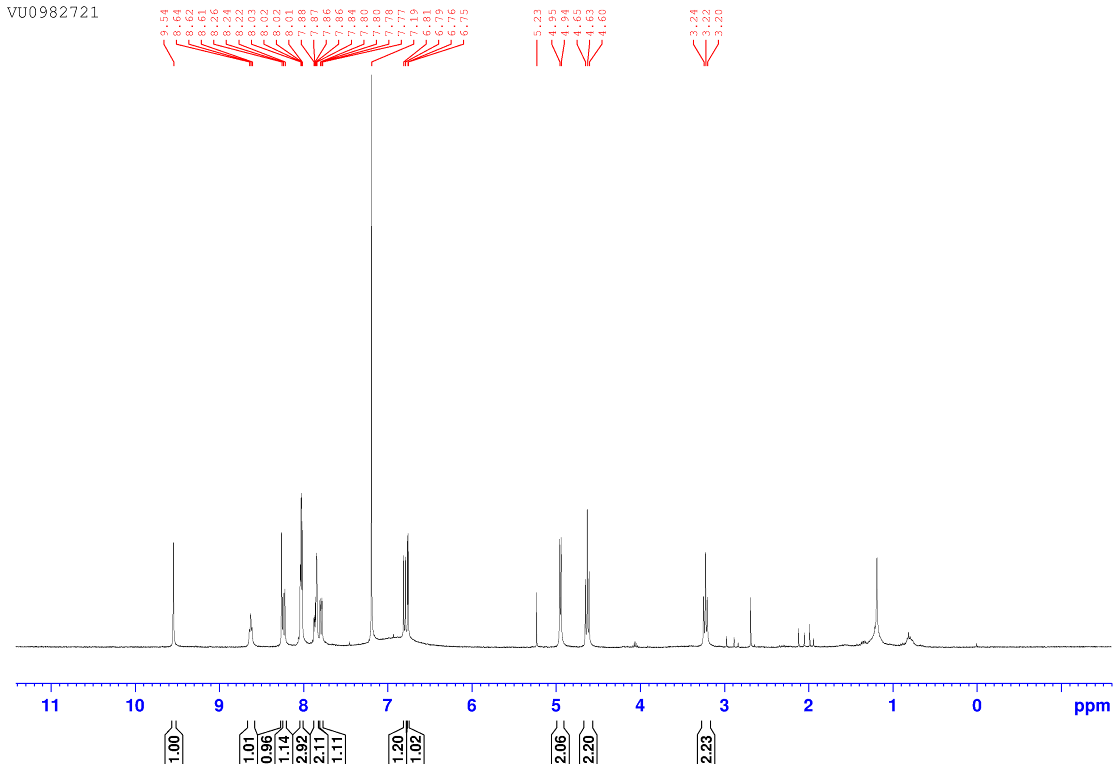

VU0982721
9.54 8.64 8.62 8.61 8.26 8.24 8.22 8.03 8.02 8.01 7.88 7.87 7.86 7.86 7.84 7.80 7.80 7.78 7.77 7.19 6.81 6.79 6.76 6.75
5.23 4.95 4.94 4.65 4.63 4.60
3.24 3.22 3.20
ppm
1.00 1.01 0.96 1.14 2.92 2.11 1.11 1.20 1.02 2.06 2.20 2.23

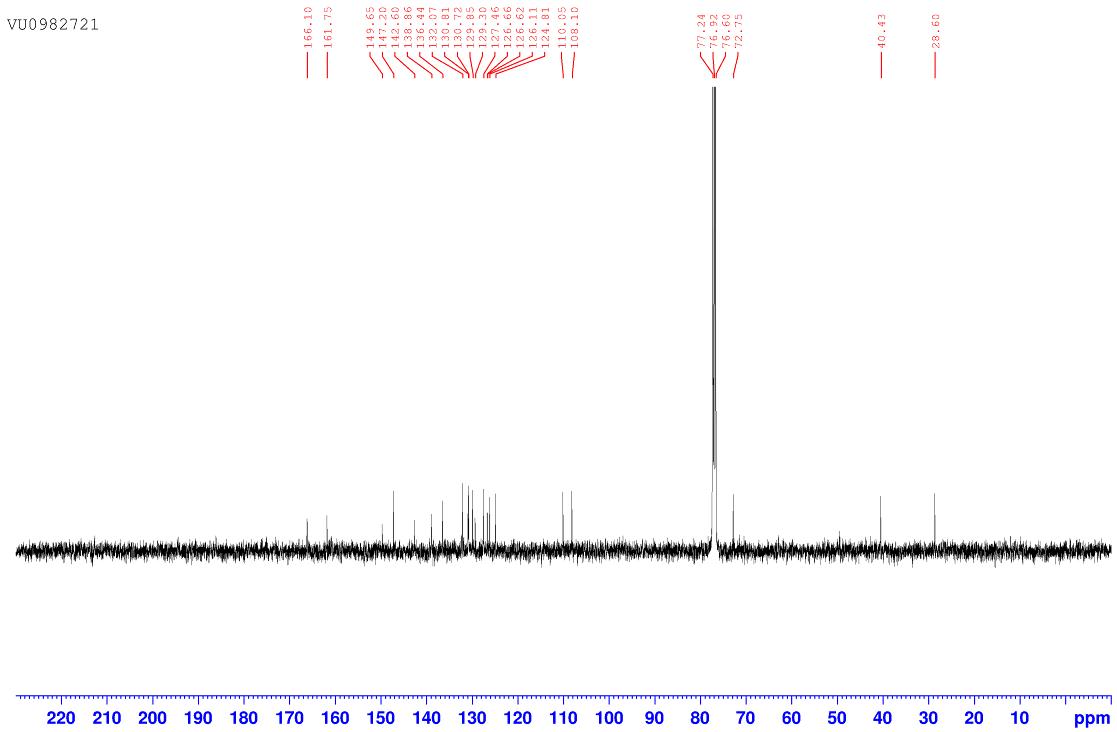
VU0982721
166.10
161.75
149.65
147.20
142.60
138.86
136.44
132.07
130.81
130.72
129.85
129.30
127.46
126.66
126.62
126.11
124.81
110.05
108.10
77.24
76.92
76.60
72.75
40.43
28.60
220
210
200
190
180
170
160
150
140
130
120
110
100
90
80
70
60
50
40
30
20
10
ppm

(10h)

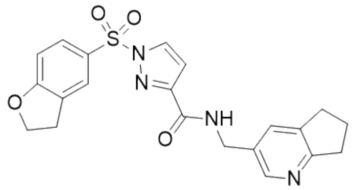

O
O
S
N
N
NH
O
O
N

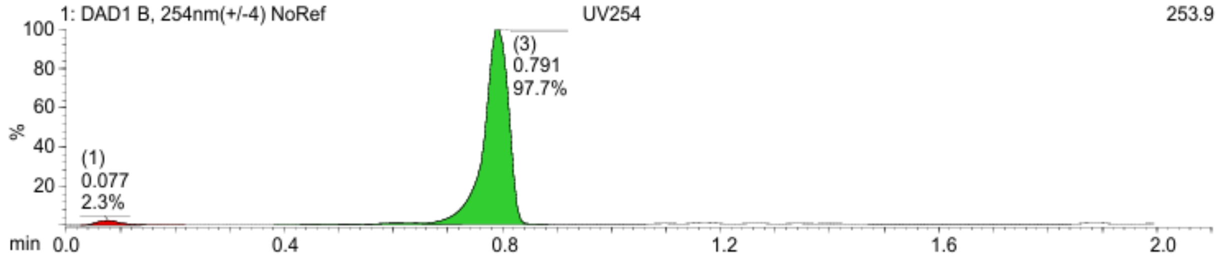

1: DAD1 B, 254nm(+/-4) NoRef
UV254
253.9
100
80
60
40
20
%
(3)
0.791
97.7%
(1)
0.077
2.3%
min 0.0
0.4
0.8
1.2
1.6
2.0

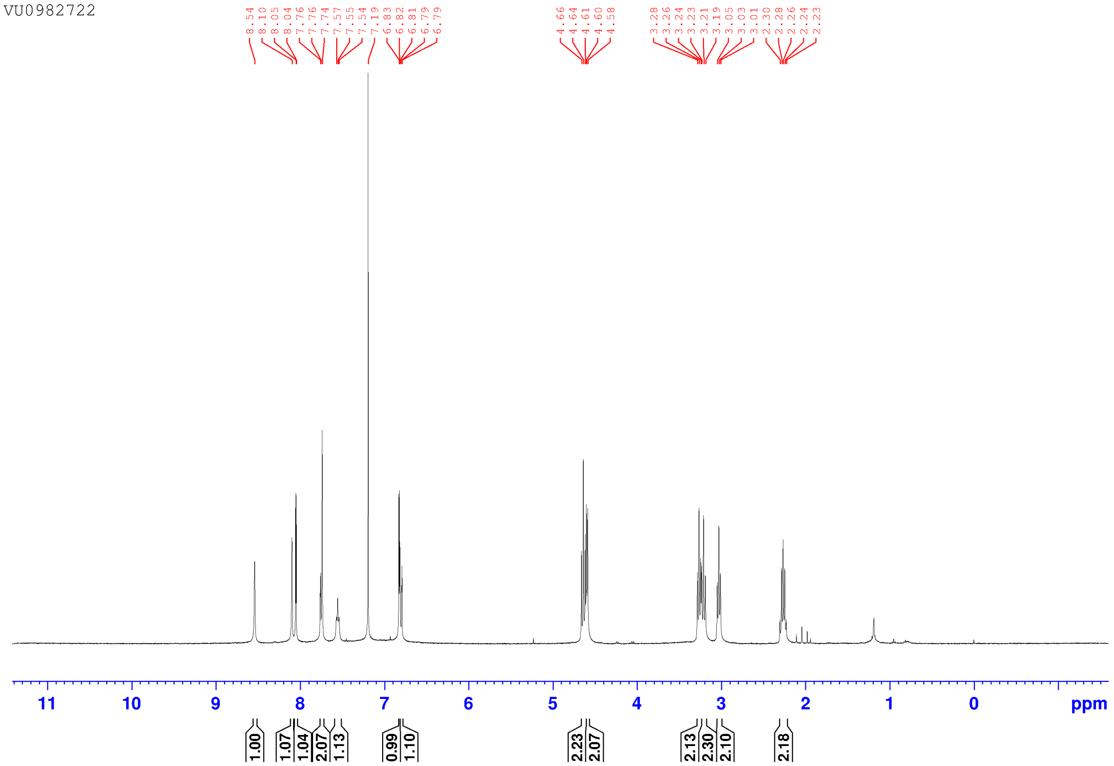

VU0982722
8.54 8.10 8.05 8.04 7.76 7.74 7.57 7.55 7.54 7.19 6.83 6.82 6.81 6.79 6.79
4.66 4.64 4.61 4.60 4.58
3.28 3.26 3.24 3.23 3.21 3.19 3.05 3.03 3.01 2.30 2.28 2.26 2.24 2.23
11 10 9 8 7 6 5 4 3 2 1 0 ppm
1.00 1.07 1.04 2.07 1.13 0.99 1.10 2.23 2.07 2.13 2.30 2.10 2.18

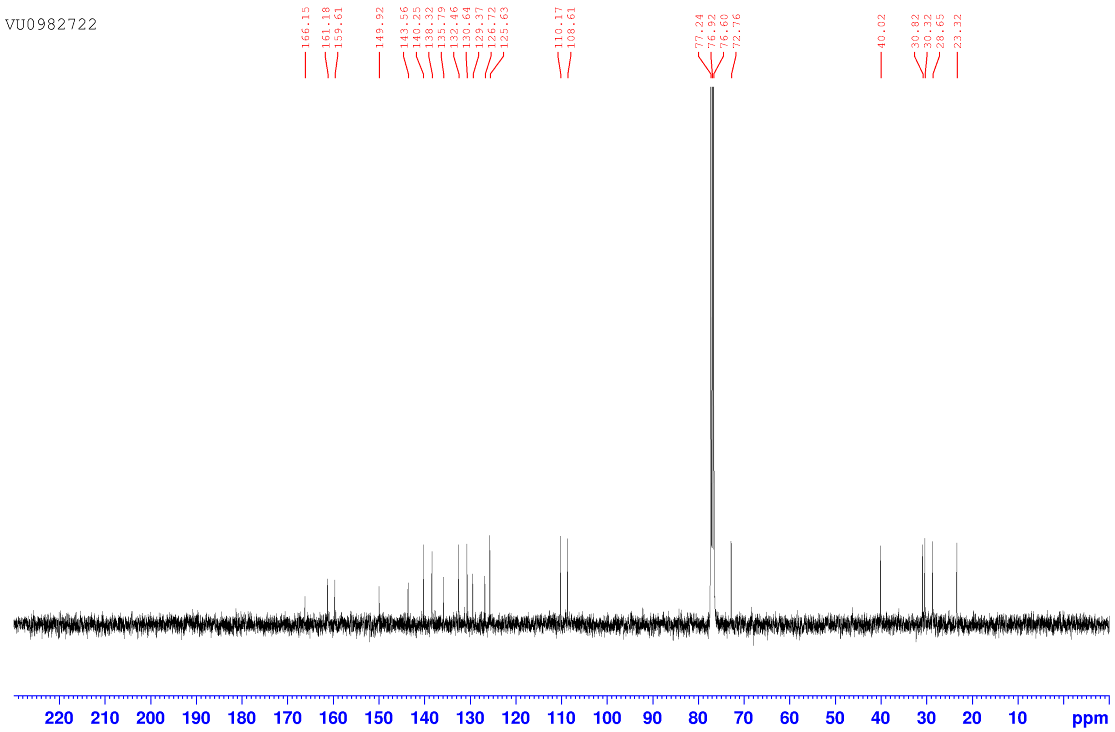
VU0982722
166.15
161.18
159.61
149.92
143.56
140.25
138.32
135.79
132.46
130.64
129.37
126.72
125.63
110.17
108.61
77.24
76.92
76.60
72.76
40.02
30.82
30.32
28.65
23.32
220 210 200 190 180 170 160 150 140 130 120 110 100 90 80 70 60 50 40 30 20 10 ppm

(10i)

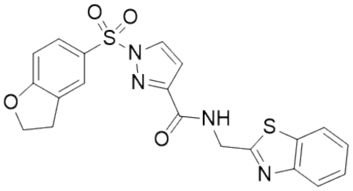

O
O
S
N
N
O
O
NH
S
N

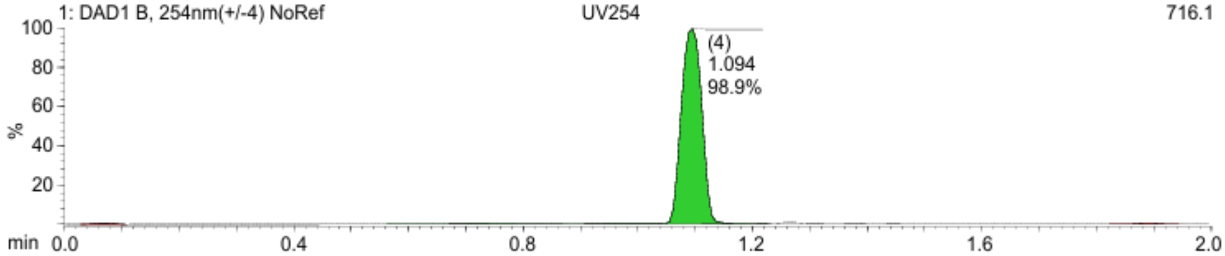

1: DAD1 B, 254nm(+/-4) NoRef
UV254
716.1
100
80
60
40
20
%
(4)
1.094
98.9%
min 0.0
0.4
0.8
1.2
1.6
2.0

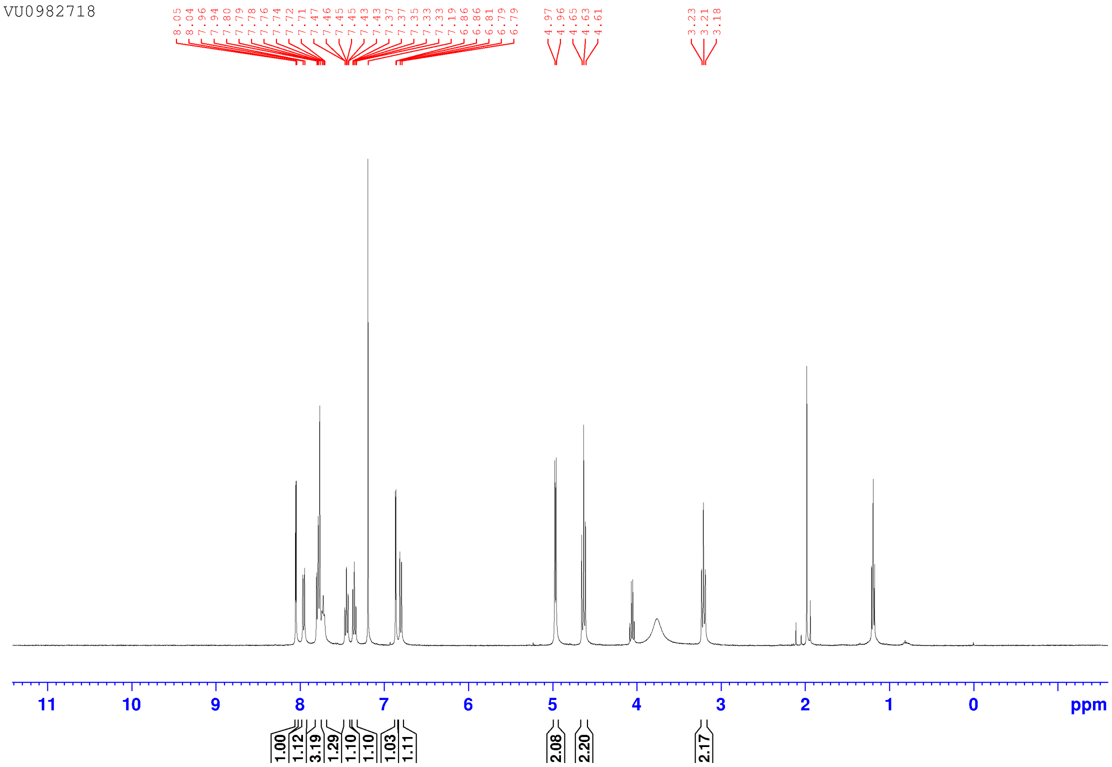

VU0982718
8.05 8.04 7.96 7.94 7.80 7.79 7.78 7.76 7.74 7.72 7.71 7.47 7.46 7.45 7.45 7.43 7.43 7.37 7.35 7.33 7.33 7.19 6.86 6.81 6.79 6.79
4.97 4.96 4.65 4.63 4.61
3.23 3.21 3.18
11 10 9 8 7 6 5 4 3 2 1 0 ppm
1.00 1.12 3.19 1.29 1.10 1.10 1.03 1.11
2.08 2.20
2.17

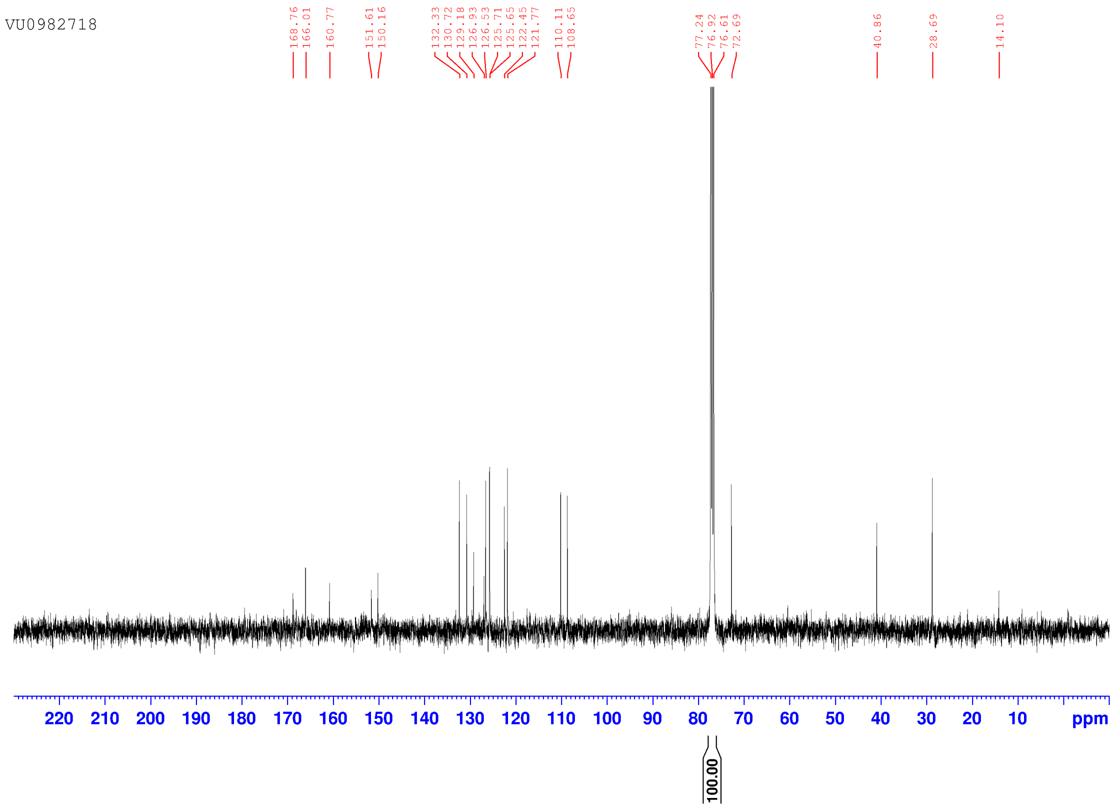
VU0982718
168.76
166.01
160.77
151.61
150.16
132.33
130.72
129.18
126.93
126.53
125.71
125.65
122.45
121.77
110.11
108.65
77.24
76.92
76.61
72.69
40.86
28.69
14.10
220 210 200 190 180 170 160 150 140 130 120 110 100 90 80 70 60 50 40 30 20 10 ppm
100.00

(10j)

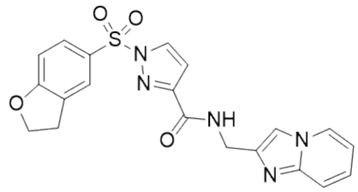

O
O
S
N
N
O
O
NH
N
N

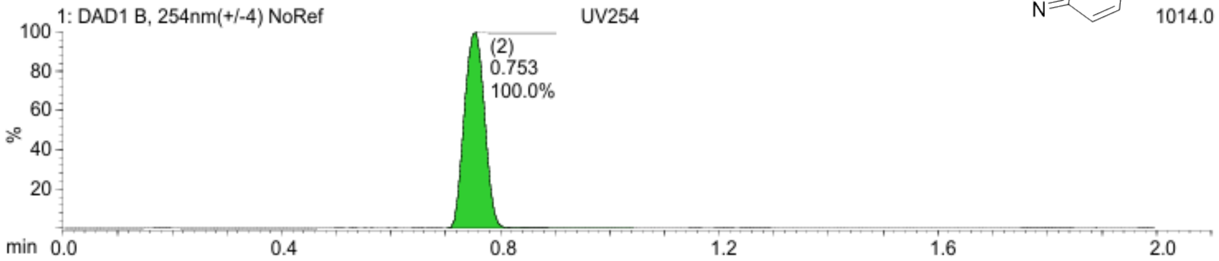

1: DAD1 B, 254nm(+/-4) NoRef
UV254
1014.0
(2)
0.753
100.0%
%
min

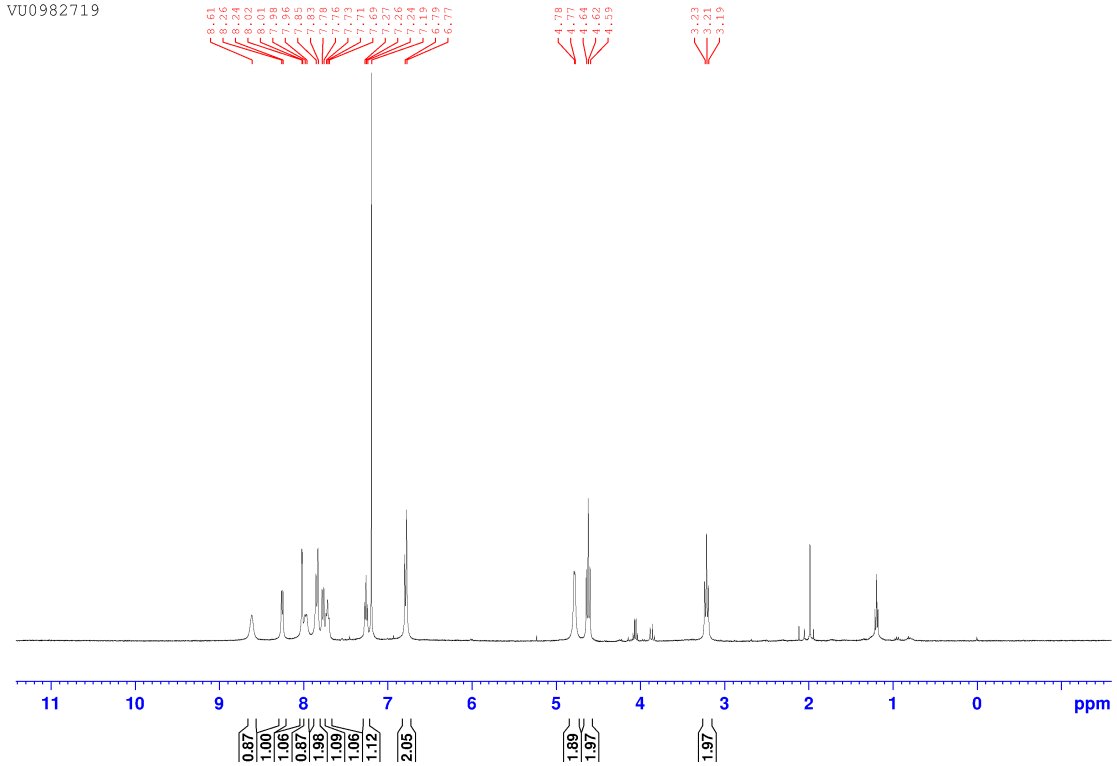

VU0982719
8.61 8.26 8.24 8.02 8.01 7.98 7.96 7.85 7.83 7.78 7.76 7.73 7.71 7.69 7.27 7.26 7.24 7.19 6.79 6.77
4.78 4.77 4.64 4.62 4.59
3.23 3.21 3.19
ppm
0.87 1.00 1.06 0.87 1.98 1.09 1.06 1.12 2.05 1.89 1.97 1.97

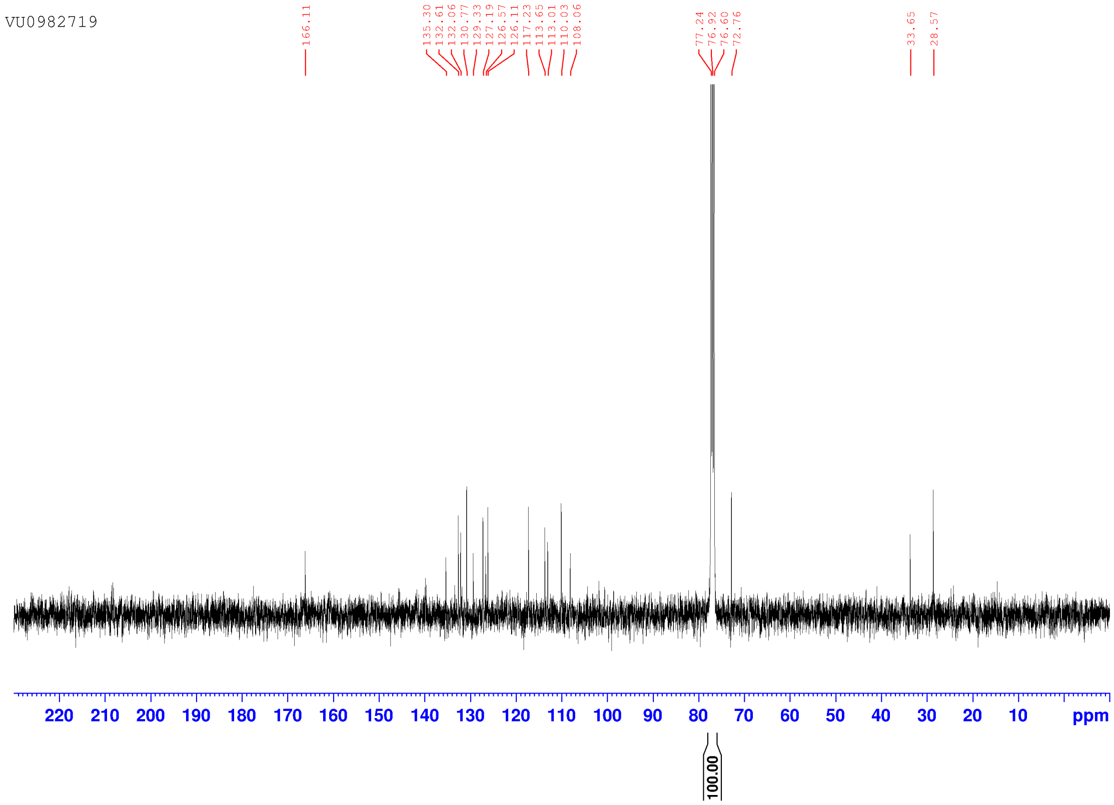

VU0982719
166.11
135.30
132.61
132.06
130.77
129.33
127.19
126.57
126.11
117.23
113.65
113.01
110.03
108.06
77.24
76.92
76.60
72.76
33.65
28.57
220 210 200 190 180 170 160 150 140 130 120 110 100 90 80 70 60 50 40 30 20 10 ppm
100.00

(11a)

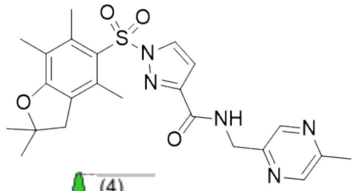

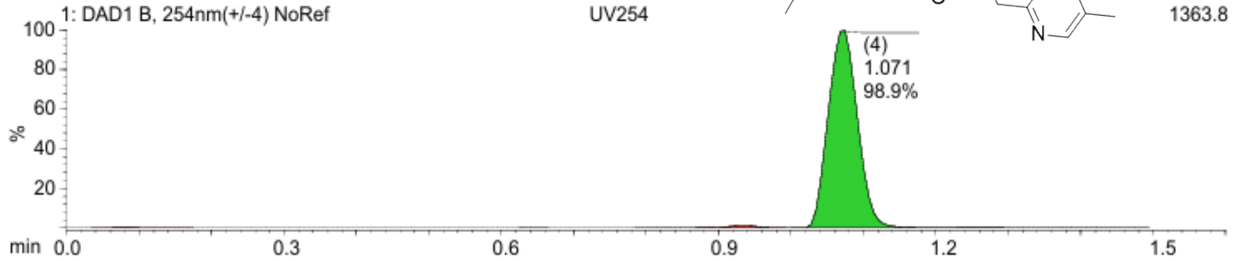

VU0981320

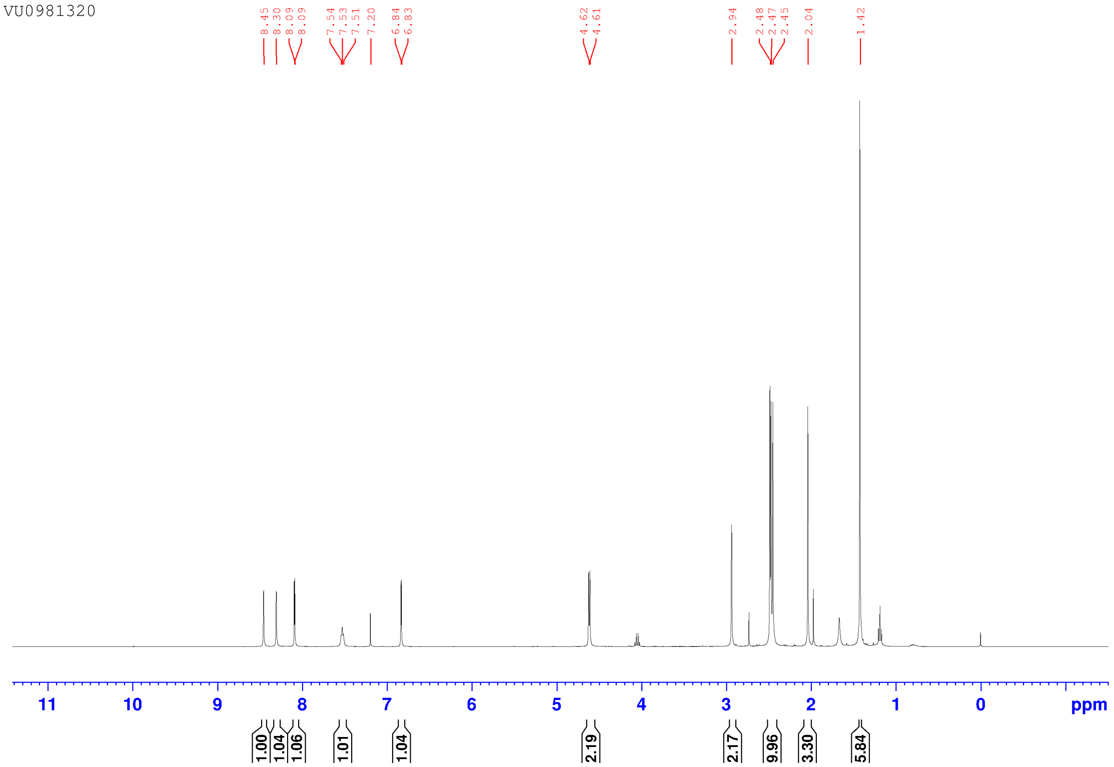

(11b)

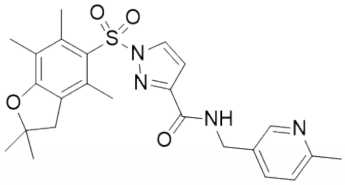
O
O
S
N
N
NH
O
N
O

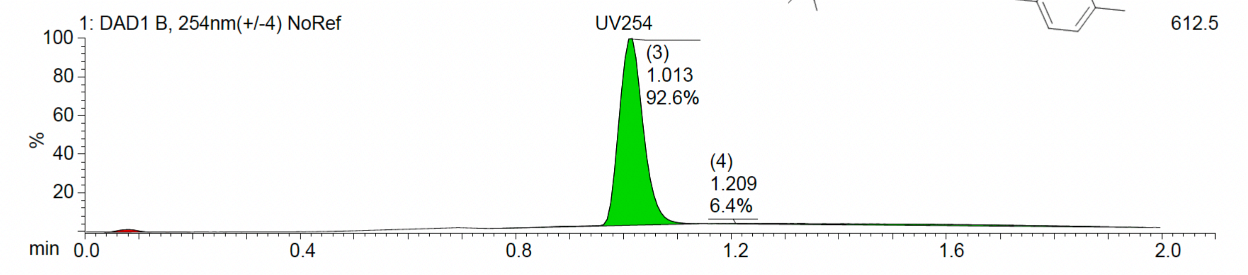
1: DAD1 B, 254nm(+/-4) NoRef
UV254
612.5
(3)
1.013
92.6%
(4)
1.209
6.4%
%
min
0.0
0.4
0.8
1.2
1.6
2.0

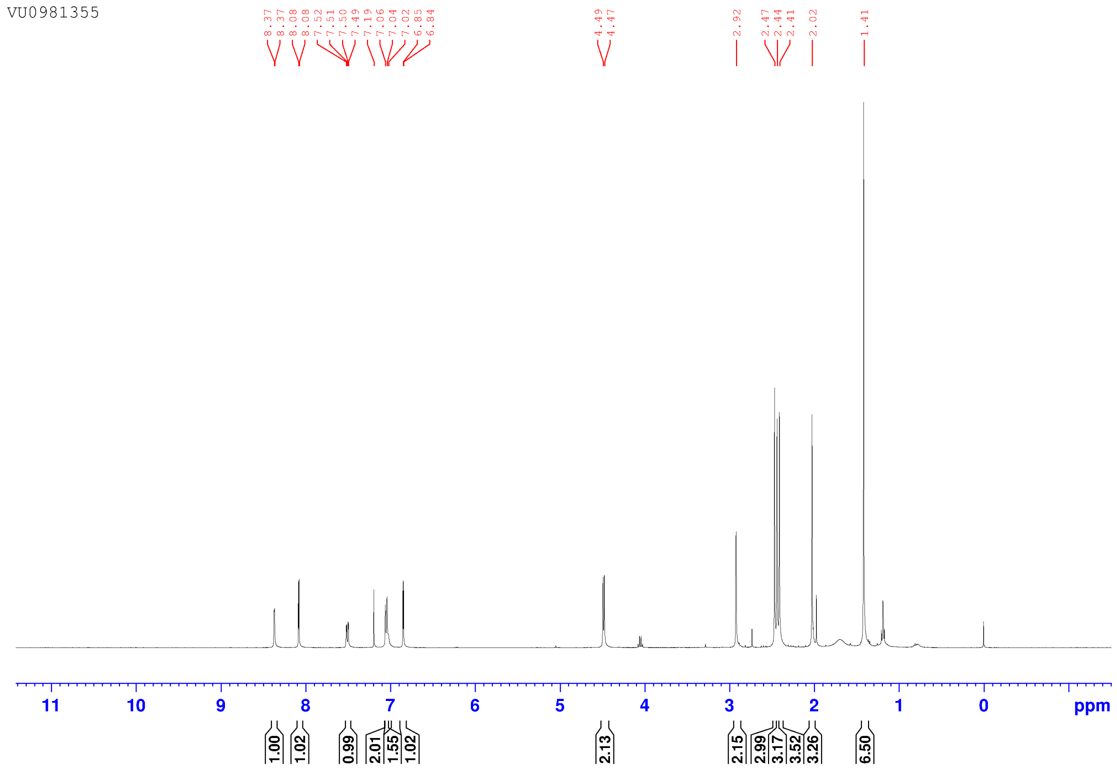
VU0981355
8.37 8.37 8.08 8.08 7.52 7.51 7.50 7.49 7.19 7.06 7.04 7.02 6.85 6.84
4.49 4.47
2.92
2.47 2.44 2.41
2.02
1.41
ppm
1.00 1.02 0.99 2.01 1.55 1.02 2.13 2.15 2.99 3.17 3.52 3.26 6.50

(11c)

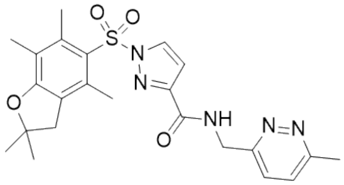

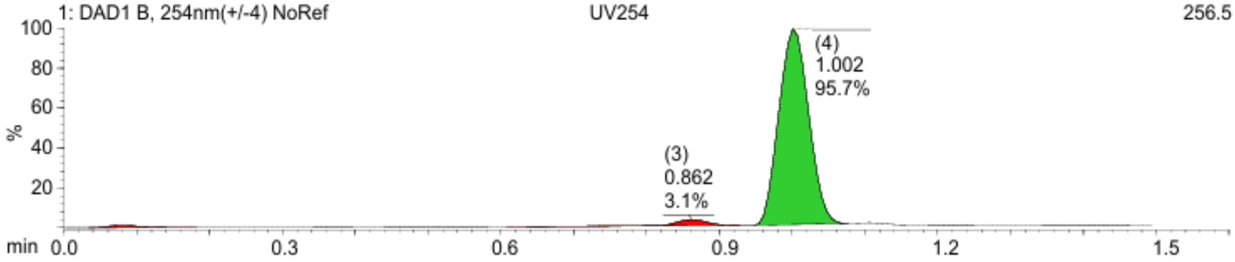

1: DAD1 B, 254nm(+/-4) NoRef
UV254
256.5
(4)
1.002
95.7%
(3)
0.862
3.1%
%
min

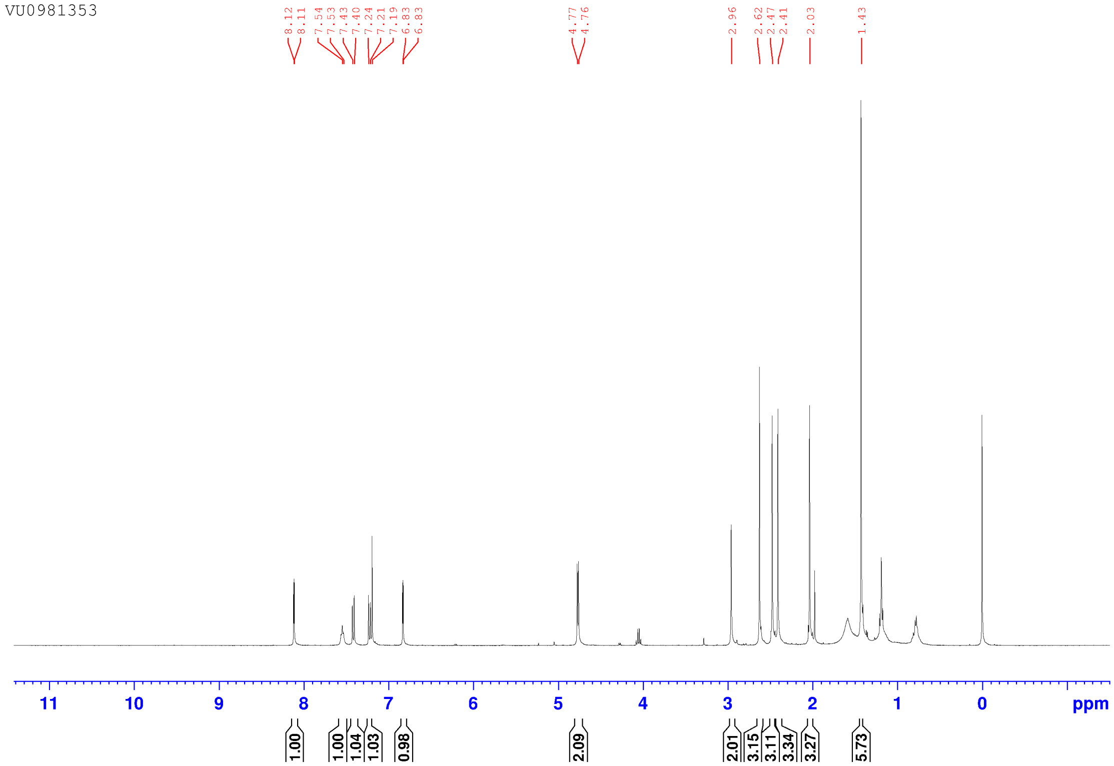

VU0981353
ppm

(11e)

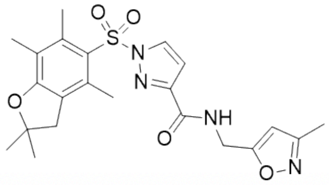

O
S
O
N
N
NH
O
O
N
O

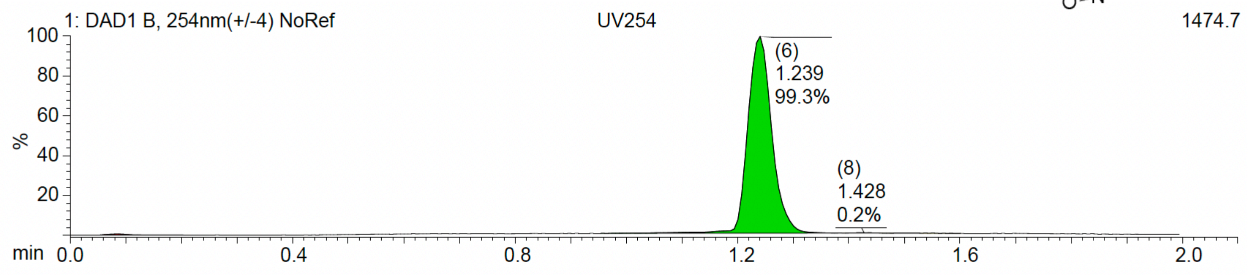

1: DAD1 B, 254nm(+/-4) NoRef
UV254
1474.7
%
(6)
1.239
99.3%
(8)
1.428
0.2%
min

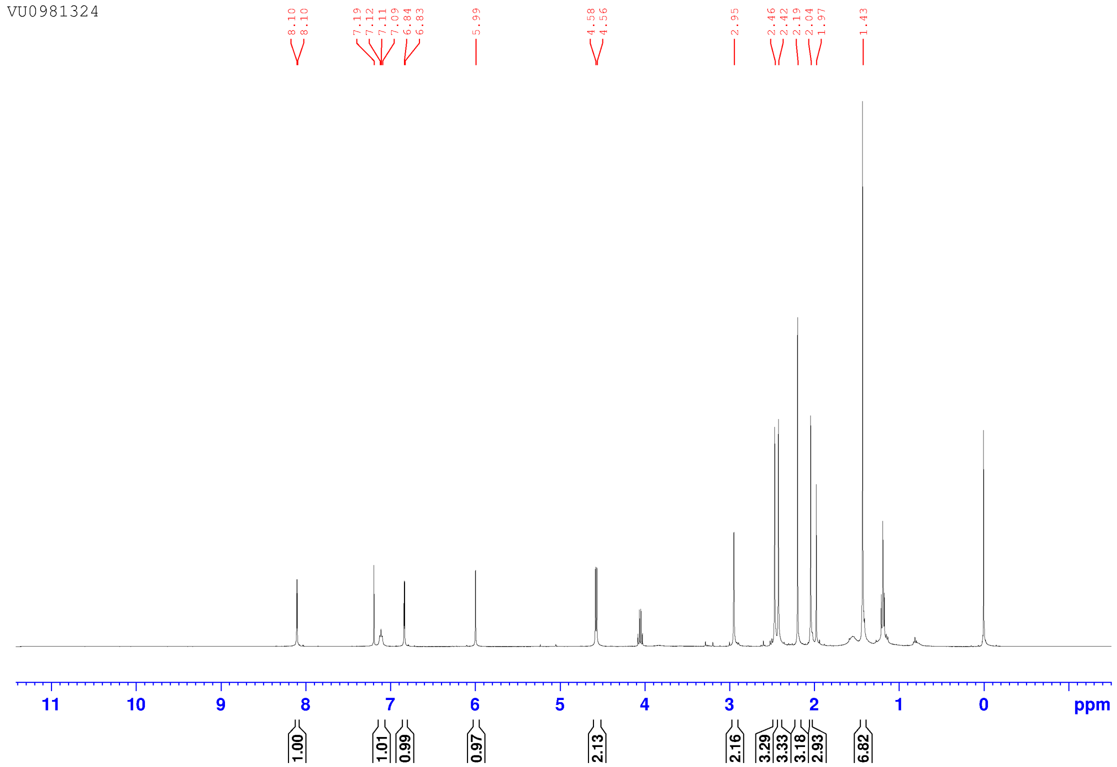

VU0981324
8.10
8.10
7.19
7.12
7.11
7.09
6.84
6.83
5.99
4.58
4.56
2.95
2.46
2.42
2.19
2.04
1.97
1.43
ppm
1.00
1.01
0.99
0.97
2.13
2.16
3.29
3.33
3.18
2.93
6.82

(11f)

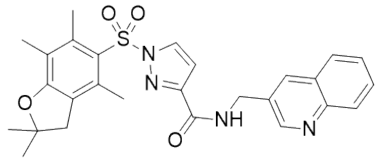

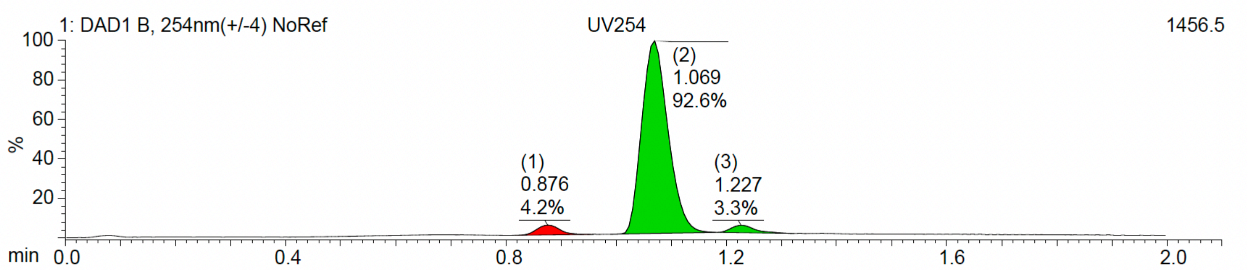
1: DAD1 B, 254nm(+/-4) NoRef
UV254
1456.5
(1)
0.876
4.2%
(2)
1.069
92.6%
(3)
1.227
3.3%
%
min

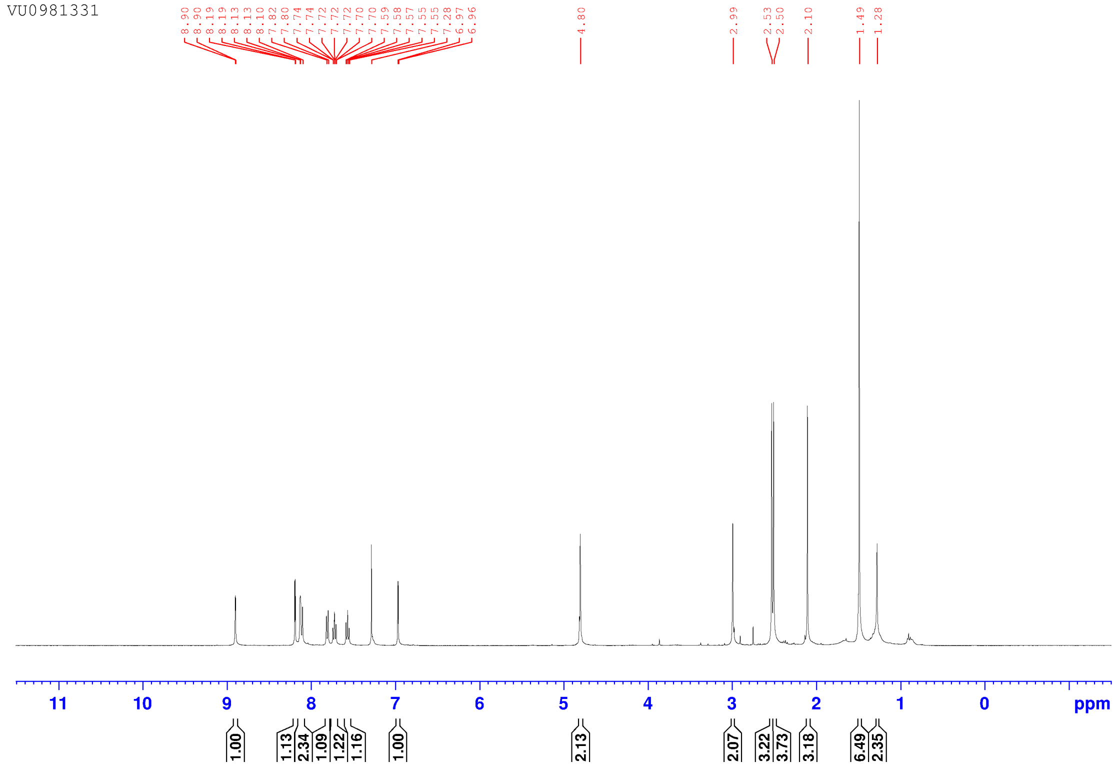
VU0981331
8.90
8.90
8.19
8.19
8.13
8.13
8.10
7.82
7.80
7.74
7.74
7.72
7.72
7.72
7.70
7.70
7.59
7.58
7.57
7.55
7.55
7.28
6.97
6.96
4.80
2.99
2.53
2.50
2.10
1.49
1.28
ppm
1.00
1.13
2.34
1.09
1.22
1.16
1.00
2.13
2.07
3.22
3.73
3.18
6.49
2.35

(11j)

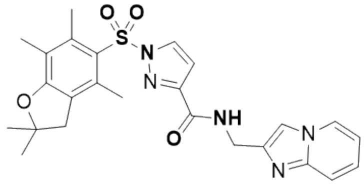

O
O
S
N
N
O
O
NH
N
N

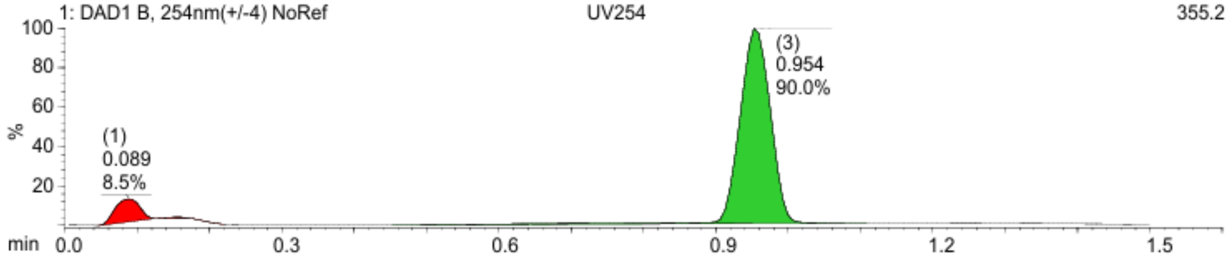

1: DAD1 B, 254nm(+/-4) NoRef
UV254
355.2
%
(1)
0.089
8.5%
(3)
0.954
90.0%
min
0.0
0.3
0.6
0.9
1.2
1.5

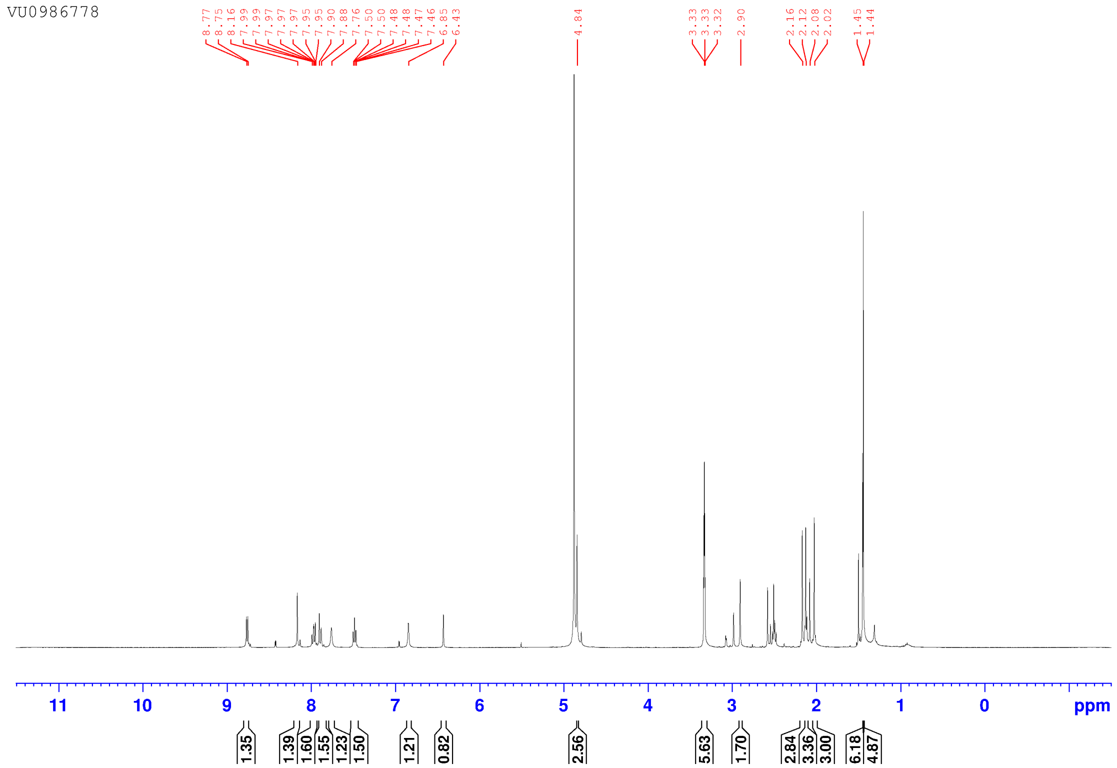

VU0986778
ppm

VU0986778

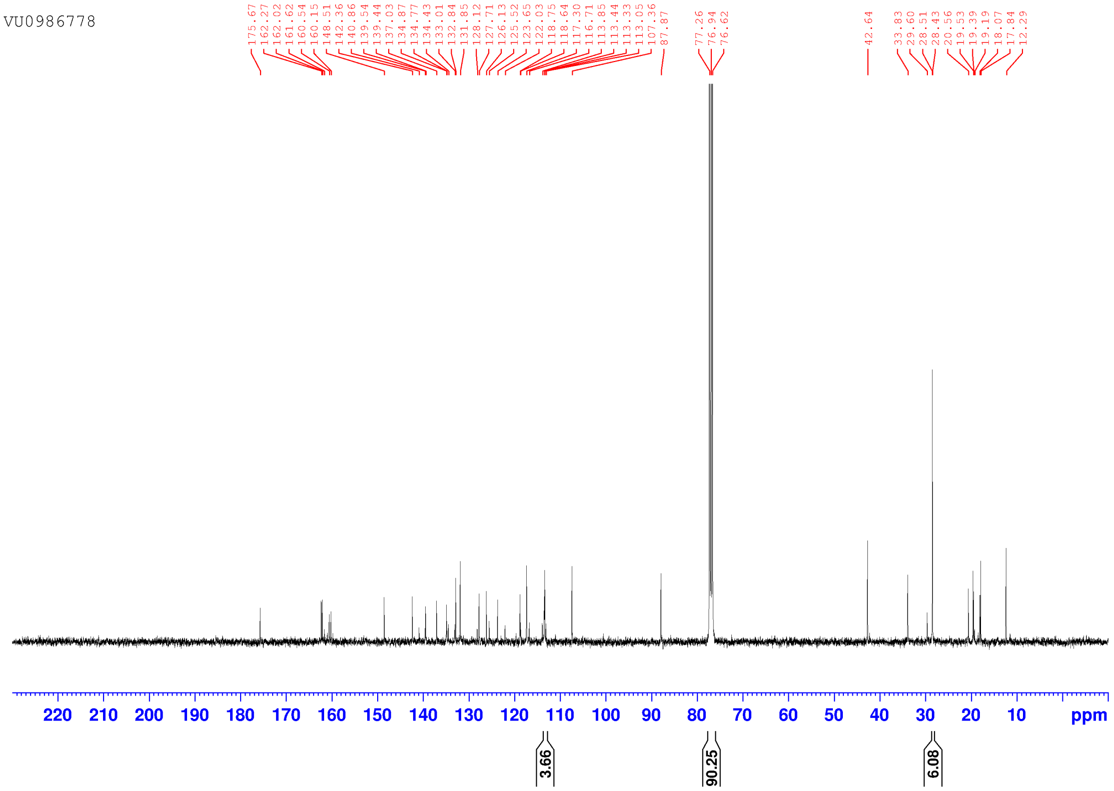

Supplement: Supplementary file 1 [file ml6c00272_si_001.pdf]
